# Enhancing Lithium Binding Affinity, Exchange Rate, and Hard–Soft Selectivity of Catenane Ligands by Post-Interlocking Modification

**DOI:** 10.1021/acs.inorgchem.6c02061

**Published:** 2026-08-10

**Authors:** Yueliang Yao, Mingda Hu, Hang Zhou, Samuel Kin-Man Lai, Ho Yu Au-Yeung

**Affiliations:** † Department of Chemistry, 25809 The University of Hong Kong, Pokfulam Road, Hong Kong SAR 999077, P. R. China; ‡ CAS-HKU Joint Laboratory on New Materials and State Key Laboratory of Synthetic Chemistry, The University of Hong Kong, Pokfulam Road, Hong Kong SAR 999077, P. R. China

## Abstract

A Li^+^-selective catenane host featuring a small, tetrahedral binding cavity lined with pyridine *N*-oxide motifs was synthesized through post-interlocking modification of a readily accessible bipyridine-derived catenane. The incorporation of *N*-oxide groups enhances binding strength and hard–soft selectivity by optimizing the hard–soft acid–base interactions with the hard Li^+^ ion, while the mechanically constrained catenane results in partially unsaturated coordination sites for Na^+^ and K^+^, further favoring Li^+^ binding. Exchange kinetics of Li^+^ binding are also accelerated with more rapid association/dissociation dynamics. The strong and dynamic Li^+^ binding enables the catenane to selectively extract the alkali metal from a solid mixture of alkali metal chlorides. Overall, the design underscores the potential of mechanically interlocked molecules with tailored cavities for selective alkali metal ion separation.

## Graphical abstract

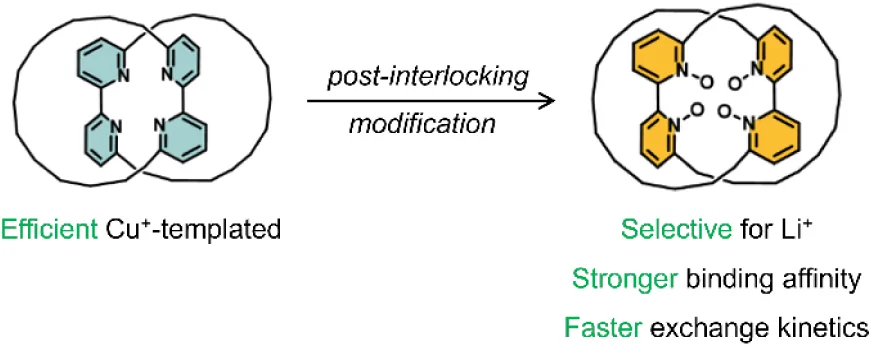

## Introduction

Lithium is an important metal resource in many everyday technologies, particularly in lithium-ion batteries that power electric vehicles, portable electronics, wearable devices, and energy storage systems.^1,2^ Molecular hosts that can bind to lithium ions (Li^+^) strongly and selectively not only are essential for the extraction, purification, and refinement of the metal in the manufacturing of high-quality battery materials, but also are important for the recovery and recycling of the metal resources from waste batteries and devices, thus marking a central role of lithium receptors in the long term and sustainable use of lithium resources.^3,4^ Chemically, lithium ions are hard metal ions that prefer oxygen-based ligands in a coordination number of 4, and their ligand exchange is generally fast.^5–7^ As a result, many of the well-studied chelators for Li^+^ are derived from crown ethers, ensuring good hard–soft matching in addition to strong binding affinity by virtue of the chelate and macrocyclic effects.^8–13^ Recently, an increasing number of heteroditopic ion-pair receptors containing complementary Li^+^ and anion (e.g., Cl^–^) binding sites have been developed for the selective extraction of lithium from mixtures of alkali-metal salts.^14–28^ Polymeric receptors, porous frameworks, and membrane systems have also been investigated for lithium separation, offering advantages in recyclability, processability, and continuous operation.^29–34^

Due to their unique topological structure, catenanes are an attractive class of mechanically interlocked molecules (MIMs) for developing selective molecular hosts.^35–43^ In addition to the strong binding from mechanical chelation, any bound guests in the interlocked cavity are also well-shielded from the external environment with strong kinetic stabilization.^44–48^ Host–guest complementarity can also be efficiently optimized via low-energy, large-amplitude co-conformational changes of the interlocked components.^49,50^ Furthermore, the mechanical interlocking could prohibit competitive guests from interacting with additional binding motifs except those available in the interlocked host, thereby further enhancing selectivity for the target guest. While these favorable binding features render catenanes a promising candidate for the development of lithium-selective receptors, the synthesis of many of the reported metal-binding catenanes is, in fact, templated by a transition metal ion (e.g., Cu^+^), and as such, their binding cavities are complementary to the original transition metal templates with strong affinity.^51–58^ Although a Li^+^-binding cavity could be created by using the small alkali metal ion as a template in the catenane synthesis,^59–64^ the need for specific lithium salts with large and hydrophobic counterions (e.g., fluorinated tetraarylborates) to ensure sufficient solubility, the weaker ion-dipole interactions involving the alkali metal ion, as well as the fast metal–ligand exchange, renders the synthesis not as general and efficient as that obtained from a transition metal template, not to mention the additional challenges in the precursor design to selectively accommodate the small Li^+^ ion.

In fact, the tetrahedral binding cavity in most catenanes obtained from passive Cu^+^ templates would also be efficient for Li^+^ binding, although they bind much more strongly to d^10^ metal ions such as Cu^+^ and Ag^+^ due to better hard–soft matching.^65,66^ As such, we propose that changing the borderline pyridine N donors to the harder O donors could switch the hard–soft acid–base preference, thereby weakening their binding to transition metals while enhancing the Li^+^ binding strength. Indeed, receptors containing *N*-oxide motifs have been previously reported for the selective binding and extraction of lithium cations and salts.^67^ Reported herein is a post-interlocking modification strategy to repurpose a bipyridine (bpy)-based catenane obtained from an efficient Cu^+^-templated synthesis for Li^+^ binding (Scheme 1). Upon oxidizing the bpy units to the corresponding bipyridine *N*,*N’*-dioxide, binding to soft metal ions is significantly inhibited, thereby reversing the hard–soft selectivity of the catenane host. In addition to the strengthening of the Li^+^ binding by an order of magnitude, the post-modification also enhances the Li^+^ binding kinetics, such that an efficient and selective solid–liquid extraction of solid LiCl from mixtures of alkali metal chlorides to an organic medium can be achieved. A preliminary version of this work was previously disclosed as a preprint.^68^

**Scheme 1:**
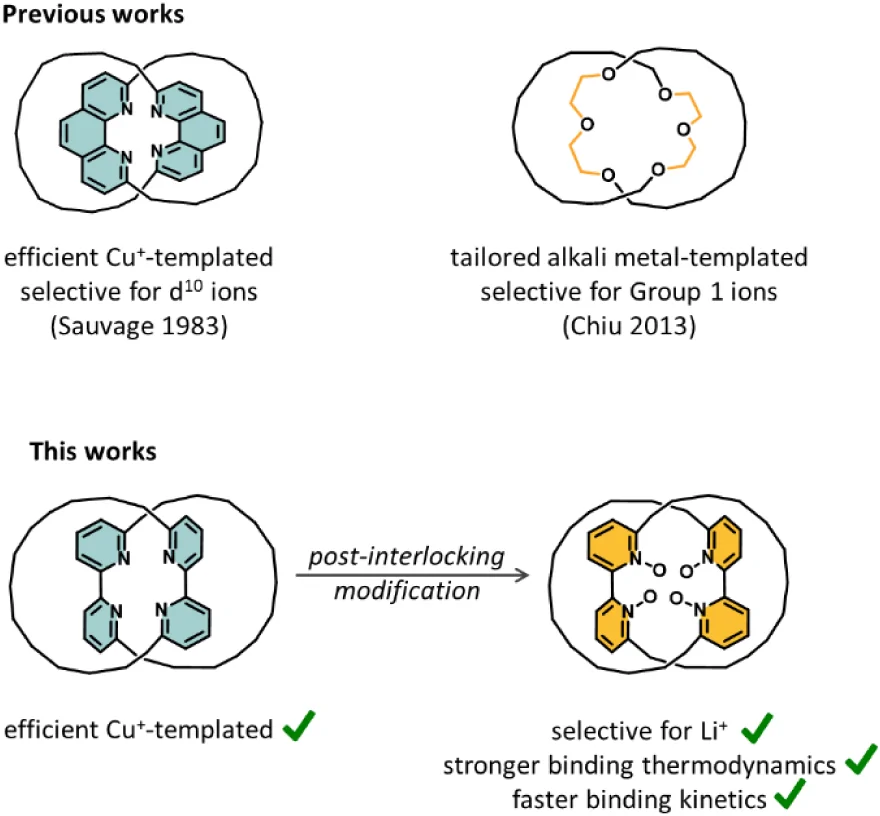
A Post-interlocking Modification Strategy to Convert a d^10^ Ion-Binding Catenane to a Li^+^-Selective Host

## Results and Discussion

Using a reported procedure for similar phenanthroline-derived catenanes,^69^ three bpy-based [2]­catenanes were efficiently obtained as their Cu­(I) complexes (Figure 1). The different lengths of the aliphatic linkers allowed various degrees of structural adjustment of the binding cavity for tuning the binding thermodynamics, kinetics, and selectivity. Prior to oxidizing the bpy units into *N*-oxides, the benzylic secondary amines were first converted to methanesulfonamides to avoid their oxidation, which would lead to the formation of a range of complicated oxidation products and potentially ring cleavage.^70,71^ Notably, the relatively small methanesulfonyl group was chosen to minimize its influence on the co-conformational flexibility and binding capability of the catenanes.^47^ Ethylenediamine was used to extract the Cu^+^ template,^72^ and three pyridine *N*-oxide catenanes, **Cn-NO** (n = 8, 10, and 12), were obtained by mCPBA oxidation (see Experimental Section for details). Although a similar catenane may be obtained from alkali metal-templated syntheses,^73^ ring-closing of pyridine oxide precursors via reductive amination with hydrides might not be chemically compatible, and the interlocking efficiency would be compromised.^39,69,74–81^ The post-modification strategy is therefore advantageous in ensuring a smooth introduction of the pyridine *N*-oxides without the need to develop a new catenane synthesis, and a similar strategy has been previously adopted by Evans and coworkers to obtain an interlocked host that contains *N*-oxides.^82–84^

**Figure 1:**
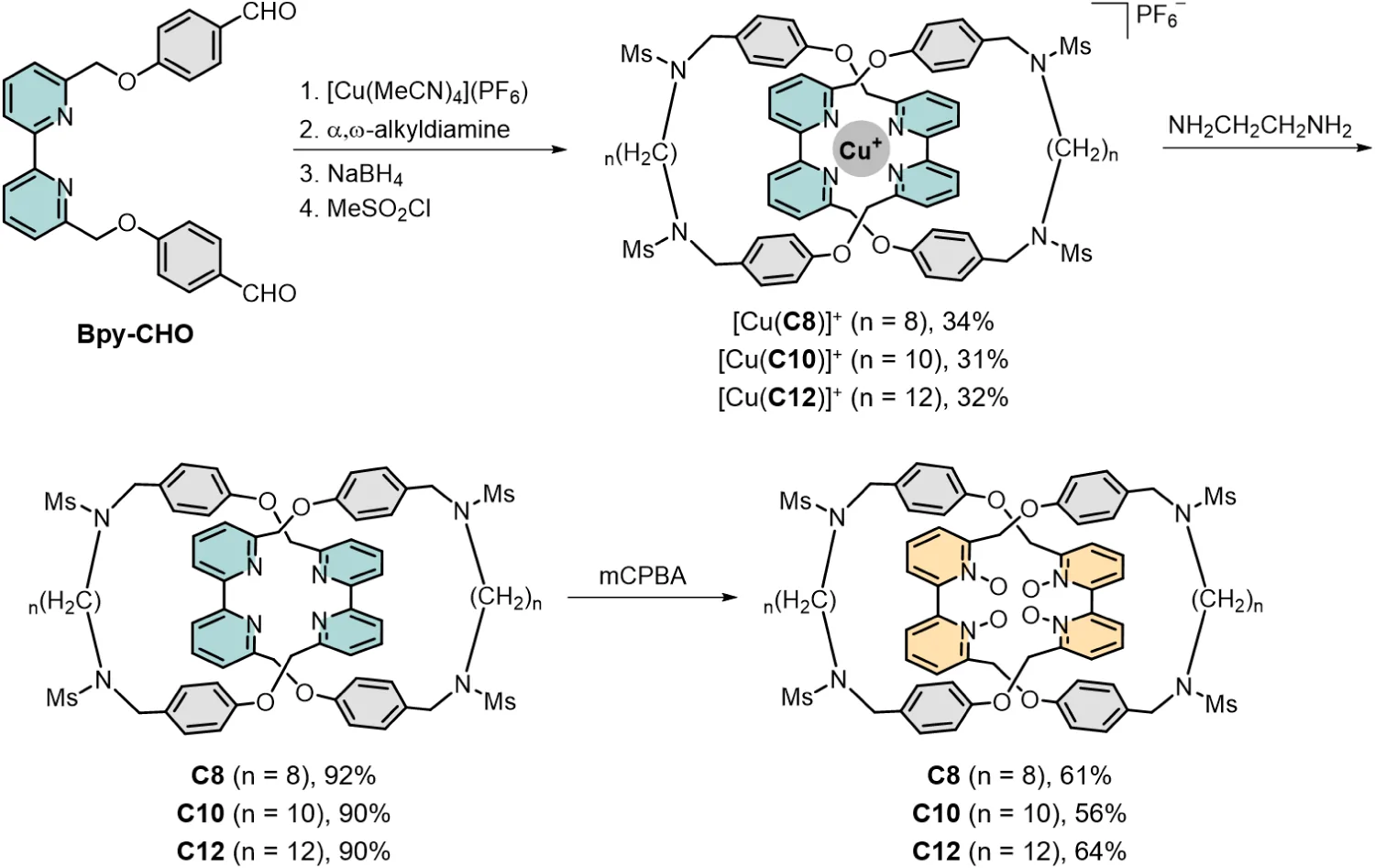
Synthesis and post-modifications of the catenane hosts.

The ^1^H NMR spectrum (500 MHz, 298 K, CD_3_CN) of **C12-NO** showed one set of sharp signals, suggesting that the catenane adopts a symmetrical structure in solution with a fast co-conformational exchange (Figure 2). On the other hand, splitting of the methylene singlets into two broader signals was observed in **C10-NO**, and multiple sets of signals were found for the smaller **C8-NO**. These observations suggest that the additional oxygen atoms and deplanarized bpy units may hinder the catenane from undergoing co-conformational exchange, such that co-conformers of lower symmetry are resolved. To avoid complications from the slow-exchanging co-conformers, binding studies were first focused on **C12-NO** with **C12** as the control host. Similar to other phenanthroline-based [2]­catenanes,^47,65^ **C12** was found to bind well to d^10^ ions including Cu^+^, Zn^2+^, and Ag^+^, and ^1^H NMR studies in CD_3_CN/CDCl_3_ (1:1 v/v) showed a complete conversion to the corresponding complexes upon the addition of 1 equiv. of the respective metal ions to a solution of **C12** (Figures S19–S21). Binding constants to Cu^+^ (*K* = 7.1 × 10^11^ M^–1^) and Ag^+^ (*K* = 1.4 × 10^10^ M^–1^) was determined by competitive exchange in CD_3_CN (see Supporting Information for details), and the large association constants obtained are consistent with the quantitative complex formation (Tables 1 and S1). Appreciable Li^+^ (as triflate) binding to **C12** was also observed, and two distinct sets of signals were found before saturation, showing a slow Li^+^ exchange relative to the NMR time scale (Figures 3 and S24). An association constant *K* = 1.1 × 10^5^ M^–1^ with a 1:1 binding stoichiometry was found for the Li^+^ binding to **C12** (Figure S26). Compared to the free host, an upfield shift of the proton signals of the phenyl linker (Δδ = 0.20–0.68 ppm) was observed for the Cu^+^, Zn^2+^, Ag^+^, and Li^+^ complexes, indicating an overall tightening of the catenane upon metal binding with a reinforced π-stacking of the phenyl bpy units.^85^ All the 1:1 metal-catenane complexes have been characterized by HR-ESI-MS (Figures S50–S53). Furthermore, weak Na^+^ (*K* = 460 M^–1^, as triflate salt) binding to **C12** was found, but the addition of KOTf showed no observable spectral change (Figures S25 and S27).

**Figure 2:**
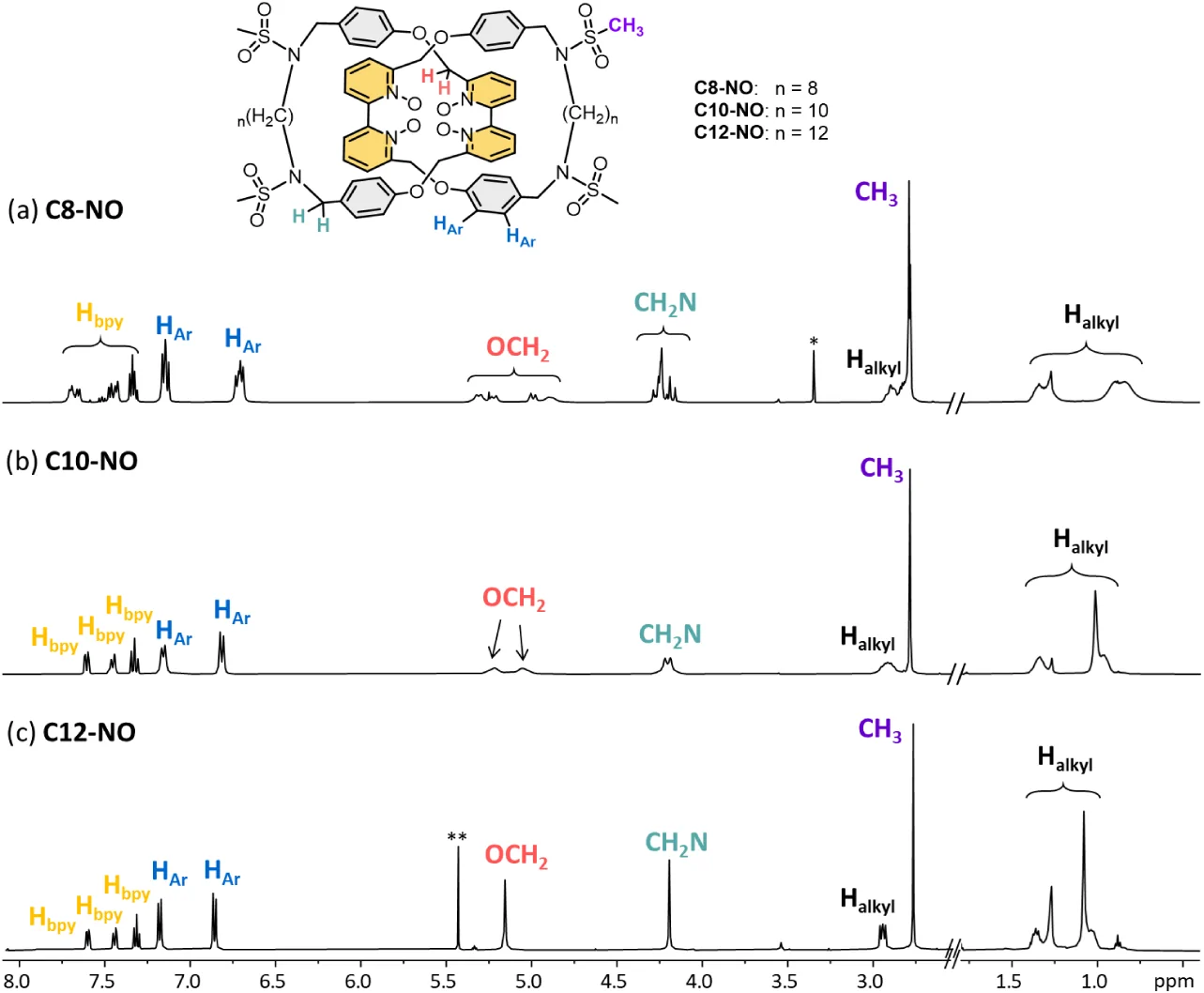
^1^H NMR (500 MHz, CD_3_CN, 298 K) spectra of (a) **C8-NO**; (b) **C10-NO**; and (c) **C12-NO**. Signals labeled with * and ** are due to residual MeOH and CH_2_Cl_2_, respectively.

**Table 1:** Binding Constant (M^–1^) of the 1:1 Binding at 298 K

	C12	C12-NO	C10-NO
Li^+^ ^a^	1.1 (±0.09) × 10^5^	4.5 (±0.2) × 10^5^	1.1 (±0.03) × 10^6^
Na^+^ ^a^	460 (±8)	5.1 (±0.1) × 10^3^	1.4 (±0.3) × 10^4^
K^+^ ^a^	no binding	210 (±4)	260 (±10)
Cu^+^ ^b^	7.1 (±0.1) × 10^11^	no binding	no binding
Ag^+^ ^c^	1.4 (±0.05) × 10^10^	110 (±4)	90 (±1)

a Triflate as an anion, titration conducted in CD_3_CN/CDCl_3_ (1:1 v/v).

b PF_6_^–^ as an anion, titration conducted in CD_3_CN.

c BF_4_^–^ as an anion, titration conducted in CD_3_CN.

**Figure 3:**
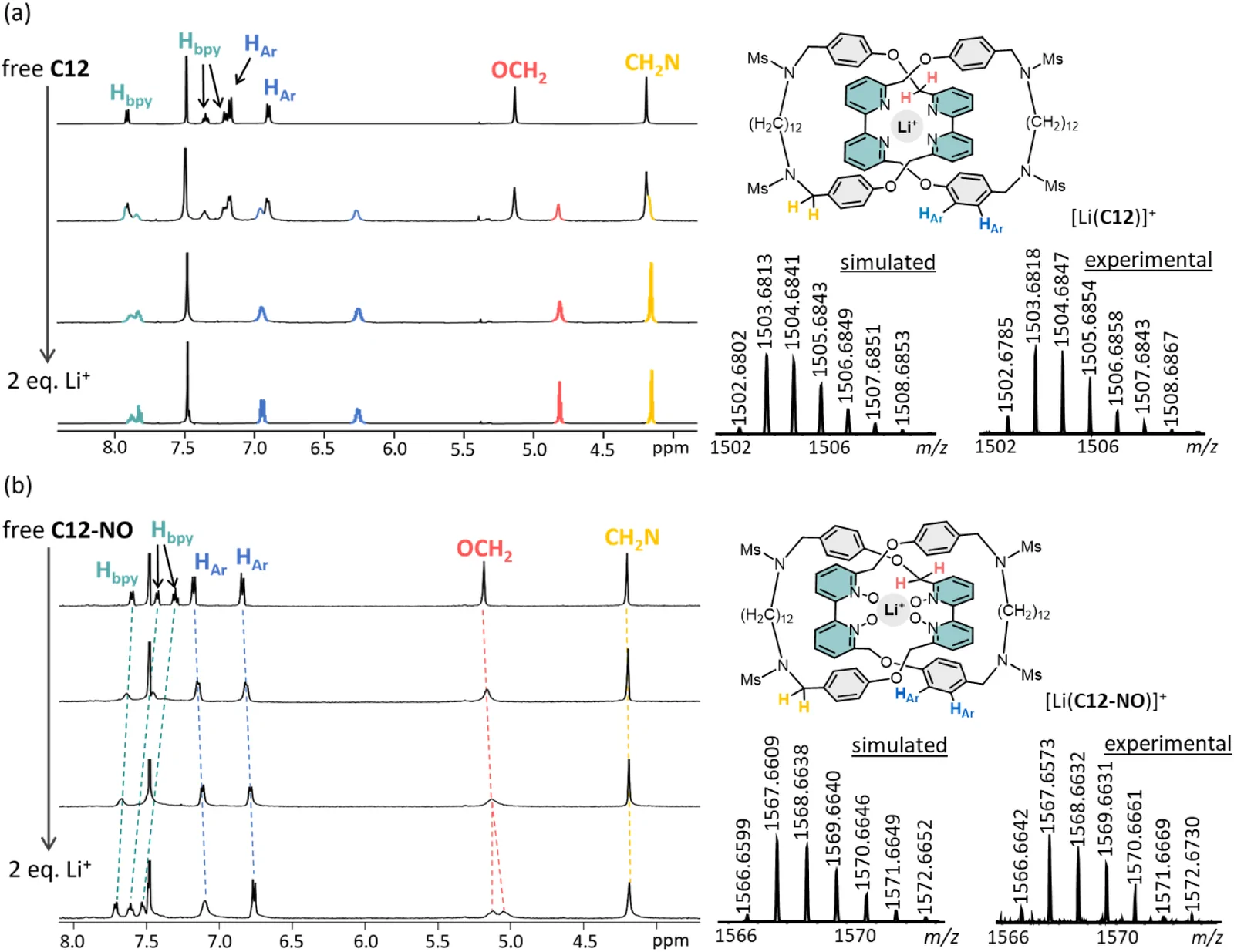
Left: partial ^1^H NMR spectra (500 MHz, 298 K, CD_3_CN/CDCl_3_ (1:1 v/v)) obtained upon titrating LiOTf to (a) **C12** (from top to bottom: 0, 0.4, 1, 2 equiv. Li^+^) and (b) **C12-NO** (from top to bottom: 0, 0.4, 0.8, 2 equiv. Li^+^). Right: isotopic pattern of the HR-ESI-MS (+ve) signal of the Li^+^ complexes. For **C12**, a new set of signals was observed (colored), indicating a slow exchange between **C12** and [Li­(**C12**)]^+^ under the NMR time scale. For C**12-NO**, gradual shifts of the proton signals were observed, indicating a fast exchange between **C12-NO** and [Li­(**C12-NO**)]^+^ under the NMR time scale.

Upon converting the bipyridines to the corresponding *N*,*N’*-dioxides, an enhanced Li^+^ binding was observed. Titration studies using **C12-NO** under the same conditions showed a 4-fold and 11-fold increase in the Li^+^ (*K* = 4.5 × 10^5^ M^–1^) and Na^+^ (*K* = 5100 M^–1^) binding constants, respectively, and weak binding was also found for K^+^ (*K* = 210 M^–1^). Apart from the enhanced binding strength, the post-modification also influences the Li^+^ binding kinetics. Gradual shifting of the proton signals was observed when LiOTf was titrated into a solution of **C12-NO** in CD_3_CN/CDCl_3_ (1:1 v/v), indicating a fast Li^+^ exchange with respect to the NMR time scale at room temperature (Figure 3). Further variable-temperature NMR studies of the equimolar mixture of **C12-NO** and its Li^+^ complex showed a coalescence temperature of 248 K, which is much lower than that of the parent **C12** (coalescence temperature = 328 K) (Figures S47 and S48). In addition to the downfield shifts of the bipyridyl protons that indicate metal coordination to the *N*-oxides, upfield shifts of the OCH_2_ signal and downfield shifts of the alkyl proton signals were also noted during the cation titration to **C12-NO** (Figures S36–S39). These spectral features are consistent with the alkali metal chelation by the *N*-oxides within the shielded catenane cavity, while the alkyl side chains are positioned at bipyridyl deshielding regions at the periphery.^49^ On the other hand, no spectral change was found when Cu^+^ was introduced into a solution of **C12-NO** (Figures S31 and S32), and Ag^+^ showed only very weak binding (*K* = 110 M^–1^ in CD_3_CN), consistent with a hard–soft selectivity reversal after the post-modification (Figures S34 and S35). Introduction of the borderline Zn^2+^ resulted in significant peak broadening of the proton signals of **C12-NO**, possibly due to the presence of multiple slow-exchanging species with nonspecific Zn^2+^ interactions (Figures S33 and S49). Another characteristic feature of catenane-based hosts is that the size, and hence host–guest complementarity, of the binding cavity can be efficiently tuned by simply changing the dimension of the interlocked macrocycles. A further increase in the binding strength by ∼2-fold was found for the Li^+^ (*K* = 1.1 × 10^6^ M^–1^) and Na^+^ (*K* = 1.4 × 10^4^ M^–1^) binding to the smaller **C10-NO**, while the K^+^ binding remains similarly weak (*K* = 260 M^–1^). While the Li^+^ binding could be further enhanced by using an even smaller catenane, following spectral changes involving **C8-NO** for binding constant determination is complicated by the complex ^1^H NMR spectrum of the free host, which exists as several slow-exchanging co-conformers, although a relatively well-resolved spectrum was obtained in the presence of excess LiOTf (Figure S46). While the structures of these catenanes are yet to be optimized, the good Li^+^ binding strength and selectivity of these catenanes highlight the strong potential of mechanical interlocking in the design of alkali metal receptors (Figure S54 and Table S2).^86–89^

Attempts to obtain suitable single crystals of the Li^+^-bound catenanes for X-ray crystallographic studies were unsuccessful, and the alkali metal ion binding by **C12-NO** was therefore further studied using density functional theory (DFT) calculations. Geometry optimization and single-point energy calculation of [M­(**C12-NO**)]^+^ (M^+^ = Li^+^, Na^+^, and K^+^) were carried out using the spin-restricted hybrid density functional B3LYP and 6-31G­(d,p) basis set.^90–92^ Consistent with the expected 4-coordination, the Li^+^ ion was found to be chelated by the two interlocked rings via the four O donors of the bipyridine *N*,*N’*-dioxide, with Li–O distances ranging from 1.907 Å to 1.948 Å. A calculated binding constant of 5.1 × 10^5^ M^–1^ was found for the Li^+^ binding, which is comparable to that determined from titration. Energy minimization of the corresponding Na^+^ and K^+^ complexes was also performed, and the obtained stability constants (Na^+^: 3800 M^–1^; K^+^: 150 M^–1^) also agree with the experimental values. A closer analysis of the Na^+^ and K^+^ coordination environment in the energy-minimized structures showed that the two bpy *N*,*N*’-dioxide units are not orthogonally oriented, with one of the interlocked rings rotated sideways so that the larger alkali metal ions are in close proximity to an ether oxygen with a 5-coordination (Figure 4). While the larger alkali metal ions would strive for additional coordination donors from external ligands to fulfill a higher coordination number, the required co-conformational change may not be favorable for catenanes with tight interlocking,^47^ limiting the feasibility for Na^+^ and K^+^ to attain their optimal coordination via formation of any ternary (or higher-order) complexes with external ligands, and thereby achieving the observed Li^+^ selectivity.^93,94^

**Figure 4:**
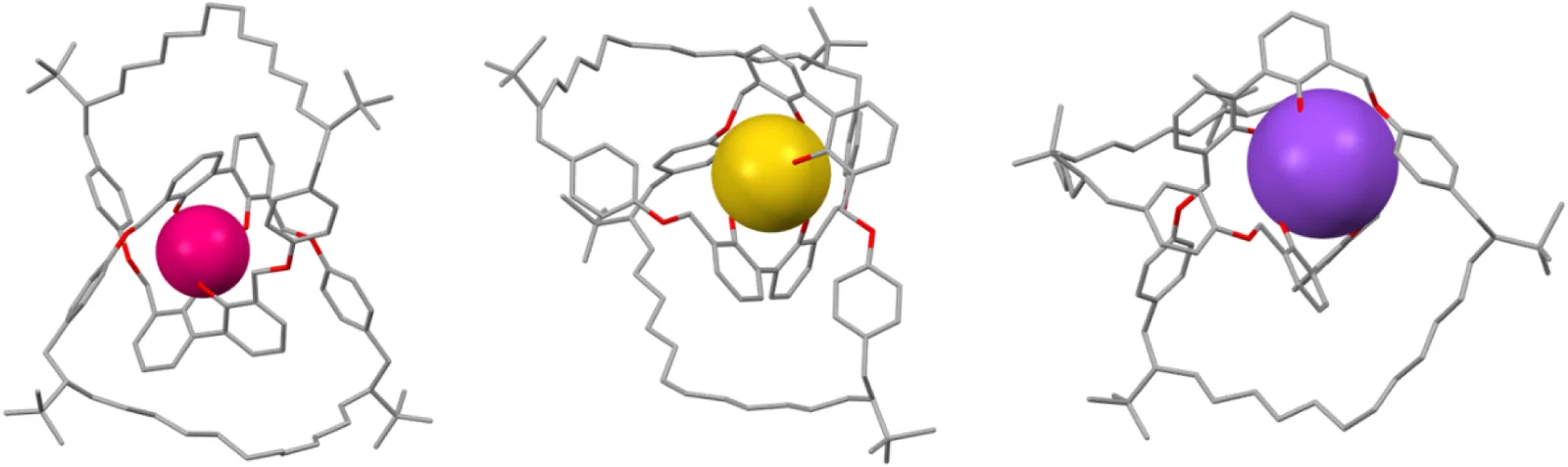
DFT-optimized structures of Li^+^ (left), Na^+^ (middle), and K^+^ (right) complexes of **C12-NO** (oxygen atoms from *N*-oxides and ethers are colored red). Hydrogen atoms are omitted for clarity.

Further to the solution-phase binding, the ability of the interlocked hosts to extract lithium from mixtures of naturally abundant alkali metal chlorides was also investigated through solid–liquid extraction experiments. A 100:100:100 equimolar mixture of solid LiCl, NaCl, and KCl was treated with a 1 mM **C12-NO** solution in CHCl_3_ (i.e., 1 mol % relative to NaCl) at room temperature for 12 h, after which the inorganic solids were filtered, and the catenane-bound metals were back-extracted into an aqueous solution for ICP-MS quantification. The extraction efficiency was calculated and expressed as the number of moles of M^+^ extracted per 1 mol of the catenane. As shown in Figure 5b, exclusive lithium extraction, with an extraction efficiency of 0.72, was observed when **C12-NO** was used as the extractant. Although decreasing the amount of LiCl in the solid mixture to LiCl/NaCl/KCl = 1:100:100—a model condition for Li^+^ extraction under a competitive high-sodium environment,^95–101^ led to a decrease in the Li^+^ extraction efficiency to 0.44, good lithium selectivity was maintained, and no statistically significant amount of Na^+^ or K^+^ was detected. ^1^H NMR experiments of a sample of **C12-NO** in CD_3_CN/CDCl_3_ (1:1 v/v) in the presence of solid LiCl or LiCl/NaCl/KCl (100:100:100 or 1:100:100 mixtures) also showed complete conversion to the Li^+^-bound catenane complex, and the Na^+^ complex was not detected, supporting the selective extraction of lithium into the organic medium by the catenane host (Figure S61). Of note, no distinguishable spectral features were found in the ^1^H NMR spectra of the Li^+^ complexes of **C12-NO** prepared from LiCl or LiOTf, supporting that the Li^+^ coordination sphere in the catenane complexes is not accessible to the counteranions. In the solid–liquid extraction experiment, the counteranion is likely present as an ion-pair with the cationic catenane complex and/or in solvated forms in the organic medium, but the structures of the anion-associated species will need to be further characterized. In fact, recent examples of molecular hosts capable of solid–liquid extraction of alkali metal chlorides are mostly ion-pair hosts designed with both cation and anion binding motifs.^20–30,64^ The ability of **C12-NO** to overcome the high lattice energy of LiCl and extract the inorganic salt into an organic medium is therefore noteworthy.^102^ In contrast, control experiments using the unmodified catenane **C12** as the extractant showed no effective lithium extraction (efficiency ≈ 0.02) from the alkali metal chloride mixtures. Considering that the Li^+^ binding affinity of **C12-NO** and **C12** differs by only ∼4-fold, the much higher lithium extraction efficiency (>30-fold) displayed by **C12-NO** compared to **C12** likely suggests a distinctive Li^+^ binding mechanism and kinetics for the two catenanes. Due to the low solubility of LiCl in chloroform, the concentration of free, solvated forms of Li^+^ is also anticipated to be very low. As such, mixtures of alkali metal triflates, which are more soluble in chloroform, were used to replace the chlorides to investigate the potential influence of solvated Li^+^ on the extraction. A much-improved lithium extraction efficiency was observed for **C12** (i.e., 0.36 and 0.23, respectively, from 100:100:100 and 1:100:100 LiOTf/NaOTf/KOTf), and the efficiency of **C12-NO** (i.e., 0.77 and 0.48) was found to be similar to the case when chlorides were used (Figure 5c). These findings suggest that the catenane post-modification may also facilitate the solvation/binding of the cation in the solid salts, thereby resulting in good extraction efficiency. Nevertheless, further ^1^H NMR studies of a 1 mM CDCl_3_ solution of **C12-NO** in the presence of 100 equiv. each of LiCl, NaCl, and KCl to mimic the solid–liquid extraction revealed that Li^+^ binding can be completed within 1 h at room temperature, while using **C12** under the same conditions showed no observable Li^+^ binding even after heating at 50 °C for 12 h (Figures S63 and S64), confirming the very different binding kinetics of the two hosts. These results not only demonstrate the potential of our catenanes as a host and extractant for Li^+^, but also highlight the importance of binding kinetics in host design when applications are concerned, which is usually a less-considered aspect compared to binding strength and selectivity.

**Figure 5:**
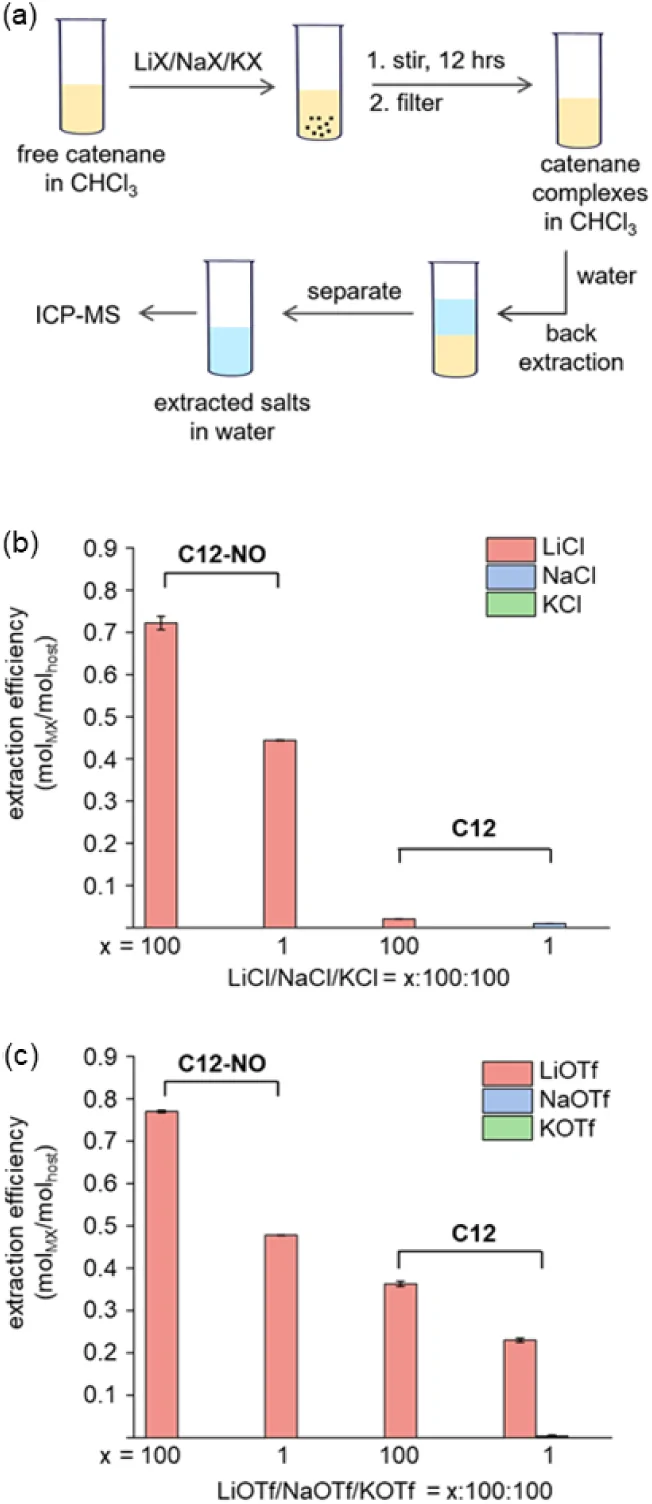
(a) A scheme showing the solid–liquid extraction of alkali metals, and the extraction efficiency by the catenane hosts for the solid–liquid extraction of (b) alkali metal chlorides and (c) alkali metal triflates by **C12-NO** and **C12**.

## Conclusion

In summary, a post-interlocking modification strategy for the facile conversion of bipyridine-based catenanes obtained from an efficient Cu^+^-template synthesis to the corresponding *N*,*N’*-dioxides for strong, selective, and dynamic Li^+^ binding is described. Binding studies revealed a hard–soft selectivity reversal after the post-modification, which not only inhibits the binding to soft Cu^+^ and Ag^+^ ions but also achieves a concomitant strengthening of the Li^+^ binding by an order of magnitude. Furthermore, the restricted coordination environment imposed by the mechanical interlocking enforces the corresponding Na^+^ and K^+^ complexes to remain unsaturated and renders a high Li^+^ selectivity. Apart from the enhanced binding strength and hard–soft selectivity, the post-modification also improves the Li^+^ binding kinetics, enabling an efficient and selective extraction of solid LiCl over NaCl. These results also highlight the importance of kinetic considerations in the design of practical supramolecular hosts. Further studies on optimizing the catenane structure, evaluating the catenane hosts for liquid–liquid extraction, improving the extraction efficiency, and incorporating the catenane hosts into materials for the refining, extraction, recycling, and management of lithium resources under practical conditions are currently underway.

## Experimental Section

### General Procedures

All reagents (purity ≥98%, unless otherwise noted) were purchased from commercial suppliers (J&K, Sigma-Aldrich, TCI, Aladdin, Bide, Energy, Macklin, Leyan, and Dkmchem) and used without further purification, unless otherwise noted. All the solvents were of analytical grade (ACI Labscan and DUKSAN Pure Chemicals). Deuterated solvents were purchased from Energy, Leyan, and J&K (≥99.8 atom % D). **Bpy-CHO** was synthesized according to literature procedures.^85^ Thin-layer chromatography (TLC) was performed on silica gel 60 F254 (Merck, Germany, aluminum sheet), and column chromatography was carried out using silica gel 60F (Silicycle, Canada). HR-ESI-MS spectra were obtained from a Bruker Impact II Ultra-High Resolution QTOF mass spectrometer. NMR spectra were recorded on Bruker DPX spectrometers with working frequencies of 400 MHz, 500 MHz, or 600 MHz for ^1^H, and 100 MHz or 125 MHz for ^13^C, respectively. Chemical shifts were reported in ppm and referenced to solvent residues. No uncommon hazards are noted for any of the syntheses and measurements.

### Synthesis of [Cu­(**Cn**)]­(PF_6_) (n = 8, 10, 12)

Under an argon atmosphere, a mixture of **Bpy-CHO** (255 mg, 0.6 mmol) and [Cu­(MeCN)_4_]­(PF_6_) (112 mg, 0.30 mmol) in CHCl_3_/MeCN/MeOH (5:3:2 v/v/v, 200 mL) was heated at 50 °C for 1 h. To the red solution obtained, an appropriate α,ω-diamine (0.9 mmol) and piperidine (0.5 mL) were added, and the reaction mixture was heated to 60 °C overnight. The reaction mixture was cooled in an ice bath, and NaBH_4_ (45 mg, 1.2 mmol) was added. The mixture was stirred while cooled in the melting ice bath for 1 h. Solvents were removed by a rotary evaporator, and the residue was redissolved in CH_2_Cl_2_ (50 mL), washed with water (2 × 25 mL) and brine (25 mL), dried over anhydrous Na_2_SO_4_ and filtered. Solvents were removed from the filtrate using a rotary evaporator, and the residue was washed with diethyl ether (2 × 15 mL). The obtained red solid was redissolved in MeCN (30 mL) and filtered, and solvents were removed from the filtrate. The crude Cu­(I)-[2]­catenane complex obtained was redissolved in CH_2_Cl_2_ (20 mL), and triethylamine (300 mg, 3.0 mmol) was added. The mixture was cooled in an ice bath, and a solution of methanesulfonyl chloride (345 mg, 3.0 mmol) in CH_2_Cl_2_ (10 mL) was slowly added, followed by the addition of 4-dimethylaminopyridine (2 mg, 0.016 mmol). The reaction mixture was stirred at room temperature for 2 h. A saturated aqueous NaHCO_3_ solution (5 mL) was added to the mixture. The organic phase was separated, washed with water (2 × 10 mL), and dried over anhydrous Na_2_SO_4_. Insoluble materials were removed by filtration, and solvents were removed using a rotary evaporator to afford a red solid, which was washed with diethyl ether (10 mL) and purified on a silica column (CH_2_Cl_2_/MeOH, 20:1 v/v) to give the Cu­(I) catenane complex [Cu­(**Cn**)]­(PF_6_) (n = 8, 10, 12).

[Cu­(**C8**)]­(PF_6_). Red solid. Yield = 163 mg, 34%. ^1^H NMR (500 MHz, CD_3_CN, 298 K) δ 7.92 (t, *J* = 7.8 Hz, 4H), 7.69 (d, *J* = 7.4 Hz, 4H), 7.65 (d, *J* = 8.0 Hz, 4H), 6.86 (d, *J* = 8.6 Hz, 8H), 6.13 (d, *J* = 8.6 Hz, 8H), 4.74 (s, 8H), 4.17 (s, 8H), 3.13–3.05 (m, 8H), 1.63 (dt, *J* = 15.1, 7.6 Hz, 8H), 1.48–1.42 (m, 8H), 1.41–1.34 (m, 8H). ^13^C­{^1^H} NMR (125 MHz, CD_3_CN, 298 K) δ 158.0, 155.1, 152.3, 139.8, 130.8, 129.4, 127.9, 121.9, 114.1, 71.9, 51.0, 48.8, 38.8, 29.7, 28.6, 27.6. ESI-HRMS (+ve) found: 1447.4682; calcd. for C_72_H_88_N_8_O_12_S_4_Cu^+^ [M]^+^: 1447.4696.

[Cu­(**C10**)]­(PF_6_). Red solid. Yield = 153 mg, 31%. ^1^H NMR (500 MHz, CD_3_CN, 298 K) δ 7.96 (t, *J* = 7.8 Hz, 4H), 7.90 (d, *J* = 8.0 Hz, 4H), 7.70 (d, *J* = 7.4 Hz, 4H), 6.93 (d, *J* = 8.4 Hz, 8H), 6.23 (d, *J* = 8.4 Hz, 8H), 4.77 (s, 8H), 4.17 (s, 8H), 3.14–3.05 (m, 8H), 2.84 (s, 12H), 1.63–1.51 (m, 8H), 1.39 (s, 16H), 1.36–1.29 (m, 8H). ^13^C­{^1^H} NMR (125 MHz, CD_3_CN, 298 K) δ 158.0, 155.5, 152.3, 139.9, 131.0, 129.8, 127.3, 122.2, 114.3, 71.8, 51.1, 48.8, 38.5, 29.8, 29.5, 28.8, 27.5. ESI-HRMS (+ve) found: 1503.5313; calcd. for C_76_H_96_N_8_O_12_S_4_Cu^+^ [M]^+^: 1503.5322.

[Cu­(**C12**)]­(PF_6_). Red solid. Yield = 164 mg, 32%. ^1^H NMR (500 MHz, CD_3_CN, 298 K) δ 8.05–7.98 (m, 8H), 7.72 (dd, *J* = 7.4, 0.9 Hz, 4H), 6.98 (d, *J* = 8.6 Hz, 8H), 6.28 (d, *J* = 8.7 Hz, 8H), 4.79 (s, 8H), 4.17 (s, 8H), 3.12–3.04 (m, 8H), 2.84 (s, 12H), 1.56–1.46 (m, 8H), 1.34 (s, 32H). ^13^C­{^1^H} NMR (125 MHz, CD_3_CN, 298 K) δ 158.0, 155.9, 152.4, 140.0, 131.1, 130.1, 126.8, 122.4, 114.5, 71.6, 51.2, 48.7, 38.5, 30.0, 29.8, 29.6, 28.8, 27.4. ESI-HRMS (+ve) found: 1559.5923; calcd. for C_80_H_104_N_8_O_12_S_4_Cu^+^ [M]^+^: 1559.5948.

### Synthesis of Cn (n = 8, 10, 12)

To a solution of [Cu­(**Cn**)]­(PF_6_) (0.06 mmol) in CH_2_Cl_2_ (10 mL), ethylenediamine (2 mL, 30 mmol) was added, and the mixture was stirred at room temperature for 5 min. Deionized water (5 mL) was added, and the yellow organic phase was separated. Another aliquot of ethylenediamine (1 mL, 15 mmol) was added to the organic solution and stirred for another 5 min to give a colorless solution. CH_2_Cl_2_ (5 mL) and deionized water (5 mL) were added, and the organic phase was separated, washed with water (2 × 5 mL), and dried over anhydrous Na_2_SO_4_. Insoluble materials were removed by filtration, and the solvents were removed using a rotary evaporator to afford a white solid. The solid obtained was further washed with hexanes and dried to give **Cn** (n = 8, 10, 12).

**C8**. White solid. Yield = 76 mg, 92%. ^1^H NMR (400 MHz, DMSO*-d*_6_, 298 K) δ 7.65–6.86 (m, 28H), 5.20 (s, 8H), 4.16 (s, 8H), 2.89 (s, 12H), 2.60 (s, 8H), 1.06 (s, 8H), 0.26 (s, 16H). ^13^C­{^1^H} NMR (100 MHz, DMSO*-d*_6_, 298 K) δ 157.4, 156.0, 154.0, 136.9, 129.2, 129.0, 122.1, 119.4, 115.3, 70.1, 49.5, 46.3, 38.4, 28.8, 27.3, 26.1. ESI-HRMS (+ve) found: 1385.5478; calcd. for C_72_H_89_N_8_O_12_S_4_^+^ [M + H]^+^: 1385.5477.

**C10**. White solid. Yield = 78 mg, 90%. ^1^H NMR (400 MHz, DMSO*-d*_6_, 298 K) δ 7.78 (d, *J* = 7.7 Hz, 4H), 7.35 (d, *J* = 7.7 Hz, 4H), 7.25 (d, *J* = 7.6 Hz, 4H), 7.15 (d, *J* = 8.6 Hz, 8H), 6.93 (d, *J* = 8.6 Hz, 8H), 5.17 (s, 8H), 4.13 (s, 8H), 2.88 (s, 12H), 2.79–2.69 (m, 8H), 1.16 (s, 8H), 0.70–0.51 (m, 24H). ^13^C­{^1^H} NMR (100 MHz, DMSO*-d*_6_, 298 K) δ 157.4, 156.3, 154.3, 137.3, 129.3, 129.1, 121.7, 119.4, 115.0, 70.4, 49.8, 46.9, 38.1, 28.7, 28.4, 27.5, 26.2. ESI-HRMS (+ve) found: 1441.6098; calcd. for C_76_H_97_N_8_O_12_S_4_^+^ [M + H]^+^: 1441.6103.

**C12**. White solid. Yield = 81 mg, 90%. ^1^H NMR (500 MHz, CD_3_CN, 298 K) δ 7.91 (d, *J* = 7.6 Hz, 4H), 7.42 (t, *J* = 7.8 Hz, 4H), 7.24 (d, *J* = 7.5 Hz, 4H), 7.19 (d, *J* = 8.7 Hz, 8H), 6.93 (d, *J* = 8.7 Hz, 8H), 5.14 (s, 8H), 4.19 (s, 8H), 2.95–2.84 (m, 8H), 2.78 (s, 12H), 1.28 (s, 8H), 0.95–0.74 (m, 32H). ^13^C­{^1^H} NMR (100 MHz, CD_3_CN, 298 K) δ 158.9, 157.6, 155.8, 138.4, 130.6, 130.4, 122.4, 120.5, 116.1, 71.7, 51.3, 48.5, 38.6, 30.2, 30.0, 29.8, 28.9, 27.5. ESI-HRMS (+ve) found: 1497.6722; calcd. for C_80_H_105_N_8_O_12_S_4_^+^ [M + H]^+^: 1497.6729.

### Synthesis of **Cn-NO** (n = 8, 10, 12)

A solution of **Cn** (0.02 mmol, n = 8, 10, 12) in CH_2_Cl_2_ (5 mL) was cooled in an ice bath, and a solution of *meta*-chloroperoxybenzoic acid (mCPBA, purity ∼85%, 100 mg, 0.49 mmol) in CH_2_Cl_2_ (10 mL) was added slowly. The mixture was stirred at room temperature for 24 h, and a saturated aqueous K_2_CO_3_ solution (5 mL) was added. The organic phase was separated, washed with water (2 × 10 mL), and dried over anhydrous Na_2_SO_4_. Insoluble materials were removed by filtration, and solvents were removed from the filtrate using a rotary evaporator to afford a white solid. The obtained solid was washed with diethyl ether (10 mL) and purified by preparative thin-layer chromatography (CH_2_Cl_2_/MeOH, 20:1 v/v) to give **Cn-NO** (n = 8, 10, 12).

**C8-NO**. White solid. Yield = 18 mg, 61%. ^1^H NMR (500 MHz, CD_3_CN, 298 K) δ 7.74–7.62 (m, 4H), 7.56–7.40 (m, 4H), 7.37–7.30 (m, 4H), 7.19–7.11 (m, 8H), 6.79–6.65 (m, 8H), 5.38–5.19 (m, 4H), 5.06–4.80 (m, 4H), 4.32–4.13 (m, 8H), 2.98–2.71 (m, 20H), 1.44–1.17 (m, 8H), 1.03–0.69 (m, 16H). ^13^C­{^1^H} NMR (125 MHz, CD_3_CN, 298 K) δ 159.1, 148.0, 144.7, 144.5, 130.4, 130.3, 128.0, 127.9, 127.3, 124.7, 116.0, 115.8, 65.5, 50.9, 48.1, 39.1, 30.2, 30.1, 28.9, 27.5. ESI-HRMS (+ve) found: 1449.5269; calcd. for C_72_H_89_N_8_O_16_S_4_^+^ [M + H]^+^: 1449.5274.

**C10-NO**. White solid. Yield = 17 mg, 56%. ^1^H NMR (400 MHz, CD_3_CN, 298 K) δ 7.61 (dd, *J* = 7.8, 1.6 Hz, 4H), 7.45 (d, *J* = 7.1 Hz, 4H), 7.33 (t, *J* = 7.8 Hz, 4H), 7.16 (d, *J* = 7.9 Hz, 8H), 6.81 (d, *J* = 8.4 Hz, 8H), 5.13 (d, *J* = 64.7 Hz, 8H), 4.20 (d, *J* = 12.5 Hz, 8H), 2.99–2.85 (m, 8H), 2.78 (s, 12H), 1.34 (s, 8H), 1.08–0.90 (m, 24H). ^13^C­{^1^H} NMR (125 MHz, CD_3_CN, 298 K) δ 158.9, 148.0, 144.4, 130.9, 130.5, 128.0, 126.9, 124.8, 115.9, 65.4, 51.2, 48.5, 38.8, 30.2, 29.9, 29.1, 27.8. ESI-HRMS (+ve) found: 1505.5852; calcd. for C_76_H_97_N_8_O_16_S_4_^+^ [M + H]^+^: 1505.5900.

**C12-NO**. White solid. Yield = 20 mg, 64%. ^1^H NMR (500 MHz, CD_3_CN, 298 K) δ 7.62 (dd, *J* = 7.8, 1.8 Hz, 4H), 7.46 (dd, *J* = 7.8, 1.8 Hz, 4H), 7.33 (t, *J* = 7.8 Hz, 4H), 7.20 (d, *J* = 8.6 Hz, 8H), 6.87 (d, *J* = 8.7 Hz, 8H), 5.17 (s, 8H), 4.21 (s, 8H), 3.04–2.92 (m, 8H), 2.78 (s, 12H), 1.40–1.33 (m, 8H), 1.12–1.00 (m, 32H). ^13^C­{^1^H} NMR (125 MHz, CD_3_CN, 298 K) δ 158.7, 148.2, 144.2, 131.2, 130.6, 127.8, 126.2, 125.0, 115.9, 65.3, 51.4, 48.7, 38.5, 30.5, 30.2, 30.0, 29.3, 27.9. ESI-HRMS (+ve) found: 1561.6541; calcd. for C_80_H_105_N_8_O_16_S_4_^+^ [M + H]^+^: 1561.6526.

### Cation Binding Studies in Solution

The cation association constants were determined by ^1^H NMR titration experiments.^103^ In a typical ^1^H NMR titration experiment, aliquots of 50 mM solutions of a guest in CD_3_CN/CDCl_3_ (1:1 v/v) or CD_3_CN were sequentially added to a 1 mM solution of the catenane host (500 μL) at 298 K in the same solvent, and mixed well before an ^1^H NMR spectrum was collected on a Bruker DPX spectrometer with a working frequency of 500 MHz. Details are provided in the Supporting Information.

### Solid–Liquid Extraction Studies

In solid–liquid extraction experiments, solid mixtures of LiX, NaX, and KX (X^–^ = Cl^–^ or OTf^–^) in 100:100:100 and 1:100:100 molar ratios were treated with a 1 mol % solution (relative to NaX or KX) of **C12-NO** or **C12** in CHCl_3_ (1500 μL, 1 mM). The mixtures were stirred at room temperature for 12 h (rpm = 1000). The mixture was allowed to stand at ambient conditions for 30 min after the stirring ceased, and the organic phase was collected using a syringe and filtered through a 0.22 μm filter membrane. The filtrate (1000 μL) was placed in a new vial, and deionized water (1000 μL) was added. The alkali metal salt was back-extracted to the aqueous phase, which was collected and diluted by 40- to 200-fold before the metal content was analyzed by an Agilent 7700x Inductively Coupled Plasma Mass Spectrometry (ICP-MS), with 1550 W forward RF power, 2 W reflected RF power, 1.05 L/min carrier gas flow, 10 mm sample depth, spray chamber cooled to 2 °C, and a nebulizer pump speed of 0.1 rps. Control experiments using the chloride salts in the absence of catenane hosts were conducted following the same procedures described above.

The solid–liquid extraction of **C12-NO** was further studied by ^1^H NMR analysis. Solid LiCl and a solid mixture of LiCl, NaCl, and KCl (100:100:100 or 1:100:100 molar ratio) were respectively treated with a 1 mol % solution of C12-NO (relative to the salt) **C12-NO** in CDCl_3_ (1500 μL, 1 mM). The resulting mixtures were sonicated for 30 min at room temperature and filtered through a 0.22-μm membrane. Samples for ^1^H NMR analysis were prepared by the addition of CD_3_CN to an equal volume of the filtrate. For the time-dependent ^1^H NMR studies to monitor the extraction process, a sample of 1 mM **C12-NO** or **C12** in CDCl_3_ (500 μL) in the presence of solid LiCl, NaCl, and KCl (1:1:1, 100 equiv. each) was prepared and analyzed by ^1^H NMR at different time intervals.

### ICP-MS Measurement

For each ICP-MS measurement, diluted samples of the ^7^Li, ^23^Na, and ^39^K standard solutions were freshly prepared at 10 μg/L, 20 μg/L, 40 μg/L, 60 μg/L, 80 μg/L, 100 μg/L, and 120 μg/L by serial dilution with deionized water (Figures S55–S60). The limit of detection (LoD) was determined using the 3σ method. The concentration of ^7^Li, ^23^Na, and ^39^K in the aqueous samples obtained from the solid–liquid extraction was calculated using the linear relation obtained from the calibration curves. Each sample was spiked with the corresponding metal at 10 μg/L and 20 μg/L, and each measurement was performed in triplicate.

### Computational Studies on Cation Binding of **C12** and **C12-NO**

The initial configurations and visualization of optimized structures for **C12**, **C12-NO**, [M­(**C12**)]^+^, and [M­(**C12-NO**)]^+^ (M^+^ = Li^+^, Na^+^, and K^+^) were generated using the Avogadro software.^104^ The resulting empirically optimized configurations were further refined using density functional theory calculations performed with Gaussian 16 (revision A.03).^105^ Geometry optimizations and single-point energy calculations were carried out using the spin-restricted hybrid density functional B3LYP,^90–92^ and the 6-31G­(d,p) basis set was employed for all atoms. The solvation effects of CH_3_CN (*ε* = 35.688) and CHCl_3_ (*ε* = 4.7113) were taken into account using the conductor-like polarizable continuum model (CPCM).^106,107^ During the structural optimization step, a convergence criterion of 10^–8^ for the density matrix was applied. Vibrational analyses were conducted to validate the optimized structures.

## Supplementary Material



## Notes

NMR spectra of the synthesized catenanes and cation complexes; experimental details of binding studies in the solution state, solid–liquid extraction, and computational studies; Job plots and fittings of NMR titration data; HR-ESI-MS isotopic patterns of the catenane-cation complexes; examples of literature-reported macrocyclic hosts for alkali metal cation binding; calculated extraction efficiency of solid–liquid extractions, calibration curves, and results of the ICP-MS measurement; DFT-optimized structures, parameters, and Cartesian coordinates of the cation complexes of **C12** and **C12-NO** (PDF)
