# Supplementary material for: Enhancing Lithium Binding Affinity, Exchange Rate, and Hard–Soft Selectivity of Catenane Ligands by Post-Interlocking Modification
Source: Inorg Chem. 2026 Aug 10;65(33):19194–204. doi: 10.1021/acs.inorgchem.6c02061 (PMC13509001; doi:10.1021/acs.inorgchem.6c02061)
Supplement: Supplementary file 1 [file ic6c02061_si_001.pdf]

Supporting Information

Enhancing Lithium Binding Affinity, Exchange Rate and Hard–Soft Selectivity of Catenane Ligands by Post-Interlocking Modification

Yueliang Yao,[1] Mingda Hu,[1] Hang Zhou,[1] Samuel Kin-Man Lai[1] and Ho Yu Au-Yeung[1,2*]

[1]Department of Chemistry, The University of Hong Kong, Pokfulam Road, Hong Kong, China

[2]CAS-HKU Joint Laboratory on New Materials and State Key Laboratory of Synthetic Chemistry, The University of Hong Kong, Pokfulam Road, Hong Kong, China

*Correspondence: hoyuay@hku.hk

1. NMR Spectra

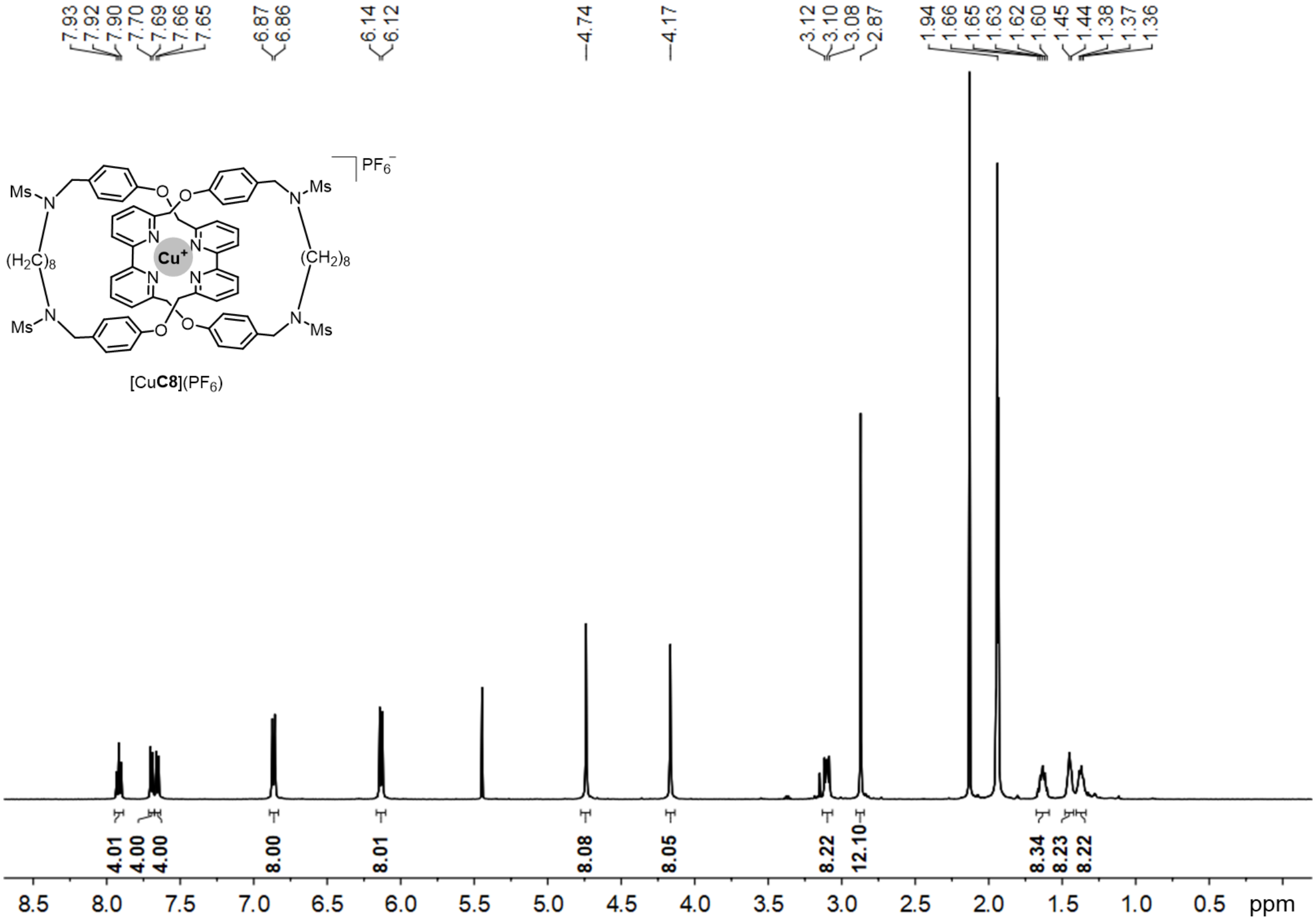

Figure S1. ^{1}H NMR (500 MHz, CD_3CN, 298 K) of [Cu(**C8**)](PF_6).

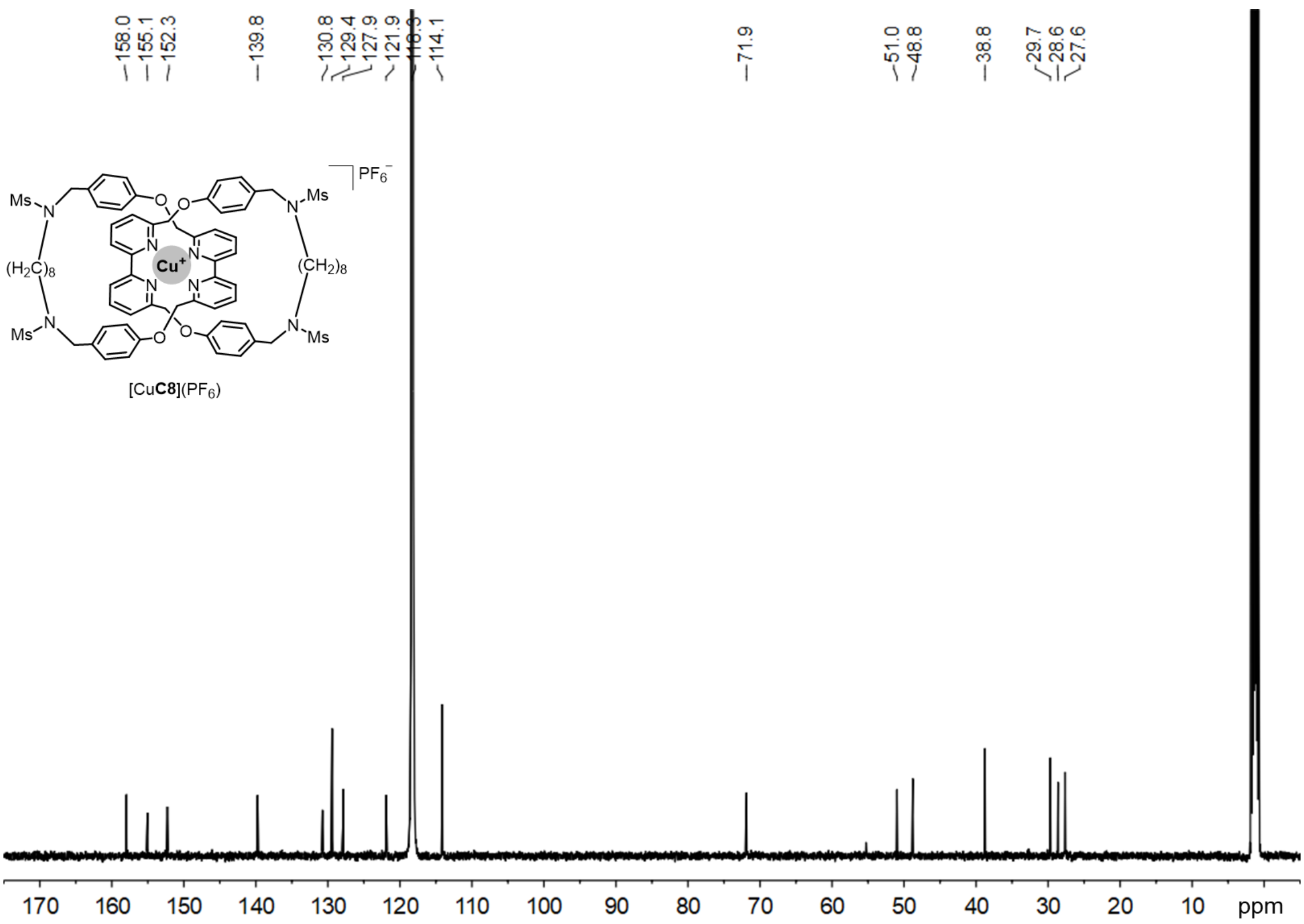

Figure S2. ^{13}C{^{1}H} NMR (125 MHz, CD_3CN, 298 K) of [Cu(**C8**)](PF_6).

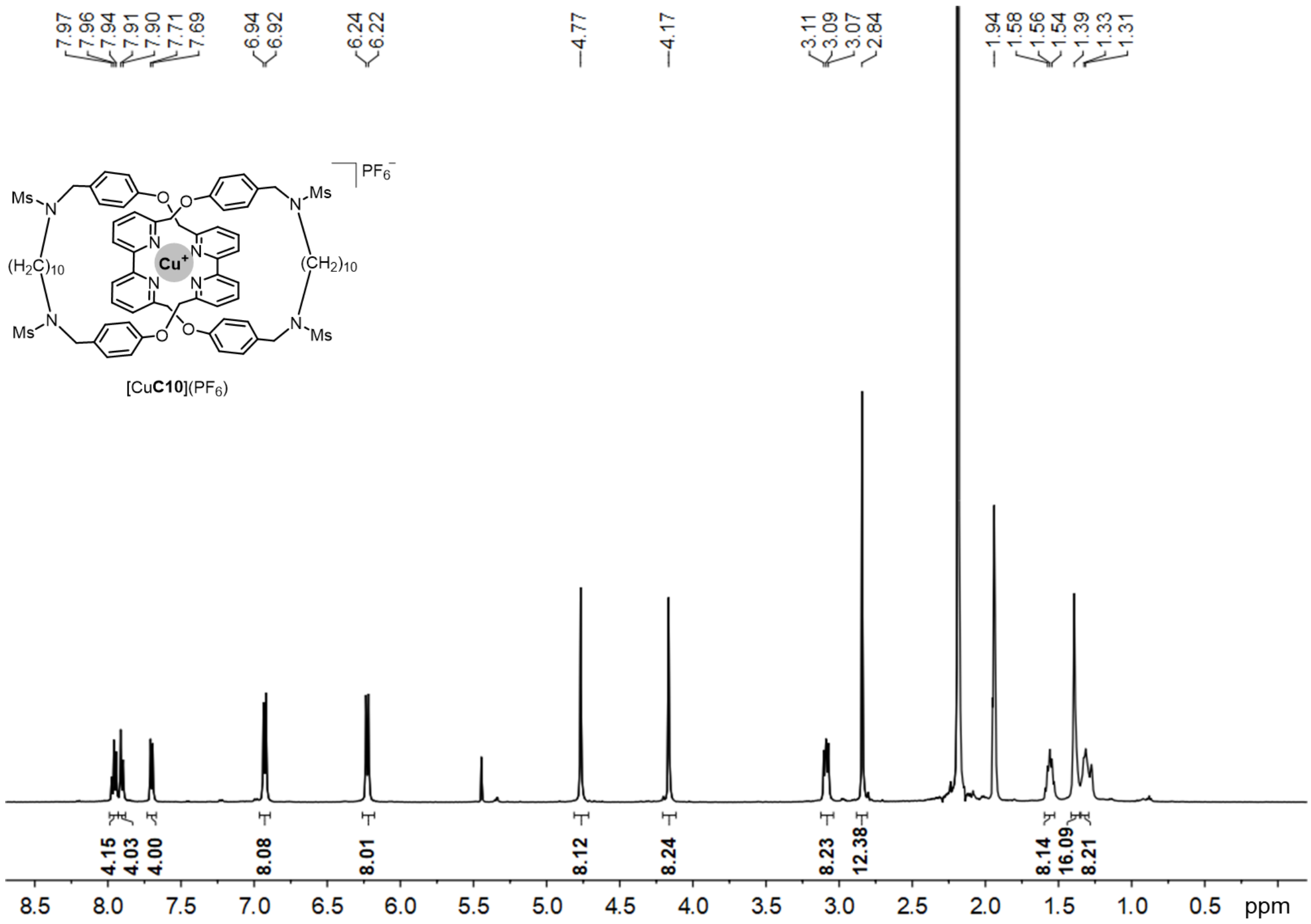

Figure S3. ^{1}H NMR (500 MHz, CD_3CN, 298 K) of [Cu(**C10**)](PF_6).

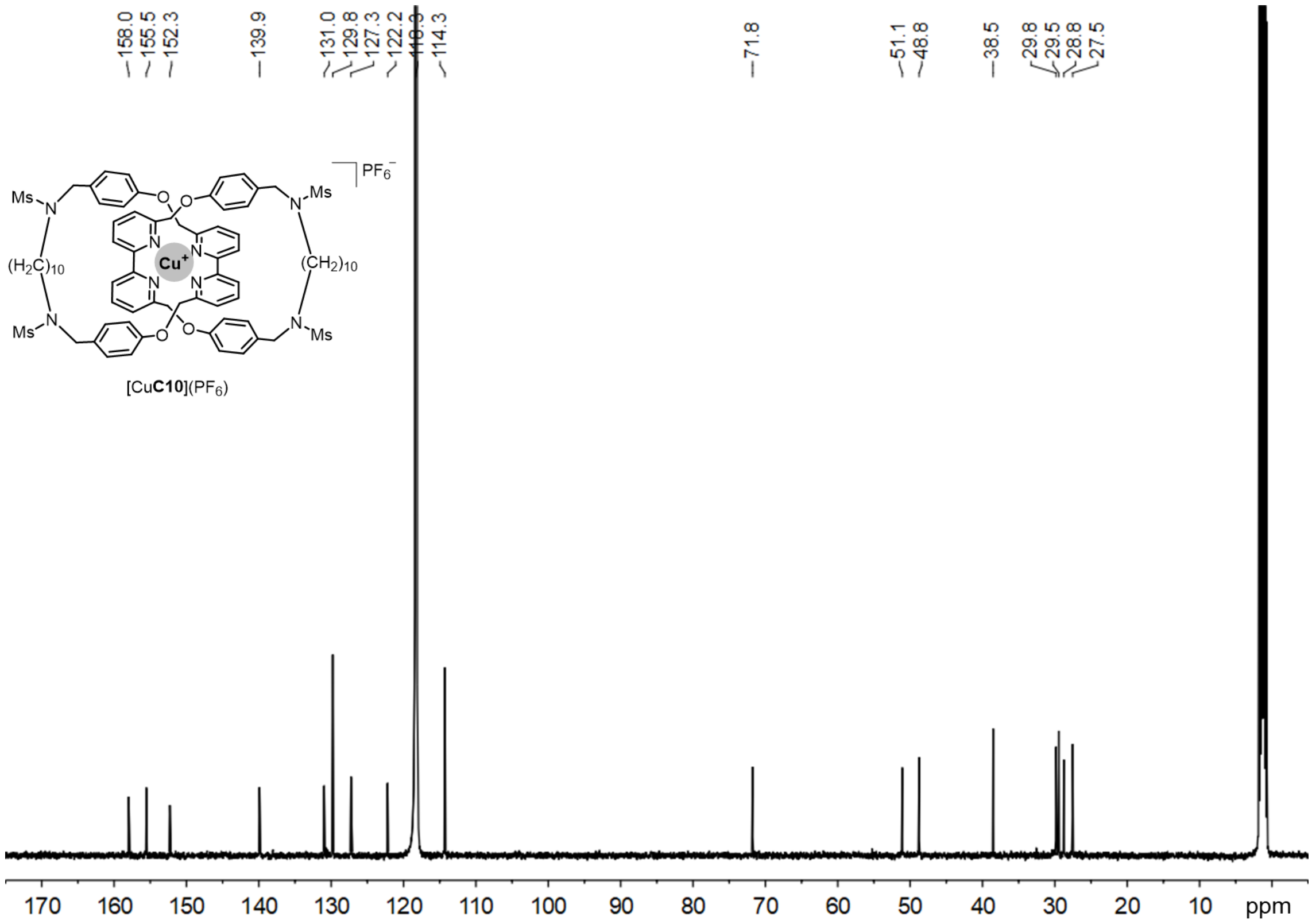

Figure S4. $^{13}C\{^{1}H\}$ NMR (125 MHz, CD_3CN, 298 K) of [Cu(**C10**)](PF_6).

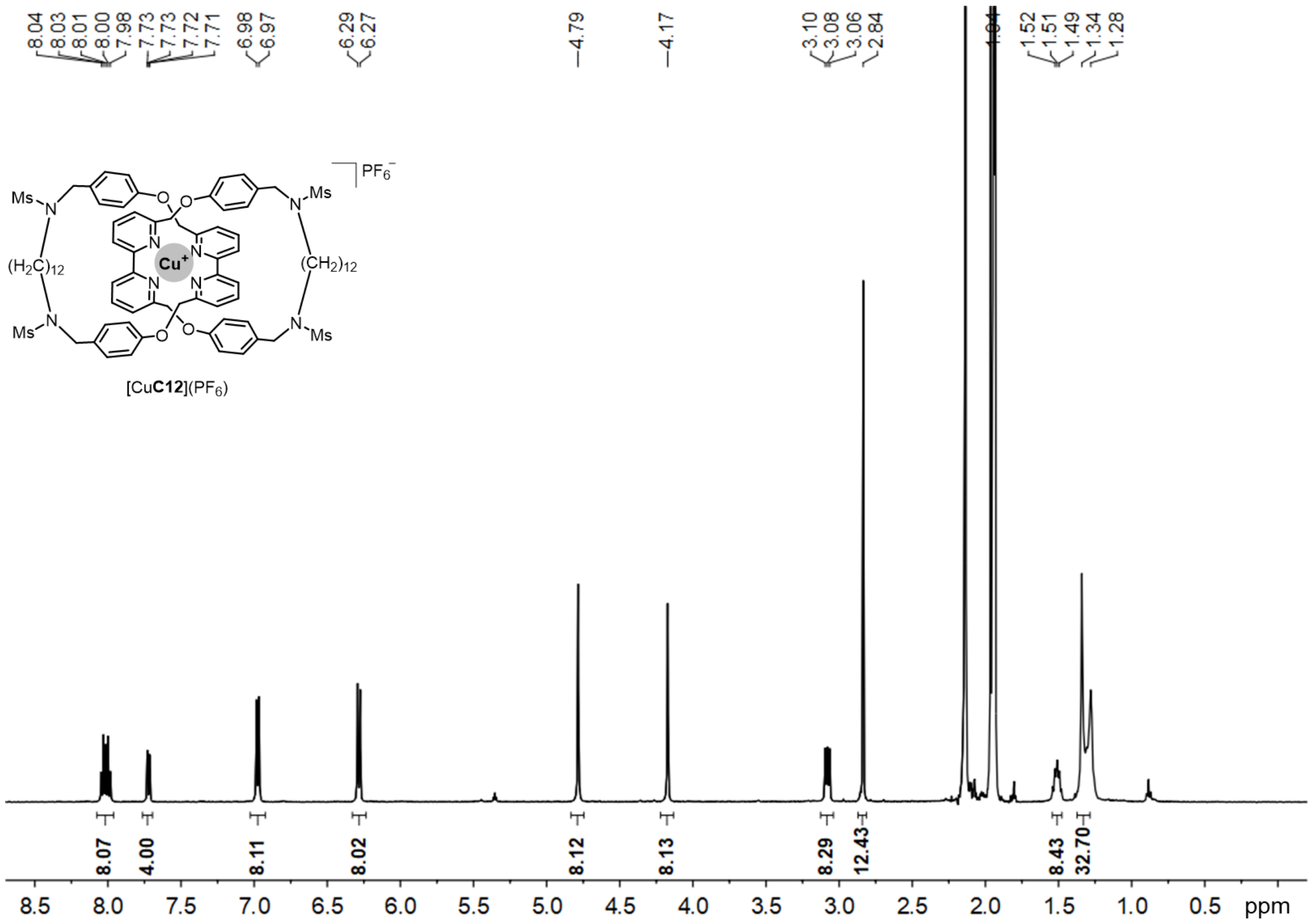

Figure S5. ^{1}H NMR (500 MHz, CD_3CN, 298 K) of [Cu(**C12**)](PF_6).

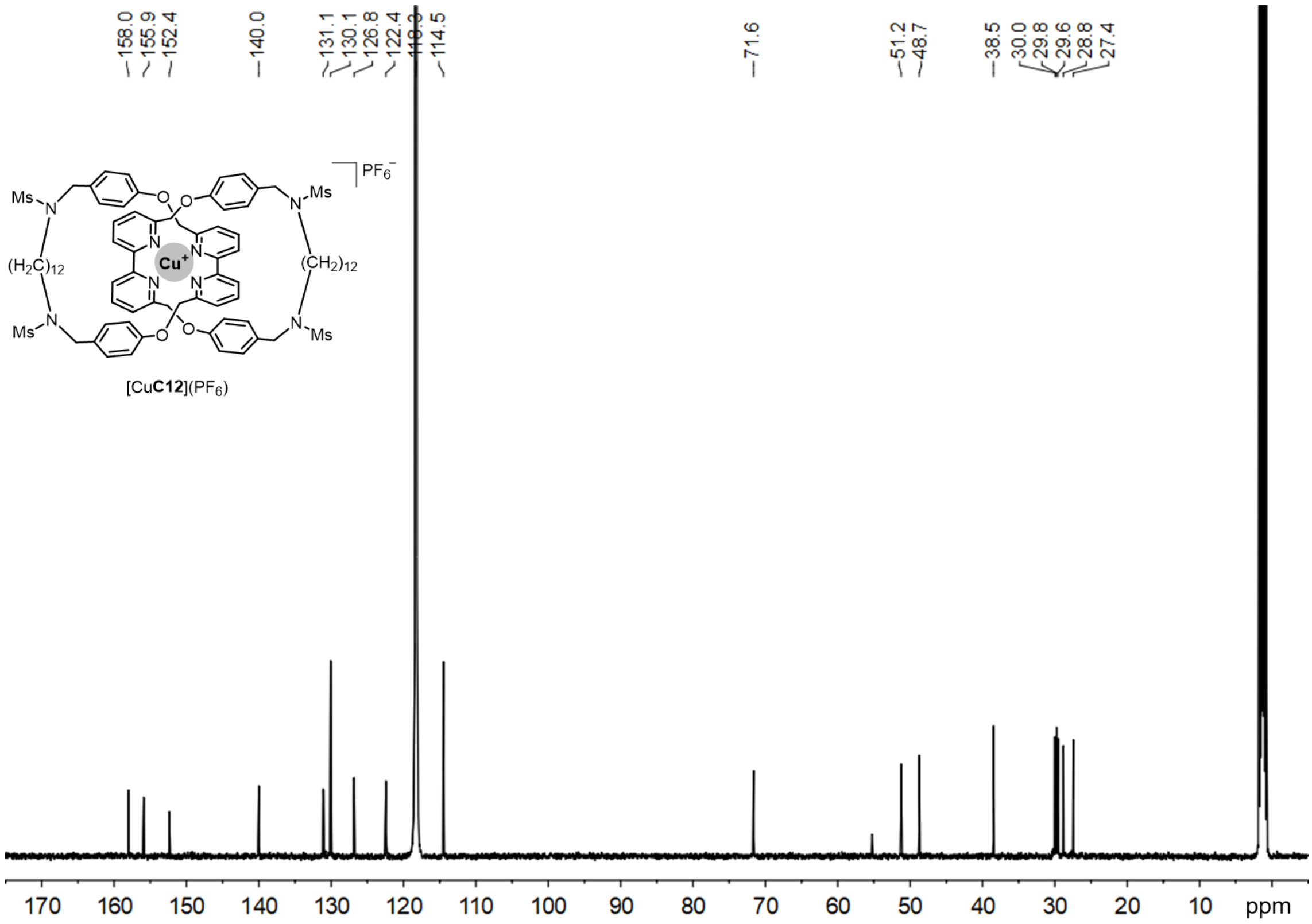

Figure S6. $^{13}C\{^{1}H\}$ NMR (125 MHz, CD_3CN, 298 K) of [Cu(**C12**)](PF_6).

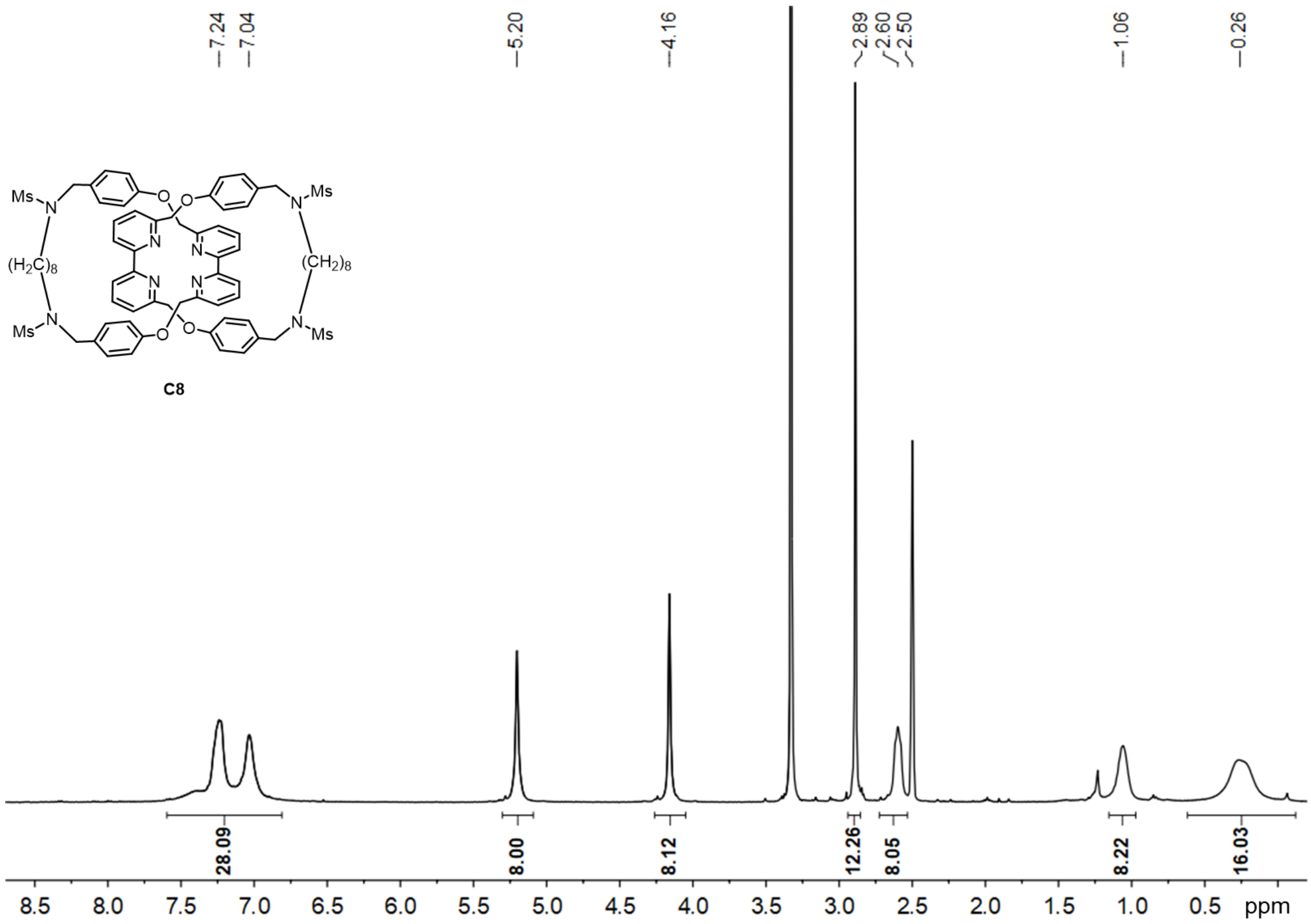

Figure S7. ^{1}H NMR (400 MHz, DMSO-d_6, 298 K) of **C8**.

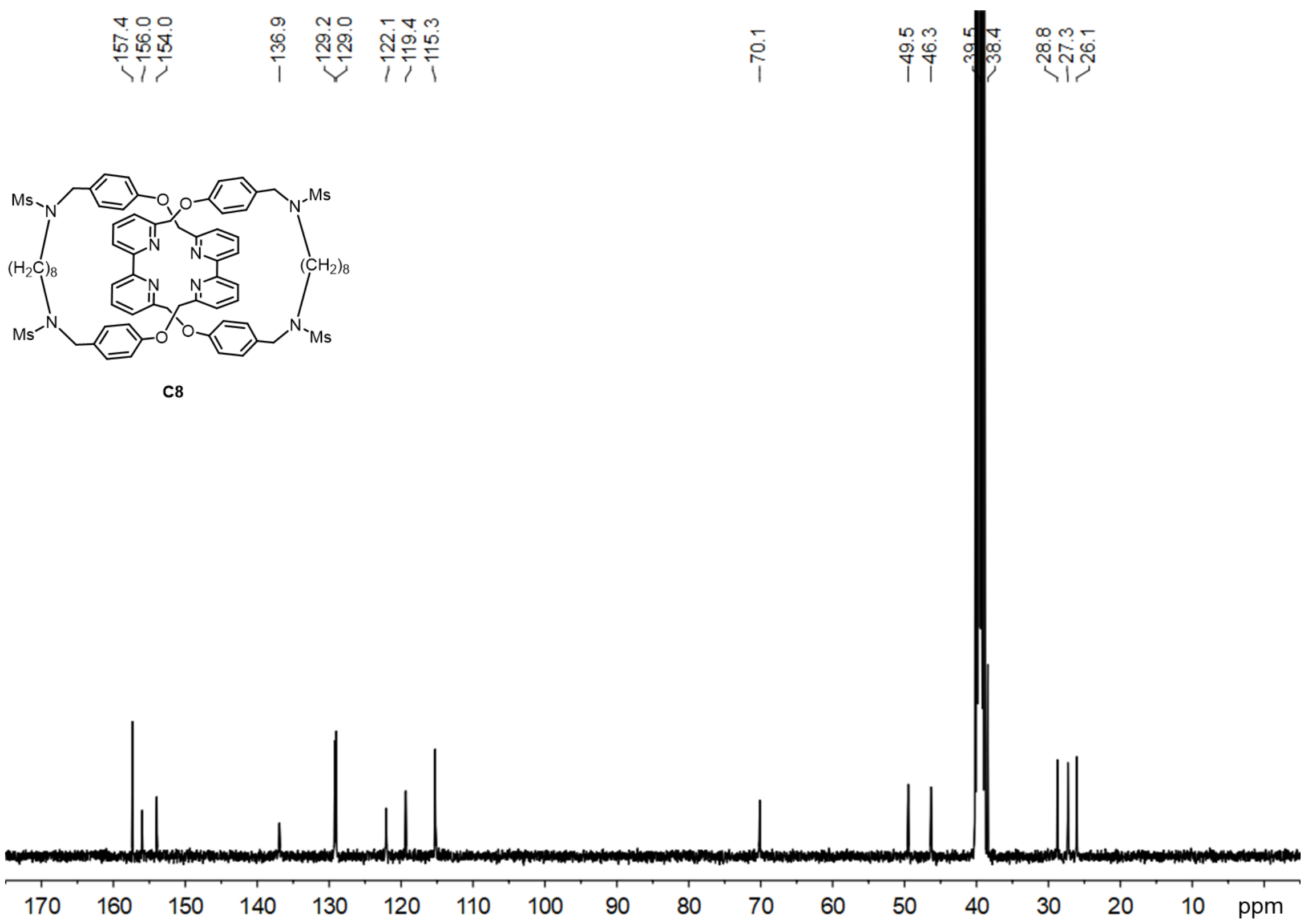

Figure S8. ^{13}C{^{1}H} NMR (100 MHz, DMSO-d_6, 298 K) of **C8**.

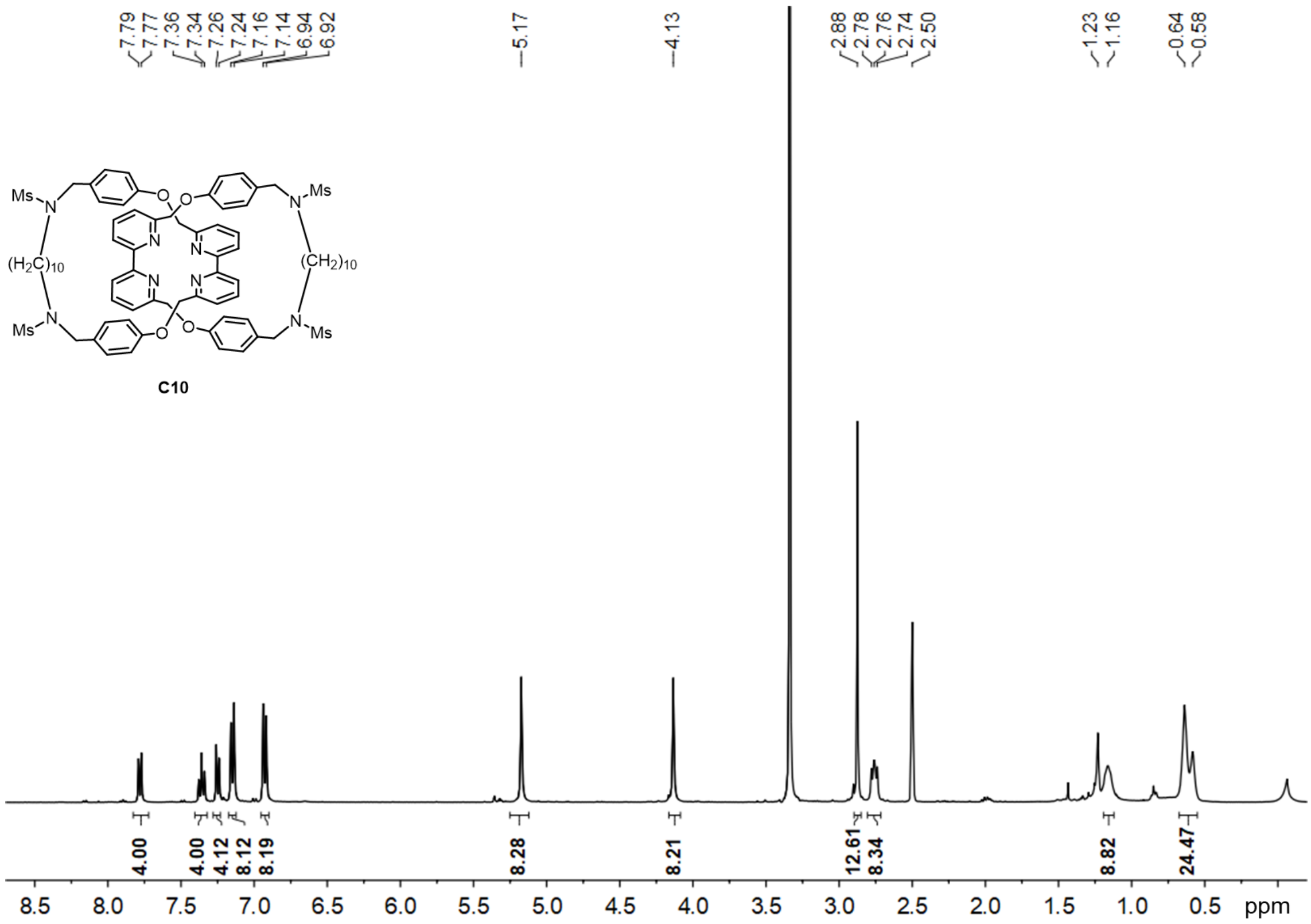

Figure S9. ^{1}H NMR (400 MHz, DMSO-d_6, 298 K) of **C10**.

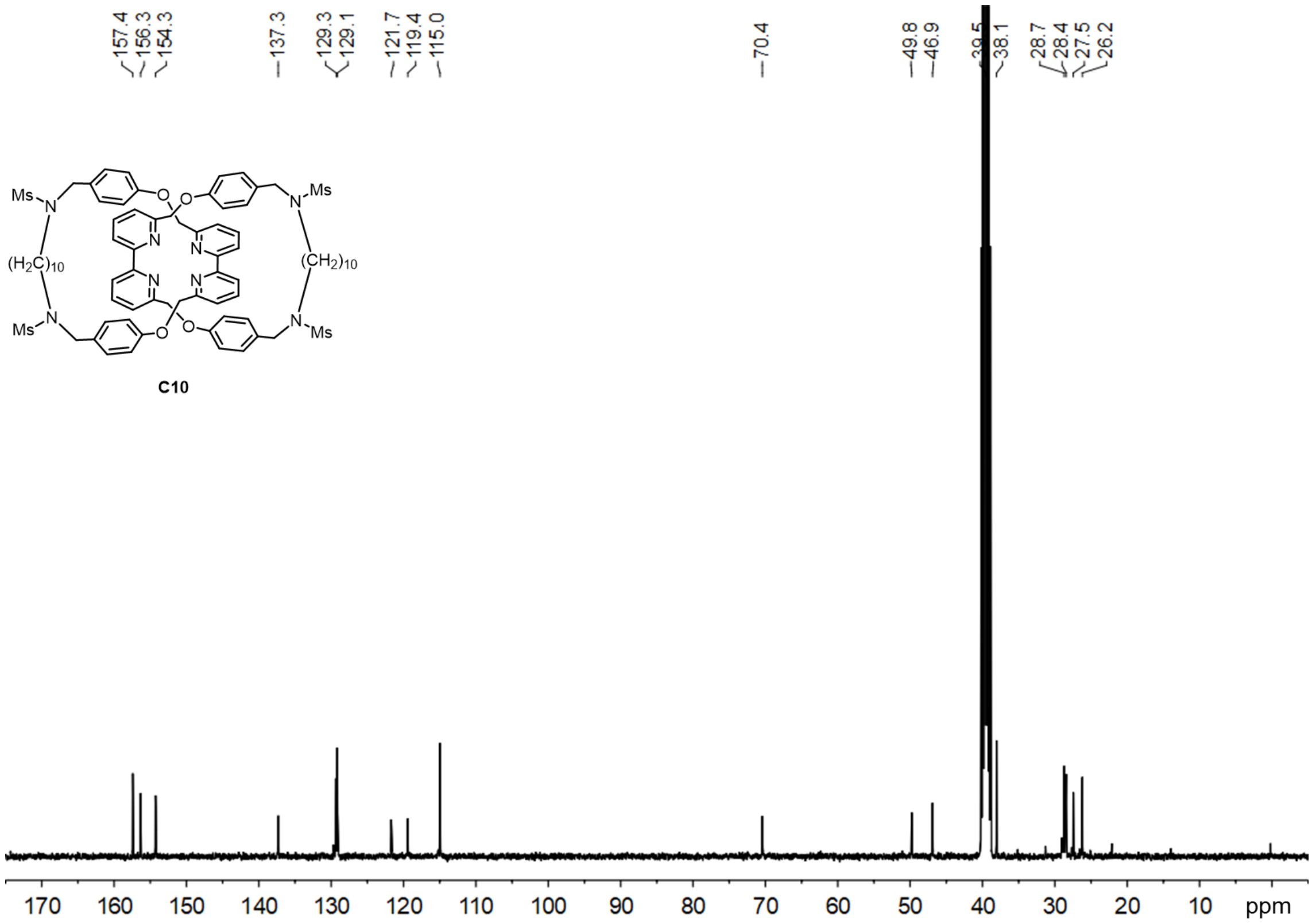

Figure S10. $^{13}C\{^{1}H\}$ NMR (100 MHz, DMSO-d_6, 298 K) of **C10**.

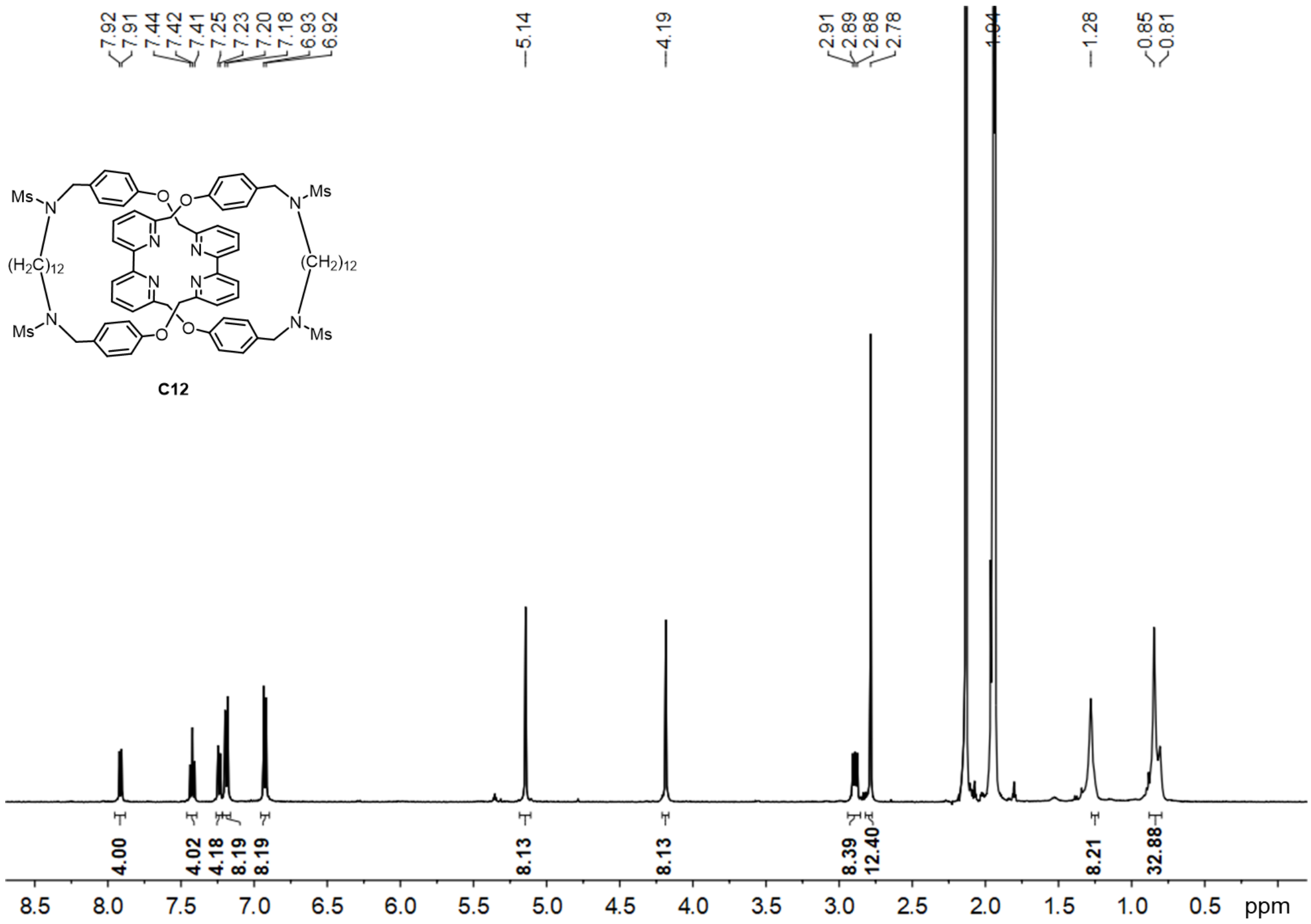

Figure S11. ^{1}H NMR (500 MHz, CD_3CN, 298 K) of **C12**.

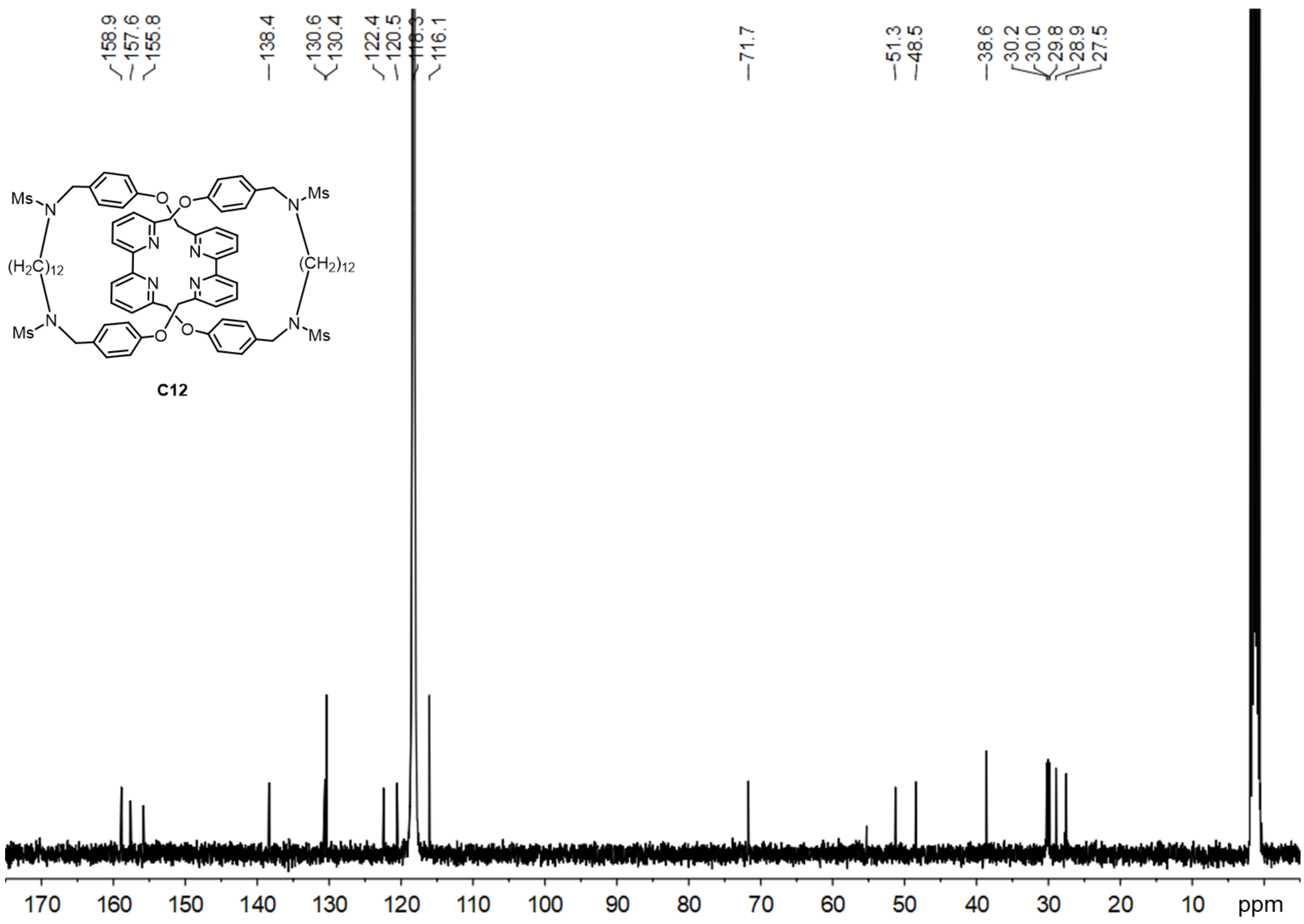

Figure S12. $^{13}C\{^{1}H\}$ NMR (100 MHz, CD_3CN, 298 K) of **C12**.

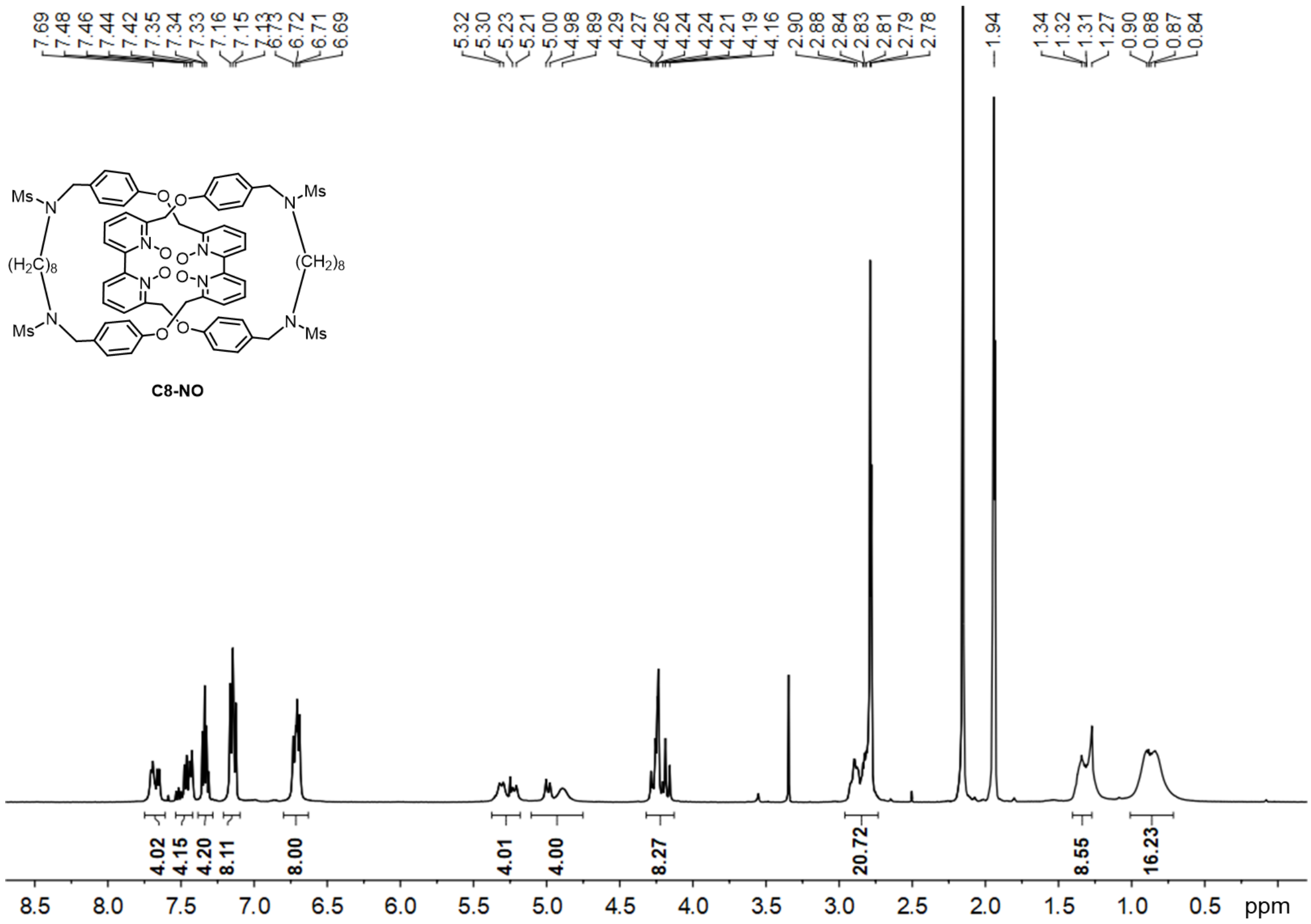

Figure S13. ^{1}H NMR (500 MHz, CD_3CN, 298 K) of **C8-NO**.

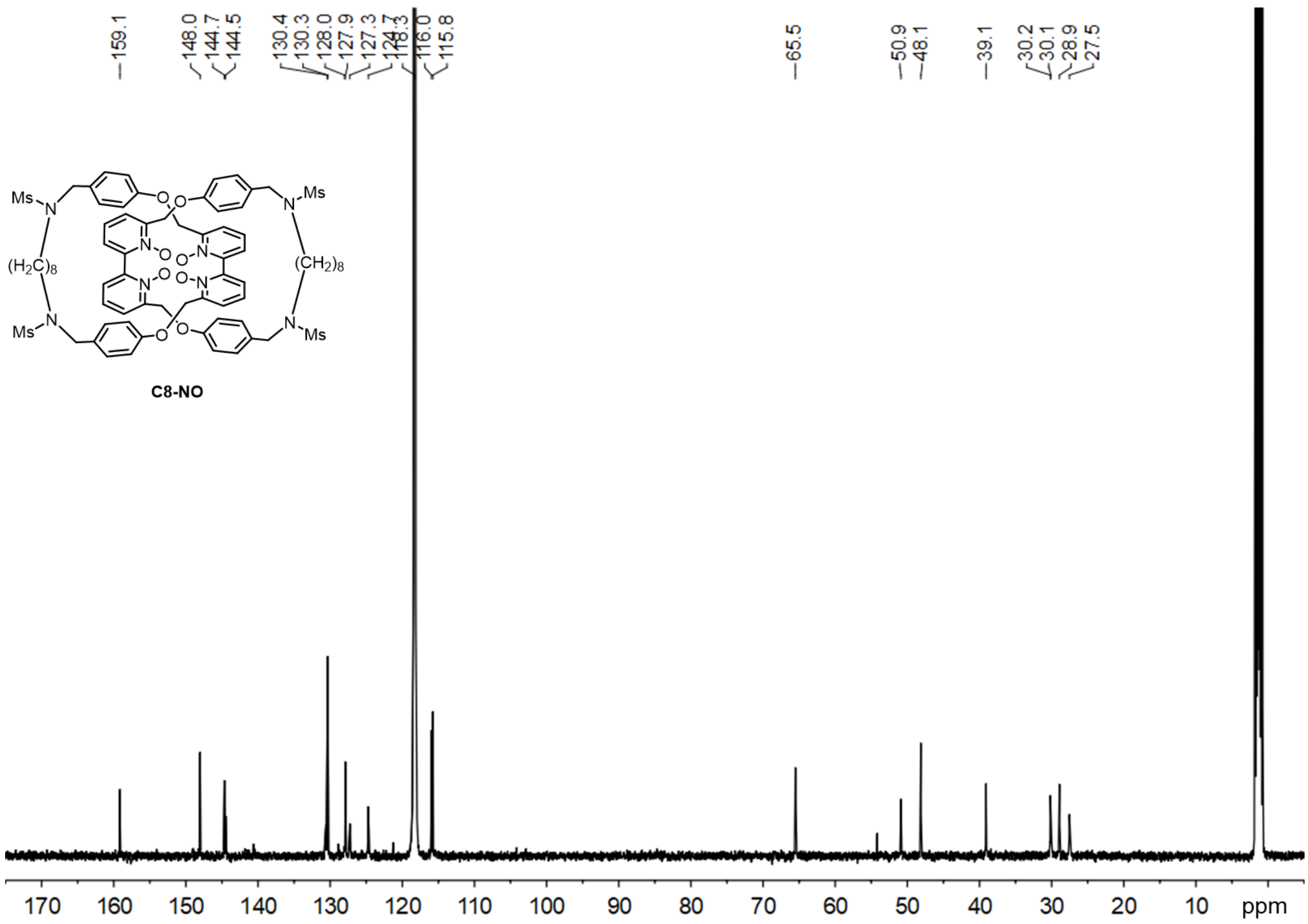

Figure S14. $^{13}C\{^{1}H\}$ NMR (125 MHz, CD_3CN, 298 K) of **C8-NO**.

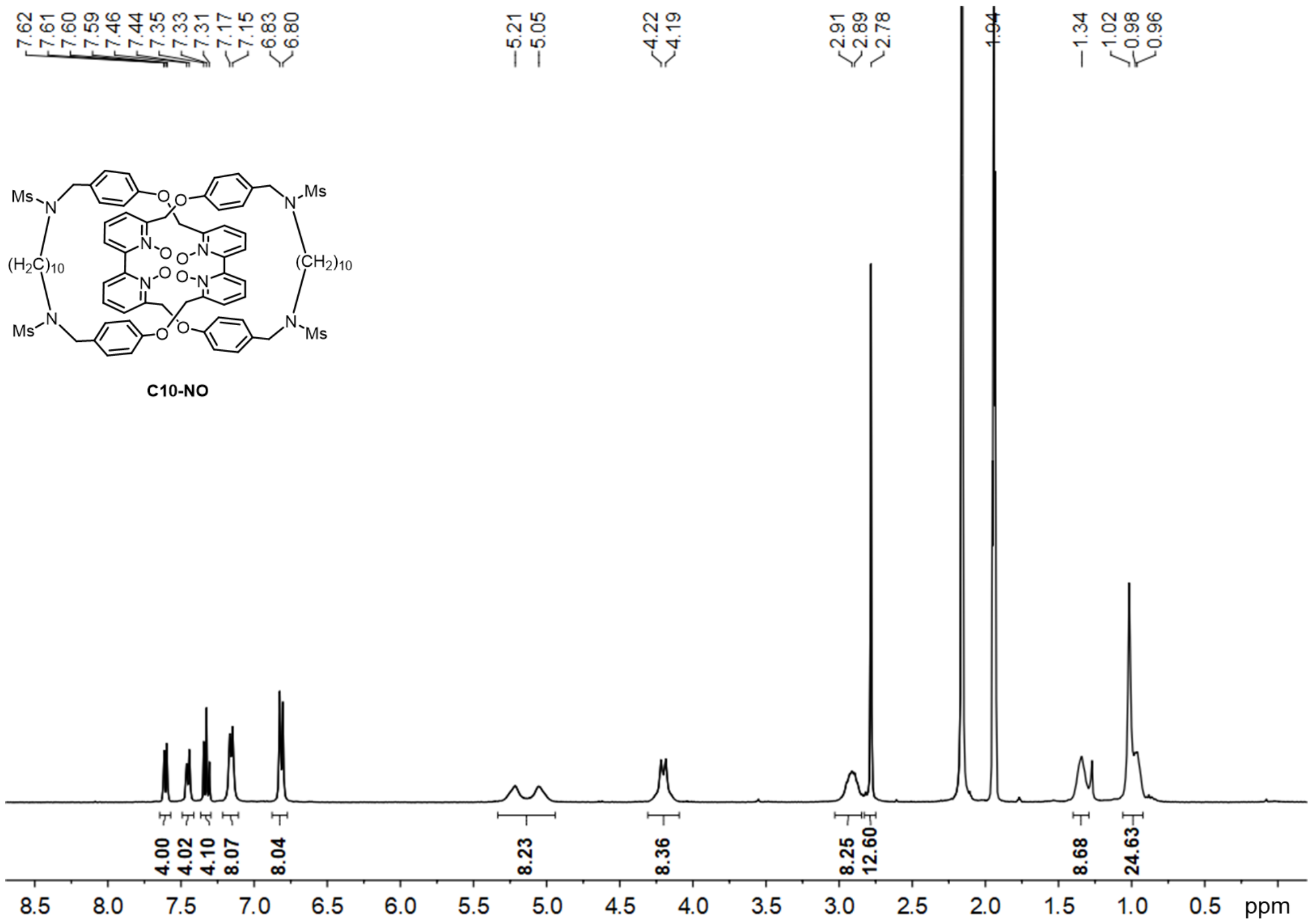

Figure S15. ^{1}H NMR (400 MHz, CD_3CN, 298 K) of **C10-NO**.

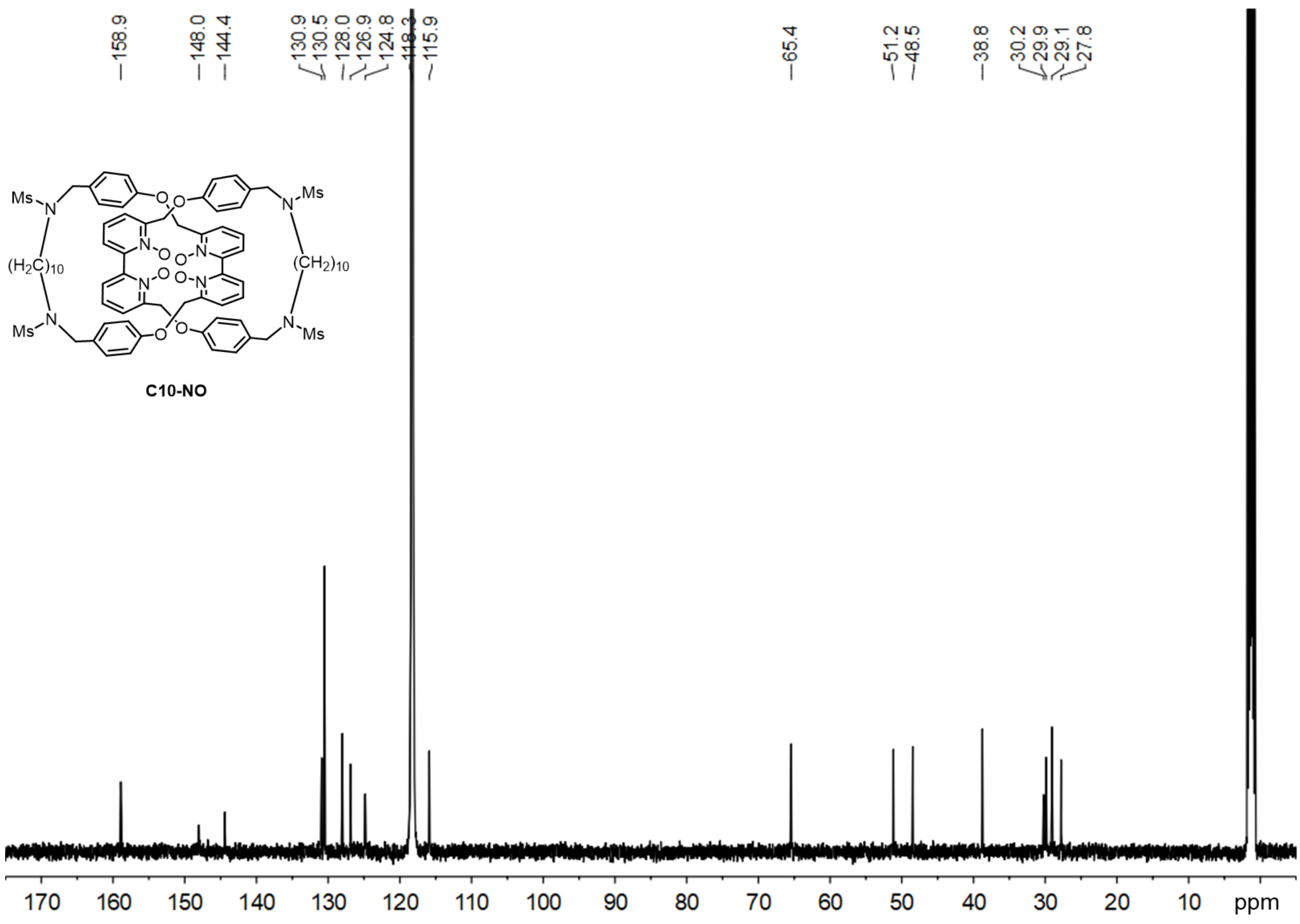

Figure S16. $^{13}C\{^{1}H\}$ NMR (125 MHz, CD_3CN, 298 K) of **C10-NO**.

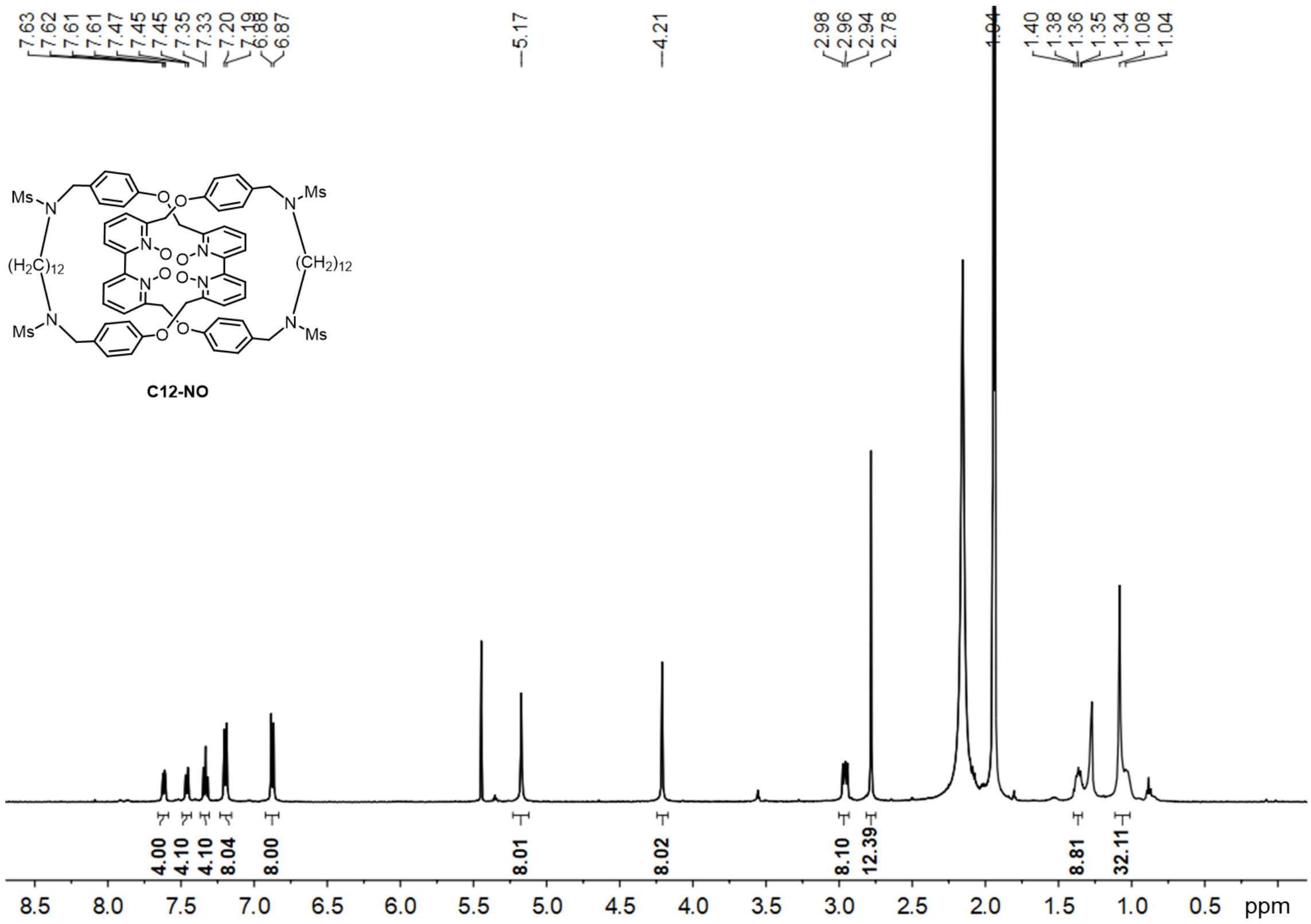

Figure S17. ^{1}H NMR (500 MHz, CD_3CN, 298 K) of **C12-NO**.

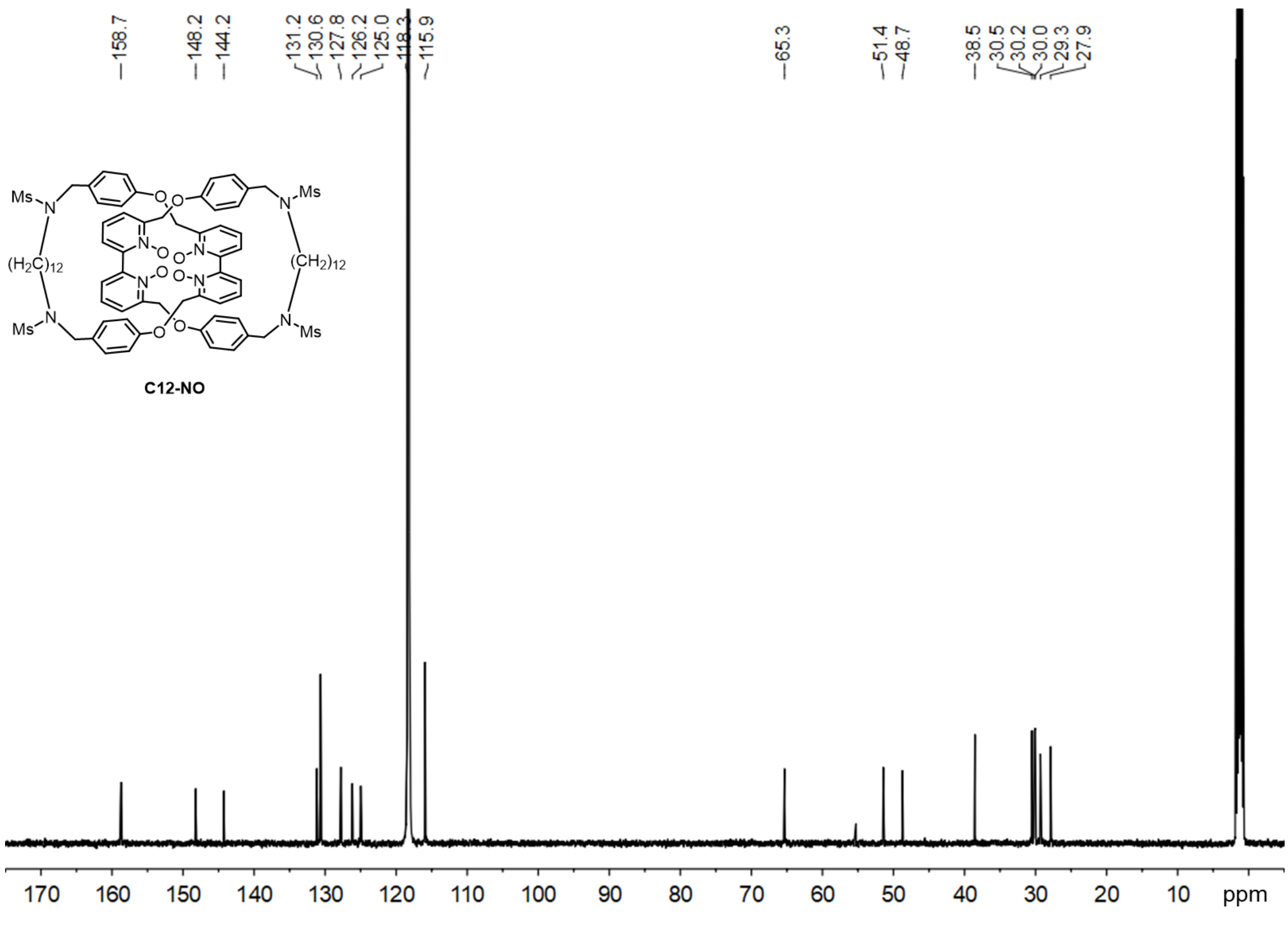

Figure S18. $^{13}C\{^{1}H\}$ NMR (125 MHz, CD_3CN, 298 K) of **C12-NO**.

2. Cation Binding

Metal salts (i.e. LiOTf, NaOTf, KOTf, $[Cu(MeCN)_4](PF_6)$, $AgBF_4$ and $Zn(ClO_4)_2$) were purchased from commercial suppliers (J&K, Sigma-Aldrich, Aladdin and TCI, purity ≥ 98%) and stored in a vacuum desiccator. Deuterated solvents $CDCl_3$ and CD_3CN for stock solutions and NMR samples were purchased from J&K (≥99.8 atom% D) and used without further purification.

In a typical ^1H NMR titration experiment, aliquots of 50 mM solutions of a guest in $CD_3CN/CDCl_3$ (1:1, *v*/*v*) or CD_3CN were sequentially added to a 1 mM solution of the host (500 µL) at 298 K in the same solvent and mixed well before a ^1H NMR spectrum is collected. ^1H NMR spectra were obtained using a Bruker DPX spectrometer with a working frequency at 500 MHz. HR-ESI-MS spectra were obtained from a Bruker Impact II Ultra-High Resolution QTOF mass spectrometer.

For the binding to **C12**, the binding stoichiometry of Li^+, Cu^+, Zn^{2+} and Ag^+ was studied by titrating 0–2 eq. of LiOTf, $[Cu(MeCN)_4](PF_6)$, $Zn(ClO_4)_2$ and $AgBF_4$ into a solution of **C12** (500 µL, 1 mM) in $CD_3CN/CDCl_3$ (1:1, *v*/*v*) or CD_3CN. A quantitative conversion to the corresponding complexes was observed upon addition of up to 1 molar equivalent of the metal ions, showing a 1:1 binding stoichiometry of these metals. Binding stoichiometry of Na^+ to **C12**, and that of Li^+, Na^+ and K^+ to **C10-NO** and **C12-NO** in $CD_3CN/CDCl_3$ (1:1, *v*/*v*) were determined by the continuous variation method (Job plot) with a 1 mM total concentration of the host and guest by monitoring the changes in the chemical shift of the host protons (H_{Ar}, OCH_2 or H_{alkyl}). For the Ag^+ binding to **C12-NO** and **C10-NO** in CD_3CN, due to the small chemical shift changes, covariances of the fit using different fitting models were compared for the best fitting model.[1]

Association constants were determined by ^1H NMR titration.[2] For fast binding relative to the NMR timescale, changes in the chemical shift of the host protons were monitored, and association constants were obtained by a global fitting (1:1 binding model) of the binding isotherms using the online program Bindfit.[3] For slow binding relative to the NMR timescale, integrations of the proton signals of the free host and host–guest complex were monitored to obtain their equilibrium concentrations. Association constants were obtained from the slope of a graph plotting the equilibrium concentration of the complex against the product of the equilibrium concentrations of the free host and guest. For the

strong binding of Ag^+, Cu^+ and Li^+, competitive titrations were adopted,[4,5] and details are described below.

$$[\mathrm{Ag}(\mathbf{C12})]^+ + \mathrm{I}^- \overset{K_{\mathrm{app}}}{\rightleftharpoons} \mathbf{C12} + \mathrm{AgI\ (s)}$$

$$\mathrm{AgI\ (s)} \overset{K_{\mathrm{sp}}}{\rightleftharpoons} \mathrm{Ag}^+ + \mathrm{I}^-$$

$$\mathbf{C12} + \mathrm{Ag}^+ \overset{K(\mathrm{Ag}^+)}{\rightleftharpoons} [\mathrm{Ag}(\mathbf{C12})]^+$$

$$K(\mathrm{Ag}^+) = (1/K_{\mathrm{sp}})/K_{\mathrm{app}}$$

Scheme S1. Equilibria of the competition between Ag(I) catenane and AgI for the determination of Ag^+ binding constant to **C12**.

For Ag^+ binding to **C12**, a 1 mM $[Ag(\mathbf{C12})]^+$ solution was first prepared by addition of 1 eq. of $AgBF_4$ to a 1 mM solution of **C12** in CD_3CN (500 μL). Aliquots (1–2 μL) of a 50 mM solution of tetrabutylammonium iodide (TBAI) in CD_3CN were sequentially added, and the solution was mixed well before a ^1H NMR spectrum was collected. Equilibrium concentrations of $[Ag(\mathbf{C12})]^+$ and free **C12** were determined from the peak integrations of the corresponding proton signals. The apparent equilibrium constant K_{app} was obtained by linear fitting (Scheme S1). Association constant of Ag^+ to **C12** was calculated by referencing to the solubility product of AgI in MeCN ($K_{sp} = 6.3 \times 10^{-15}$ M^2).[6]

$$[\mathrm{Ag}(\mathbf{C12})]^+ + \mathrm{Cu}^+ \overset{K_{\mathrm{app}}}{\rightleftharpoons} [\mathrm{Cu}(\mathbf{C12})]^+ + \mathrm{Ag}^+$$

$$\mathbf{C12} + \mathrm{Ag}^+ \overset{K(\mathrm{Ag}^+)}{\rightleftharpoons} [\mathrm{Ag}(\mathbf{C12})]^+$$

$$\mathbf{C12} + \mathrm{Cu}^+ \overset{K(\mathrm{Cu}^+)}{\rightleftharpoons} [\mathrm{Cu}(\mathbf{C12})]^+$$

$$K(\mathrm{Cu}^+) = K(\mathrm{Ag}^+) \times K_{\mathrm{app}}$$

Scheme S2. Equilibria of the competition between Ag(I) catenane and Cu(I) catenane for the determination of Cu^+ binding constant to **C12**.

For Cu^+ binding to **C12**, a mixture containing 20 eq. of $AgBF_4$ in a 1 mM solution of **C12** (500 μL) in CD_3CN was first prepared. Aliquots (2–30 μL) of a 50 mM solution of $[Cu(MeCN)_4](PF_6)$ in the same solvent were sequentially added, and the solution was mixed well before a ^1H NMR spectrum was collected. Equilibrium concentrations of

$[Ag(\mathbf{C12})]^+$ and $[Cu(\mathbf{C12})]^+$ were determined from the peak integrations of the corresponding proton signals. The apparent equilibrium constant K_{app} was obtained by linear fitting (Scheme S2). Association constant of Cu^+ to **C12** was calculated by referencing to the Ag^+ binding constant obtained as described above.

$$Li^+ + [Na(\mathbf{cat})]^+ \xrightleftharpoons{K_{app}} Na^+ + [Li(\mathbf{cat})]^+$$

$$\mathbf{cat} + Na^+ \xrightleftharpoons{K(Na^+)} [Na(\mathbf{cat})]^+$$

$$\mathbf{cat} + Li^+ \xrightleftharpoons{K(Li^+)} [Li(\mathbf{cat})]^+$$

$$K(Li^+) = K(Na^+) \times K_{app}$$

cat = **C12**, **C12-NO** or **C10-NO**

Scheme S3. Equilibria of the competition between Na^+ catenane and Li^+ catenane for the determination of Li^+ binding constant to **C12**, **C12-NO** and **C10-NO**.

For Li^+ binding to **C12**, **C12-NO** and **C10-NO**, a mixture containing 100 eq. of NaOTf in a 1 mM solution of the catenane host in 1:1 (*v*/*v*) $CD_3CN/CDCl_3$ (500 µL) was first prepared. Aliquots (2–30 µL) of a 50 mM solution of LiOTf in the same solvent were sequentially added, and the solution was mixed well before a ^1H NMR spectrum was collected. The apparent equilibrium constants K_{app} were obtained by a global fitting of the binding isotherms to a 1:1 model using the online program Bindfit.[3] Association constants of Li^+ to the catenane hosts were calculated by referencing to the corresponding Na^+ binding constants obtained as described above.

Table S1. Summary of 1:1 cation association constant (M^{-1}) of catenane hosts at 298 K.

Host	Guest	Solvent	K (M^{-1})	method
**C12**	LiOTf	$CD_3CN/CDCl_3$ (1:1, *v*/*v*)	$1.1 (\pm 0.09) \times 10^5$	competitive titration with NaOTf
**C12-NO**	LiOTf	$CD_3CN/CDCl_3$ (1:1, *v*/*v*)	$4.5 (\pm 0.2) \times 10^5$	competitive titration with NaOTf
**C10-NO**	LiOTf	$CD_3CN/CDCl_3$ (1:1, *v*/*v*)	$1.1 (\pm 0.03) \times 10^6$	competitive titration with NaOTf
**C12**	NaOTf	$CD_3CN/CDCl_3$ (1:1, *v*/*v*)	460 (±8)	direct titration
**C12-NO**	NaOTf	$CD_3CN/CDCl_3$ (1:1, *v*/*v*)	$5.1 (\pm 0.1) \times 10^3$	direct titration
**C10-NO**	NaOTf	$CD_3CN/CDCl_3$ (1:1, *v*/*v*)	$1.4 (\pm 0.3) \times 10^4$	direct titration
**C12**	KOTf	$CD_3CN/CDCl_3$ (1:1, *v*/*v*)	no binding	direct titration
**C12-NO**	KOTf	$CD_3CN/CDCl_3$ (1:1, *v*/*v*)	210 (±4)	direct titration
**C10-NO**	KOTf	$CD_3CN/CDCl_3$ (1:1, *v*/*v*)	260 (±10)	direct titration
**C12**	$[Cu(MeCN)_4](PF_6)$	CD_3CN	$7.1 (\pm 0.1) \times 10^{11}$	competitive titration with $AgBF_4$
**C12-NO**	$[Cu(MeCN)_4](PF_6)$	CD_3CN	no binding	direct titration
**C10-NO**	$[Cu(MeCN)_4](PF_6)$	CD_3CN	no binding	direct titration
**C12**	$AgBF_4$	CD_3CN	$1.4 (\pm 0.05) \times 10^{10}$	competitive titration with TBAI
**C12-NO**	$AgBF_4$	CD_3CN	110 (±4)	direct titration
**C10-NO**	$AgBF_4$	CD_3CN	90 (±1)	direct titration

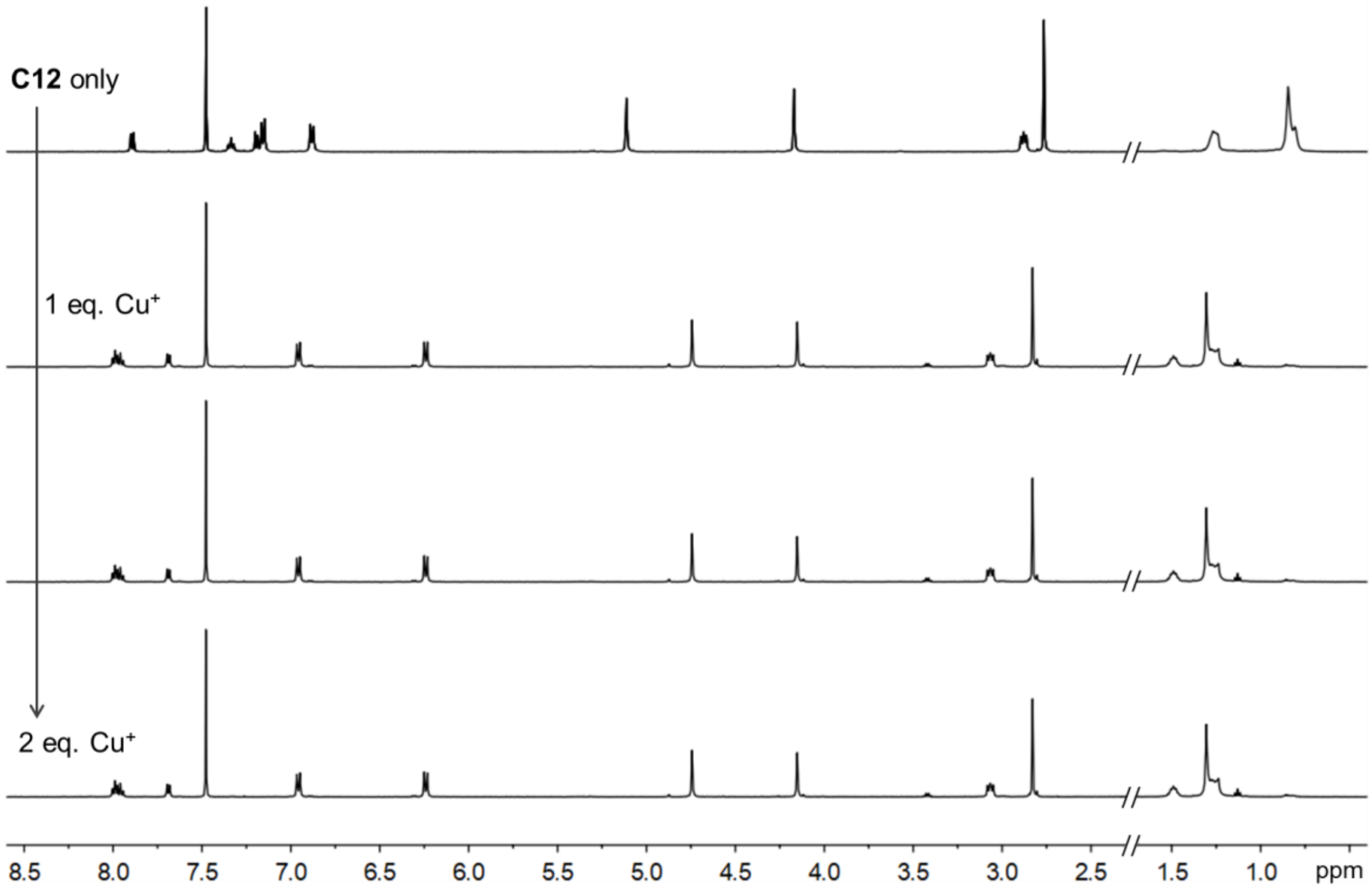

Figure S19. Partial ^{1}H NMR spectra (500 MHz, 298 K, $CD_3CN/CDCl_3$ (1:1, *v/v*)) of a 1 mM solution of **C12** in the presence of 0 eq. (top), 1 eq., 1.5 eq. and 2 eq. (bottom) of $[Cu(MeCN)_4](PF_6)$.

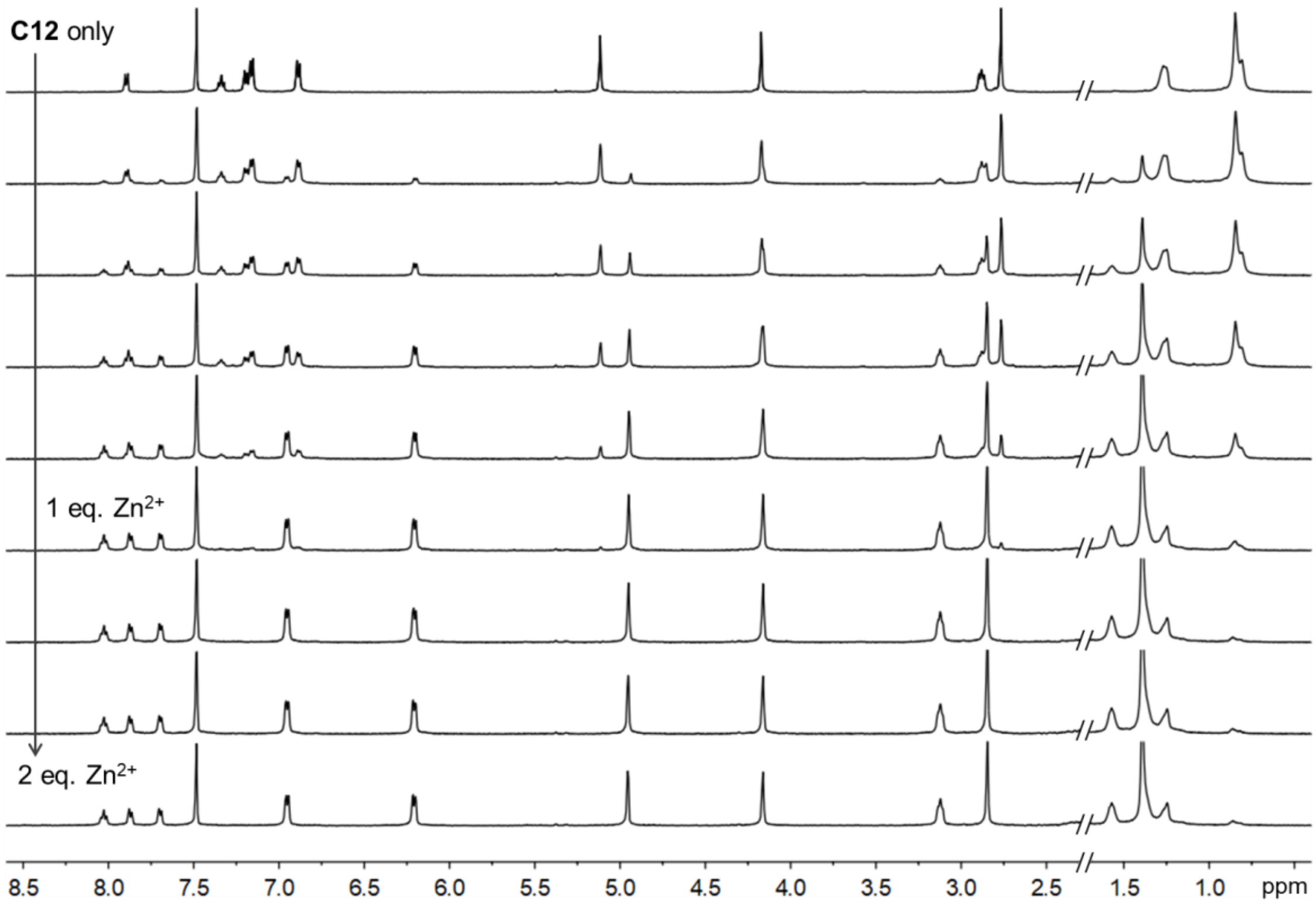

Figure S20. Partial ^{1}H NMR spectra (500 MHz, 298 K, $CD_3CN/CDCl_3$ (1:1, *v/v*)) of a 1 mM solution of **C12** in the presence of 0 eq. (top), 0.2 eq., 0.4 eq., 0.6 eq., 0.8 eq., 1 eq., 1.2 eq., 1.5 eq. and 2 eq. (bottom) of $Zn(ClO_4)_2$.

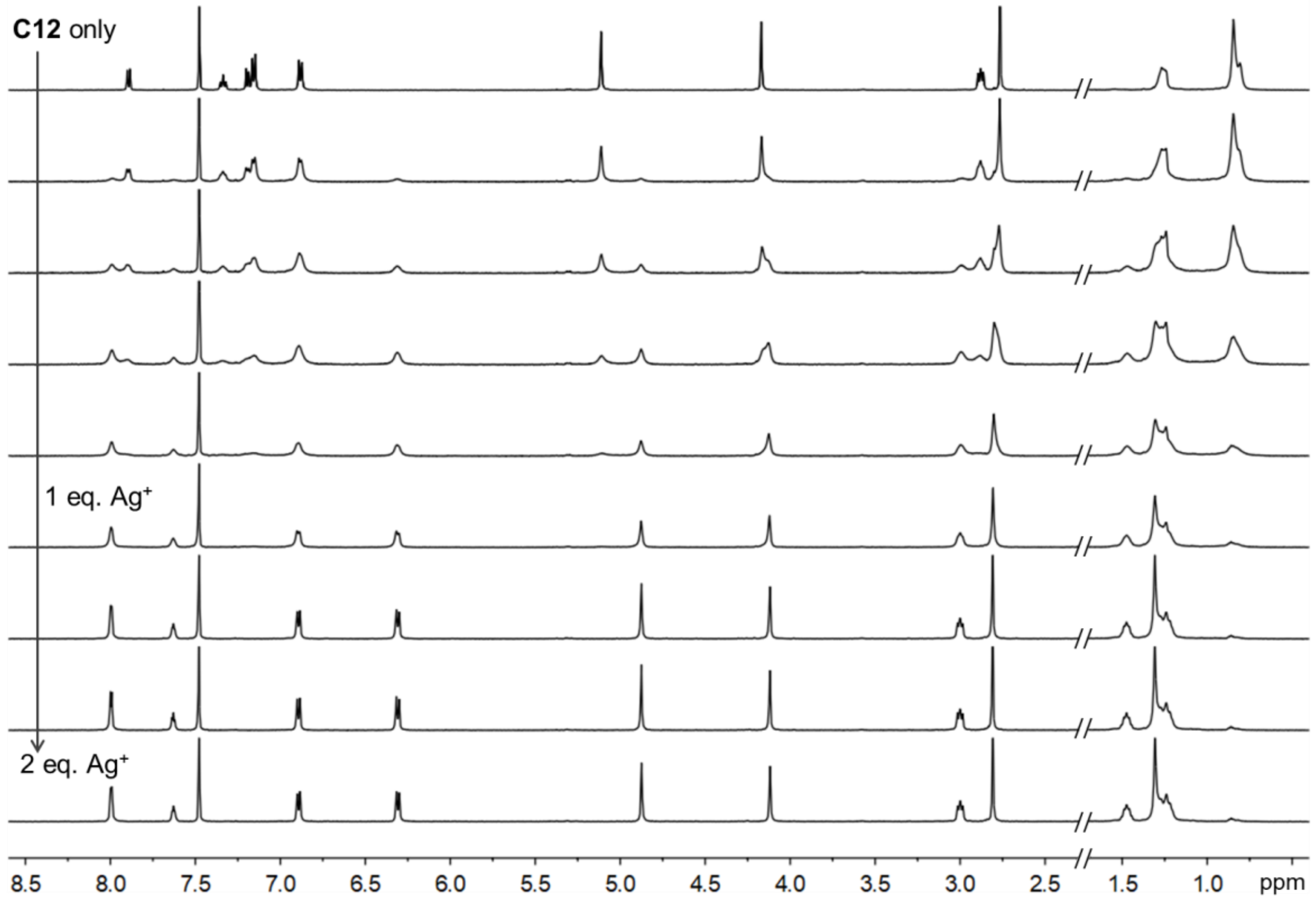

Figure S21. Partial ^{1}H NMR spectra (500 MHz, 298 K, $CD_3CN/CDCl_3$ (1:1, *v*/*v*)) of a 1 mM solution of **C12** in the presence of 0 eq. (top), 0.2 eq., 0.4 eq., 0.6 eq., 0.8 eq., 1 eq., 1.2 eq., 1.5 eq. and 2 eq. (bottom) of $AgBF_4$.

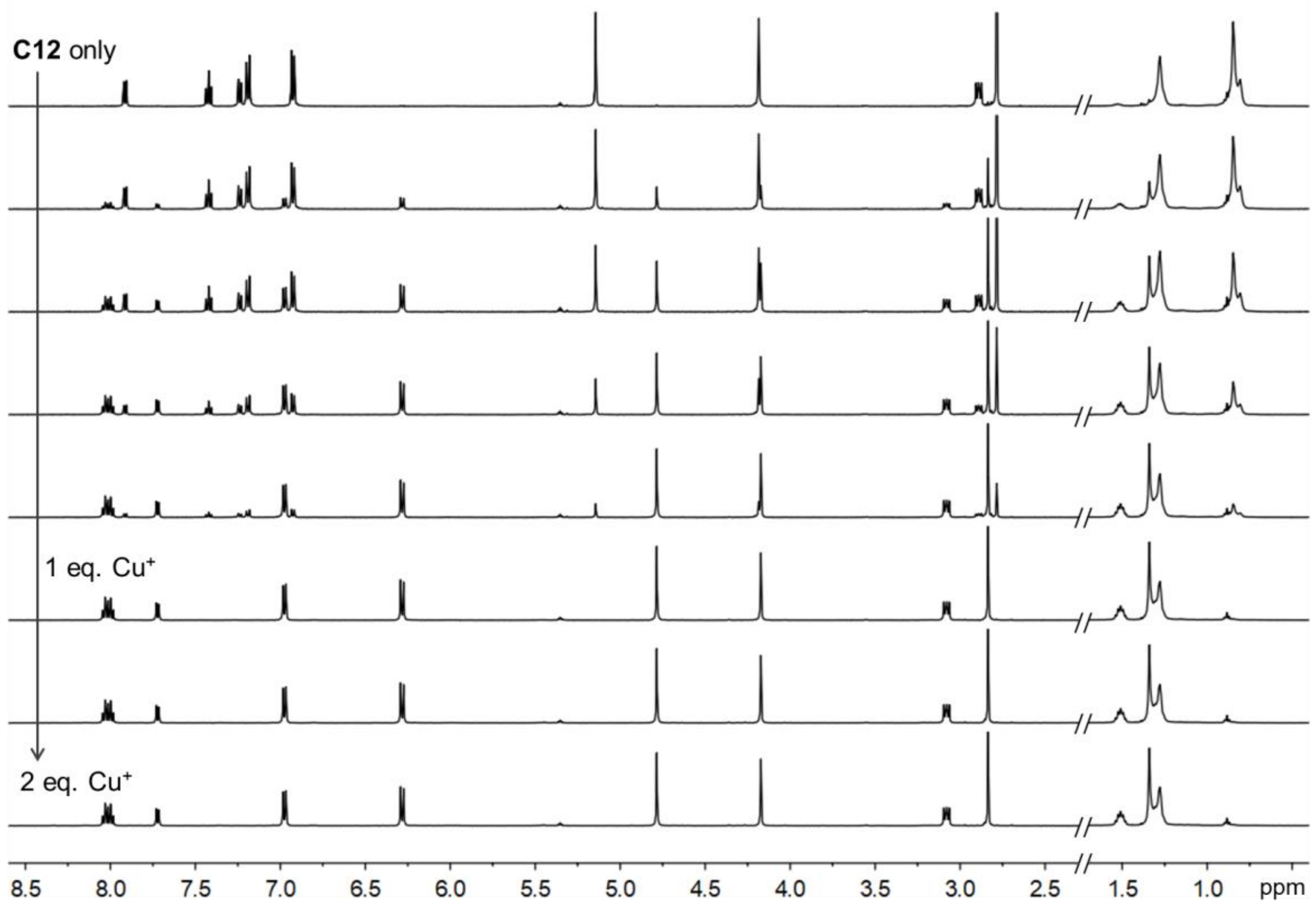

Figure S22. Partial ^{1}H NMR spectra (500 MHz, 298 K, CD_3CN) of a 1 mM solution of **C12** in the presence of 0 eq. (top), 0.2 eq., 0.4 eq., 0.6 eq., 0.8 eq., 1 eq., 1.5 eq. and 2 eq. (bottom) of $[Cu(MeCN)_4](PF_6)$.

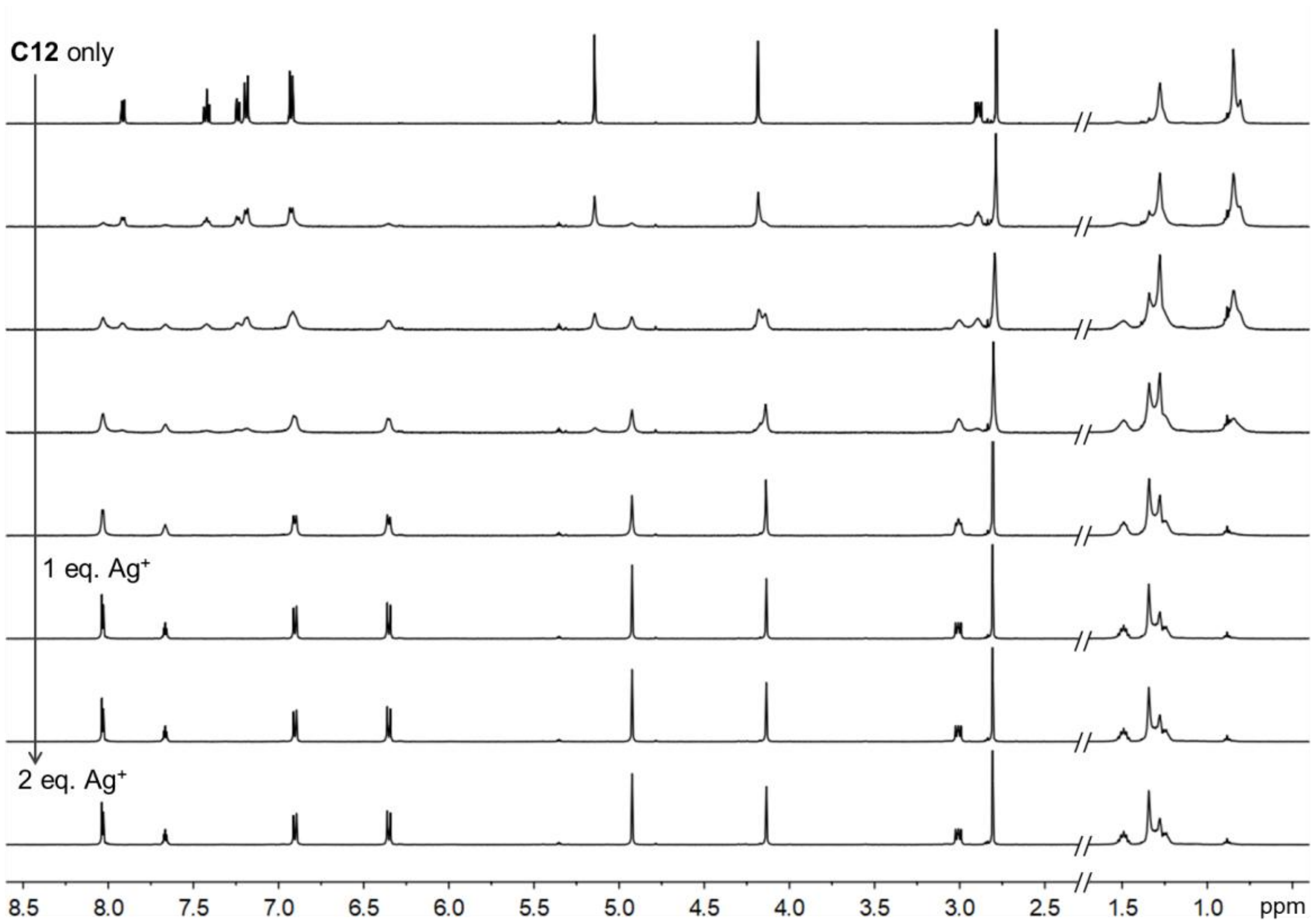

Figure S23. Partial ^{1}H NMR spectra (500 MHz, 298 K, CD_3CN) of a 1 mM solution of **C12** in the presence of 0 eq. (top), 0.2 eq., 0.4 eq., 0.6 eq., 0.8 eq., 1 eq., 1.5 eq. and 2 eq. (bottom) of $AgBF_4$.

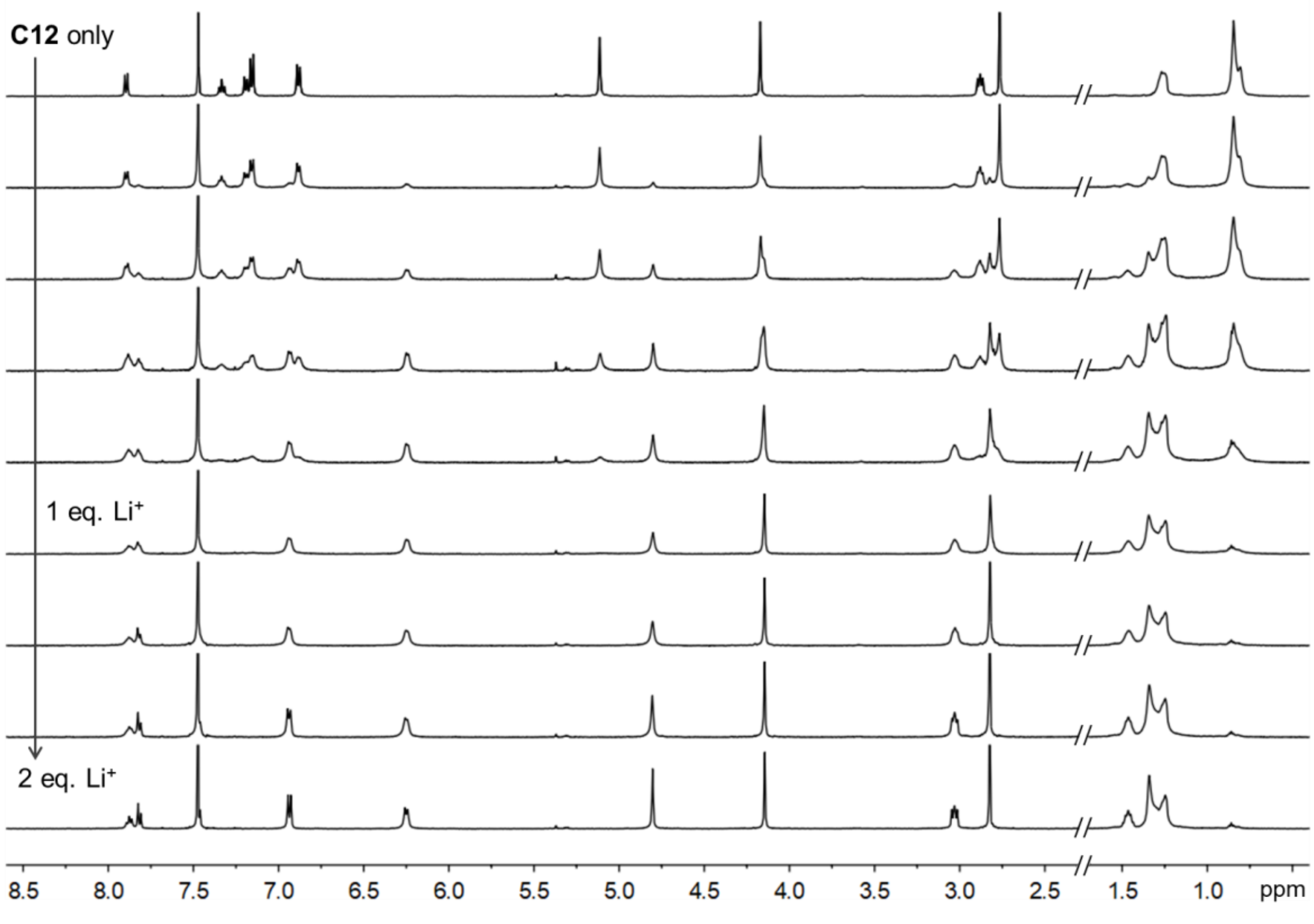

Figure S24. Partial ^{1}H NMR spectra (500 MHz, 298 K, $CD_3CN/CDCl_3$ (1:1, *v*/*v*)) of a 1 mM solution of **C12** in the presence of 0 eq. (top), 0.2 eq., 0.4 eq., 0.6 eq., 0.8 eq., 1 eq., 1.2 eq., 1.5 eq. and 2 eq. (bottom) of LiOTf.

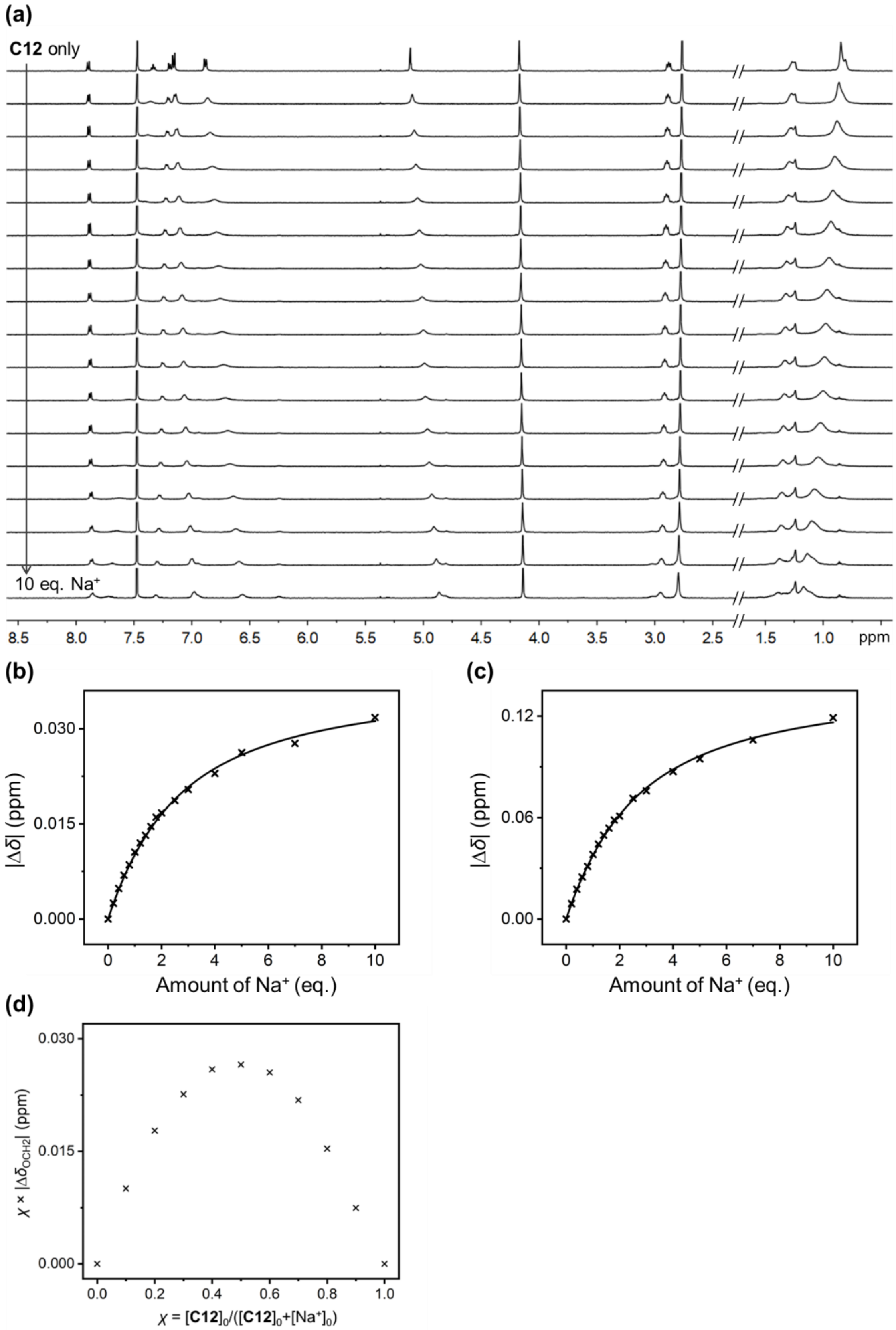

Figure S25. (a) Partial ^{1}H NMR spectra (500 MHz, 298 K, $CD_3CN/CDCl_3$ (1:1, *v*/*v*)) of **C12** in the presence of 0 eq. (top), 0.2 eq., 0.4 eq., 0.6 eq., 0.8 eq., 1 eq., 1.2 eq., 1.4 eq., 1.6 eq., 1.8 eq., 2 eq., 2.5 eq., 3 eq., 4 eq., 5 eq., 7 eq. and 10 eq. (bottom) of NaOTf. Binding isotherms obtained by monitoring chemical shift changes of signals initially at (b) 7.89 ppm and (c) 7.19 ppm. Association constant calculated: $K(Na^+) = 460\ (\pm\ 8)\ M^{-1}$. (d) Job plot of the Na^+ binding to **C12** in $CD_3CN/CDCl_3$ (1:1, *v*/*v*).

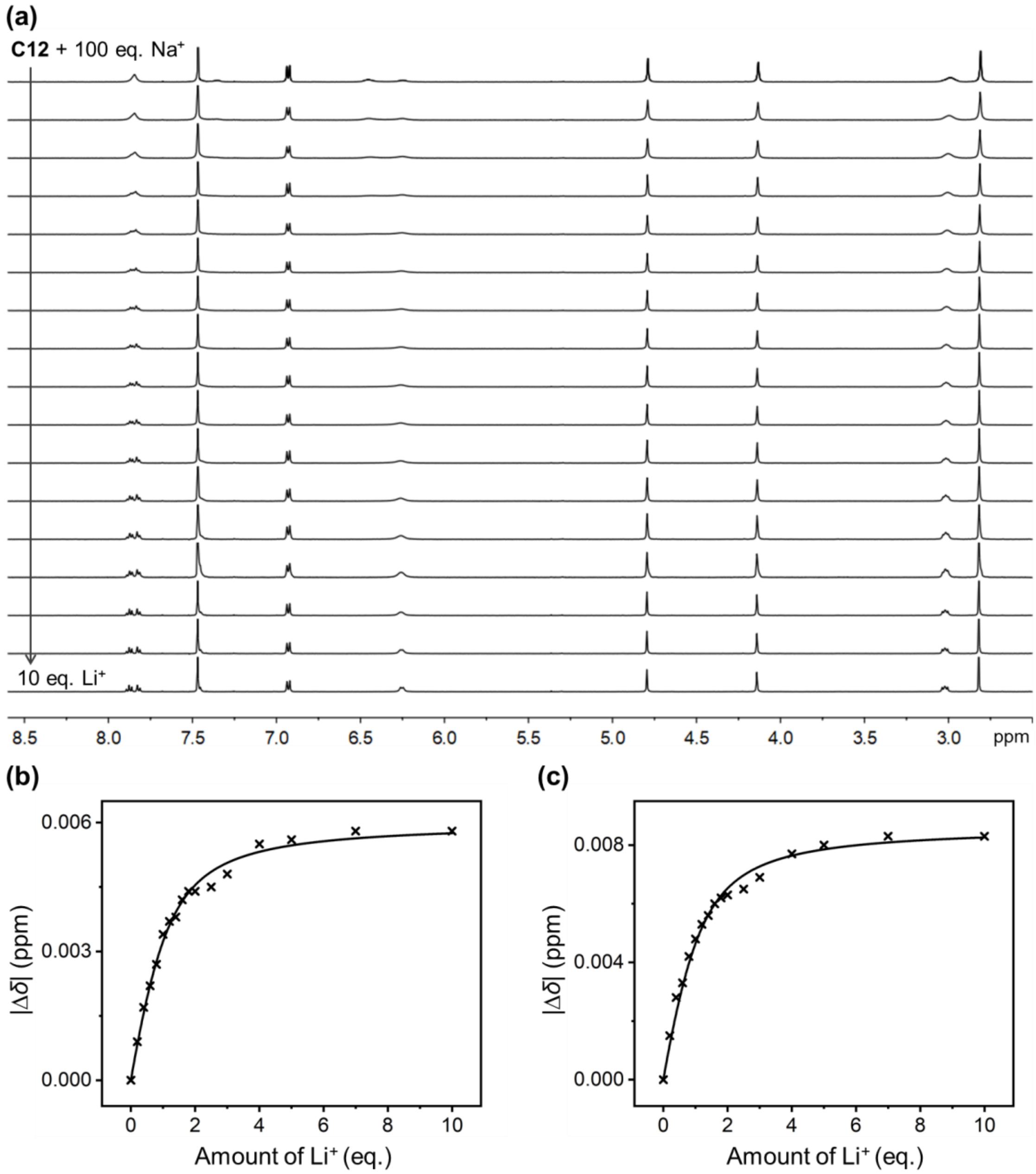

Figure S26. (a) Partial ^{1}H NMR spectra (500 MHz, 298 K, $CD_3CN/CDCl_3$ (1:1, *v*/*v*)) of a mixture of **C12** and 100 eq. of NaOTf (0.1 M) in the presence of 0 eq. (top), 0.2 eq., 0.4 eq., 0.6 eq., 0.8 eq., 1 eq., 1.2 eq., 1.4 eq., 1.6 eq., 1.8 eq., 2 eq., 2.5 eq., 3 eq., 4 eq., 5 eq., 7 eq. and 10 eq. (bottom) of LiOTf. Binding isotherms obtained by monitoring chemical shift changes of signals initially at (b) 4.79 ppm and (c) 4.13 ppm. Apparent association constant calculated: $K_{app} = 250\ (\pm\ 20)\ M^{-1}$. Association constant calculated: $K(Li^+) = 1.1 \times 10^5\ M^{-1}$.

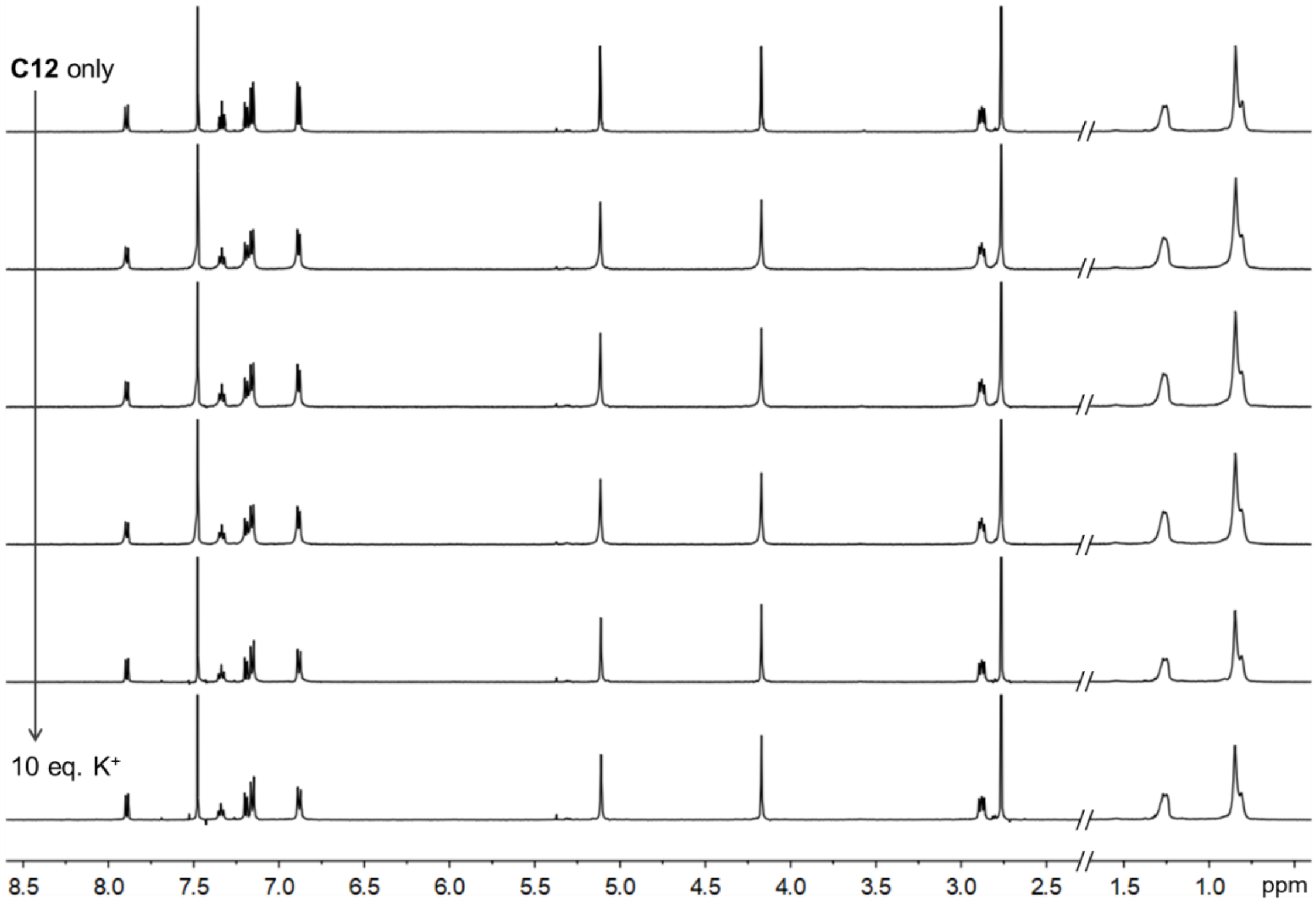

Figure S27. Partial ^{1}H NMR spectra (500 MHz, 298 K, $CD_3CN/CDCl_3$ (1:1, *v*/*v*)) of 1 mM solution of **C12** in the presence of 0 eq. (top), 0.2 eq., 0.5 eq., 1 eq., 5 eq. and 10 eq. (bottom) of KOTf.

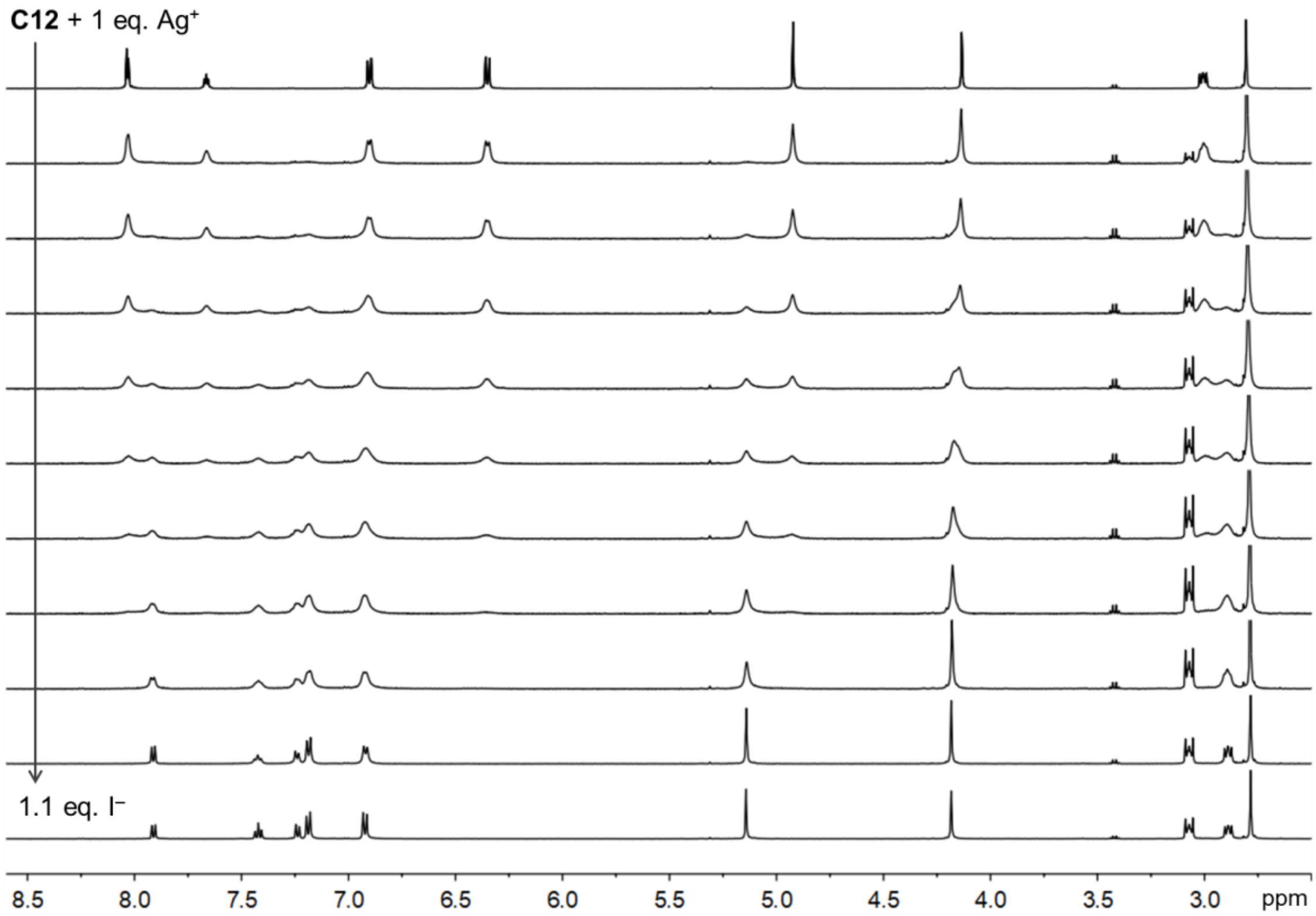

Figure S28. Partial ^{1}H NMR spectra (500 MHz, 298 K, CD_3CN) of a mixture of **C12** and 1 eq. of $AgBF_4$ in the presence of 0 eq. (top), 0.2 eq., 0.3 eq., 0.4 eq., 0.5 eq., 0.6 eq., 0.7 eq., 0.8 eq., 0.9 eq., 1 eq. and 1.1 eq. (bottom) of TBAI.

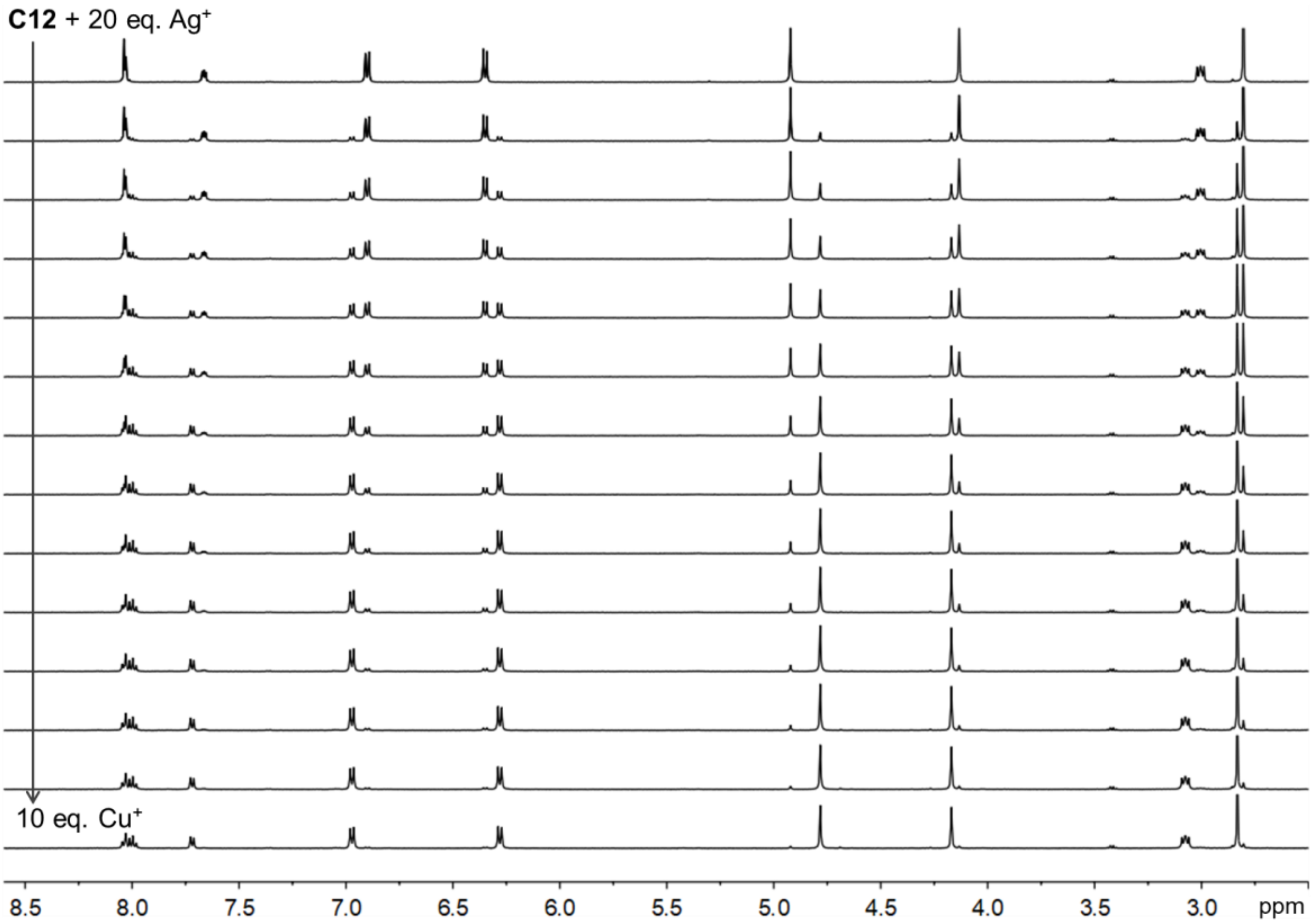

Figure S29. Partial ^{1}H NMR spectra (500 MHz, 298 K, CD_3CN) of a mixture of **C12** and 20 eq. of $AgBF_4$ in the presence of 0 eq. (top), 0.2 eq., 0.4 eq., 0.6 eq., 0.8 eq., 1 eq., 1.5 eq., 2 eq., 2.5 eq., 3 eq., 4 eq., 5 eq., 7 eq. and 10 eq. (bottom) of $[Cu(MeCN)_4](PF_6)$.

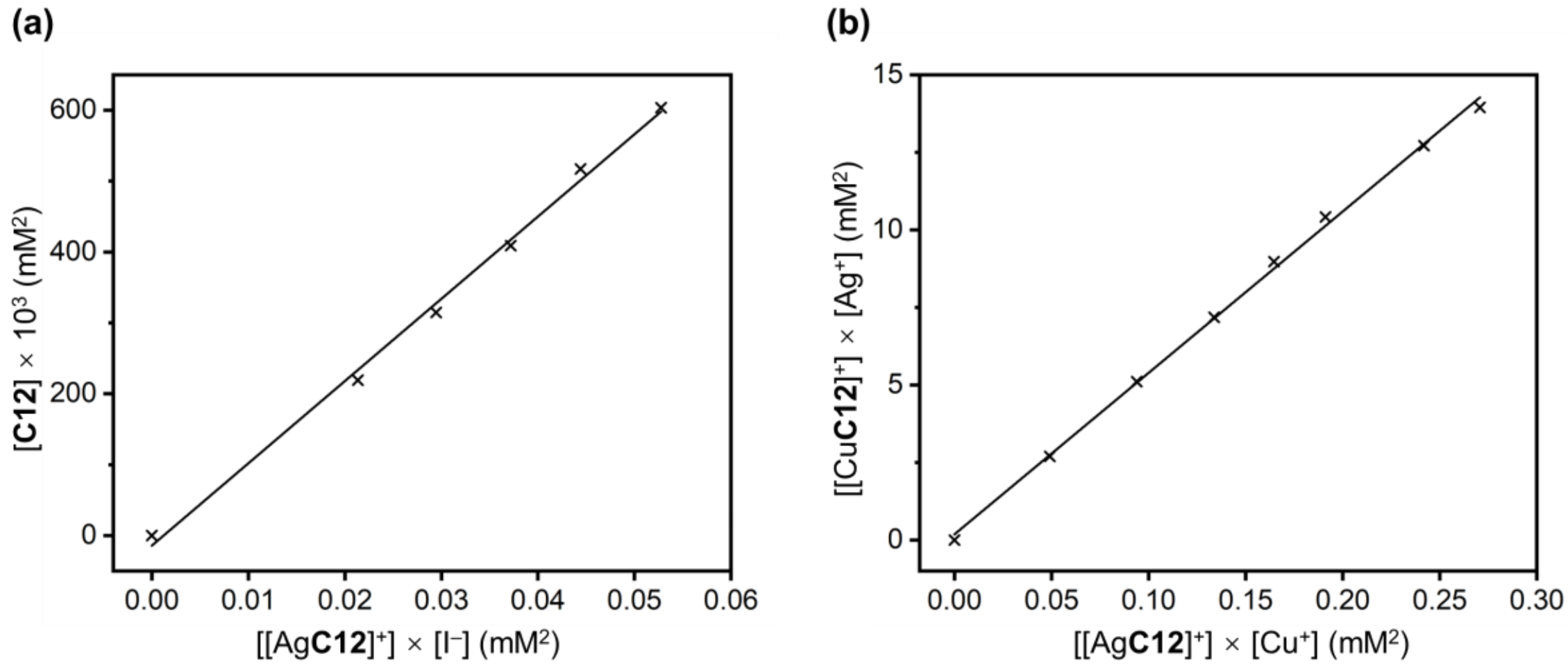

Figure S30. (a) Curve obtained by linear fitting using the equilibrium concentrations in the Ag^+ extraction from [Ag**C12**]$^+$ by I^- in CD_3CN. Apparent equilibrium constant calculated $K_{app} = 11600 \pm 400$ ($R^2 = 0.996$). Association constant calculated: $K(Ag^+) = 1.4 \times 10^{10}$ M^{-1}. (b) Curve obtained by linear fitting using the equilibrium concentrations in the Ag^+ replacement from [Ag**C12**]$^+$ by Cu^+ in CD_3CN. Apparent equilibrium constant calculated $K_{app} = 52 \pm 1$ ($R^2 = 0.998$). Association constant calculated: $K(Cu^+) = 7.1 \times 10^{11}$ M^{-1}.

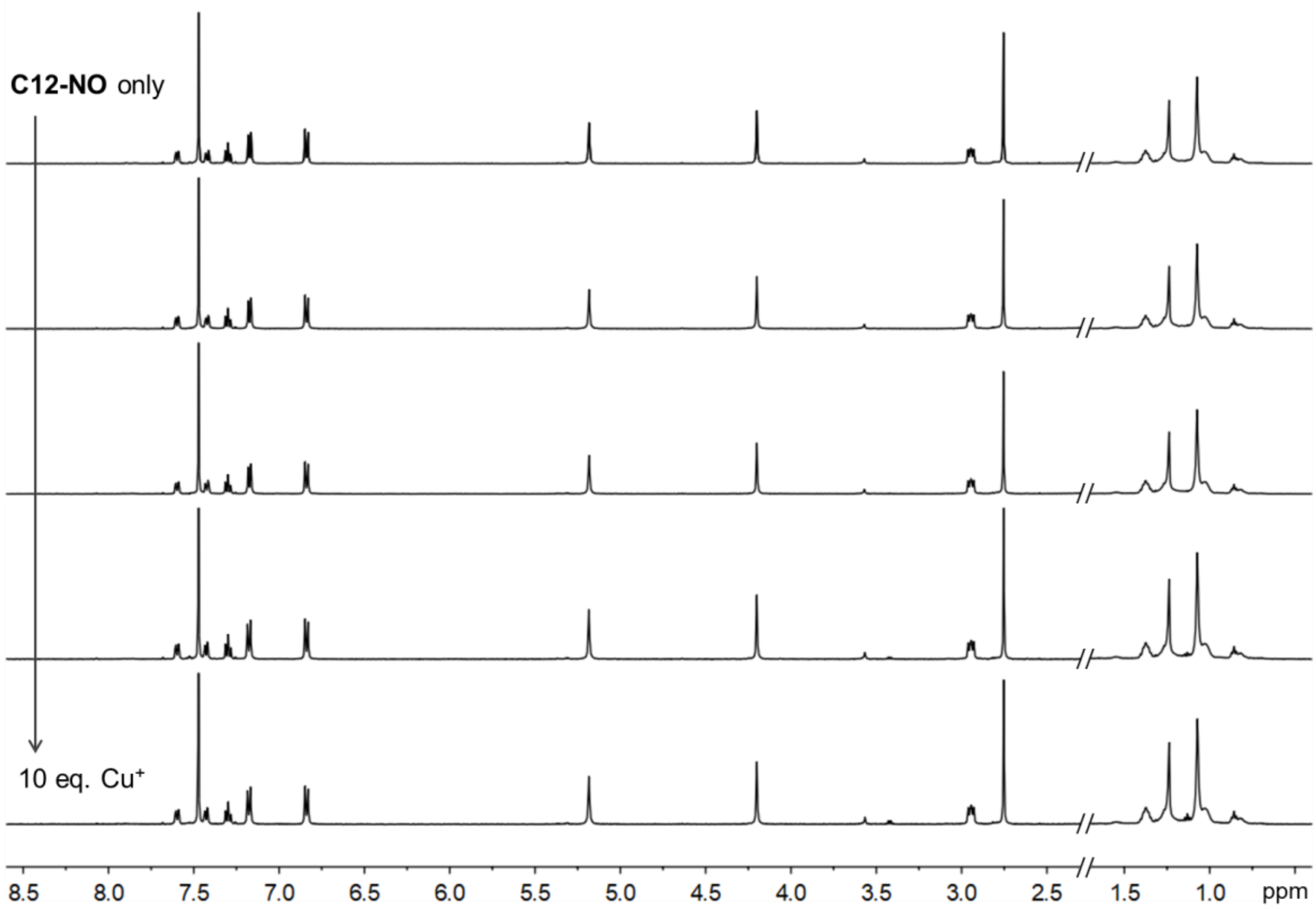

Figure S31. Partial ^{1}H NMR spectra (500 MHz, 298 K, $CD_3CN/CDCl_3$ (1:1, *v*/*v*)) of 1 mM solution of **C12-NO** in the presence of 0 eq. (top), 0.5 eq., 1 eq., 5 eq. and 10 eq. (bottom) of $[Cu(MeCN)_4](PF_6)$.

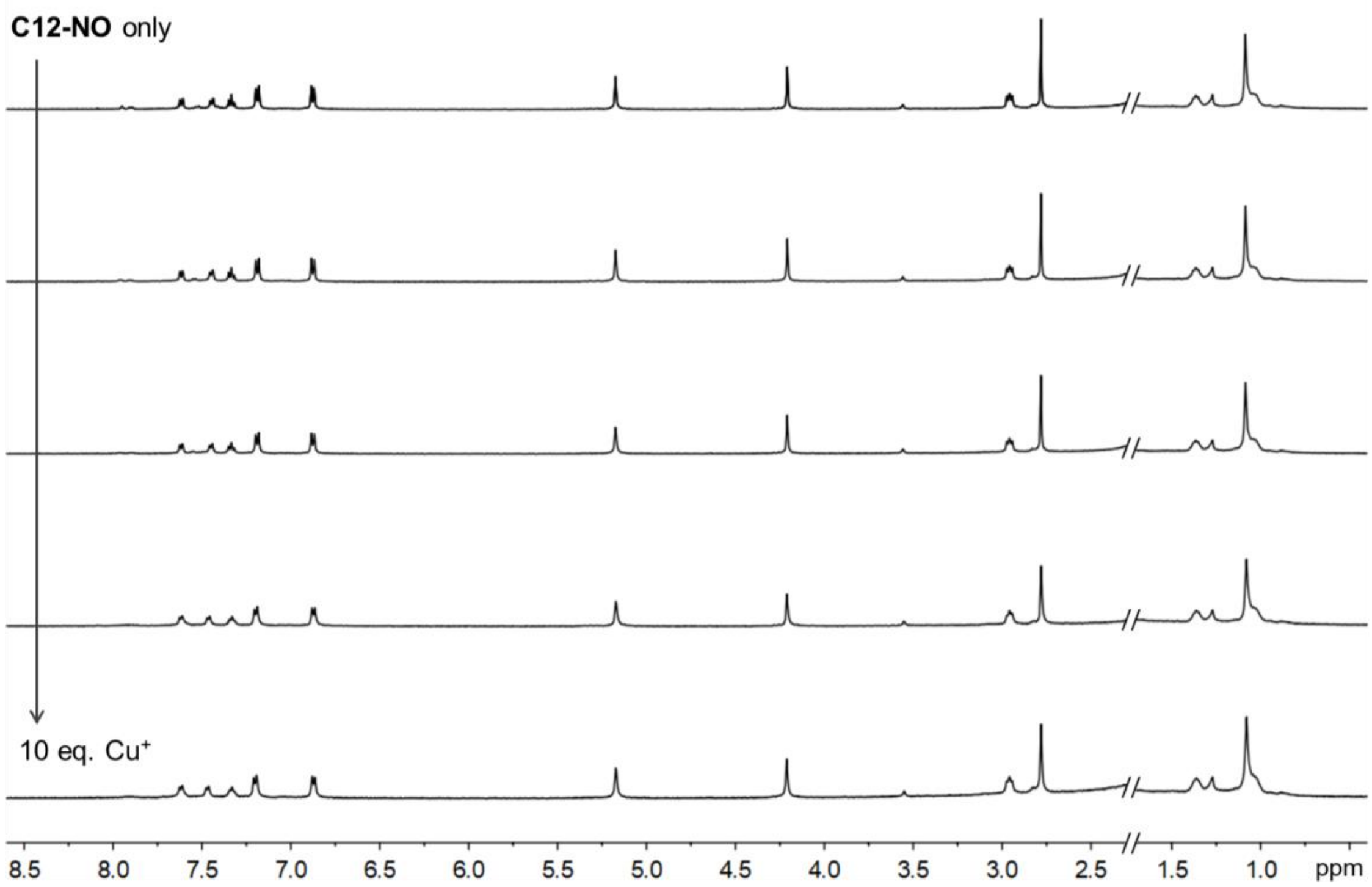

Figure S32. Partial ^{1}H NMR spectra (500 MHz, 298 K, CD_3CN) of 1 mM solution of **C12-NO** in the presence of 0 eq. (top), 0.5 eq., 1 eq., 5 eq. and 10 eq. (bottom) of $[Cu(MeCN)_4](PF_6)$.

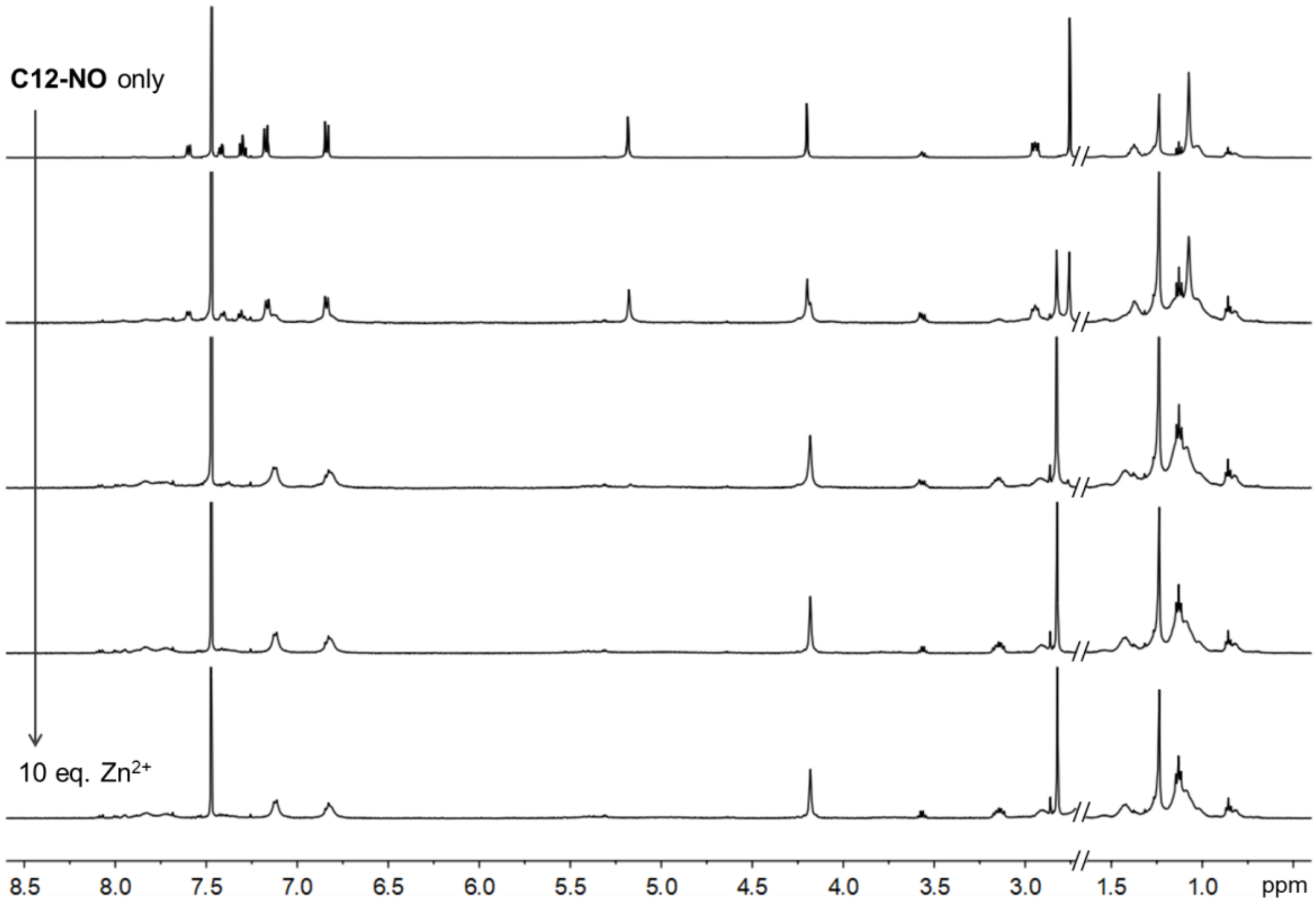

Figure S33. Partial ^{1}H NMR spectra (500 MHz, 298 K, $CD_3CN/CDCl_3$ (1:1, *v*/*v*)) of 1 mM solution of **C12-NO** in the presence of 0 eq. (top), 0.5 eq., 1 eq., 5 eq. and 10 eq. (bottom) of $Zn(ClO_4)_2$.

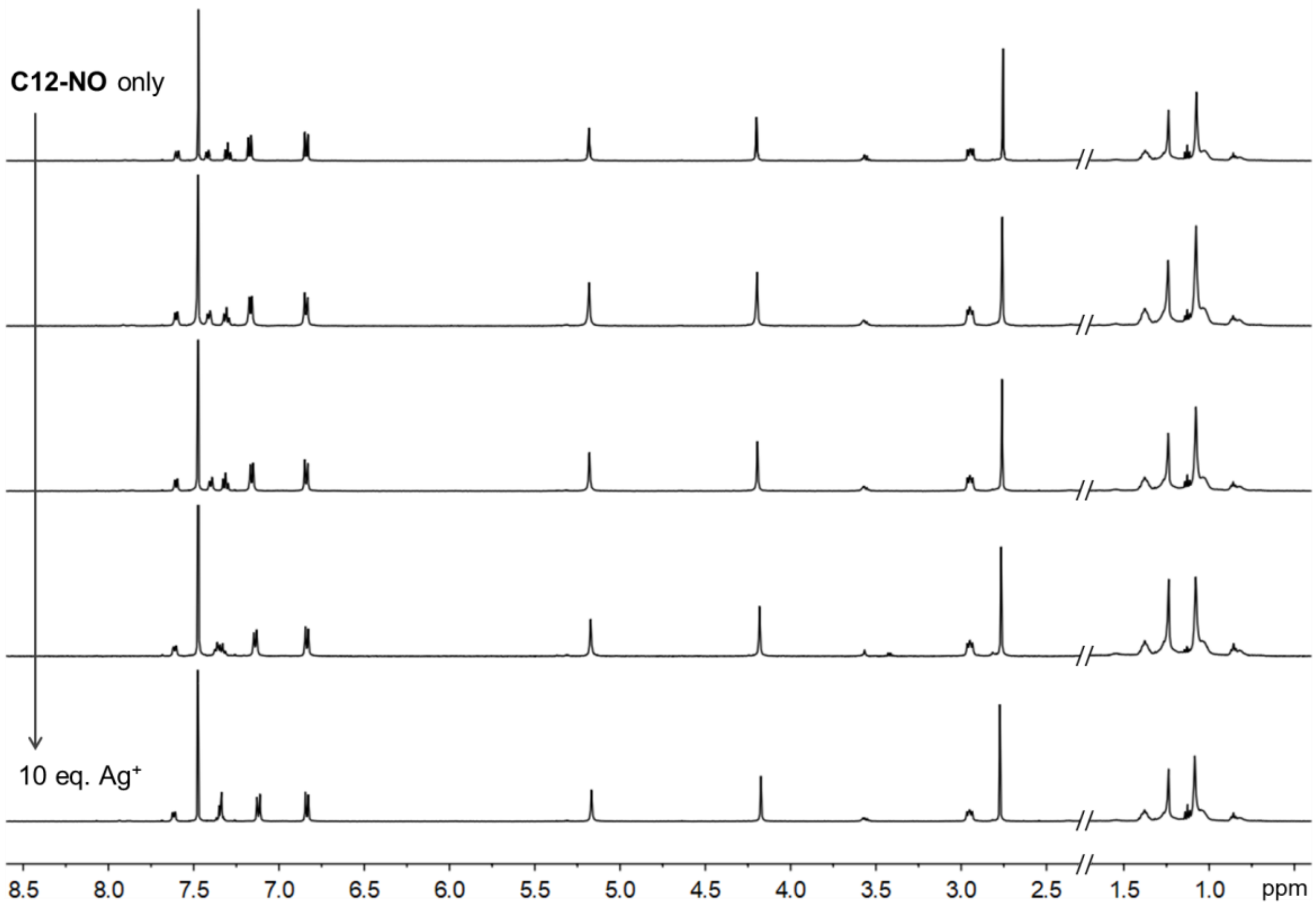

Figure S34. Partial ^{1}H NMR spectra (500 MHz, 298 K, $CD_3CN/CDCl_3$ (1:1, *v*/*v*)) of 1 mM solution of **C12-NO** in the presence of 0 eq. (top), 0.5 eq., 1 eq., 5 eq. and 10 eq. (bottom) of $AgBF_4$.

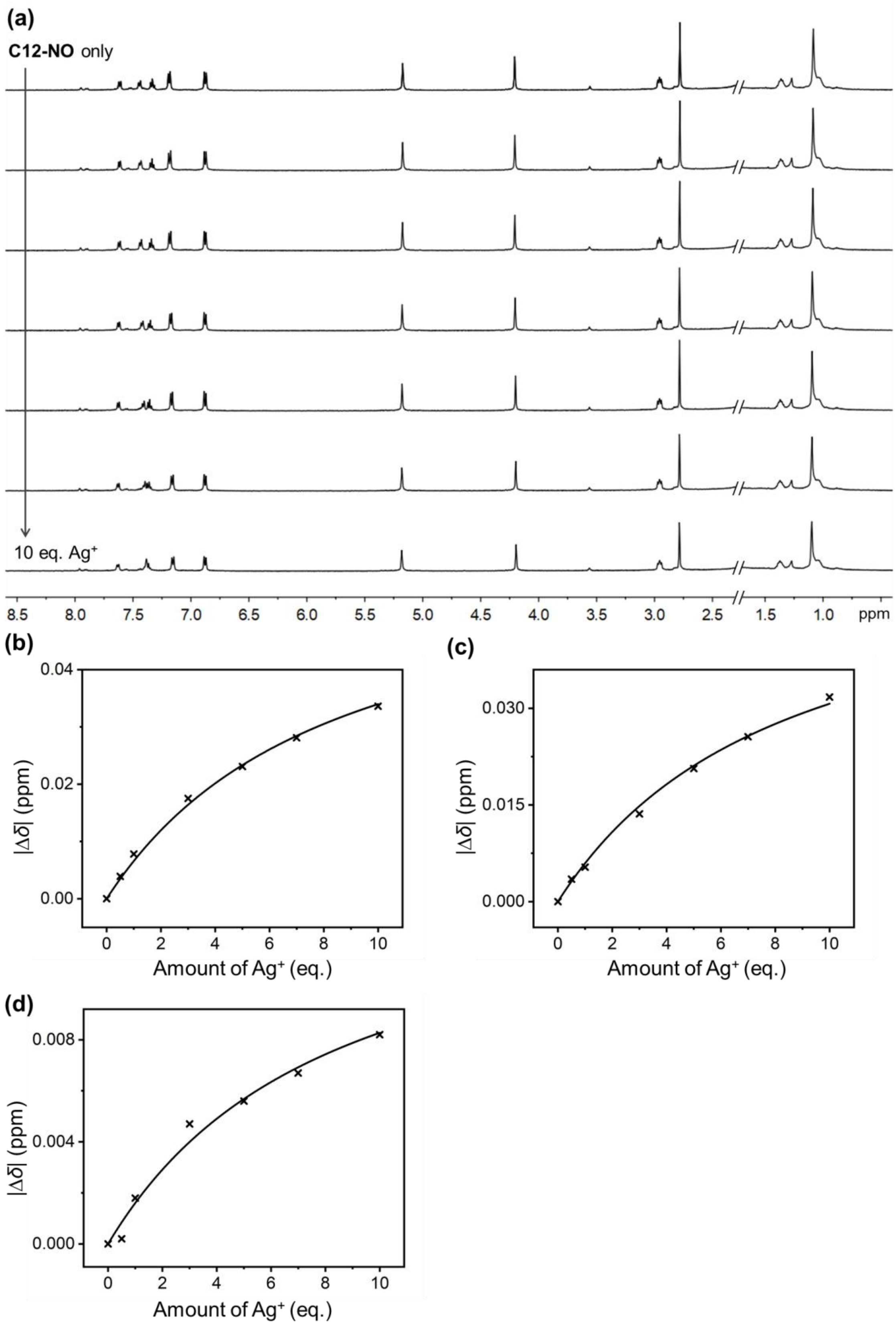

Figure S35. (a) Partial ^{1}H NMR spectra (500 MHz, 298 K, CD_3CN) of **C12-NO** in the presence of 0 eq. (top), 0.5 eq., 1 eq., 3 eq., 5 eq., 7 eq. and 10 eq. (bottom) of $AgBF_4$. Binding isotherms obtained by monitoring chemical shift changes of signals initially at (b) 7.33 ppm, (c) 7.19 ppm and (d) 5.17 ppm. Association constant calculated: $K(Ag^{+}) = 110\ (\pm 4)\ M^{-1}$.

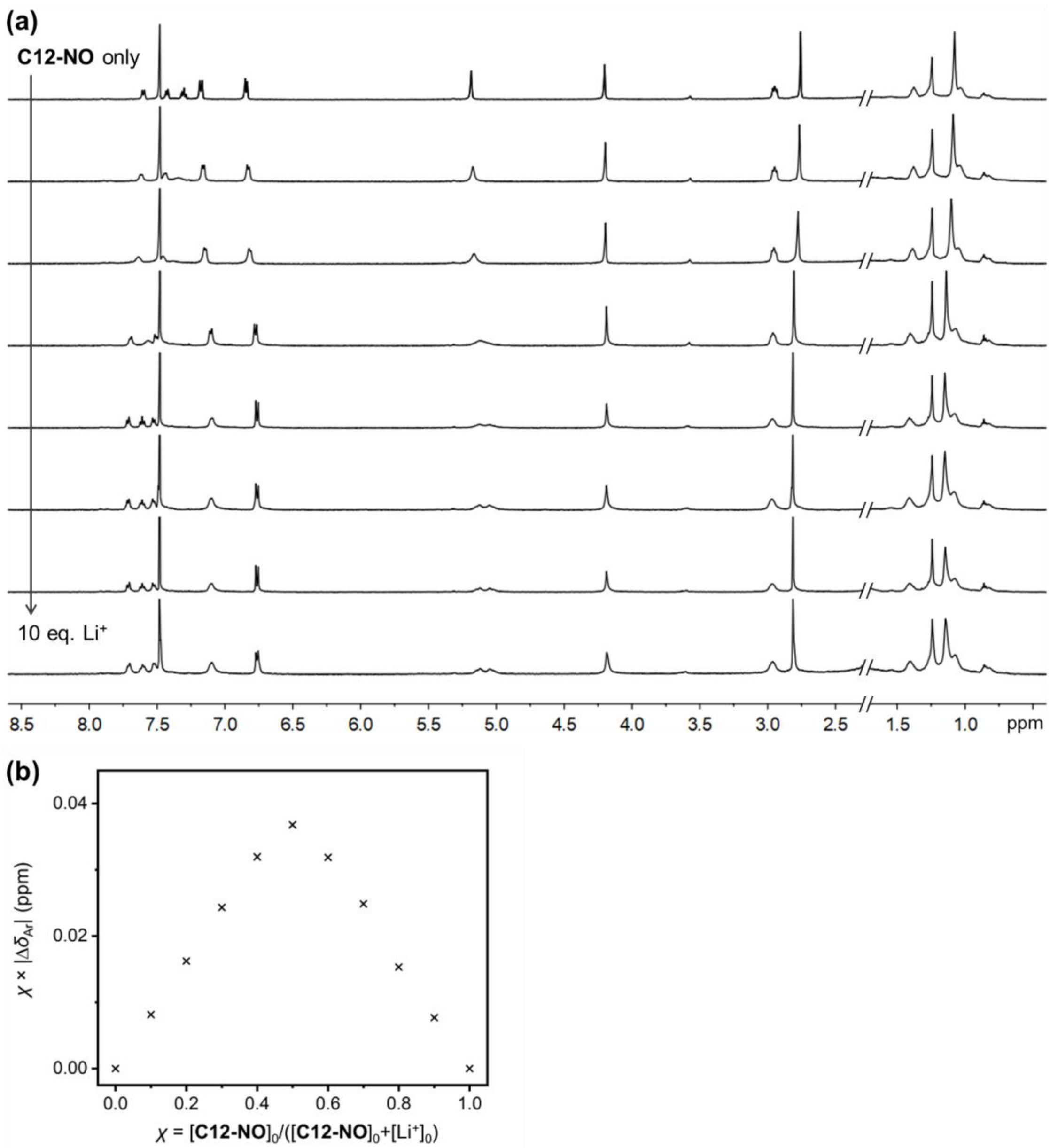

Figure S36. (a) Partial ^{1}H NMR spectra (500 MHz, 298 K, $CD_3CN/CDCl_3$ (1:1, *v*/*v*)) of 1 mM solution of **C12-NO** in the presence of 0 eq. (top), 0.2 eq., 0.4 eq., 1 eq., 1.4 eq., 2 eq., 5 eq. and 10 eq. (bottom) of LiOTf. (b) Job plot of the Li^+ binding to **C12-NO** in $CD_3CN/CDCl_3$ (1:1, *v*/*v*).

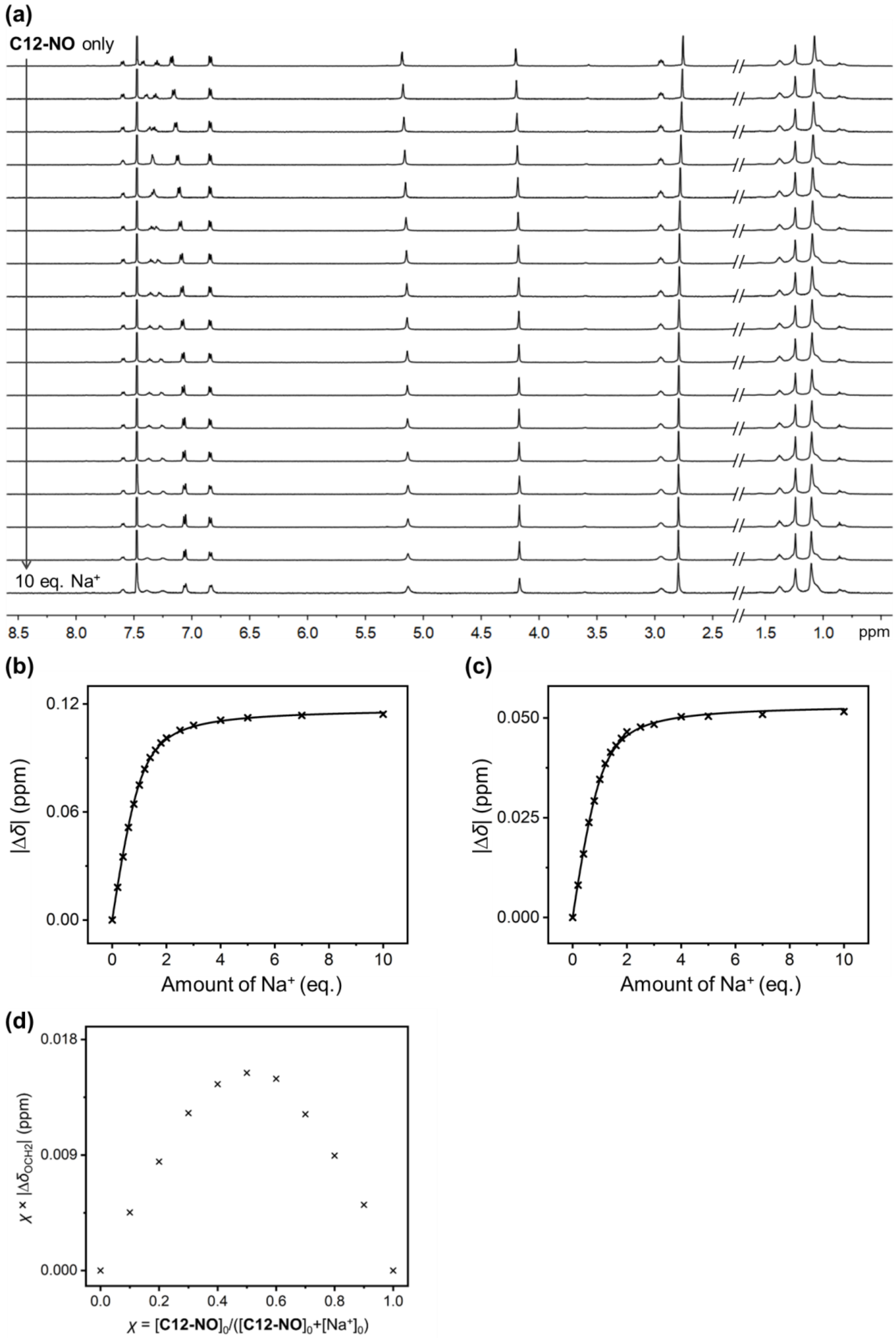

Figure S37. (a) Partial ^{1}H NMR spectra (500 MHz, 298 K, $CD_3CN/CDCl_3$ (1:1, *v*/*v*)) of **C12-NO** in the presence of 0 eq. (top), 0.2 eq., 0.4 eq., 0.6 eq., 0.8 eq., 1 eq., 1.2 eq., 1.4 eq., 1.6 eq., 1.8 eq., 2 eq., 2.5 eq., 3 eq., 4 eq., 5 eq., 7 eq. and 10 eq. (bottom) of NaOTf. Binding isotherms obtained by monitoring chemical shift changes of signals initially at (b) 7.17 ppm and (c) 5.18 ppm. Association constant calculated: $K(Na^+) = 5100\ (\pm\ 140)\ M^{-1}$. (d) Job plot of the Na^+ binding to **C12-NO** in $CD_3CN/CDCl_3$ (1:1, *v*/*v*).

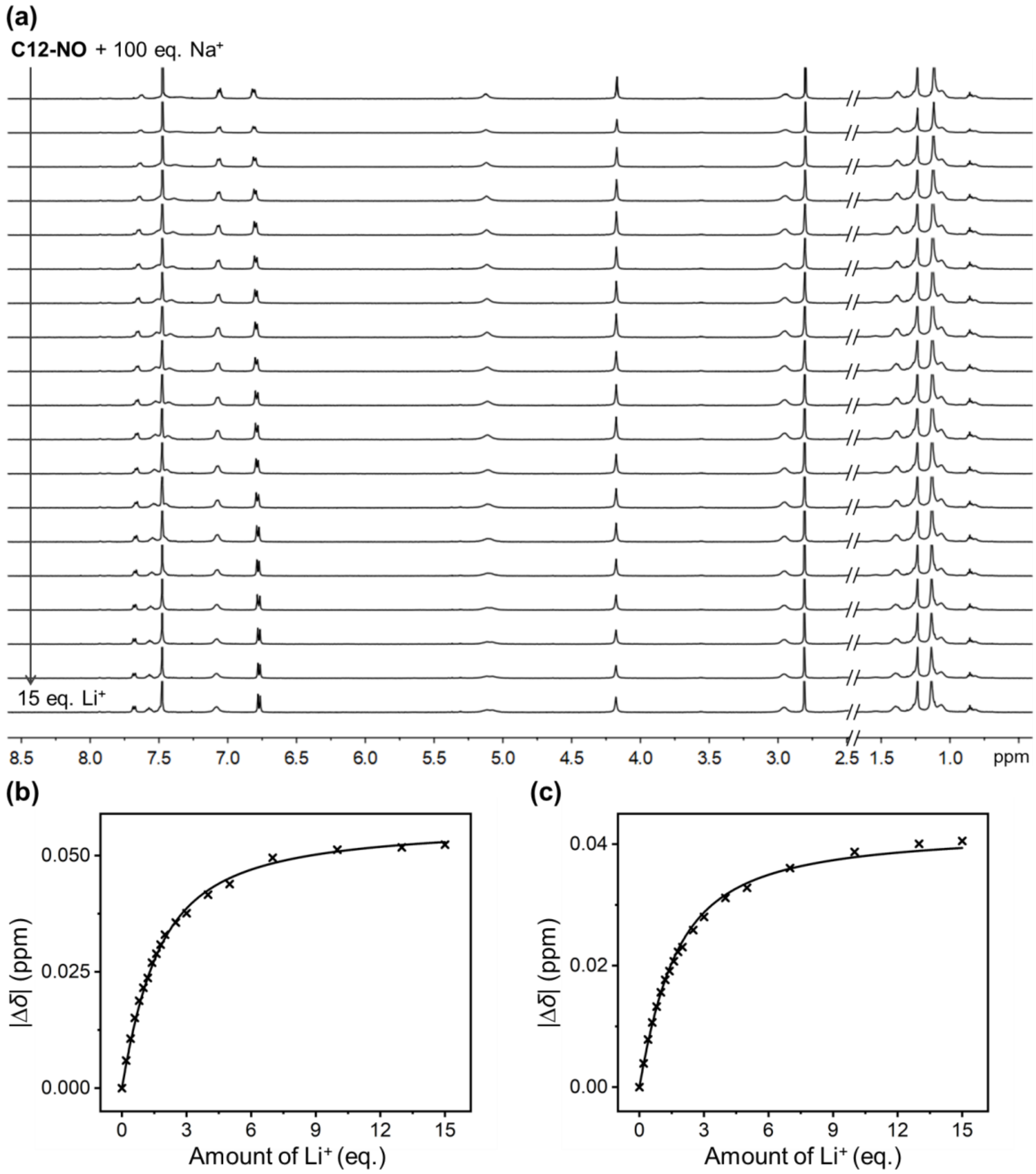

Figure S38. (a) Partial ^{1}H NMR spectra (500 MHz, 298 K, $CD_3CN/CDCl_3$ (1:1, *v*/*v*)) of a mixture of **C12-NO** and 100 eq. of NaOTf (0.1 M) in the presence of 0 eq. (top), 0.2 eq., 0.4 eq., 0.6 eq., 0.8 eq., 1 eq., 1.2 eq., 1.4 eq., 1.6 eq., 1.8 eq., 2 eq., 2.5 eq., 3 eq., 4 eq., 5 eq., 7 eq., 10 eq., 13 eq. and 15 eq. (bottom) of LiOTf. Binding isotherms obtained by monitoring chemical shift changes of signals initially at (b) 7.63 ppm and (c) 6.81 ppm. Apparent association constant calculated: K_{app} = 90 (± 3) M^{-1}. Association constant calculated: $K(Li^+) = 4.5 \times 10^5\ M^{-1}$.

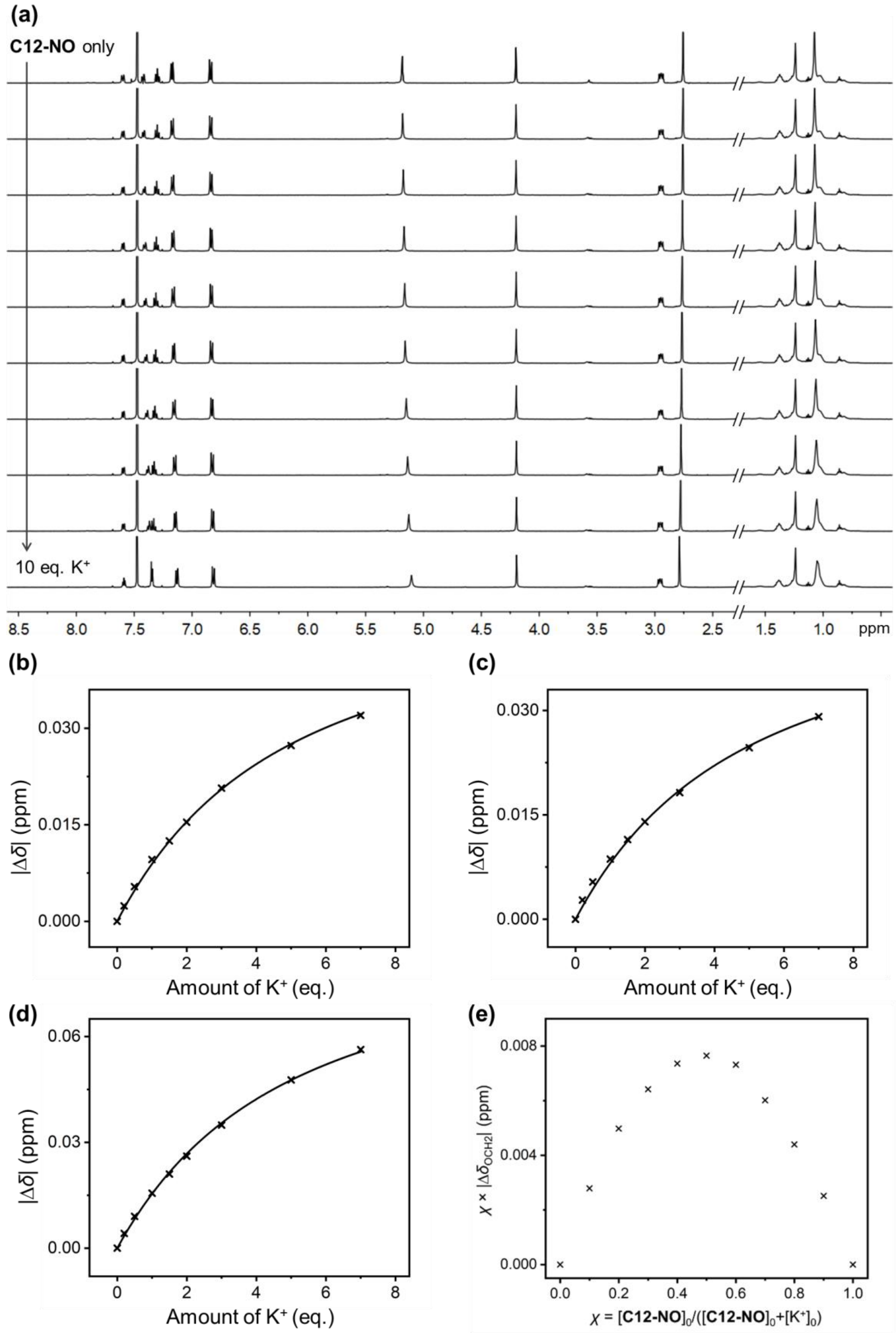

Figure S39. (a) Partial ^{1}H NMR spectra (500 MHz, 298 K, $CD_3CN/CDCl_3$ (1:1, *v*/*v*)) of **C12-NO** in the presence of 0 eq. (top), 0.2 eq., 0.5 eq., 1 eq., 1.5 eq., 2 eq., 3 eq., 5 eq., 7 eq. and 10 eq. (bottom) of KOTf. Binding isotherms obtained by monitoring chemical shift changes of signals initially at (b) 7.30 ppm, (c) 7.17 ppm and (d) 5.18 ppm. Association constant calculated: $K(K^+) = 210\ (\pm\ 4)\ M^{-1}$. (e) Job plot of the K^+ binding to **C12-NO** in $CD_3CN/CDCl_3$ (1:1, *v*/*v*).

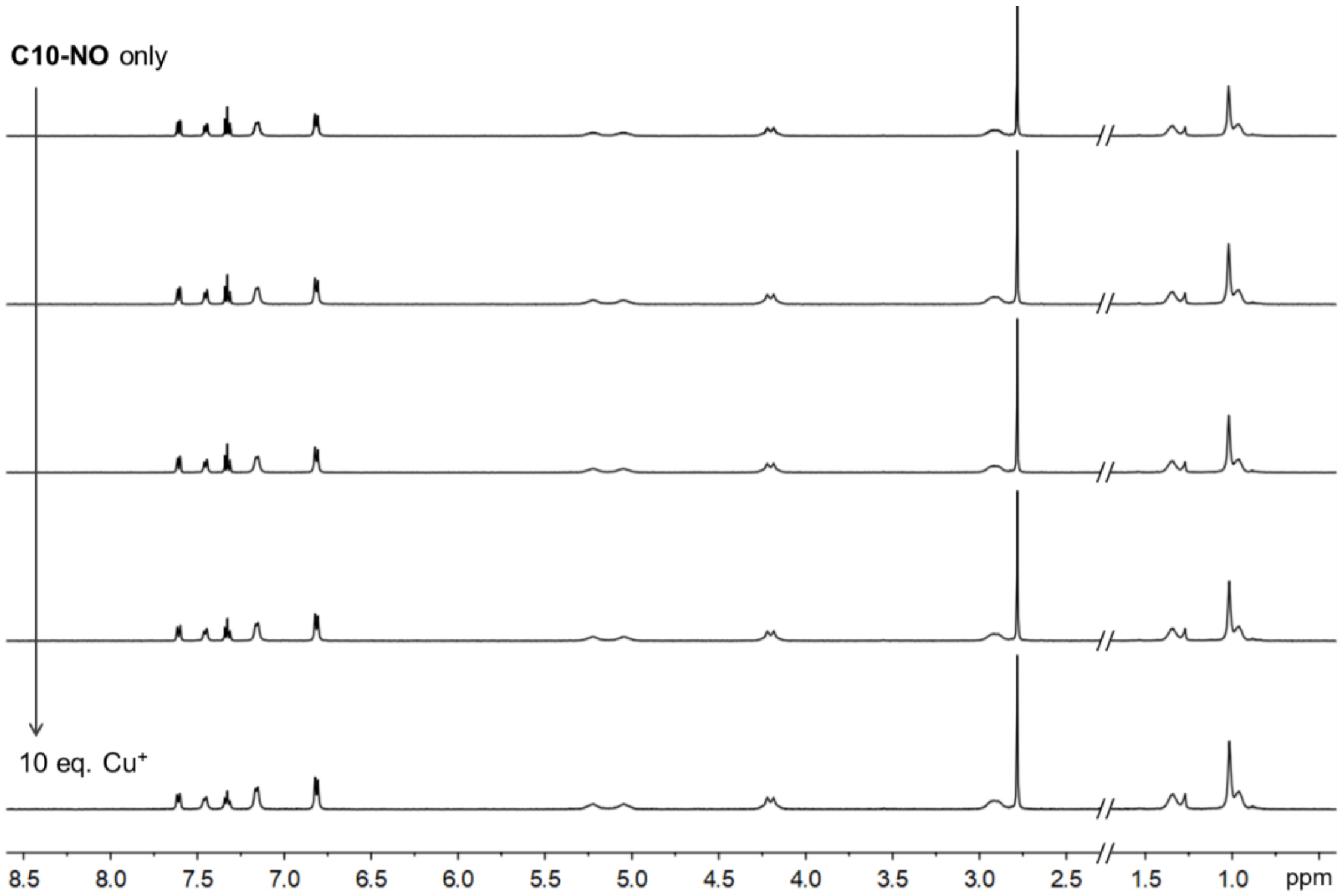

Figure S40. Partial ^{1}H NMR spectra (500 MHz, 298 K, CD_3CN) of 1 mM solution of **C10-NO** in the presence of 0 eq. (top), 0.5 eq., 1 eq., 5 eq. and 10 eq. (bottom) of $[Cu(MeCN)_4](PF_6)$.

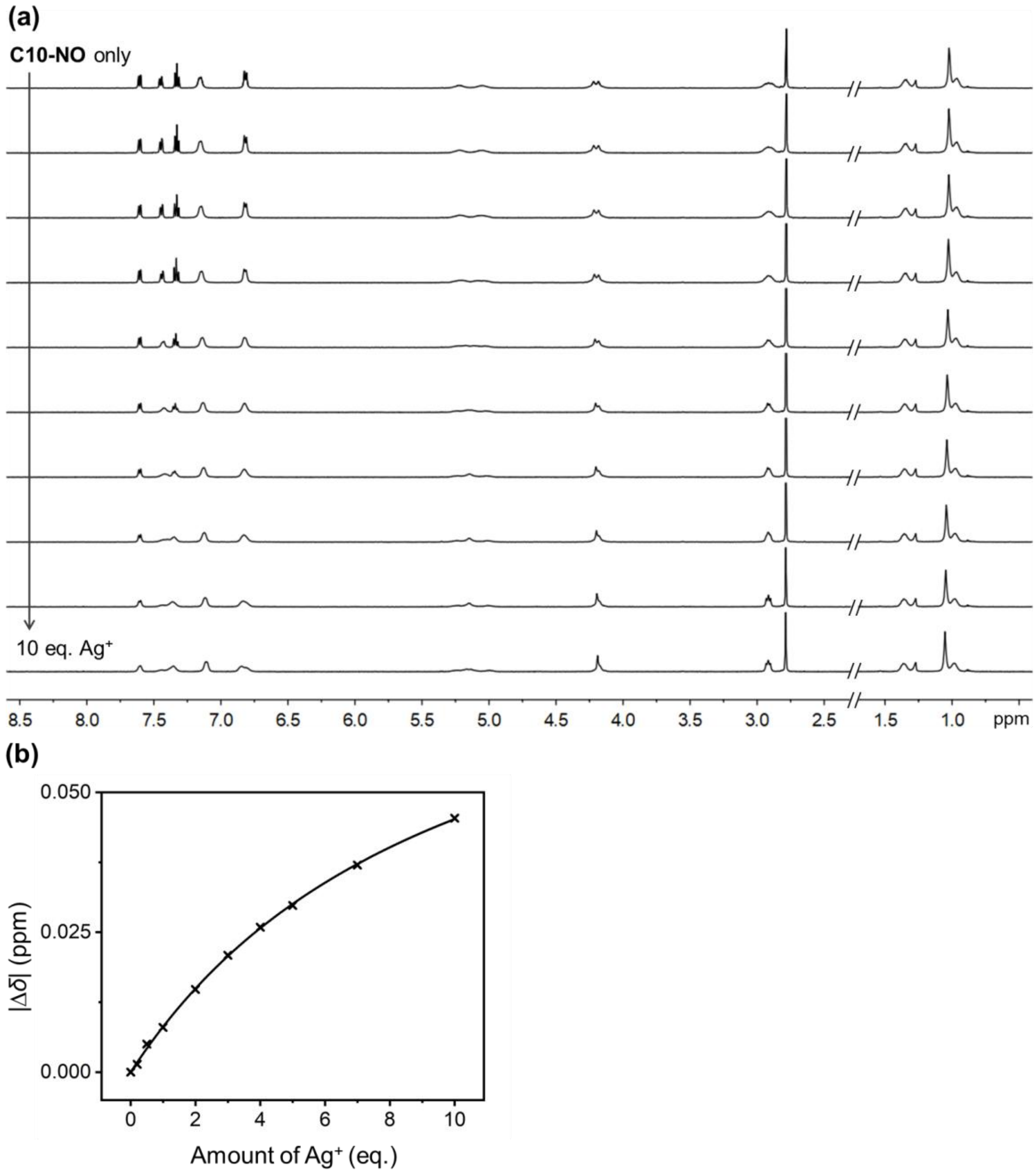

Figure S41. (a) Partial ^{1}H NMR spectra (500 MHz, 298 K, CD_3CN) of **C10-NO** in the presence of 0 eq. (top), 0.2 eq., 0.5 eq., 1 eq., 2 eq., 3 eq., 4 eq., 5 eq., 7 eq. and 10 eq. (bottom) of $AgBF_4$. Binding isotherm obtained by monitoring the chemical shift changes of the signal initially at (b) 7.15 ppm. Association constant calculated: $K(Ag^{+}) = 90\ (\pm\ 1)\ M^{-1}$.

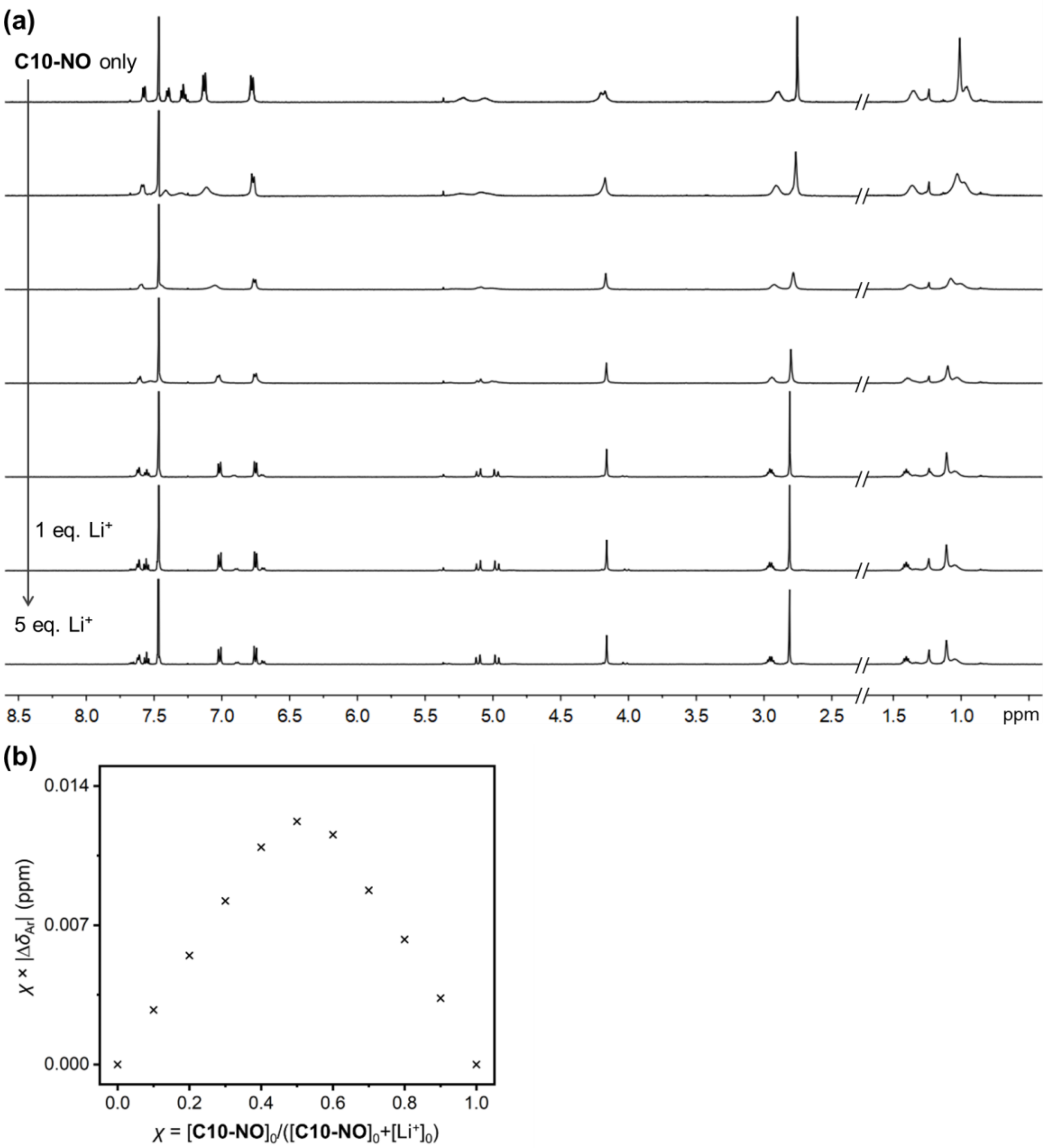

Figure S42. (a) Partial ^{1}H NMR spectra (500 MHz, 298 K, $CD_3CN/CDCl_3$ (1:1, *v*/*v*)) of 1 mM solution of **C10-NO** in the presence of 0 eq. (top), 0.2 eq., 0.4 eq., 0.6 eq., 0.8 eq., 1 eq. and 5 eq. (bottom) of LiOTf. (b) Job plot of the Li^+ binding to **C10-NO** in $CD_3CN/CDCl_3$ (1:1, *v*/*v*).

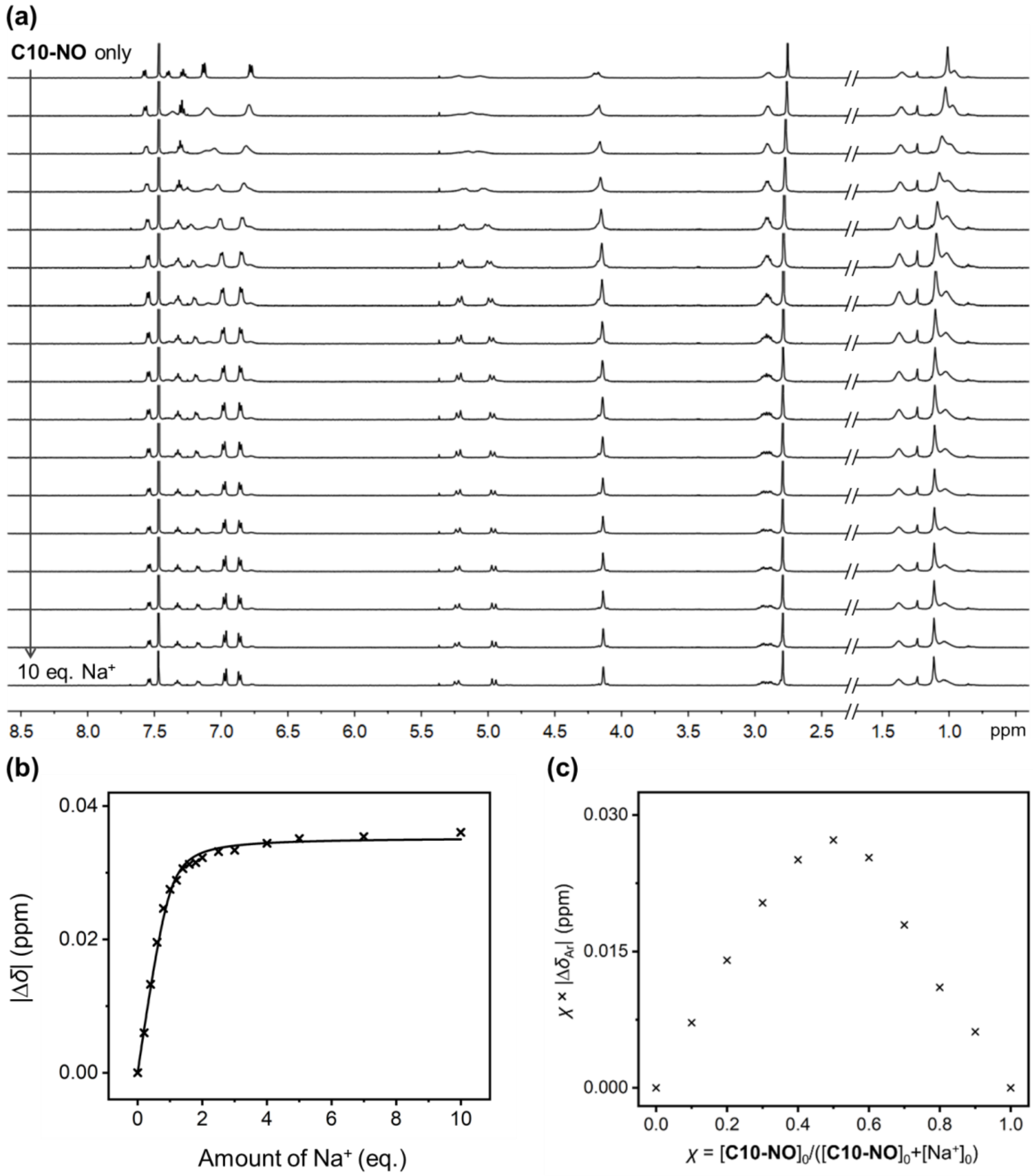

Figure S43. (a) Partial ^{1}H NMR spectra (500 MHz, 298 K, $CD_3CN/CDCl_3$ (1:1, *v*/*v*)) of **C10-NO** in the presence of 0 eq. (top), 0.2 eq., 0.4 eq., 0.6 eq., 0.8 eq., 1 eq., 1.2 eq., 1.4 eq., 1.6 eq., 1.8 eq., 2 eq., 2.5 eq., 3 eq., 4 eq., 5 eq., 7 eq. and 10 eq. (bottom) of NaOTf. Binding isotherm obtained by monitoring chemical shift changes of the signal initially at (b) 7.57 ppm. Association constant calculated: $K(Na^+) = 1.4\ (\pm\ 0.3) \times 10^{4}\ M^{-1}$. (c) Job plot of the Na^+ binding to **C10-NO** in $CD_3CN/CDCl_3$ (1:1, *v*/*v*).

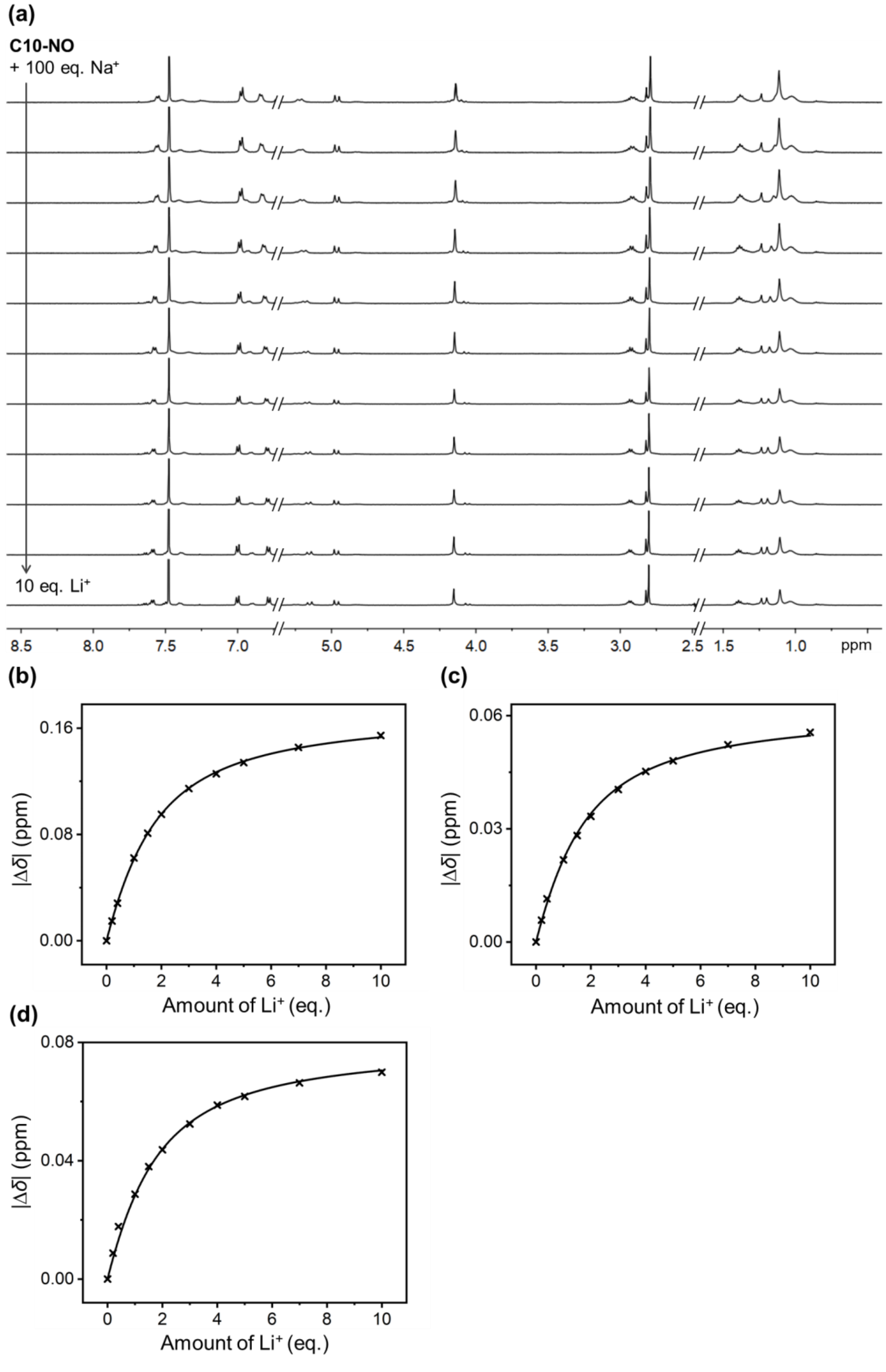

Figure S44. (a) Partial ^{1}H NMR spectra (500 MHz, 298 K, $CD_3CN/CDCl_3$ (1:1, *v*/*v*)) of a mixture of **C10-NO** and 100 eq. of NaOTf (0.1 M) in the presence of 0 eq. (top), 0.2 eq., 0.4 eq., 1 eq., 1.5 eq., 2 eq., 3 eq., 4 eq., 5 eq., 7 eq. and 10 eq. (bottom) of LiOTf. Binding isotherms obtained by monitoring chemical shift changes of signals initially at (b) 7.24 ppm, (c) 6.84 ppm and (d) 5.22 ppm. Apparent association constant calculated: K_{app} = 80 (± 2) M^{-1}. Association constant calculated: $K(Li^+) = 1.1 \times 10^6$ M^{-1}.

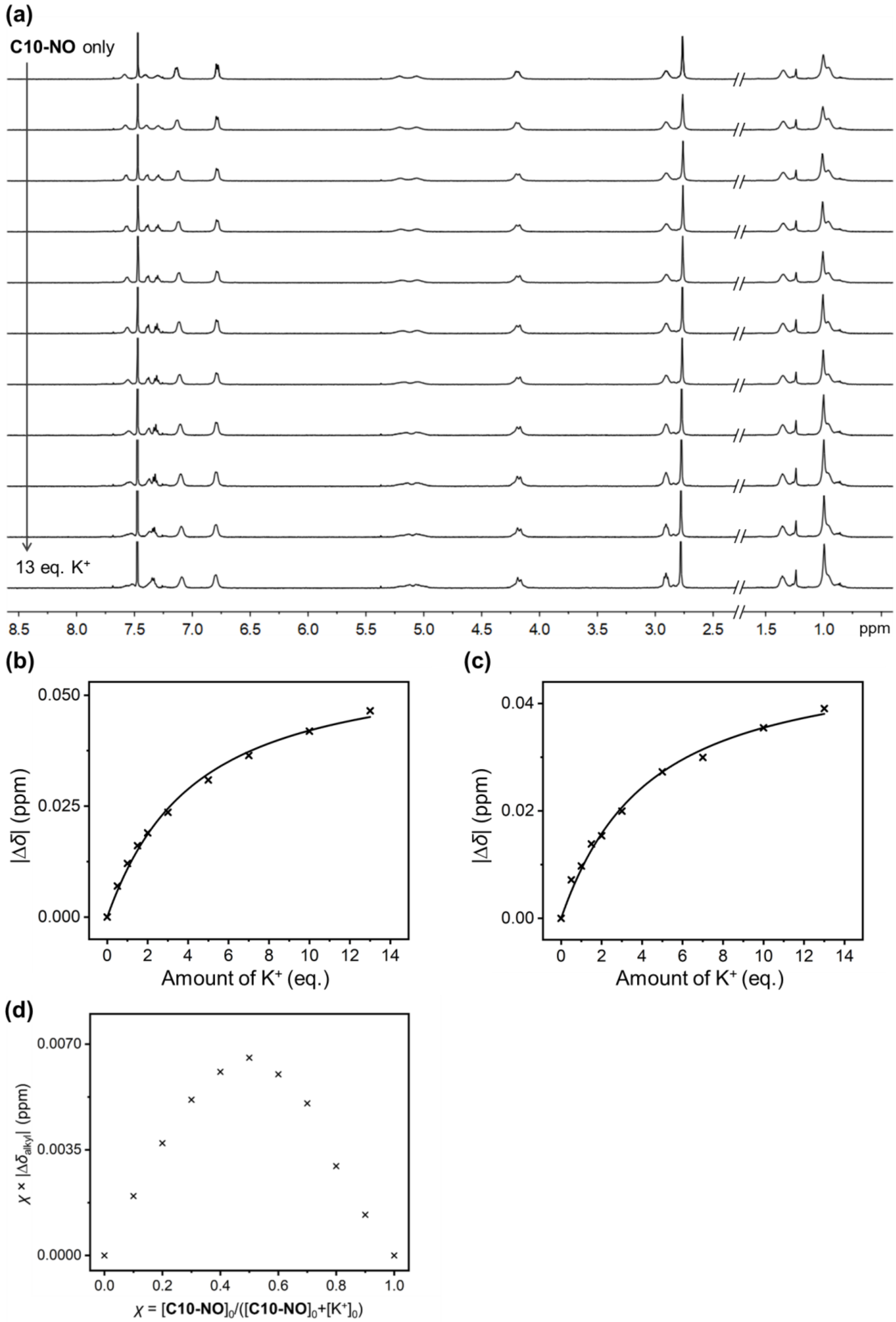

Figure S45. (a) Partial ^{1}H NMR spectra (500 MHz, 298 K, $CD_3CN/CDCl_3$ (1:1, *v*/*v*)) of **C10-NO** in the presence of 0 eq. (top), 0.2 eq., 0.5 eq., 1 eq., 1.5 eq., 2 eq., 3 eq., 5 eq., 7 eq., 10 eq. and 13 eq. (bottom) of KOTf. Binding isotherms obtained by monitoring chemical shift changes of signals initially at (b) 7.29 ppm and (c) 7.13 ppm. Association constant calculated: $K(K^+)$ = 260 (± 13) M^{-1}. (d) Job plot of the K^+ binding to **C10-NO** in $CD_3CN/CDCl_3$ (1:1, *v*/*v*).

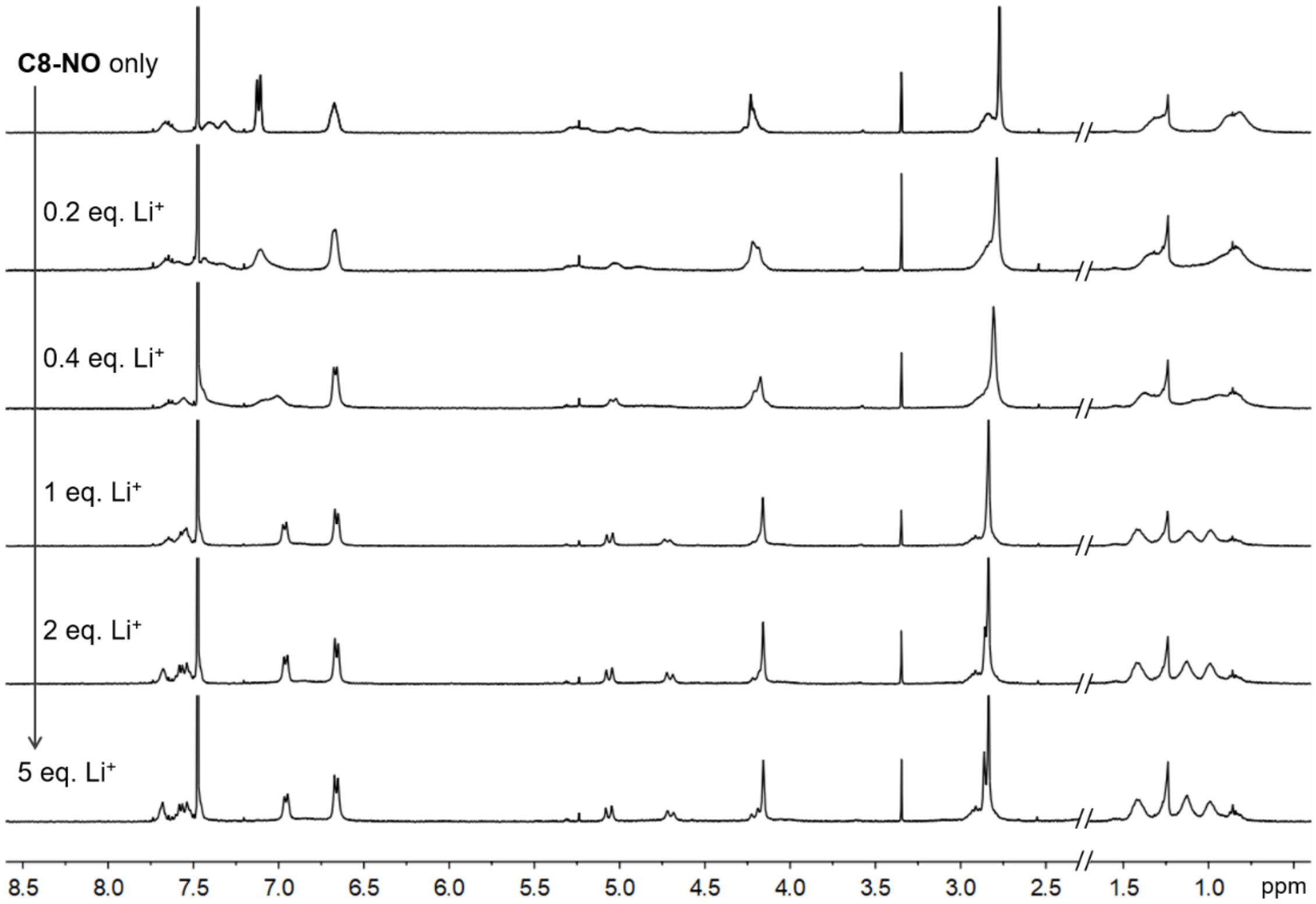

Figure S46. Partial ^{1}H NMR spectra (400 MHz, 298 K, $CD_3CN/CDCl_3$ (1:1, *v*/*v*)) of 1 mM solution of **C8-NO** in the presence of 0 eq. (top), 0.2 eq., 0.4 eq., 1 eq., 2 eq. and 5 eq. (bottom) of LiOTf.

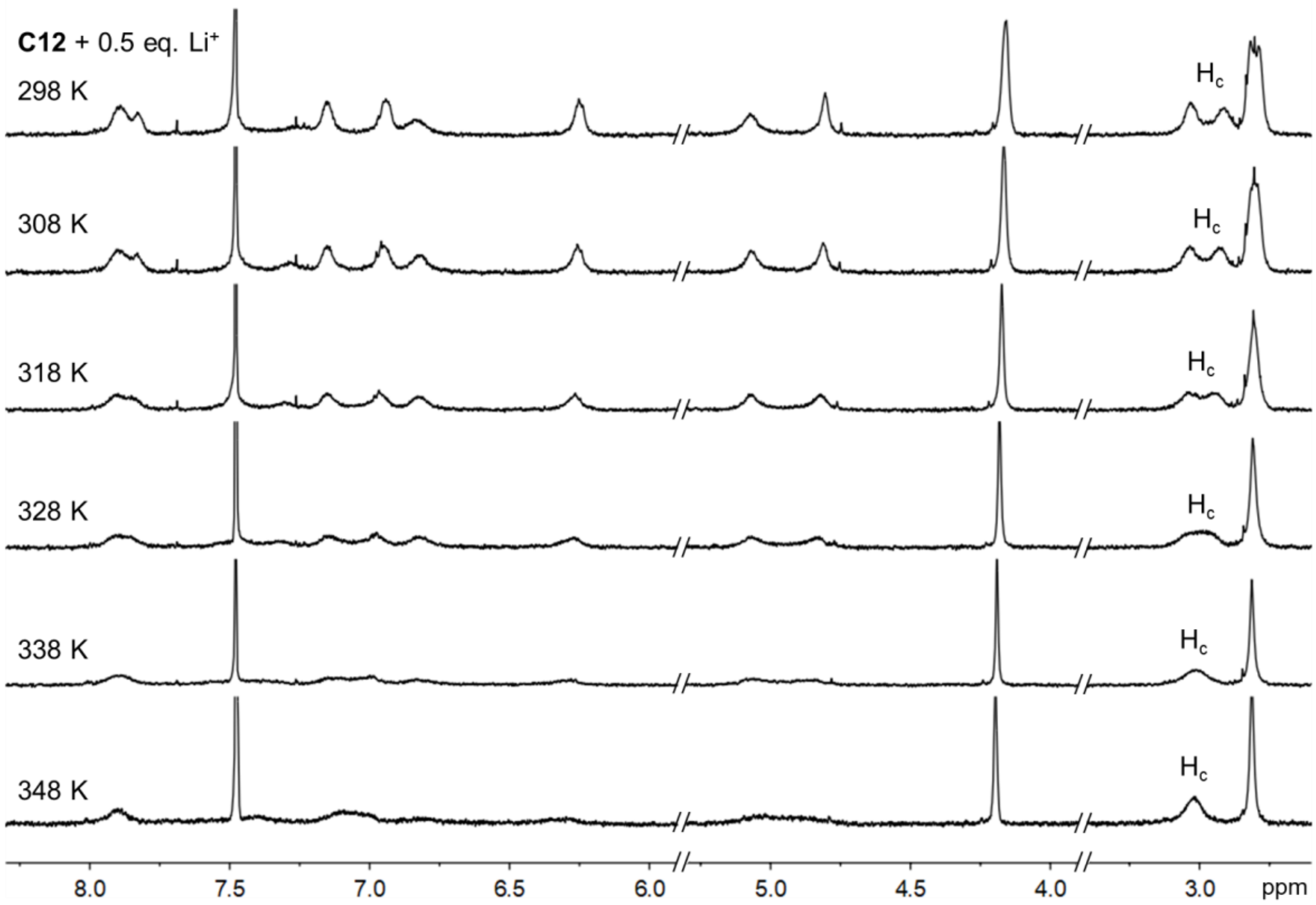

Figure S47. Partial ^{1}H NMR spectra (500 MHz, $CD_3CN/CDCl_3$ (1:1, *v*/*v*)) of a mixture of **C12** and 0.5 eq. of LiOTf obtained at temperatures from 298 K to 348 K. Coalescence temperature for signal H_c was found at 328 K.

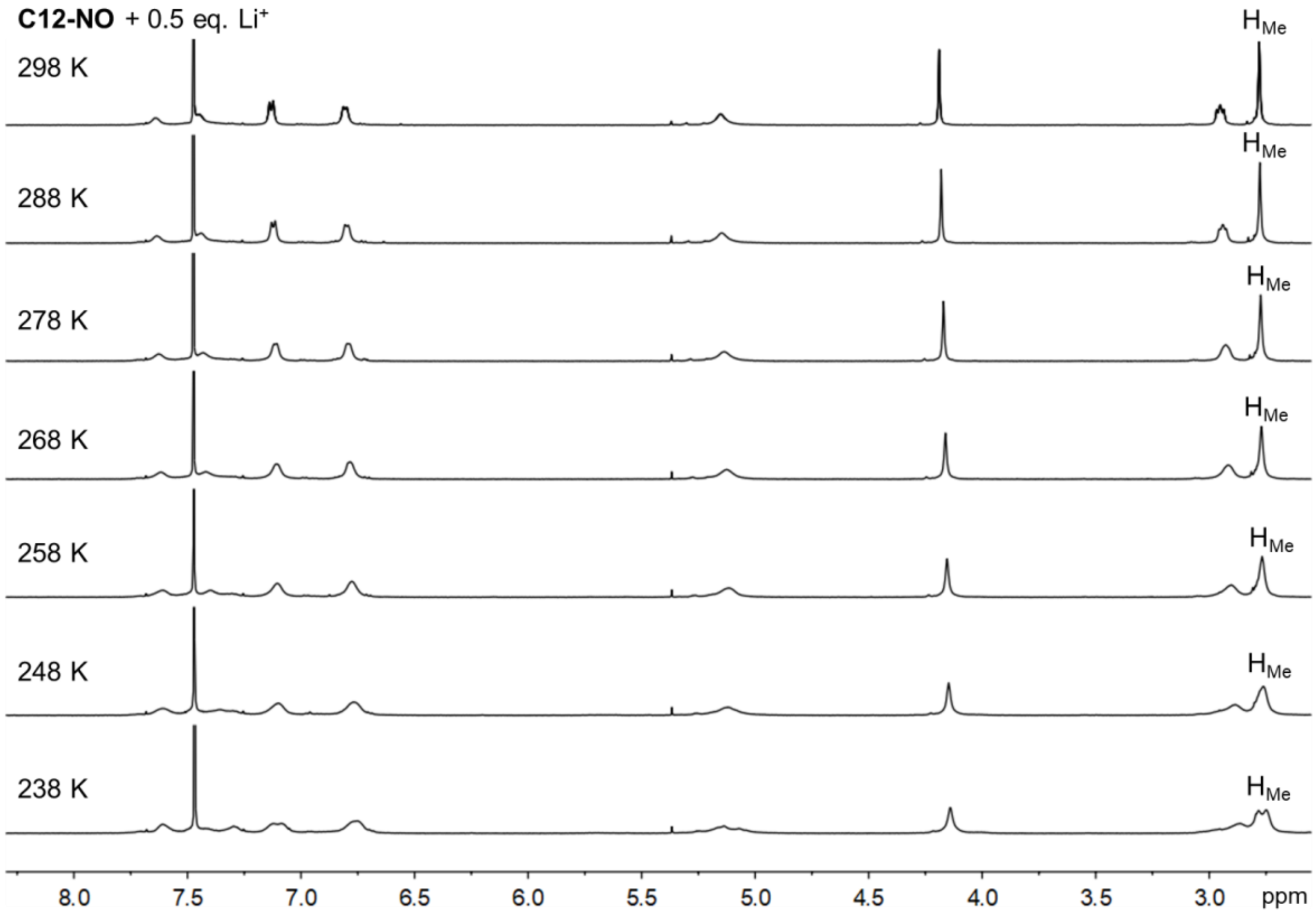

Figure S48. Partial ^{1}H NMR spectra (500 MHz, $CD_3CN/CDCl_3$ (1:1, *v*/*v*)) of a mixture of **C12-NO** and 0.5 eq. of LiOTf obtained at temperatures from 238 K to 298 K. Coalescence temperature for signal H_{Me} and H_{Ar} was found at 248 K.

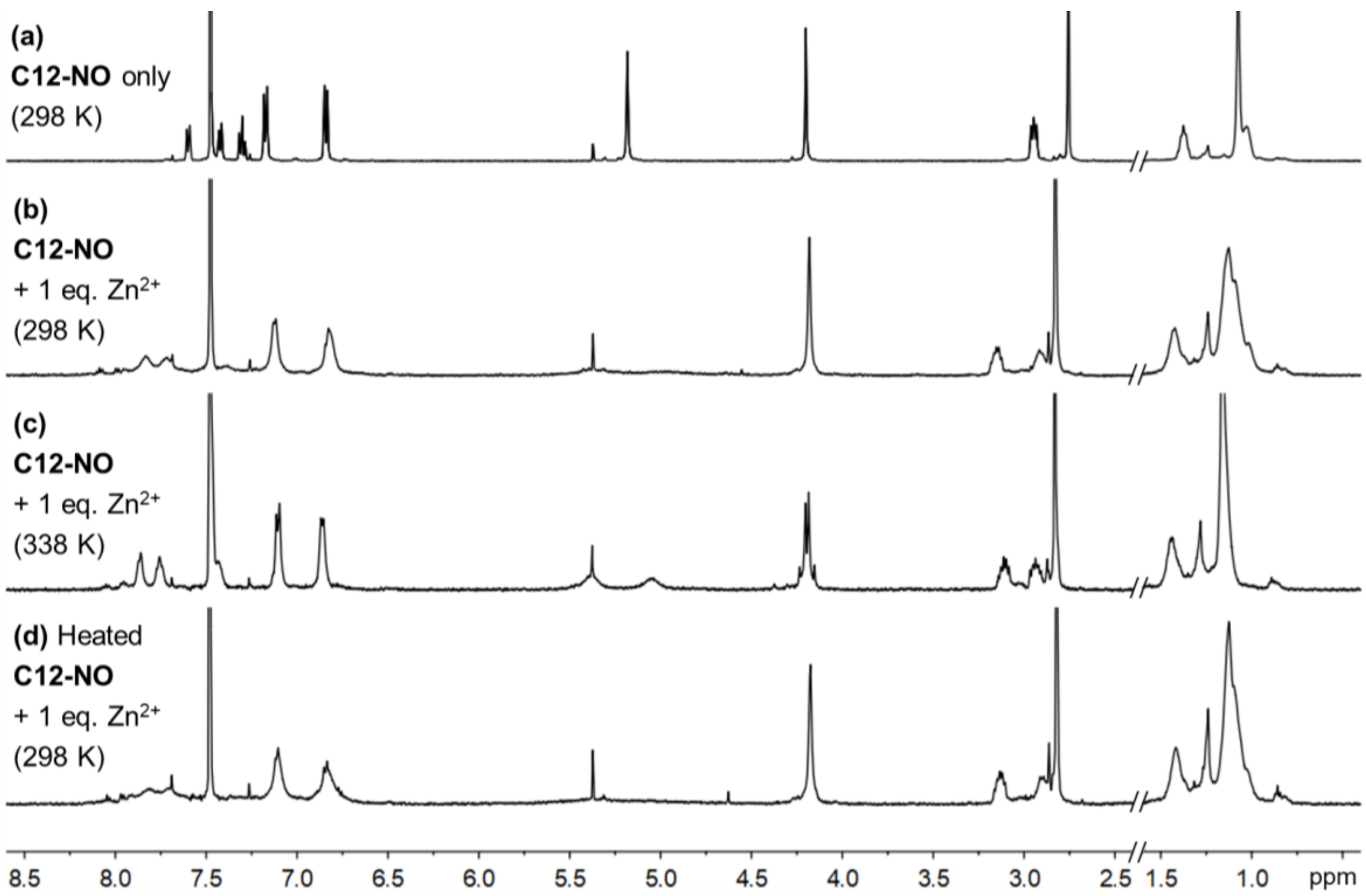

Figure S49. Partial ^{1}H NMR spectra (500 MHz, $CD_3CN/CDCl_3$ (1:1, *v*/*v*)) of (a) **C12-NO** (spectrum obtained at 298 K), (b) a mixture of **C12-NO** and 1 eq. of $Zn(ClO_4)_2$ (spectrum obtained at 298 K), (c) a mixture of **C12-NO** and 1 eq. of $Zn(ClO_4)_2$ (spectrum obtained at 338 K) and (d) a mixture of **C12-NO** and 1 eq. of $Zn(ClO_4)_2$ that was heated at 338 K for 8 hours and cooled down to room temperature (spectrum obtained at 298 K).

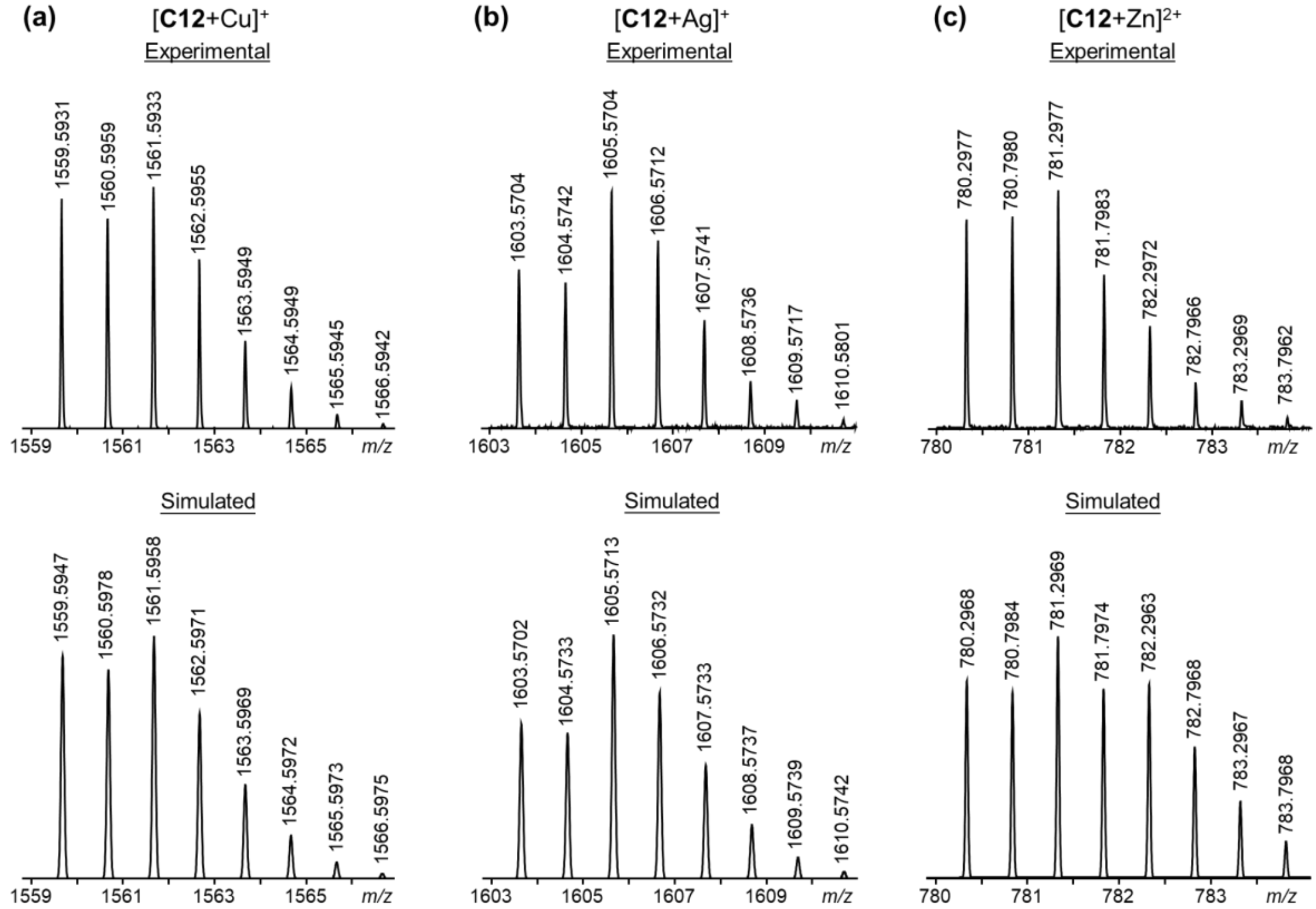

Figure S50. Isotopic patterns of the HR-ESI-MS (+ve) signals (above: experimental; below: simulated) of (a) [**C12**+Cu]$^{+}$, (b) [**C12**+Ag]$^{+}$ and (c) [**C12**+Zn]$^{2+}$.

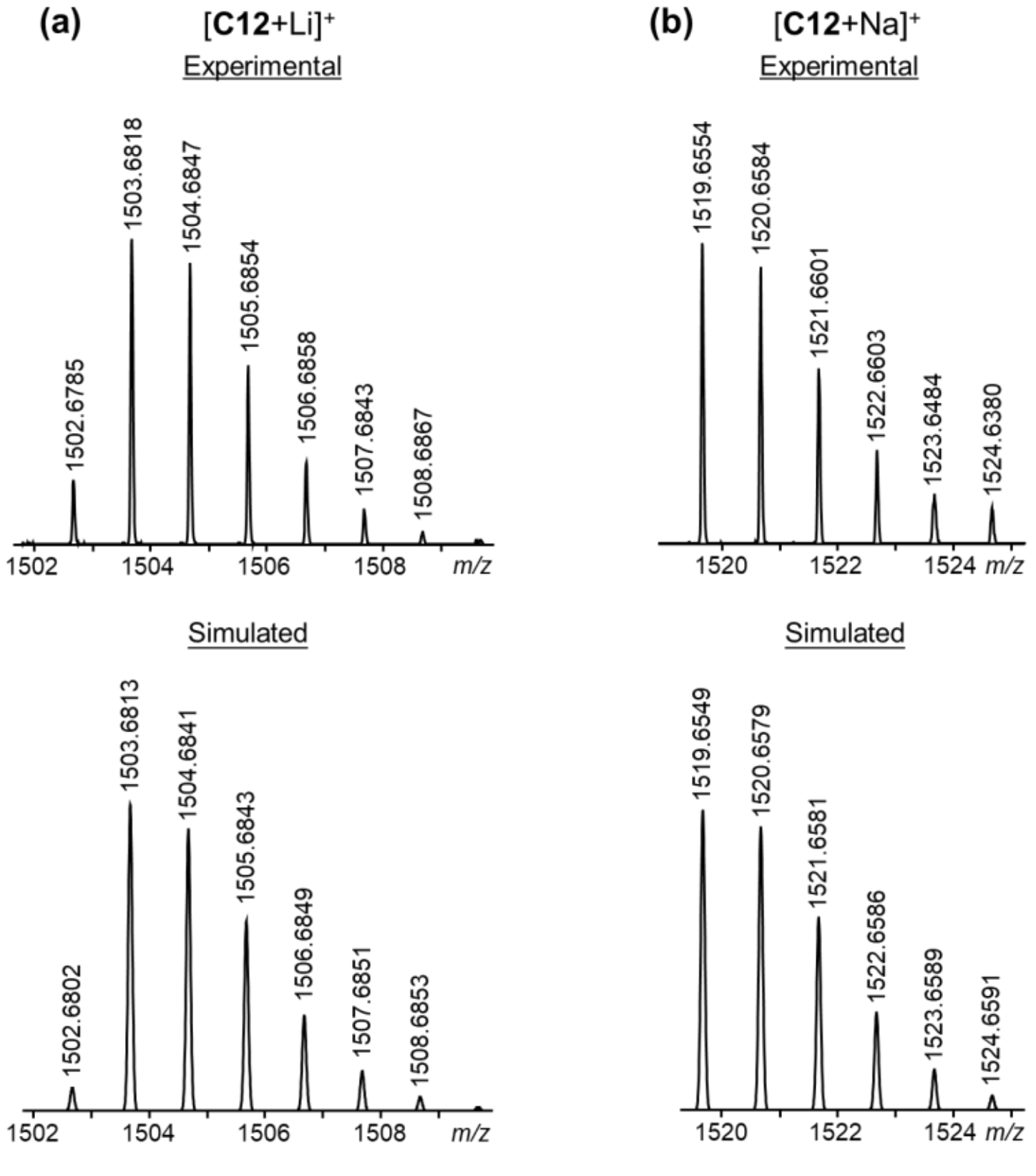

Figure S51. Isotopic patterns of the HR-ESI-MS (+ve) signals (above: experimental; below: simulated) of (a) [**C12**+Li]$^{+}$ and (b) [**C12**+Na]$^{+}$.

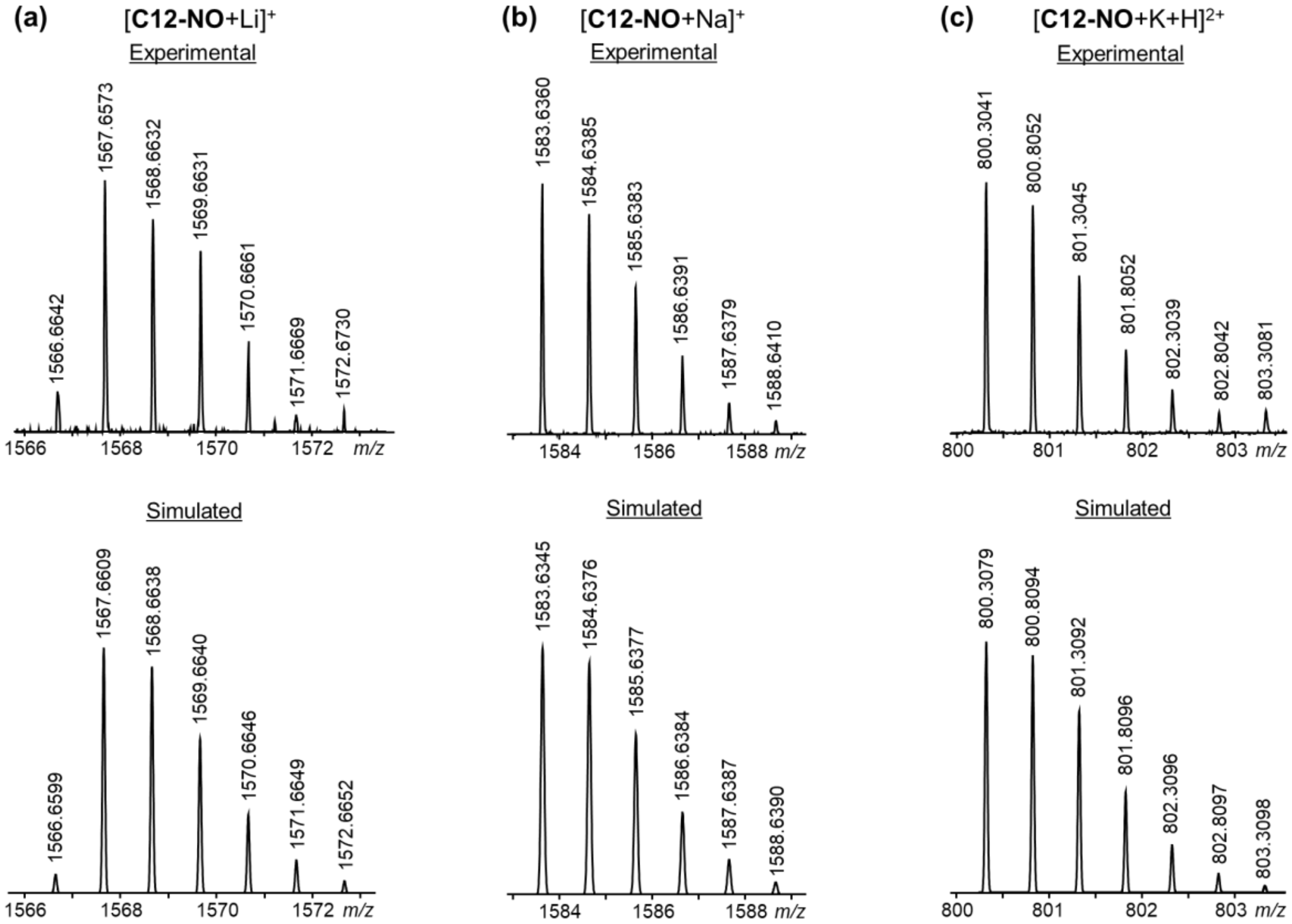

Figure S52. Isotopic patterns of the HR-ESI-MS (+ve) signals (above: experimental; below: simulated) of (a) [**C12-NO**+Li]$^{+}$, (b) [**C12-NO**+Na]$^{+}$ and (c) [**C12-NO**+K+H]$^{2+}$.

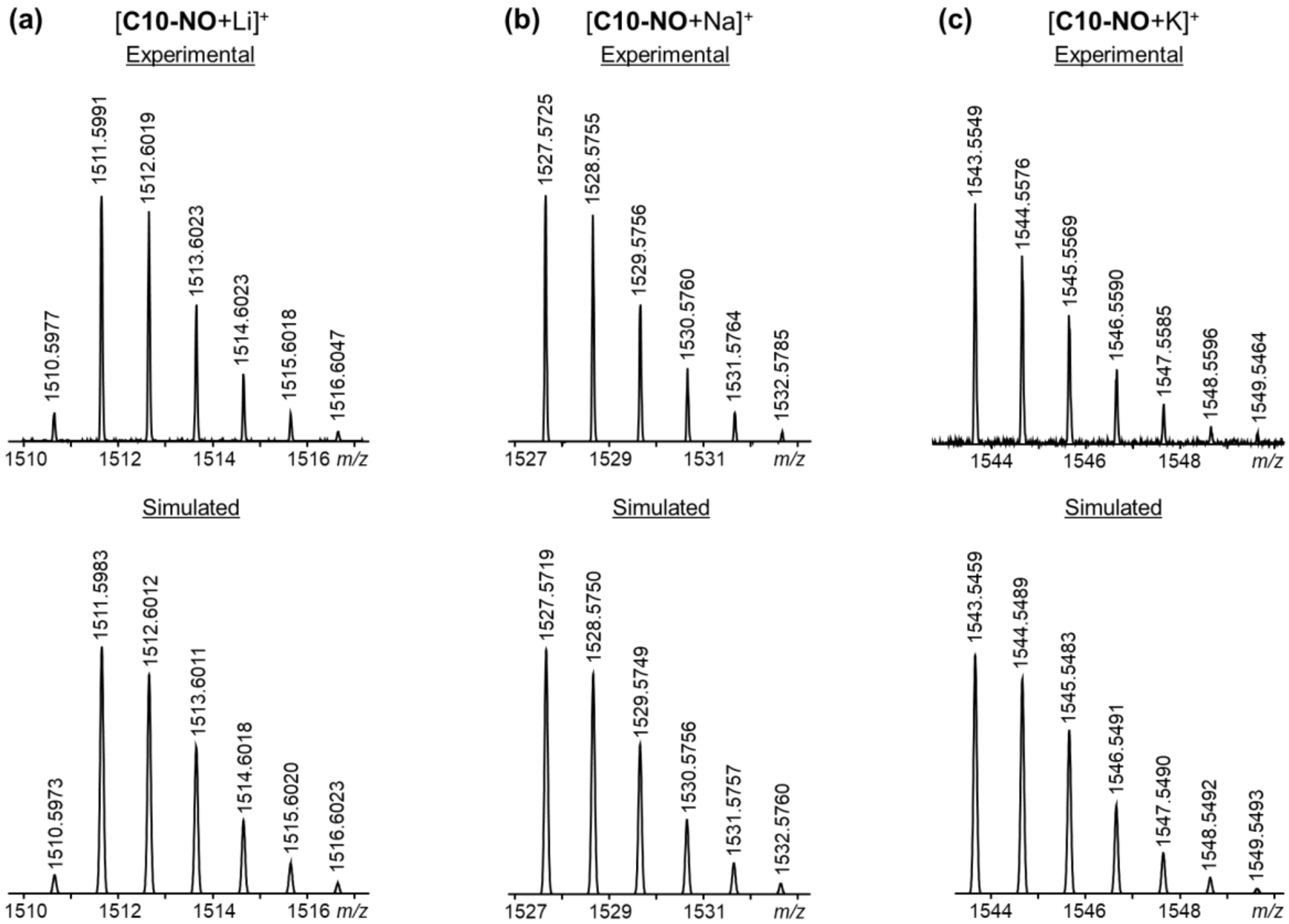

Figure S53. Isotopic patterns of the HR-ESI-MS (+ve) signals (above: experimental; below: simulated) of (a) [**C10-NO**+Li]$^{+}$, (b) [**C10-NO**+Na]$^{+}$ and (c) [**C10-NO**+K]$^{+}$.

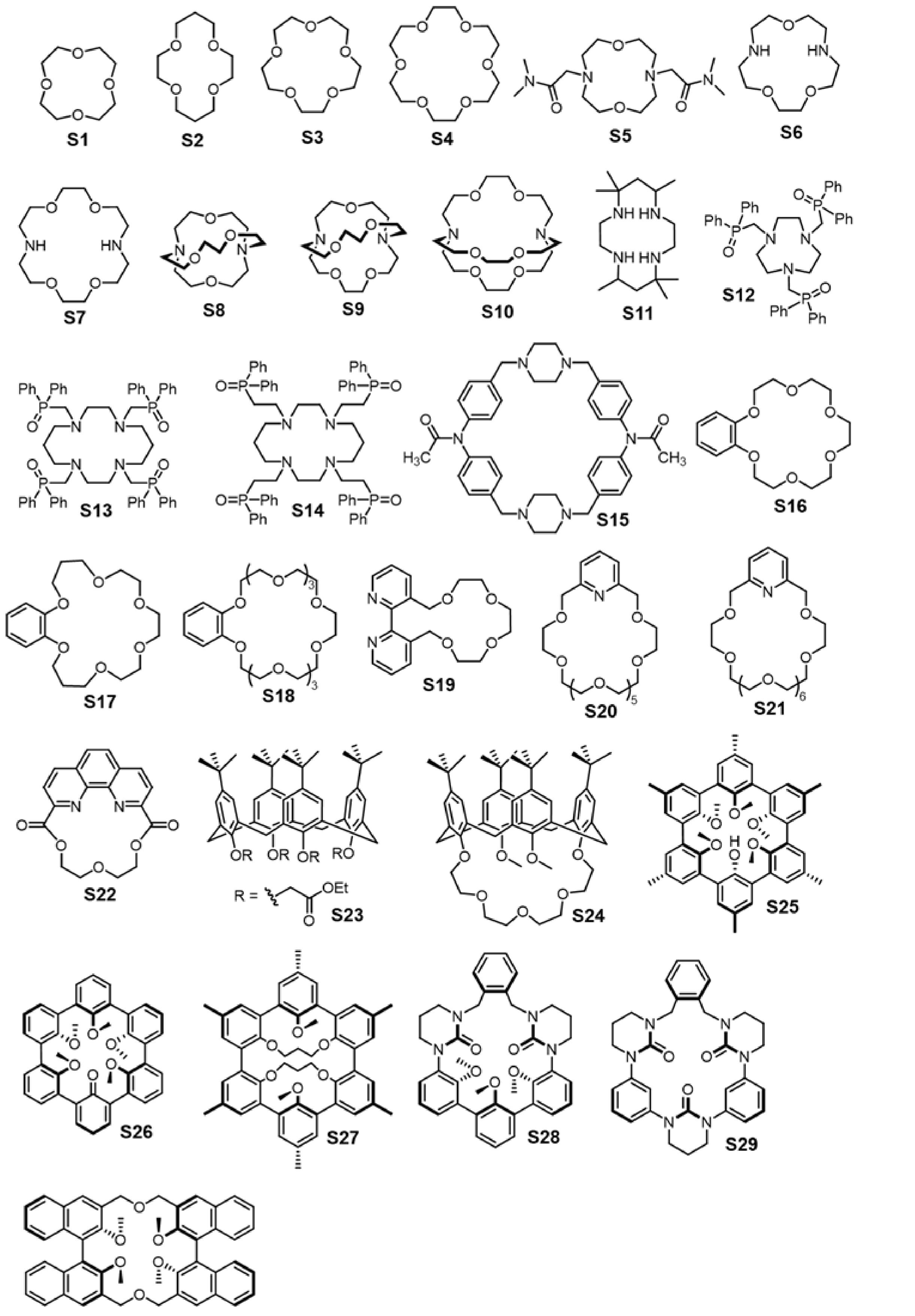

Figure S54. Examples of reported macrocyclic hosts for alkali metal cation binding.

Table S2. Reported binding data of the above hosts at 298 K (n.d. = not determined).

Host	log $K(Li^+)$	log $K(Na^+)$	log $K(K^+)$	$K(Li^+)/K(Na^+)$	$K(Li^+)/K(K^+)$	Ref.
**C12-NO**[a]	5.7	3.7	2.3	88	2.1×10^3	this work
**C10-NO**[a]	6.0	4.1	2.4	79	4.2×10^3	this work
**S1**[b]	2.9	3.6	2.2	0.20	5.0	[7]
**S2**[c]	2.3	1.6	n.d.	5.0	n.d.	[8]
**S3**[d]	3.4	4.3	4.3	0.14	0.14	[9]
**S4**[d]	2.4	4.5	5.9	8.9×10^{-3}	3.3×10^{-4}	[9]
**S5**[c]	5.4	4.7	3.8	4.6	34	[10]
**S6**[d]	3.1	3.4	2.6	0.60	3.3	[9]
**S7**[d]	1.5	3.6	3.8	8.1×10^{-3}	4.7×10^{-3}	[9]
**S8**[c]	8.0	6.1	2.3	79	5.0×10^5	[11]
**S9**[c]	5.4	9.6	8.5	5.4×10^{-5}	6.9×10^{-4}	[11]
**S10**[c]	2.6	8.0	10.4	4.0×10^{-6}	1.6×10^{-8}	[11]
**S11**[e]	3.8	3.6	2.3	1.6	32	[12]
**S12**[e]	5.6	4.5	3.2	13	2.5×10^2	[13]
**S13**[e]	4.7	3.8	3.0	7.9	50	[14]
**S14**[e]	6.2	5.9	4.9	2.0	20	[14]
**S15**[f]	8.1	6.6	6.7	32	25	[15]
**S16**[g]	5.8	5.7	7.2	1.3	0.040	[16]
**S17**[g]	5.5	3.8	5.3	50	1.6	[16]
**S18**[g]	5.5	5.0	6.6	3.2	0.079	[16]
**S19**[g]	4.3	3.6	3.8	5.0	3.2	[17]
**S20**[c]	2.1	1.7	3.2	2.5	0.079	[18]
**S21**[c]	2.6	1.4	3.0	16	0.40	[18]
**S22**[g]	6.3	6.2	5.0	1.3	20	[19]
**S23**[h]	6.4	5.8	4.5	4.0	79	[20]
**S24**[f]	4.7	4.6	5.2	1.3	0.32	[21]
**S25**[f]	8.3	6.9	4.4	25	7.9×10^3	[22]
**S26**[f]	9.2	7.2	4.8	1.0×10^2	2.5×10^4	[22]
**S27**[f]	13.3	10.0	n.d.	2.0×10^3	n.d.	[23,24]
**S28**[f]	12.2	11.3	7.9	7.9	2.0×10^4	[25]
**S29**[f]	6.2	5.7	5.2	3.2	11	[26]
**S30**[g]	3.7	3.6	3.7	1.3	1.0	[27]

Solvent: [a] acetonitrile/chloroform (1:1, *v/v*); [b] propylene carbonate; [c] methanol; [d] acetone; [e] THF/chloroform (4:1, *v/v*); [f] water-saturated chloroform; [g] chloroform; [h] acetonitrile.

3. Solid–Liquid Extraction

In solid–liquid extraction experiments, 100:100:100 and 1:100:100 equimolar mixtures of solid LiX, NaX and KX (X^- = Cl^- or OTf^-) were treated with a 1 mol% solution of (relative to NaX or KX) **C12-NO** or **C12** in $CHCl_3$ (1500 μL, 1 mM). The mixtures were stirred at room temperature for 12 hours (rpm = 1000). The mixture was allowed to stand at ambient condition for 30 minutes after the stirring ceased, and the organic phase was collected using a syringe and filtered through a 0.22 μm filter membrane. The filtrate (1000 μL) was placed in a new vial and deionized water (1000 μL) was added. The alkali metal salt was back extracted to the aqueous phase, which was collected and diluted by 40 to 200 folds before the metal content was analyzed by an Agilent 7700x Inductively Coupled Plasma Mass Spectrometry (ICP-MS), with 1550 W forward RF power, 2W reflected RF power, 1.05 L/min carrier gas flow, 10 mm sample depth, spray chamber cooled to 2 °C, and a nebulizer pump speed of 0.1 rps. Control experiments using the chloride salts in the absence of catenane hosts were conducted following the same procedures described above.

Standard solutions of ^{7}Li, ^{23}Na and ^{39}K (1 g/L) were purchased from Supelco. For each ICP-MS measurement, diluted samples of the ^{7}Li, ^{23}Na and ^{39}K standard were freshly prepared at 10 μg/L, 20 μg/L, 40 μg/L, 60 μg/L, 80 μg/L, 100 μg/L and 120 μg/L by serial dilution with deionized water (Figures S55–S60). Limit of detection (LoD) was determined by the 3σ method.[28] Concentration of ^{7}Li, ^{23}Na and ^{39}K in the aqueous samples obtained from the solid–liquid extraction was calculated using the linear relation obtained from the calibration curves. Each sample was spiked with the corresponding metal at 10 μg/L and 20 μg/L, and each measurement was triplicated.

The solid–liquid extraction of **C12-NO** was further studied by ^{1}H NMR analysis. Solid LiCl or solid mixtures of LiCl/NaCl/KCl (100:100:100 or 1:100:100) were respectively treated with a 1 mol% solution of **C12-NO** in $CDCl_3$ (1500 μL, 1 mM), and the resulting samples were sonicated for 30 minutes at room temperature. The organic solution was collected using a syringe and filtered through a 0.22 μm filter membrane. The filtrate (225 μL) was mixed with CD_3CN (225 μL) to give a solution in $CD_3CN/CDCl_3$ (1:1, *v*/*v*) for ^{1}H NMR analysis. For the time-dependent ^{1}H NMR studies to monitor the extraction, a sample of 1 mM **C12-NO** or **C12** in $CDCl_3$ (500 μL) in the presence of solid LiCl, NaCl and KCl (1:1:1, 100 eq. each) was prepared and analyzed at different time intervals.

Table S3. Summary of extraction efficiency (mol_{MX}/mol_{host}) of solid–liquid extractions.

Host (1 mM)	Host:LiX:NaX:KX (*mol/mol/mol/mol*)	Salts	Extraction efficiency
**C12-NO**	1:100:100:100	LiCl	0.72 ± 0.02
		NaCl	< LoD
		KCl	< LoD
**C12-NO**	1:1:100:100	LiCl	0.44 ± 0.01
		NaCl	< LoD
		KCl	< LoD
**C12**	1:100:100:100	LiCl	0.020 ± 0.001
		NaCl	< LoD
		KCl	< LoD
**C12**	1:1:100:100	LiCl	< LoD
		NaCl	0.010 ± 0.001
		KCl	< LoD
**C12-NO**	1:100:100:100	LiOTf	0.770 ± 0.003
		NaOTf	< LoD
		KOTf	< LoD
**C12-NO**	1:1:100:100	LiOTf	0.480 ± 0.001
		NaOTf	< LoD
		KOTf	< LoD
**C12**	1:100:100:100	LiOTf	0.36 ± 0.01
		NaOTf	< LoD
		KOTf	< LoD
**C12**	1:1:100:100	LiOTf	0.23 ± 0.01
		NaOTf	0.003 ± 0.003
		KOTf	< LoD
N/A	0:100:100:100	LiCl	< LoD
		NaCl	< LoD
		KCl	< LoD
N/A	0:1:100:100	LiCl	< LoD
		NaCl	< LoD
		KCl	< LoD

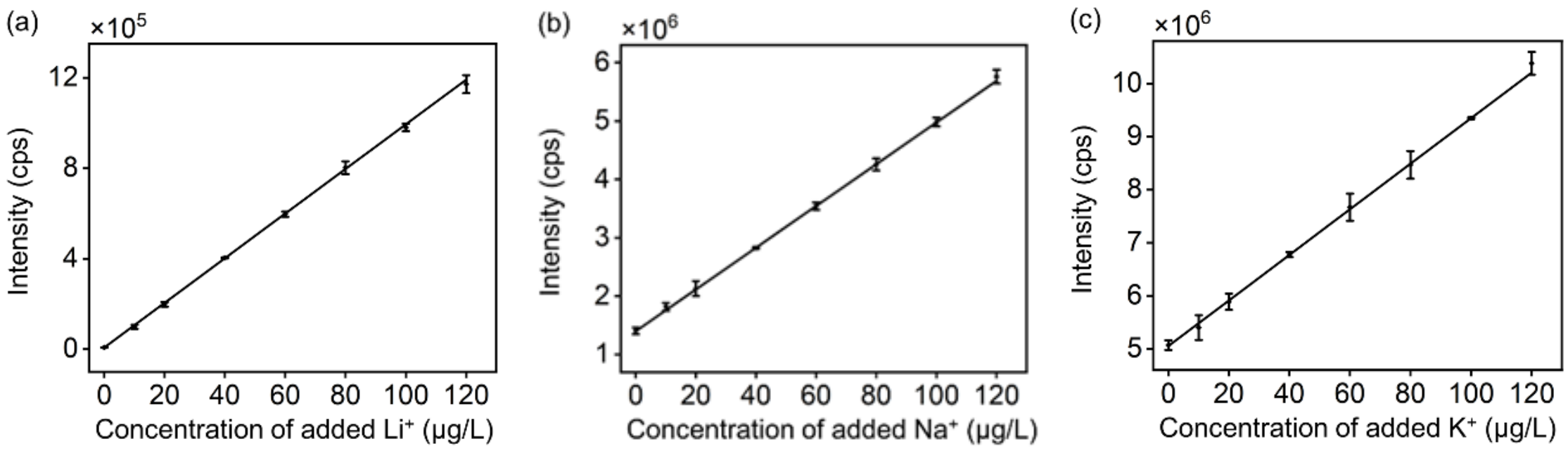

Figure S55. Calibration curves for the ICP-MS measurement of the solid–liquid extraction using **C12-NO** (1 mM in $CHCl_3$) with a 100:100:100 equimolar mixture of solid LiCl, NaCl and KCl. (a) $^{7}Li^{+}$ calibration curve, $y = 9870x + 7700$ ($R^2 = 0.9999$), LoD = 0.0455 μg/L. (b) $^{23}Na^{+}$ calibration curve, $y = 35700x + 1400000$ ($R^2 = 0.9995$), LoD = 0.981 μg/L. (c) $^{39}K^{+}$ calibration curve, $y = 42900x + 5060000$ ($R^2 = 0.9998$), LoD = 5.41 μg/L.

Table S4. Summary of results involving the solid–liquid extraction of a 100:100:100 equimolar mixture of solid LiCl, NaCl and KCl using 1 mol% **C12-NO** (1 mM in $CHCl_3$). **C12-NO**:LiCl:NaCl:KCl = 1:100:100:100 (*mol/mol/mol/mol*)

Cation	Intensity ($\times10^5$ cps)	Measured concentration (μg/L)	Dilution factor	Calculated concentration (μg/L)
$^{7}Li^{+}$	2.55 ± 0.06	25.1 ± 0.6	200	5020 ± 120
$^{23}Na^{+}$	14.3 ± 0.6	< LoD	200	Not detected
$^{39}K^{+}$	51.2 ± 0.6	< LoD	200	Not detected

Table S5. Summary of results measured after spiking the samples with the corresponding metal solutions at 10 μg/L (1st spike) and 20 μg/L (2nd spike). **C12-NO**:LiCl:NaCl:KCl = 1:100:100:100 (*mol/mol/mol/mol*)

Cation	Intensity of 1st spike ($\times10^5$ cps)	Concentration of 1st spike (recovery) (μg/L)	Intensity of 2nd spike ($\times10^5$ cps)	Concentration of 2nd spike (recovery) (μg/L)
$^{7}Li^{+}$	3.48 ± 0.11	34.5 ± 0.1 (98%)	4.39 ± 0.06	44.5 ± 0.6 (99%)
$^{23}Na^{+}$	17.6 ± 0.1	10.2 ± 0.3 (102%)	21.2 ± 0.4	20.1 ± 1.1 (101%)
$^{39}K^{+}$	54.6 ± 0.2	9.30 ± 0.6 (93%)	59.4 ± 0.1	20.6 ± 0.3 (103%)

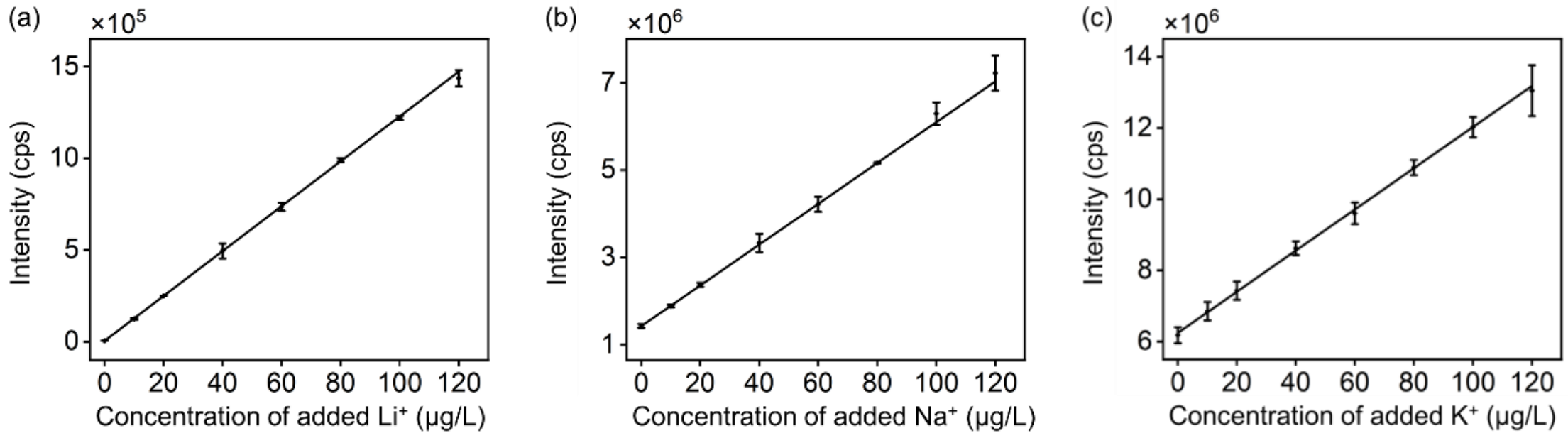

Figure S56. Calibration curves for the ICP-MS measurement of the solid–liquid extraction using **C12** (1 mM in $CHCl_3$) with a 100:100:100 equimolar mixture of solid LiCl, NaCl and KCl. (a) $^{7}Li^{+}$ calibration curve, $y = 12230x + 5260$ ($R^2 = 0.9999$), LoD = 0.0209 µg/L. (b) $^{23}Na^{+}$ calibration curve, $y = 46700x + 1430000$ ($R^2 = 0.9992$), LoD = 2.43 µg/L. (c) $^{39}K^{+}$ calibration curve, $y = 57800x + 6250000$ ($R^2 = 0.9991$), LoD = 8.28 µg/L.

Table S6. Summary of results involving the solid–liquid extraction of a 100:100:100 equimolar mixture of solid LiCl, NaCl and KCl using 1 mol% **C12** (1 mM in $CHCl_3$). **C12**:LiCl:NaCl:KCl = 1:100:100:100 (*mol/mol/mol/mol*)

Cation	Intensity ($\times10^5$ cps)	Measured concentration (µg/L)	Dilution factor	Calculated concentration (µg/L)
$^{7}Li^{+}$	0.53 ± 0.01	3.94 ± 0.08	40	158 ± 3
$^{23}Na^{+}$	14.1 ± 1.4	< LoD	40	Not detected
$^{39}K^{+}$	62.8 ± 1.8	< LoD	40	Not detected

Table S7. Summary of results measured after spiking the samples with the corresponding metal solutions at 10 µg/L (1st spike) and 20 µg/L (2nd spike). **C12**:LiCl:NaCl:KCl = 1:100:100:100 (*mol/mol/mol/mol*)

Cation	Intensity of 1st spike ($\times10^5$ cps)	Concentration of 1st spike (recovery) (µg/L)	Intensity of 2nd spike ($\times10^5$ cps)	Concentration of 2nd spike (recovery) (µg/L)
$^{7}Li^{+}$	1.77 ± 0.09	14.1 ± 0.7 (101%)	2.93 ± 0.07	23.5 ± 0.6 (98%)
$^{23}Na^{+}$	19.0 ± 0.2	10.3 ± 0.4 (103%)	23.4 ± 0.2	19.5 ± 0.4 (98%)
$^{39}K^{+}$	68.7 ± 0.2	10.8 ± 0.4 (108%)	74.6 ± 0.1	21.0 ± 0.2 (105%)

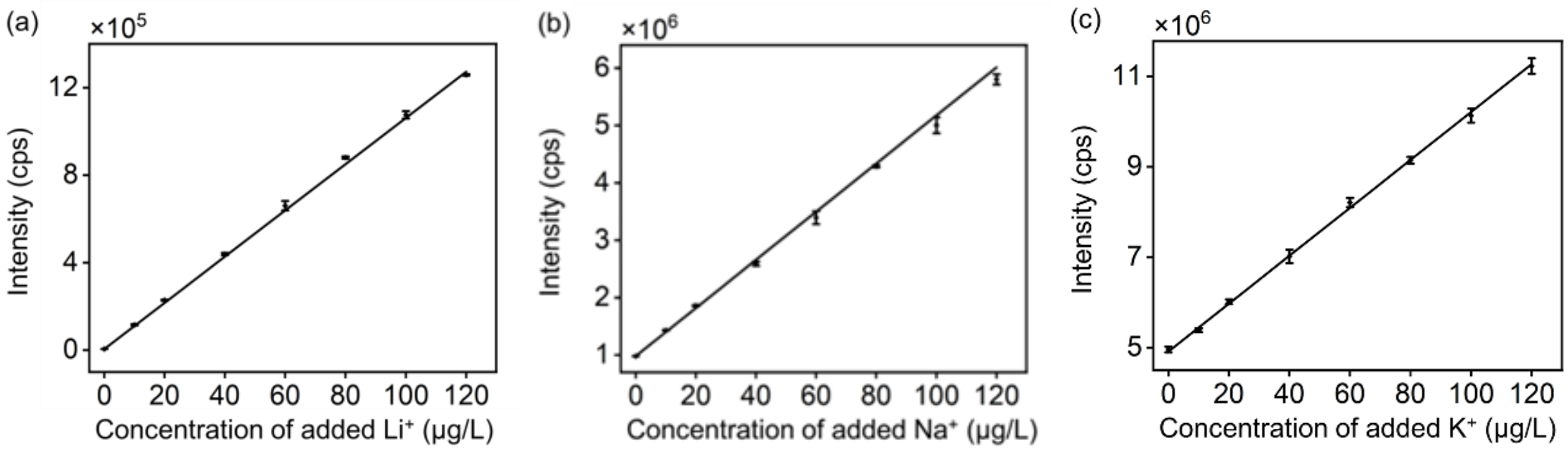

Figure S57. Calibration curves for the ICP-MS measurement of the solid–liquid extraction using **C12-NO** or **C12** (1 mM in $CHCl_3$) with a 1:100:100 equimolar mixture of solid LiCl, NaCl and KCl. (a) $^{7}Li^{+}$ calibration curve, $y = 10560x + 5340$ ($R^2 = 0.9995$), LoD = 0.0443 μg/L. (b) $^{23}Na^{+}$ calibration curve, $y = 41900x + 982000$ ($R^2 = 0.9990$), LoD = 0.178 μg/L. (c) $^{39}K^{+}$ calibration curve, $y = 47700x + 5320000$ ($R^2 = 0.9990$), LoD = 3.90 μg/L.

Table S8. Summary of results involving the solid–liquid extraction of a 1:100:100 equimolar mixture of solid LiCl, NaCl and KCl using 1 mol% **C12-NO** (1 mM in $CHCl_3$). **C12-NO**:LiCl:NaCl:KCl = 1:1:100:100 (*mol/mol/mol/mol*)

Cation	Intensity ($\times 10^5$ cps)	Measured concentration (μg/L)	Dilution factor	Calculated concentration (μg/L)
$^{7}Li^{+}$	1.69 ± 0.01	15.5 ± 0.1	200	3100 ± 20
$^{23}Na^{+}$	9.80 ± 0.04	< LoD	200	Not detected
$^{39}K^{+}$	53.3 ± 0.3	< LoD	200	Not detected

Table S9. Summary of results measured after spiking the samples with the corresponding metal solutions at 10 μg/L (1st spike) and 20 μg/L (2nd spike).
C12-NO:LiCl:NaCl:KCl = 1:1:100:100 (*mol/mol/mol/mol*)

Cation	Intensity of 1st spike ($\times 10^5$ cps)	Concentration of 1st spike (recovery) (μg/L)	Intensity of 2nd spike ($\times 10^5$ cps)	Concentration of 2nd spike (recovery) (μg/L)
$^{7}Li^{+}$	2.86 ± 0.01	26.6 ± 0.3 (104%)	3.86 ± 0.03	36.1 ± 0.2 (102%)
$^{23}Na^{+}$	14.2 ± 0.1	10.5 ± 0.2 (105%)	18.1 ± 0.1	19.8 ± 0.2 (99%)
$^{39}K^{+}$	58.2 ± 0.2	10.4 ± 0.3 (104%)	63.1 ± 0.2	20.8 ± 0.4 (104%)

Table S10. Summary of results involving the solid–liquid extraction of a 1:100:100 equimolar mixture of solid LiCl, NaCl and KCl using 1 mol% **C12** (1 mM in $CHCl_3$). **C12**:LiCl:NaCl:KCl = 1:1:100:100 (*mol/mol/mol/mol*)

Cation	Intensity ($\times 10^5$ cps)	Measured concentration (μg/L)	Dilution factor	Calculated concentration (μg/L)
$^{7}Li^{+}$	0.05	< LoD	40	Not detected
$^{23}Na^{+}$	12.5 ± 0.01	6.45 ± 0.02	40	258 ± 1
$^{39}K^{+}$	53.3 ± 0.2	< LoD	40	Not detected

Table S11. Summary of results measured after spiking the samples with the corresponding metal solutions at 10 μg/L (1st spike) and 20 μg/L (2nd spike).
C12:LiCl:NaCl:KCl = 1:1:100:100 (*mol/mol/mol/mol*)

Cation	Intensity of 1st spike (×10^5 cps)	Concentration of 1st spike (recovery) (μg/L)	Intensity of 2nd spike (×10^5 cps)	Concentration of 2nd spike (recovery) (μg/L)
$^{7}Li^{+}$	1.14 ± 0.04	10.3 ± 0.4 (103%)	2.14 ± 0.07	19.8 ± 0.7 (102%)
$^{23}Na^{+}$	16.4 ± 0.1	15.9 ± 0.2 (97%)	20.6 ± 0.2	25.8 ± 0.6 (98%)
$^{39}K^{+}$	58.2 ± 0.2	10.3 ± 0.4 (103%)	62.7 ± 0.1	20.0 ± 0.1 (100%)

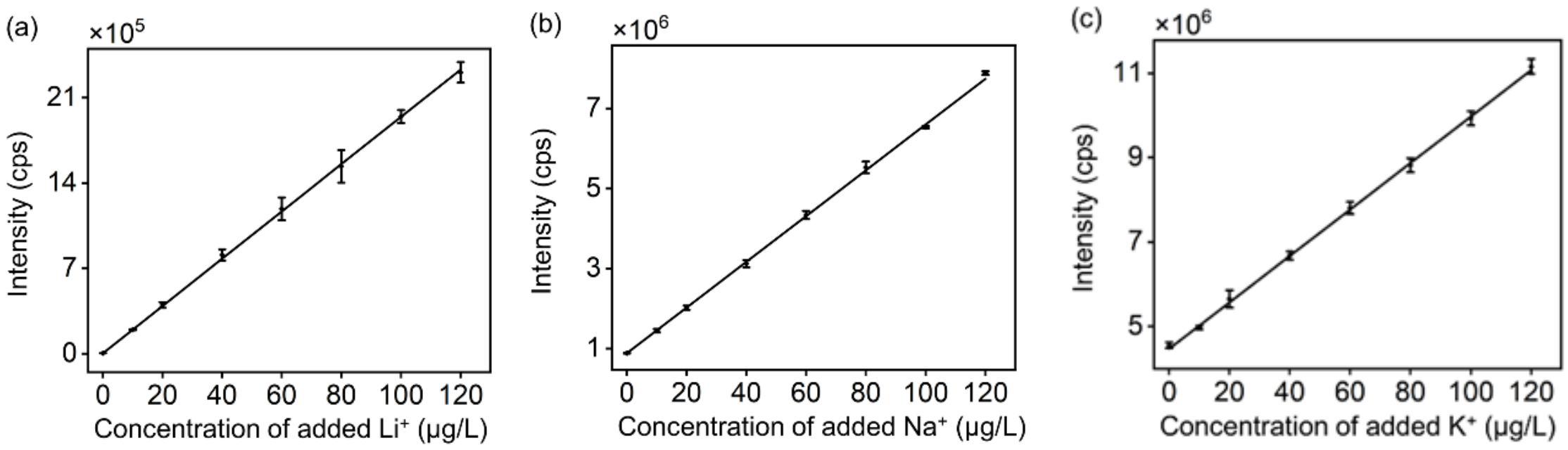

Figure S58. Calibration curves for the ICP-MS measurement of the solid–liquid extraction using **C12-NO** (1 mM in $CHCl_3$) with a 100:100:100 or 1:100:100 equimolar mixture of solid LiOTf, NaOTf and KOTf. (a) $^{7}Li^{+}$ calibration curve, $y = 19380x + 4160$ ($R^2 = 0.9998$), LoD = 0.0164 μg/L. (b) $^{23}Na^{+}$ calibration curve, $y = 54300x + 890000$ ($R^2 = 0.9997$), LoD = 0.438 μg/L. (c) $^{39}K^{+}$ calibration curve, $y = 49500x + 4920000$ ($R^2 = 0.9990$), LoD = 4.52 μg/L.

Table S12. Summary of results involving the solid–liquid extraction of a 100:100:100 equimolar mixture of solid LiOTf, NaOTf and KOTf using 1 mol% **C12-NO** (1 mM in $CHCl_3$). **C12-NO**:LiOTf:NaOTf:KOTf = 1:100:100:100 (*mol/mol/mol/mol*)

Cation	Intensity (×10^5 cps)	Measured concentration (μg/L)	Dilution factor	Calculated concentration (μg/L)
$^{7}Li^{+}$	5.30 ± 0.04	27.1 ± 0.2	200	5420 ± 40
$^{23}Na^{+}$	8.93 ± 0.4	< LoD	200	Not detected
$^{39}K^{+}$	49.1 ± 0.4	< LoD	200	Not detected

Table S13. Summary of results measured after spiking the samples with the corresponding metal solutions at 10 μg/L (1st spike) and 20 μg/L (2nd spike). **C12-NO**:LiOTf:NaOTf:KOTf = 1:100:100:100 (*mol/mol/mol/mol*)

Cation	Intensity of 1st spike (×10^5 cps)	Concentration of 1st spike (recovery) (μg/L)	Intensity of 2nd spike (×10^5 cps)	Concentration of 2nd spike (recovery) (μg/L)
$^{7}Li^{+}$	7.24 ± 0.05	37.2 ± 0.3 (100%)	9.19 ± 0.08	47.2 ± 0.4 (100%)
$^{23}Na^{+}$	14.1 ± 0.4	9.53 ± 0.7 (95%)	19.6 ± 0.2	19.7 ± 0.3 (99%)
$^{39}K^{+}$	54.1 ± 0.3	10.0 ± 0.5 (100%)	59.4 ± 0.4	20.5 ± 0.8 (103%)

Table S14. Summary of results involving the solid–liquid extraction of a 1:100:100 equimolar mixture of solid LiOTf, NaOTf and KOTf using 1 mol% **C12-NO** (1 mM in $CHCl_3$). **C12-NO**:LiOTf:NaOTf:KOTf = 1:1:100:100 (*mol/mol/mol/mol*)

Cation	Intensity ($\times 10^5$ cps)	Measured concentration (μg/L)	Dilution factor	Calculated concentration (μg/L)
$^{7}Li^{+}$	3.30 ± 0.05	16.8 ± 0.3	200	3360 ± 60
$^{23}Na^{+}$	8.99 ± 0.4	< LoD	200	Not detected
$^{39}K^{+}$	49.2 ± 0.2	< LoD	200	Not detected

Table S15. Summary of results measured after spiking the samples with the corresponding metal solutions at 10 μg/L (1st spike) and 20 μg/L (2nd spike). **C12-NO**:LiOTf:NaOTf:KOTf = 1:1:100:100 (*mol/mol/mol/mol*)

Cation	Intensity of 1st spike ($\times 10^5$ cps)	Concentration of 1st spike (recovery) (μg/L)	Intensity of 2nd spike ($\times 10^5$ cps)	Concentration of 2nd spike (recovery) (μg/L)
$^{7}Li^{+}$	5.16 ± 0.1	26.4 ± 0.6 (99%)	7.10 ± 0.08	36.4 ± 0.4 (99%)
$^{23}Na^{+}$	14.1 ± 0.1	9.60 ± 0.3 (96%)	19.5 ± 0.2	19.6 ± 0.3 (98%)
$^{39}K^{+}$	54.3 ± 0.3	10.3 ± 0.7 (103%)	59.2 ± 0.1	20.2 ± 0.3 (101%)

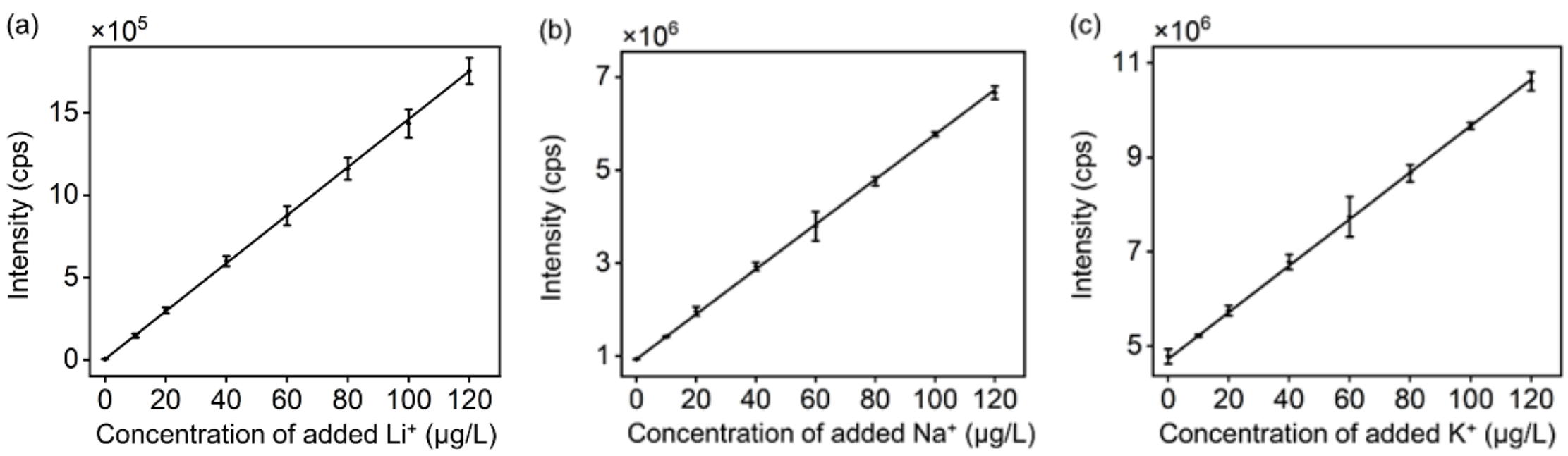

Figure S59. Calibration curves for the ICP-MS measurement of the solid–liquid extraction using **C12** (1 mM in $CHCl_3$) with a 100:100:100 or 1:100:100 equimolar mixture of solid LiOTf, NaOTf and KOTf. (a) $^{7}Li^{+}$ calibration curve, $y = 14570x + 4440$ ($R^2 = 0.9998$), LoD = 0.0170 μg/L. (b) $^{23}Na^{+}$ calibration curve, $y = 44600x + 942000$ ($R^2 = 0.9999$), LoD = 0.178 μg/L. (c) $^{39}K^{+}$ calibration curve, $y = 43100x + 5015000$ ($R^2 = 0.9999$), LoD = 8.86 μg/L.

Table S16. Summary of results involving the solid–liquid extraction of a 100:100:100 equimolar mixture of solid LiOTf, NaOTf and KOTf using 1 mol% **C12** (1 mM in $CHCl_3$). **C12**:LiOTf:NaOTf:KOTf = 1:100:100:100 (*mol/mol/mol/mol*)

Cation	Intensity ($\times 10^5$ cps)	Measured concentration (μg/L)	Dilution factor	Calculated concentration (μg/L)
$^{7}Li^{+}$	1.90 ± 0.03	12.7 ± 0.2	200	2540 ± 40
$^{23}Na^{+}$	9.30 ± 0.02	< LoD	200	Not detected
$^{39}K^{+}$	49.6 ± 0.3	< LoD	200	Not detected

Table S17. Summary of results measured after spiking the samples with the corresponding metal solutions at 10 µg/L (1st spike) and 20 µg/L (2nd spike).
C12:LiOTf:NaOTf:KOTf = 1:100:100:100 (*mol/mol/mol/mol*)

Cation	Intensity of 1st spike (×10^5 cps)	Concentration of 1st spike (recovery) (µg/L)	Intensity of 2nd spike (×10^5 cps)	Concentration of 2nd spike (recovery) (µg/L)
$^{7}Li^{+}$	3.36 ± 0.07	22.8 ± 0.5 (100%)	4.71 ± 0.08	32.1 ± 0.5 (98%)
$^{23}Na^{+}$	13.8 ± 0.2	9.80 ± 0.4 (98%)	18.6 ± 0.4	20.5 ± 0.9 (103%)
$^{39}K^{+}$	55.2 ± 0.5	11.7 ± 1.1 (117%)	58.5 ± 0.5	19.3 ± 1.1 (97%)

Table S18. Summary of results involving the solid–liquid extraction of a 1:100:100 equimolar mixture of solid LiOTf, NaOTf and KOTf using 1 mol% **C12** (1 mM in $CHCl_3$). **C12**:LiOTf:NaOTf:KOTf = 1:1:100:100 (*mol/mol/mol/mol*)

Cation	Intensity (×10^5 cps)	Measured concentration (µg/L)	Dilution factor	Calculated concentration (µg/L)
$^{7}Li^{+}$	1.24 ± 0.03	8.19 ± 0.18	200	1638 ± 36
$^{23}Na^{+}$	9.60 ± 0.1	0.38 ± 0.32	200	76 ± 64
$^{39}K^{+}$	49.0 ± 0.5	< LoD	200	Not detected

Table S19. Summary of results measured after spiking the samples with the corresponding metal solutions at 10 µg/L (1st spike) and 20 µg/L (2nd spike).
C12:LiOTf:NaOTf:KOTf = 1:1:100:100 (*mol/mol/mol/mol*)

Cation	Intensity of 1st spike (×10^5 cps)	Concentration of 1st spike (recovery) (µg/L)	Intensity of 2nd spike (×10^5 cps)	Concentration of 2nd spike (recovery) (µg/L)
$^{7}Li^{+}$	2.72 ± 0.04	18.3 ± 0.3 (101%)	4.16 ± 0.1	28.2 ± 0.7 (100%)
$^{23}Na^{+}$	14.2 ± 0.1	10.7 ± 0.2 (103%)	18.6 ± 0.2	20.6 ± 0.5 (101%)
$^{39}K^{+}$	54.8 ± 0.3	10.8 ± 0.7 (108%)	59.2 ± 0.2	21.0 ± 0.4 (105%)

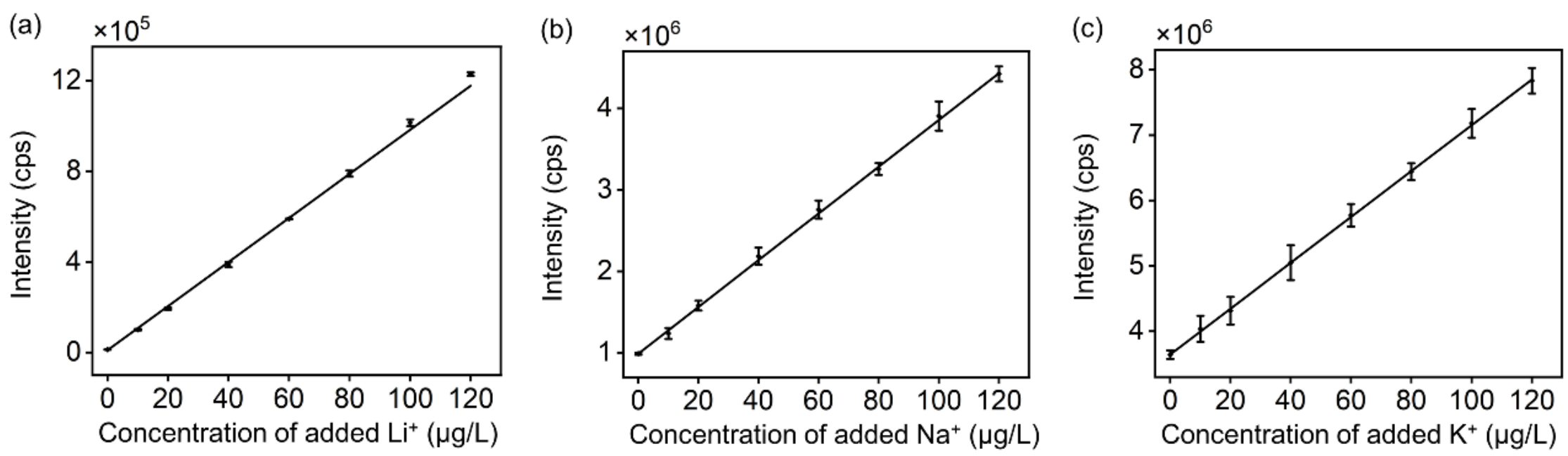

Figure S60. Calibration curves for the ICP-MS measurement of the solid–liquid extraction using $CHCl_3$ (without **C12** or **C12-NO**) with a 100:100:100 or 1:100:100 equimolar mixture of solid LiCl, NaCl and KCl. (a) $^{7}Li^{+}$ calibration curve, $y = 9700x + 11000$ ($R^2 = 0.9992$), LoD = 0.474 µg/L. (b) $^{23}Na^{+}$ calibration curve, $y = 28700x + 988000$ ($R^2 = 0.9996$), LoD = 1.05 µg/L. (c) $^{39}K^{+}$ calibration curve, $y = 35100x + 3645000$ ($R^2 = 0.9998$), LoD = 4.49 µg/L.

Table S20. Summary of results involving the solid–liquid extraction of a 100:100:100 equimolar mixture of solid LiCl, NaCl and KCl without catenane hosts.

Cation	Intensity ($\times 10^5$ cps)	Measured concentration (μg/L)	Dilution factor	Calculated concentration (μg/L)
$^{7}Li^{+}$	0.13 ± 0.01	< LoD	200	Not detected
$^{23}Na^{+}$	8.9 ± 0.3	< LoD	200	Not detected
$^{39}K^{+}$	34.4 ± 0.2	< LoD	200	Not detected

Table S21. Summary of results measured after spiking the samples with the corresponding metal solutions at 10 μg/L (1st spike) and 20 μg/L (2nd spike).
LiCl:NaCl:KCl = 100:100:100 (*mol/mol/mol*), without catenane hosts

Cation	Intensity of 1st spike ($\times 10^5$ cps)	Concentration of 1st spike (recovery) (μg/L)	Intensity of 2nd spike ($\times 10^5$ cps)	Concentration of 2nd spike (recovery) (μg/L)
$^{7}Li^{+}$	1.05 ± 0.02	9.7 ± 0.2 (97%)	1.90 ± 0.03	18.4 ± 0.3 (92%)
$^{23}Na^{+}$	13.0 ± 0.2	10.8 ± 0.7 (108%)	15.9 ± 0.2	20.9 ± 0.7 (105%)
$^{39}K^{+}$	40.2 ± 0.8	10.8 ± 2.4 (108%)	43.4 ± 0.5	19.9 ± 1.4 (100%)

Table S22. Summary of results involving the solid–liquid extraction of a 1:100:100 equimolar mixture of solid LiCl, NaCl and KCl without catenane hosts.

Cation	Intensity ($\times 10^5$ cps)	Measured concentration (μg/L)	Dilution factor	Calculated concentration (μg/L)
$^{7}Li^{+}$	0.13 ± 0.01	< LoD	200	Not detected
$^{23}Na^{+}$	8.8 ± 0.2	< LoD	200	Not detected
$^{39}K^{+}$	34.5 ± 0.2	< LoD	200	Not detected

Table S23. Summary of results measured after spiking the samples with the corresponding metal solutions at 10 μg/L (1st spike) and 20 μg/L (2nd spike). LiCl:NaCl:KCl = 1:100:100 (*mol/mol/mol*), without catenane hosts.

Cation	Intensity of 1st spike ($\times 10^5$ cps)	Concentration of 1st spike (recovery) (μg/L)	Intensity of 2nd spike ($\times 10^5$ cps)	Concentration of 2nd spike (recovery) (μg/L)
$^{7}Li^{+}$	1.06 ± 0.02	9.8 ± 0.2 (98%)	1.87 ± 0.02	18.1 ± 0.2 (91%)
$^{23}Na^{+}$	12.9 ± 0.3	10.4 ± 1.0 (104%)	15.5 ± 0.4	19.7 ± 1.4 (99%)
$^{39}K^{+}$	40.2 ± 0.4	10.6 ± 1.2 (106%)	43.0 ± 0.3	18.8 ± 0.8 (95%)

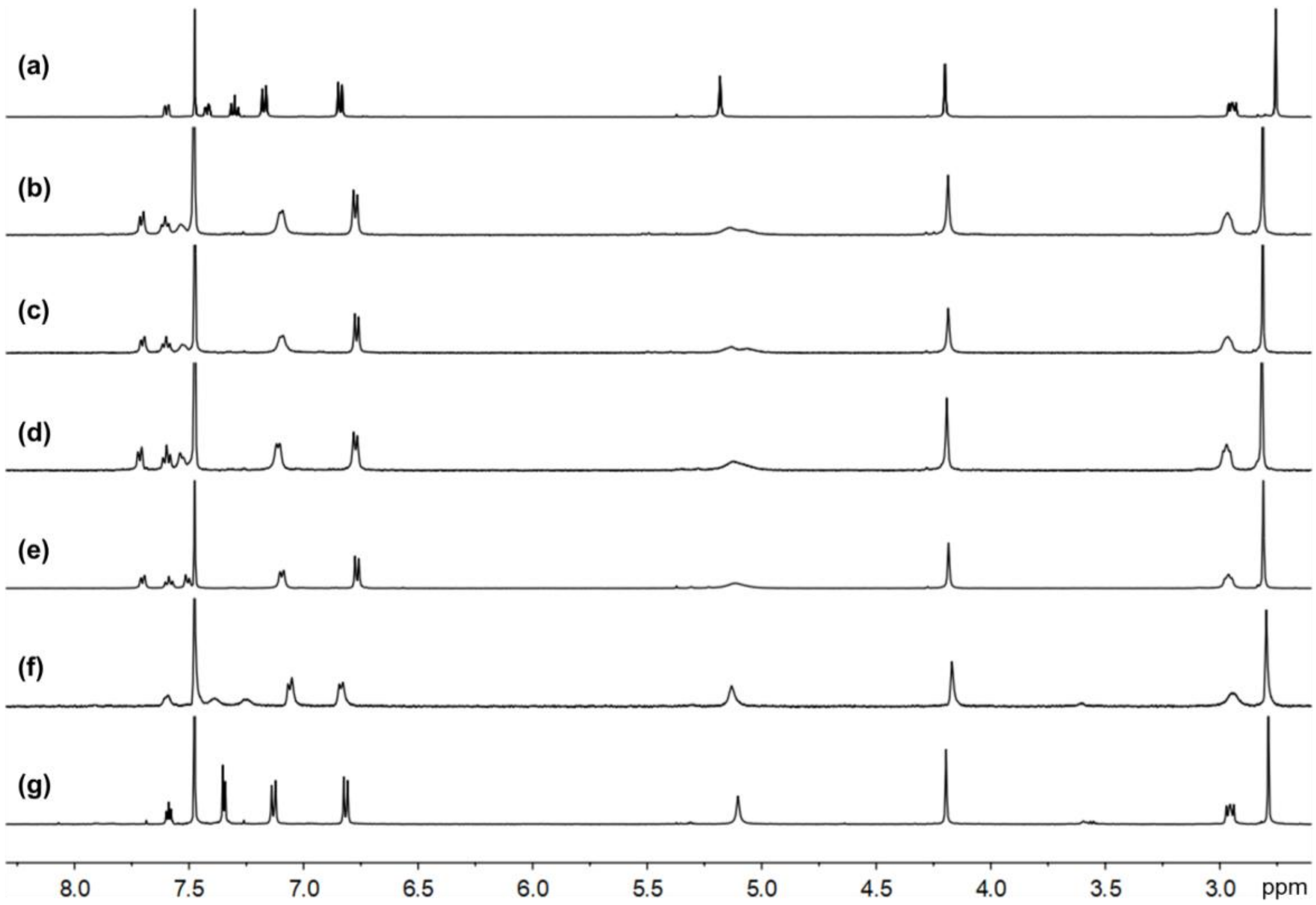

Figure S61. Partial ^{1}H NMR spectra (500 MHz, 298 K, $CD_3CN/CDCl_3$ (1:1, *v*/*v*)) of (a) **C12-NO**; (b) the filtrate obtained after sonicating solid LiCl in 1 mol% **C12-NO** (1 mM); (c) the filtrate obtained after sonicating the 100:100:100 equimolar mixture of solid LiCl, NaCl and KCl in 1 mol% **C12-NO** (1 mM); (d) the filtrate obtained after sonicating the 1:100:100 equimolar mixture of solid LiCl, NaCl and KCl in 1 mol% **C12-NO** (1 mM); (e) **C12-NO** with LiOTf (1 eq.); (f) **C12-NO** with NaOTf (10 eq.); and (g) **C12-NO** with KOTf (10 eq.).

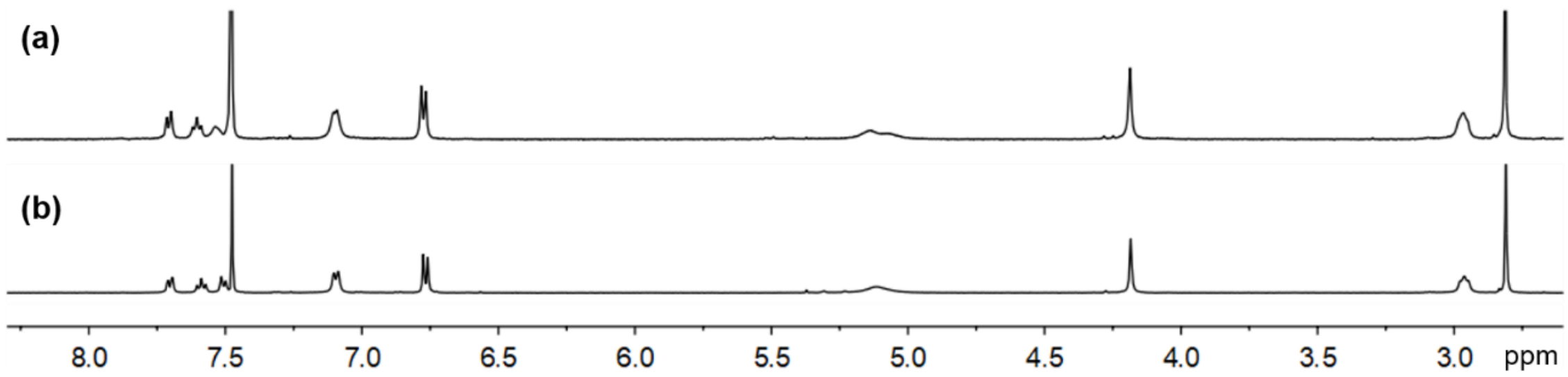

Figure S62. Partial ^{1}H NMR spectra (500 MHz, 298 K, $CD_3CN/CDCl_3$ (1:1, *v*/*v*)) of (a) a filtrate obtained after sonicating solid LiCl in 1 mol% **C12-NO** (1 mM); and (b) **C12-NO** with LiOTf (1 eq.).

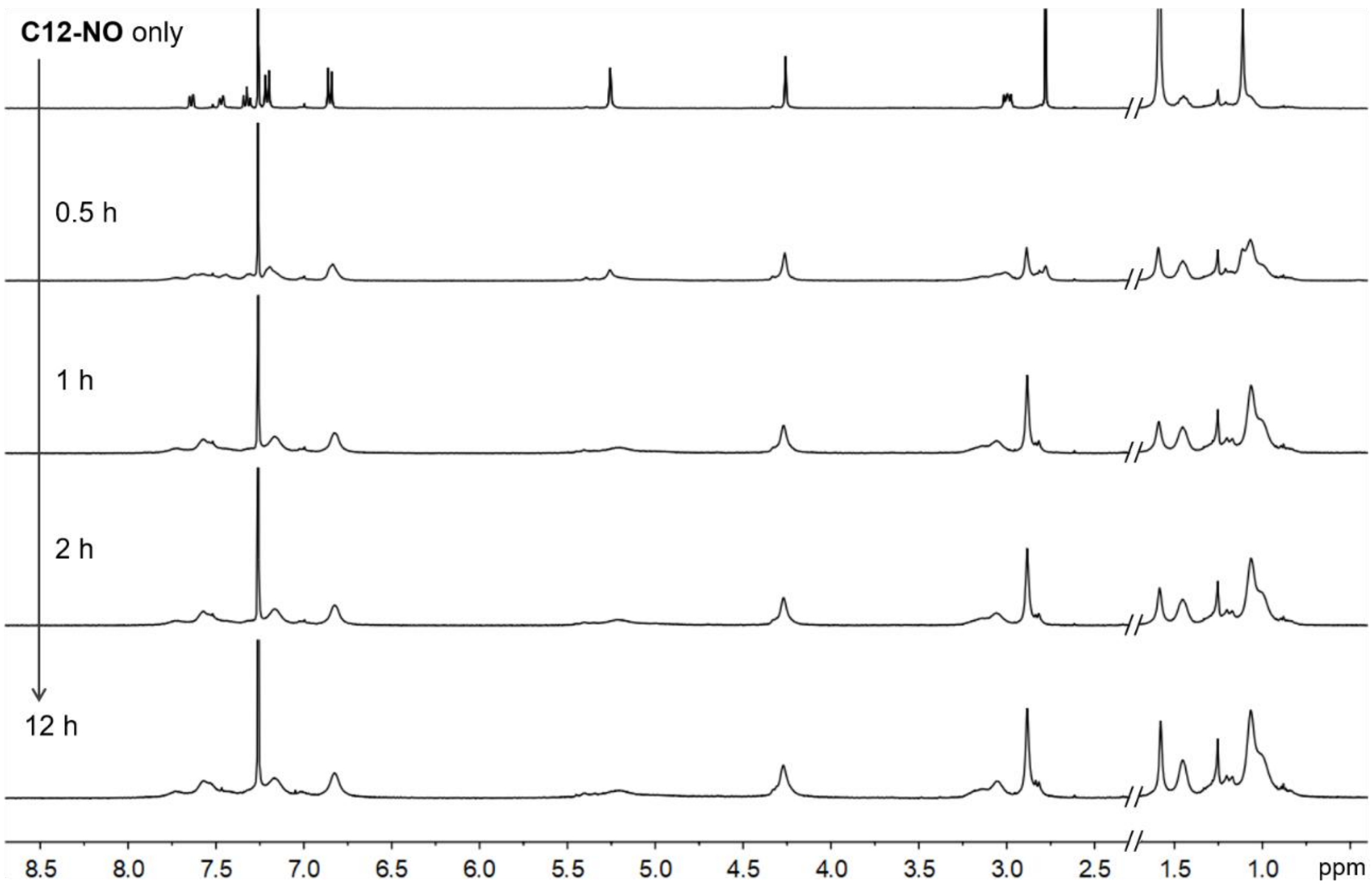

Figure S63. Partial ^{1}H NMR spectra (400 MHz, 298 K, $CDCl_3$) of 1 mM solution of **C12-NO** before (top) and after the addition of a 100:100:100 equimolar mixture of solid LiCl, NaCl and KCl for 0.5 h, 1 h, 2 h and 12 h (bottom) at room temperature. Formation of the Li^+ complex was completed within 1 hour as shown by the shifted bpy signals at ~7.7 ppm and the diastereotopically split signal at ~5.2 and 5.4 ppm.

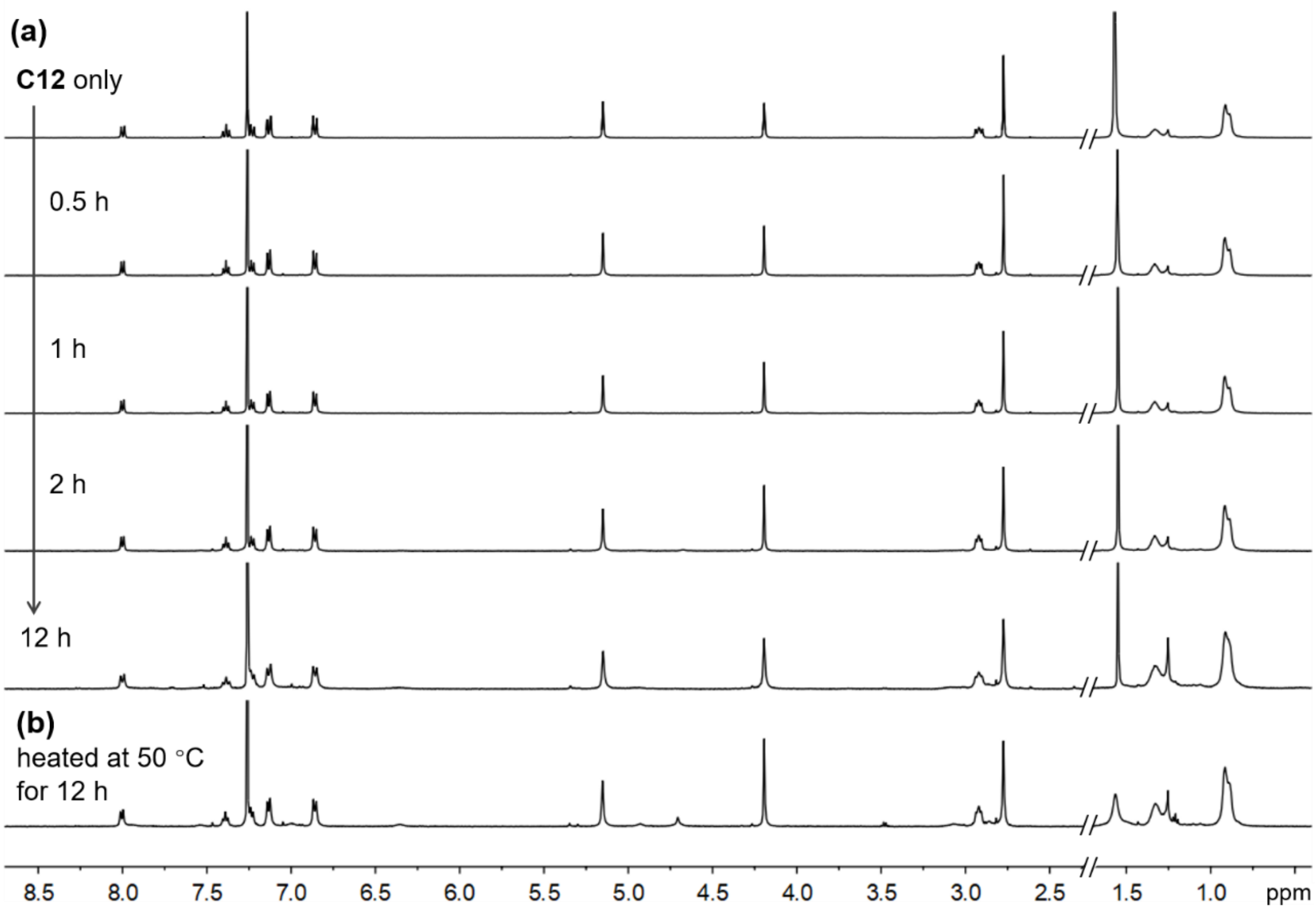

Figure S64. Partial ^{1}H NMR spectra (400 MHz, 298 K, $CDCl_3$) of 1 mM solution of **C12-NO** (a) before (top) and after the addition of a 100:100:100 equimolar mixture of solid LiCl, NaCl and KCl for 0.5 h, 1 h, 2 h and 12 h (bottom) at room temperature or (b) 12 h at 50 °C.

4. Computational Studies on Cation Binding of C12 and C12-NO

The initial configurations and visualization of optimized structures for **C12**, **C12-NO**, [M(**C12**)]$^+$ and [M(**C12-NO**)]$^+$ (M^+ = Li^+, Na^+ and K^+) were generated using the Avogadro software.[29] The resulting empirical optimized configurations were further refined using density functional theory (DFT) calculations performed with Gaussian 16 (revision A.03).[30] Geometry optimizations and single-point energy calculations were carried out using the spin-restricted hybrid density functional B3LYP,[31–33] and the 6-31G (d,p) basis set was employed for all atoms. The solvation effects of CH_3CN (ε = 35.688) and $CHCl_3$ (ε = 4.7113) were taken into account using the conductor-like polarizable continuum model (CPCM).[34,35] During the structural optimization step, a convergence criterion of 10^{-8} for the density matrix was applied. Vibrational analyses were conducted to validate the optimized structures.

Table S24. Structural parameters of [M(**C12**)]$^+$ (M^+ = Li^+, Na^+ and K^+).

	$d_{M\text{-}N}$ (Å)				
	M-N1	M-N2	M-N3	M-N4	avg.
[Li(**C12**)]$^+$	2.104	2.101	2.136	2.097	2.110
[Na(**C12**)]$^+$	2.542	2.419	2.538	2.413	2.478
[K(**C12**)]$^+$	2.885	2.884	2.887	2.882	2.884

Table S25. Structural parameters of [M(**C12-NO**)]$^+$ (M^+ = Li^+, Na^+ and K^+).

	$d_{M\text{-}O}$ (Å)					
	M-O1	M-O2	M-O3	M-O4	M-O5	avg.
[Li(**C12-NO**)]$^+$	1.932	1.920	1.948	1.907		1.927
[Na(**C12-NO**)]$^+$	2.284	2.285	2.345	2.274	2.609	2.359
[K(**C12-NO**)]$^+$	2.635	2.723	2.691	2.639	2.925	2.723

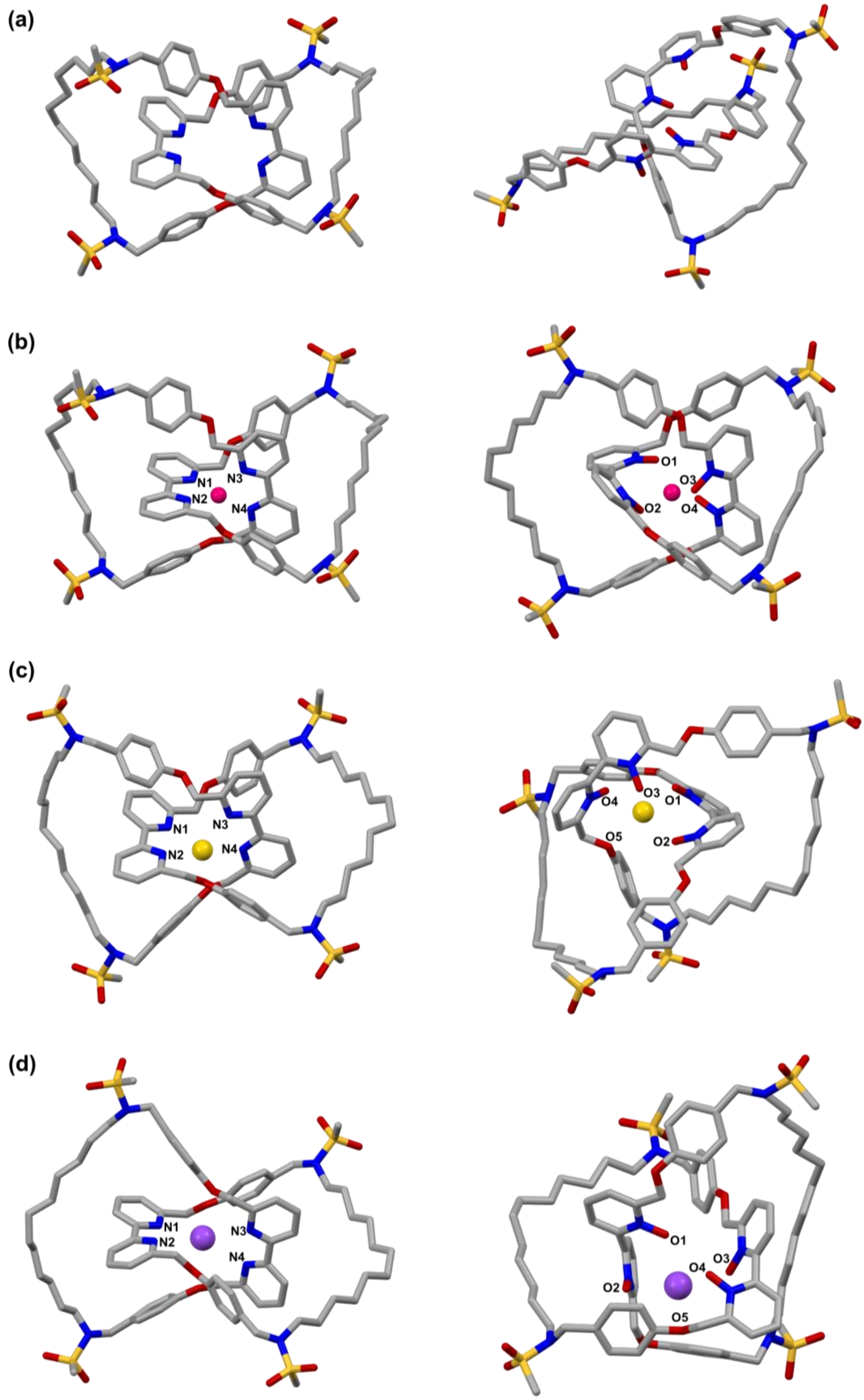

Figure S65. DFT-optimized structures of (a) **C12** (left) and **C12-NO** (right); (b) $[Li(\mathbf{C12})]^+$ (left) and $[Li(\mathbf{C12\text{-}NO})]^+$ (right); (c) $[Na(\mathbf{C12})]^+$ (left) and $[Na(\mathbf{C12\text{-}NO})]^+$ (right); and (d) $[K(\mathbf{C12})]^+$ (left) and $[K(\mathbf{C12\text{-}NO})]^+$ (right). Hydrogen atoms are omitted for clarity. Colour code: grey, C; blue, N; red, O; yellow, S; pink, Li^+; yellow, Na^+; purple, K^+.

Table S26. Cartesian coordinates of **C12** at its optimized geometry.

Atom		x	y	z	Atom		x	y	z
1	O	-0.9046	3.7269	0.2676	46	C	9.3927	-2.3304	-1.0237
2	N	-2.4915	1.3418	-0.3870	47	C	9.1149	-3.1482	0.2494
3	N	4.4028	7.2721	-0.7707	48	C	7.7887	-4.9395	1.4426
4	S	4.9803	8.6350	-1.5583	49	S	7.3722	-7.5535	0.9670
5	O	3.8133	9.4343	-1.9661	50	O	8.3940	-7.3621	-0.0740
6	O	5.9285	8.1680	-2.5819	51	O	6.2078	-8.4109	0.6891
7	C	5.9101	9.6004	-0.3530	52	C	8.1981	-8.2109	2.4285
8	C	-1.8160	3.6291	-0.8373	53	O	0.2301	-1.3039	-3.3314
9	C	3.3652	7.4992	0.2661	54	N	2.1516	-0.0129	-0.6180
10	C	2.2607	6.4630	0.2219	55	N	-5.6114	-0.8925	-6.0981
11	C	1.4043	6.3788	-0.8818	56	S	-6.7513	0.1579	-6.7613
12	C	0.3417	5.4743	-0.9153	57	O	-7.9970	-0.0059	-5.9966
13	C	0.1198	4.6267	0.1806	58	O	-6.1244	1.4876	-6.8332
14	C	0.9823	4.6836	1.2851	59	C	-7.0700	-0.3915	-8.4486
15	C	2.0362	5.5940	1.2988	60	C	0.5837	-0.2595	-2.4229
16	C	-2.8826	2.6227	-0.4781	61	C	1.9319	-0.5731	-1.8149
17	C	-4.2032	3.0284	-0.2637	62	C	-4.2853	-0.8673	-6.7724
18	C	-3.4084	0.4081	-0.0810	63	C	-3.1319	-1.0008	-5.8001
19	C	-4.7572	0.7292	0.1402	64	C	-2.2087	-2.0491	-5.9171
20	C	-5.1559	2.0589	0.0473	65	C	-1.0987	-2.1290	-5.0799
21	C	5.4199	5.1012	-1.5663	66	C	-0.8900	-1.1516	-4.0971
22	C	7.5548	1.8076	-1.8961	67	C	-1.8140	-0.1064	-3.9521
23	C	5.3901	6.1957	-0.4964	68	C	-2.9187	-0.0423	-4.8027
24	C	6.4421	4.0100	-1.2223	69	C	2.8872	-1.3510	-2.4775
25	C	6.5300	2.8888	-2.2662	70	C	3.3487	-0.1901	-0.0318
26	C	7.6541	0.6720	-2.9247	71	C	4.3638	-0.9543	-0.6227
27	O	0.8535	-3.5761	0.4660	72	C	4.1205	-1.5465	-1.8607
28	N	-1.7041	-1.2087	0.5168	73	C	-7.5572	-3.6755	-1.4420
29	N	6.7868	-6.0353	1.3691	74	C	-6.7319	-2.3296	-4.3261
30	C	0.1129	-2.6341	1.2522	75	C	-8.6092	-3.8243	-0.3312
31	C	-1.2505	-2.4644	0.6196	76	C	-8.1732	-3.8853	-2.8337
32	C	5.6570	-6.0009	2.3308	77	C	-7.2137	-3.7596	-4.0285
33	C	4.4330	-5.2720	1.8110	78	C	-6.1018	-2.2467	-5.7204
34	C	3.8069	-5.6783	0.6200	79	O	0.3402	1.6019	3.6422
35	C	2.6246	-5.0875	0.1949	80	N	2.4996	0.4549	2.1438
36	C	2.0283	-4.0686	0.9583	81	C	1.4549	0.9651	4.2778
37	C	2.6505	-3.6329	2.1331	82	C	-4.7476	1.9281	6.1780
38	C	3.8437	-4.2410	2.5452	83	C	-3.4068	1.7645	5.4958
39	C	-1.9952	-3.5779	0.2102	84	C	-2.3195	1.2213	6.1839
40	C	-2.9368	-1.0067	0.0127	85	C	-1.0491	1.1341	5.6034
41	C	-3.7465	-2.0631	-0.4242	86	C	-0.8573	1.6044	4.2997
42	C	-3.2612	-3.3667	-0.3256	87	C	-1.9445	2.1398	3.5866
43	C	8.3325	-1.2684	-1.3472	88	C	-3.1980	2.2155	4.1806
44	C	7.9577	-4.1532	0.1382	89	C	2.6441	1.0281	3.3485
45	C	8.6846	-0.4175	-2.5772	90	C	3.8379	1.6240	3.7658
91	C	3.5489	0.4489	1.3042	139	H	-1.5837	-4.5769	0.3074
92	C	4.7890	1.0082	1.6511	140	H	-4.7229	-1.8684	-0.8543

93	C	4.9305	1.6053	2.8998
94	N	-5.8167	1.0680	5.5854
95	C	-6.4718	-1.4225	5.2496
96	C	-7.3746	-2.6124	1.6190
97	C	-5.6256	-0.3635	5.9620
98	C	-6.4088	-1.4357	3.7169
99	C	-7.1584	-2.6458	3.1388
100	C	-8.0742	-3.8822	1.1082
101	S	-7.3710	1.7291	5.7587
102	O	-7.2620	3.1611	5.4341
103	O	-8.3085	0.9075	4.9794
104	C	-7.8249	1.5821	7.4958
105	H	5.2388	9.9159	0.4489
106	H	6.3130	10.4741	-0.8726
107	H	6.7252	8.9905	0.0434
108	H	-1.2730	3.3024	-1.7331
109	H	-2.2708	4.6053	-1.0392
110	H	2.9361	8.4882	0.0883
111	H	3.8283	7.5089	1.2611
112	H	1.5631	7.0370	-1.7327
113	H	-0.3033	5.4443	-1.7859
114	H	0.8181	4.0043	2.1166
115	H	2.6910	5.6342	2.1665
116	H	-4.4734	4.0769	-0.3411
117	H	-5.4725	-0.0446	0.3968
118	H	-6.1913	2.3367	0.2227
119	H	5.6645	5.5550	-2.5336
120	H	4.4173	4.6634	-1.6595
121	H	7.2921	1.3970	-0.9112
122	H	8.5456	2.2724	-1.7782
123	H	5.1143	5.7619	0.4700
124	H	6.3937	6.6215	-0.3716
125	H	6.1890	3.5728	-0.2450
126	H	7.4348	4.4699	-1.1040
127	H	5.5398	2.4251	-2.3860
128	H	6.7886	3.3202	-3.2446
129	H	7.9142	1.1044	-3.9017
130	H	6.6645	0.2083	-3.0509
131	H	0.0029	-3.0154	2.2774
132	H	0.6246	-1.6673	1.2959
133	H	5.9957	-5.5452	3.2679
134	H	5.3760	-7.0328	2.5598
135	H	4.2488	-6.4775	0.0305
136	H	2.1345	-5.4105	-0.7192
137	H	2.2288	-2.8271	2.7234
138	H	4.3126	-3.9032	3.4669
187	H	-2.4547	0.8670	7.2033
188	H	-0.2313	0.7125	6.1761
189	H	-1.7796	2.4955	2.5739
141	H	-3.8637	-4.2038	-0.6669
142	H	7.3589	-1.7500	-1.5140
143	H	8.2105	-0.6204	-0.4680
144	H	8.1616	-4.8567	-0.6769
145	H	7.0147	-3.6457	-0.1001
146	H	9.6675	0.0531	-2.4223
147	H	8.8001	-1.0840	-3.4441
148	H	10.3637	-1.8279	-0.9073
149	H	9.5038	-3.0142	-1.8785
150	H	10.0258	-3.7042	0.5139
151	H	8.9261	-2.4586	1.0861
152	H	8.7608	-5.3409	1.7569
153	H	7.4569	-4.2695	2.2417
154	H	7.4730	-8.2986	3.2409
155	H	9.0140	-7.5412	2.7096
156	H	8.5935	-9.1964	2.1676
157	H	-7.4570	-1.4128	-8.4292
158	H	-6.1445	-0.3396	-9.0262
159	H	-7.8170	0.2846	-8.8731
160	H	-0.1585	-0.1580	-1.6241
161	H	0.6341	0.6994	-2.9593
162	H	-4.2006	0.0890	-7.2942
163	H	-4.2320	-1.6634	-7.5265
164	H	-2.3511	-2.8072	-6.6839
165	H	-0.3805	-2.9379	-5.1791
166	H	-1.6849	0.6494	-3.1862
167	H	-3.6249	0.7764	-4.6882
168	H	2.6616	-1.7921	-3.4415
169	H	5.3091	-1.1045	-0.1131
170	H	4.8822	-2.1590	-2.3348
171	H	-6.7561	-4.4151	-1.2893
172	H	-7.0841	-2.6860	-1.3814
173	H	-5.9978	-1.9965	-3.5818
174	H	-7.5797	-1.6392	-4.2759
175	H	-9.1783	-4.7470	-0.5179
176	H	-9.3348	-3.0009	-0.4129
177	H	-9.0039	-3.1769	-2.9718
178	H	-8.6241	-4.8879	-2.8574
179	H	-6.3484	-4.4227	-3.8817
180	H	-7.7376	-4.1381	-4.9184
181	H	-5.2247	-2.8994	-5.7617
182	H	-6.8070	-2.6263	-6.4732
183	H	1.2035	-0.0825	4.4917
184	H	1.6910	1.4638	5.2247
185	H	-5.0774	2.9635	6.0823
186	H	-4.6404	1.7033	7.2489

190	H	-4.0283	2.6378	3.6195					
191	H	3.9056	2.0864	4.7456					
192	H	5.6155	0.9911	0.9484					
193	H	5.8741	2.0586	3.1899					
194	H	-7.5177	-1.3497	5.5631					
195	H	-6.1015	-2.3844	5.6335					
196	H	-6.4096	-2.4823	1.1096					
197	H	-7.9827	-1.7315	1.3629					
198	H	-5.7393	-0.4781	7.0515					
199	H	-4.5719	-0.5533	5.7369					
200	H	-5.3611	-1.4516	3.3827					
201	H	-6.8503	-0.5091	3.3320					
202	H	-6.6202	-3.5664	3.4104					
203	H	-8.1431	-2.7182	3.6249					
204	H	-7.3873	-4.7364	1.2066					
205	H	-8.9227	-4.0997	1.7736					
206	H	-7.1001	2.1262	8.1058					
207	H	-7.8510	0.5267	7.7752					
208	H	-8.8188	2.0238	7.6068					

Table S27. Cartesian coordinates of **C12-NO** at its optimized geometry.

Atom		x	y	z	Atom		x	y	z
1	O	2.5641	1.5814	0.9579	46	C	-6.4022	-3.1066	0.6637
2	N	0.4569	2.7289	-1.7461	47	C	-7.5366	-3.3008	-0.3527
3	N	3.4639	-4.1907	3.7715	48	C	-9.4261	-2.1964	-1.6664
4	S	4.3632	-5.5987	3.6456	49	S	-11.705	-0.9182	-1.0118
5	O	5.4749	-5.3331	2.7176	50	O	-11.457	-1.5187	0.3082
6	O	3.4199	-6.6873	3.3477	51	O	-12.208	0.4625	-1.0945
7	C	5.0726	-5.9213	5.2704	52	C	-12.870	-1.9795	-1.8858
8	C	1.7071	1.4147	-0.1660	53	O	-2.5127	3.3817	-1.7850
9	C	4.2238	-2.9478	4.0103	54	O	-0.0344	1.5938	-2.1179
10	C	3.7269	-1.7725	3.1853	55	O	4.7483	-3.5678	-2.5205
11	C	3.0979	-1.9433	1.9516	56	N	1.9269	-3.8341	-1.4662
12	C	2.6792	-0.8550	1.1791	57	N	9.5314	0.8028	-2.8854
13	C	2.9205	0.4441	1.6390	58	S	11.1641	1.2117	-2.9679
14	C	3.5643	0.6363	2.8704	59	O	11.4394	2.1380	-1.8595
15	C	3.9505	-0.4617	3.6338	60	O	11.9251	-0.0447	-3.0572
16	C	1.3834	2.7649	-0.7355	61	C	11.4106	2.1170	-4.5064
17	C	1.9573	3.9710	-0.3591	62	C	4.3573	-3.7408	-1.1496
18	C	0.0678	3.8932	-2.3735	63	C	3.0738	-4.5090	-1.0933
19	C	0.6476	5.1028	-2.0187	64	C	9.0820	-0.1425	-3.9345
20	C	1.6013	5.1548	-1.0027	65	C	7.9226	-1.0166	-3.4924
21	C	0.9974	-4.5288	3.3738	66	C	7.7855	-1.4785	-2.1805
22	C	-2.8370	-5.3429	3.6338	67	C	6.7315	-2.3189	-1.8096
23	C	2.1181	-4.3067	4.3954	68	C	5.7989	-2.7312	-2.7709
24	C	-0.3676	-4.7688	4.0308	69	C	5.9321	-2.2851	-4.0942
25	C	-1.4959	-4.9419	3.0030	70	C	6.9756	-1.4330	-4.4410
26	C	-3.9922	-5.5180	2.6316	71	C	3.0001	-5.8269	-0.6640

27	O	-5.3538	2.3283	-4.4235	72	C	0.7019	-4.4666	-1.3654
28	N	-2.2195	3.4392	-3.0417	73	C	0.6312	-5.7942	-0.9522
29	N	-10.256	-0.9770	-1.8522	74	C	1.7789	-6.4949	-0.5947
30	C	-4.5276	2.8378	-3.3814	75	C	6.0293	4.4750	1.0358
31	C	-3.1989	3.2080	-3.9730	76	C	8.4267	2.1733	-1.0649
32	C	-10.237	-0.3394	-3.1859	77	C	6.0742	5.1040	2.4373
33	C	-8.9266	0.3676	-3.4744	78	C	7.2573	3.6196	0.6967
34	C	-8.3240	1.2030	-2.5280	79	C	7.2972	3.1706	-0.7715
35	C	-7.1301	1.8730	-2.8020	80	C	8.5859	1.9092	-2.5654
36	C	-6.5253	1.7207	-4.0567	81	O	-2.6638	-0.8620	0.5924
37	C	-7.1268	0.9049	-5.0233	82	N	-0.8441	-2.5869	-1.0910
38	C	-8.3105	0.2335	-4.7262	83	C	-2.2121	-0.5813	-0.7412
39	C	-2.9217	3.3378	-5.3269	84	C	-3.5106	3.3128	4.3788
40	C	-0.9371	3.7393	-3.4465	85	C	-3.2040	2.2447	3.3458
41	C	-0.6515	3.8926	-4.7958	86	C	-2.9373	2.5478	2.0071
42	C	-1.6458	3.6983	-5.7542	87	C	-2.7321	1.5445	1.0540
43	C	-5.7138	-4.4133	1.0803	88	C	-2.8129	0.1986	1.4406
44	C	-8.3215	-2.0101	-0.6213	89	C	-3.0758	-0.1208	2.7812
45	C	-4.5637	-4.2052	2.0763	90	C	-3.2602	0.8923	3.7162
91	C	-1.9592	-1.8639	-1.4687	139	H	-8.7864	1.3275	-1.5527
92	C	-2.7593	-2.3110	-2.5111	140	H	-6.6872	2.5056	-2.0408
93	C	-0.4957	-3.7211	-1.7994	141	H	-6.6458	0.7895	-5.9902
94	C	-1.3066	-4.1759	-2.8353	142	H	-8.7578	-0.4117	-5.4790
95	C	-2.4532	-3.4801	-3.2049	143	H	-3.7231	3.1535	-6.0325
96	N	-2.6584	4.5207	4.2412	144	H	0.3639	4.1466	-5.0807
97	C	-0.2188	5.1266	3.8326	145	H	-1.4260	3.8119	-6.8103
98	C	3.6623	4.8618	3.3145	146	H	-6.4630	-5.0841	1.5273
99	C	-1.2383	4.3222	4.6459	147	H	-5.3336	-4.9315	0.1872
100	C	1.2078	4.5877	4.0134	148	H	-7.6394	-1.2191	-0.9579
101	C	2.2755	5.5113	3.4081	149	H	-8.7804	-1.6607	0.3110
102	C	4.7675	5.8168	2.8357	150	H	-3.7711	-3.6186	1.5918
103	S	-3.4070	5.9474	4.7450	151	H	-4.9250	-3.5960	2.9188
104	O	-4.7208	6.0021	4.0841	152	H	-5.6553	-2.4142	0.2482
105	O	-2.4569	7.0487	4.5309	153	H	-6.8060	-2.6164	1.5621
106	C	-3.6865	5.8063	6.5199	154	H	-7.1213	-3.6886	-1.2948
107	O	1.9995	-2.6366	-1.9157	155	H	-8.2288	-4.0712	0.0187
108	O	-0.1445	-2.2129	-0.0846	156	H	-8.9921	-2.4354	-2.6428
109	H	5.7327	-5.0952	5.5445	157	H	-10.060	-3.0463	-1.3888
110	H	5.6421	-6.8521	5.2020	158	H	-12.471	-2.9960	-1.9212
111	H	4.2645	-6.0274	5.9979	159	H	-13.021	-1.5891	-2.8948
112	H	0.7845	0.8993	0.1273	160	H	-13.811	-1.9641	-1.3301
113	H	2.1831	0.8012	-0.9434	161	H	10.7785	3.0078	-4.5065
114	H	5.2691	-3.1445	3.7530	162	H	11.1666	1.4694	-5.3512
115	H	4.1986	-2.6876	5.0774	163	H	12.4646	2.4051	-4.5455
116	H	2.9148	-2.9466	1.5824	164	H	4.2289	-2.7602	-0.6804
117	H	2.1629	-1.0521	0.2467	165	H	5.1218	-4.3021	-0.6020
118	H	3.7399	1.6478	3.2239	166	H	9.9379	-0.7763	-4.1842
119	H	4.4338	-0.2949	4.5942	167	H	8.7983	0.3993	-4.8480
120	H	2.6918	3.9616	0.4369	168	H	8.5073	-1.1754	-1.4278

121	H	0.3300	5.9990	-2.5410	169	H	6.6537	-2.6478	-0.7794
122	H	2.0550	6.0980	-0.7180	170	H	5.1989	-2.6000	-4.8310
123	H	1.2590	-5.3918	2.7515	171	H	7.0543	-1.0819	-5.4677
124	H	0.9399	-3.6601	2.7070	172	H	3.9246	-6.3210	-0.3827
125	H	-3.1258	-4.5986	4.3915	173	H	-0.3474	-6.2600	-0.9043
126	H	-2.6929	-6.2881	4.1767	174	H	1.7228	-7.5261	-0.2641
127	H	1.9513	-3.3763	4.9472	175	H	5.9307	5.2816	0.2936
128	H	2.1139	-5.1161	5.1363	176	H	5.1289	3.8535	0.9318
129	H	-0.6117	-3.9324	4.7025	177	H	8.2191	1.2226	-0.5571
130	H	-0.3117	-5.6665	4.6647	178	H	9.3727	2.5499	-0.6632
131	H	-1.6042	-4.0108	2.4296	179	H	6.9078	5.8194	2.4713
132	H	-1.2032	-5.7148	2.2763	180	H	6.3070	4.3300	3.1844
133	H	-4.8075	-6.0671	3.1242	181	H	7.2725	2.7360	1.3518
134	H	-3.6560	-6.1557	1.7998	182	H	8.1745	4.1837	0.9224
135	H	-4.3798	2.0853	-2.5976	183	H	6.3326	2.7181	-1.0449
136	H	-4.9840	3.7161	-2.9035	184	H	7.4116	4.0582	-1.4115
137	H	-11.054	0.3873	-3.2104	185	H	7.6239	1.6010	-2.9849
138	H	-10.433	-1.0839	-3.9693	186	H	8.8718	2.8329	-3.0875
187	H	-1.2965	0.0181	-0.7148					
188	H	-2.9810	-0.0206	-1.2840					
189	H	-3.4092	2.8831	5.3855					
190	H	-4.5489	3.6364	4.2649					
191	H	-2.8852	3.5868	1.6932					
192	H	-2.5220	1.8285	0.0278					
193	H	-3.1222	-1.1652	3.0751					
194	H	-3.4557	0.6273	4.7530					
195	H	-3.6311	-1.7185	-2.7699					
196	H	-1.0080	-5.0804	-3.3548					
197	H	-3.0808	-3.8306	-4.0168					
198	H	-0.2620	6.1805	4.1223					
199	H	-0.4940	5.0781	2.7711					
200	H	3.5960	4.0005	2.6357					
201	H	3.9466	4.4544	4.2965					
202	H	-1.0543	3.2557	4.4865					
203	H	-1.1061	4.5047	5.7226					
204	H	1.2762	3.5931	3.5493					
205	H	1.4215	4.4429	5.0829					
206	H	1.9600	5.8184	2.4001					
207	H	2.3341	6.4347	4.0032					
208	H	4.4036	6.4005	1.9764					
209	H	4.9718	6.5463	3.6318					
210	H	-4.3486	4.9603	6.7169					
211	H	-4.1581	6.7372	6.8461					
212	H	-2.7284	5.6741	7.0278					

Table S28. Cartesian coordinates of [Li(**C12**)]$^+$ at its optimized geometry.

Atom		x	y	z	Atom		x	y	z
1	O	0.3251	3.7015	-0.3581	46	C	9.5035	-3.2700	0.0488
2	N	-1.5950	1.4892	-0.2727	47	C	8.9894	-3.9222	1.3429
3	N	5.8343	6.7692	-1.8082	48	C	7.4273	-5.5651	2.4654
4	S	6.6207	7.9034	-2.7614	49	S	6.7616	-8.1239	1.9514
5	O	5.5864	8.7026	-3.4378	50	O	7.8452	-8.0400	0.9601
6	O	7.6090	7.1705	-3.5678	51	O	5.5267	-8.8495	1.6109
7	C	7.5236	8.9989	-1.6514	52	C	7.4425	-8.8717	3.4436
8	C	-0.4198	3.2481	-1.4952	53	O	-0.1253	-1.8191	-3.2328
9	C	4.7290	7.2729	-0.9620	54	N	1.5877	0.0030	-0.6490
10	C	3.5953	6.2739	-0.8333	55	N	-5.9145	-2.1421	-6.1478
11	C	2.9930	5.7077	-1.9620	56	S	-7.0434	-1.3132	-7.0868
12	C	1.9088	4.8341	-1.8511	57	O	-8.3033	-1.2796	-6.3289
13	C	1.3979	4.5242	-0.5824	58	O	-6.4185	-0.0449	-7.4949
14	C	1.9942	5.0787	0.5584	59	C	-7.3268	-2.2927	-8.5724
15	C	3.0833	5.9366	0.4272	60	C	0.0011	-0.7296	-2.3236
16	C	-1.6985	2.6078	-1.0117	61	C	1.4118	-0.6983	-1.7814
17	C	-2.9331	3.1549	-1.3676	62	C	-4.5708	-2.2664	-6.7642
18	C	-2.7179	0.8530	0.1211	63	C	-3.4486	-2.1696	-5.7501
19	C	-3.9913	1.3491	-0.1944	64	C	-2.3758	-3.0716	-5.7938
20	C	-4.0958	2.5151	-0.9440	65	C	-1.2840	-2.9354	-4.9410
21	C	6.6585	4.4052	-2.0685	66	C	-1.2481	-1.8865	-4.0121
22	C	8.3186	0.8672	-1.6053	67	C	-2.3260	-0.9945	-3.9321
23	C	6.6749	5.6869	-1.2318	68	C	-3.4064	-1.1398	-4.8052
24	C	7.4993	3.2909	-1.4336	69	C	2.4693	-1.3150	-2.4547
25	C	7.4975	1.9916	-2.2496	70	C	2.8350	0.1363	-0.1478
26	C	8.3326	-0.4273	-2.4308	71	C	3.9430	-0.4601	-0.7613
27	O	0.9059	-3.0471	1.6466	72	C	3.7507	-1.1966	-1.9268
28	N	-1.2941	-0.6777	1.3514	73	C	-8.0126	-3.6008	-0.9823
29	N	6.3208	-6.5504	2.3377	74	C	-7.0651	-3.0803	-4.0872
30	C	0.3173	-2.0879	2.5412	75	C	-9.1329	-3.4467	0.0593
31	C	-1.0802	-1.8088	2.0427	76	C	-8.5511	-4.2052	-2.2885
32	C	5.1921	-6.4131	3.2918	77	C	-7.5370	-4.3877	-3.4291
33	C	4.1304	-5.4370	2.8274	78	C	-6.4026	-3.3579	-5.4406
34	C	3.4266	-5.6601	1.6314	79	O	-0.4168	2.3357	3.0278
35	C	2.3715	-4.8389	1.2571	80	N	1.8418	1.1061	1.8318
36	C	1.9883	-3.7652	2.0790	81	C	0.6528	1.8367	3.8354
37	C	2.7035	-3.5018	3.2517	82	C	-5.4593	3.5768	5.3800
38	C	3.7626	-4.3443	3.6140	83	C	-4.1265	3.2028	4.7697
39	C	-2.0954	-2.7376	2.2928	84	C	-3.1259	2.6020	5.5366
40	C	-2.5376	-0.4155	0.8882	85	C	-1.8742	2.2874	4.9960
41	C	-3.5982	-1.3068	1.0920	86	C	-1.6107	2.5876	3.6554
42	C	-3.3709	-2.4818	1.8036	87	C	-2.6035	3.1979	2.8711
43	C	8.5920	-2.1860	-0.5433	88	C	-3.8436	3.4925	3.4243
44	C	7.7730	-4.8426	1.1598	89	C	1.9121	1.7497	3.0089
45	C	9.1716	-1.5641	-1.8229	90	C	3.1111	2.2618	3.5095
91	C	2.9701	0.9154	1.1170	139	H	4.2986	-4.1442	4.5391
92	C	4.2086	1.4035	1.5549	140	H	-1.8776	-3.6390	2.8564

93	C	4.2742	2.0889	2.7632
94	N	-6.5696	2.6885	4.9175
95	C	-7.3783	0.2240	5.0484
96	C	-8.0161	-1.7718	1.7250
97	C	-6.5155	1.3626	5.5986
98	C	-7.1878	-0.1324	3.5680
99	C	-7.9203	-1.4372	3.2207
100	C	-8.6943	-3.1327	1.4974
101	S	-8.0702	3.4750	4.8331
102	O	-7.8273	4.7835	4.2032
103	O	-9.0283	2.5596	4.1964
104	C	-8.6238	3.7639	6.5231
105	Li	0.1114	0.7482	0.7032
106	H	6.8132	9.5090	-0.9969
107	H	8.0565	9.7270	-2.2690
108	H	8.2346	8.4106	-1.0665
109	H	0.1756	2.5229	-2.0653
110	H	-0.6614	4.0915	-2.1496
111	H	4.3545	8.1879	-1.4289
112	H	5.1011	7.5369	0.0371
113	H	3.3749	5.9491	-2.9505
114	H	1.4743	4.4120	-2.7500
115	H	1.5911	4.8343	1.5365
116	H	3.5348	6.3609	1.3210
117	H	-2.9745	4.0619	-1.9614
118	H	-4.8873	0.8462	0.1470
119	H	-5.0721	2.9195	-1.1936
120	H	7.0347	4.6335	-3.0725
121	H	5.6199	4.0688	-2.1839
122	H	7.9219	0.6676	-0.5999
123	H	9.3542	1.2109	-1.4622
124	H	6.2817	5.4862	-0.2299
125	H	7.7069	6.0335	-1.0984
126	H	7.1237	3.0840	-0.4202
127	H	8.5353	3.6403	-1.3107
128	H	6.4602	1.6512	-2.3861
129	H	7.8876	2.1965	-3.2576
130	H	8.7229	-0.1980	-3.4329
131	H	7.2999	-0.7776	-2.5805
132	H	0.2653	-2.5086	3.5516
133	H	0.9149	-1.1721	2.5640
134	H	5.5746	-6.1119	4.2743
135	H	4.7316	-7.3976	3.4091
136	H	3.7003	-6.5011	0.9992
137	H	1.8134	-5.0259	0.3440
138	H	2.4497	-2.6617	3.8882
187	H	-5.3874	3.5392	6.4759
188	H	-3.3153	2.3773	6.5837
189	H	-1.1268	1.8213	5.6271
141	H	-4.5833	-1.1037	0.6908
142	H	-4.1809	-3.1856	1.9696
143	H	7.6011	-2.6062	-0.7678
144	H	8.4328	-1.4071	0.2157
145	H	7.9925	-5.5893	0.3890
146	H	6.8968	-4.2754	0.8214
147	H	10.1855	-1.1891	-1.6161
148	H	9.2896	-2.3586	-2.5738
149	H	10.4866	-2.8215	0.2520
150	H	9.6766	-4.0538	-0.7038
151	H	9.8063	-4.5151	1.7790
152	H	8.7553	-3.1387	2.0792
153	H	8.3251	-6.0521	2.8687
154	H	7.1044	-4.8386	3.2174
155	H	6.6758	-8.8839	4.2217
156	H	8.3132	-8.2970	3.7671
157	H	7.7392	-9.8934	3.1917
158	H	-7.7090	-3.2755	-8.2880
159	H	-6.3913	-2.3869	-9.1280
160	H	-8.0697	-1.7606	-9.1728
161	H	-0.7175	-0.8296	-1.5038
162	H	-0.2016	0.2198	-2.8384
163	H	-4.4658	-1.4533	-7.4878
164	H	-4.4897	-3.2116	-7.3172
165	H	-2.3883	-3.8864	-6.5142
166	H	-0.4500	-3.6297	-4.9845
167	H	-2.3343	-0.1869	-3.2098
168	H	-4.2301	-0.4336	-4.7460
169	H	2.2804	-1.8753	-3.3619
170	H	4.9328	-0.3754	-0.3297
171	H	4.5938	-1.6757	-2.4155
172	H	-7.2237	-4.2555	-0.5814
173	H	-7.5407	-2.6281	-1.1765
174	H	-6.3548	-2.5482	-3.4421
175	H	-7.9221	-2.4169	-4.2406
176	H	-9.7093	-4.3834	0.0825
177	H	-9.8345	-2.6707	-0.2820
178	H	-9.3843	-3.5867	-2.6549
179	H	-8.9859	-5.1871	-2.0521
180	H	-6.6697	-4.9609	-3.0702
181	H	-8.0125	-5.0099	-4.2013
182	H	-5.5197	-3.9869	-5.2931
183	H	-7.0874	-3.9305	-6.0819
184	H	0.3959	0.8381	4.2161
185	H	0.8205	2.4967	4.6931
186	H	-5.7152	4.5970	5.0917

190	H	-2.3812	3.4461	1.8383					
191	H	-4.6026	3.9671	2.8069					
192	H	3.1244	2.7847	4.4601					
193	H	5.1027	1.2647	0.9598					
194	H	5.2212	2.4852	3.1169					
195	H	-8.4381	0.4208	5.2353					
196	H	-7.1135	-0.6491	5.6629					
197	H	-7.0139	-1.7665	1.2725					
198	H	-8.5902	-0.9838	1.2149					
199	H	-6.7186	1.4892	6.6734					
200	H	-5.4653	1.0682	5.5131					
201	H	-6.1166	-0.2342	3.3398					
202	H	-7.5684	0.6871	2.9467					
203	H	-7.4314	-2.2708	3.7475					
204	H	-8.9427	-1.3861	3.6246					
205	H	-8.0268	-3.9309	1.8557					
206	H	-9.5883	-3.1795	2.1367					
207	H	-7.8907	4.3875	7.0402					
208	H	-8.7469	2.8067	7.0346					
209	H	-9.5842	4.2829	6.4625					

Table S29. Cartesian coordinates of [Na(**C12**)]$^{+}$ at its optimized geometry.

Atom		x	y	z	Atom		x	y	z
1	N	-1.8435	0.2904	-0.8527	46	N	6.0527	-4.8898	3.8027
2	N	5.2041	5.9934	-4.3053	47	O	0.1139	-2.7502	2.3026
3	O	0.1041	2.8084	-2.0755	48	C	-4.5576	-2.6701	1.8001
4	C	-3.0715	0.0942	-0.3206	49	C	-4.4586	-1.6662	0.8416
5	C	-4.1593	0.9138	-0.6419	50	C	-3.2288	-1.0151	0.6655
6	C	-3.9802	1.9504	-1.5560	51	C	-3.4370	-2.9966	2.5616
7	C	-2.7266	2.1333	-2.1245	52	C	2.6395	-3.9172	4.7481
8	C	-1.6830	1.2815	-1.7422	53	C	1.4706	-3.3256	4.2594
9	C	-0.3185	1.4685	-2.3647	54	C	1.2472	-3.2885	2.8803
10	C	2.8056	3.4054	-4.5370	55	C	2.2022	-3.8235	2.0038
11	C	1.8403	2.7012	-3.8059	56	C	3.3552	-4.4165	2.5106
12	C	1.0893	3.3747	-2.8375	57	C	3.5884	-4.4843	3.8937
13	C	1.3298	4.7381	-2.5950	58	C	4.7755	-5.2242	4.4798
14	C	2.2953	5.4163	-3.3265	59	C	-2.2483	-2.3035	2.3251
15	C	3.0467	4.7637	-4.3193	60	C	-1.0244	-2.5692	3.1657
16	C	4.0678	5.5205	-5.1426	61	C	6.5419	-3.5227	4.1247
17	C	9.6916	2.6868	0.9874	62	C	7.5554	-2.9442	3.1345
18	C	8.8468	2.6235	-0.2936	63	C	7.7195	-1.4280	3.3124
19	C	8.3323	3.9770	-0.7986	64	C	8.8564	-0.8453	2.4605
20	C	7.5475	3.8401	-2.1100	65	C	8.7988	0.6820	2.3342
21	C	6.8553	5.1301	-2.5699	66	C	9.9870	1.2904	1.5680
22	C	6.1398	4.9119	-3.9055	67	C	-8.3457	-0.1243	-3.5635
23	N	1.2444	1.0155	1.5694	68	N	2.2312	-0.5666	-0.4946
24	N	-7.5784	4.2545	4.1126	69	N	-3.9493	-6.4139	-3.9571
25	O	-1.4771	2.8598	2.5910	70	O	0.6712	-2.7457	-1.2925

26	C	2.6806	3.2847	2.3397	71	C	3.0940	0.1761	0.2234
27	C	3.2552	2.3352	1.4976	72	C	4.4818	0.0057	0.1047
28	C	2.5124	1.2048	1.1373	73	C	4.9714	-0.9447	-0.7856
29	C	1.3852	3.0810	2.7970	74	C	4.0733	-1.7025	-1.5349
30	C	-4.5716	2.3490	4.5605	75	C	2.7070	-1.4792	-1.3556
31	C	-3.3319	2.0528	3.9785	76	C	1.6909	-2.2390	-2.1733
32	C	-2.6922	3.0213	3.1988	77	C	-1.2129	-4.9752	-3.5758
33	C	-3.3099	4.2670	2.9938	78	C	-0.3403	-3.9647	-3.1665
34	C	-4.5393	4.5404	3.5787	79	C	-0.1802	-3.7065	-1.7989
35	C	-5.1908	3.5879	4.3815	80	C	-0.9051	-4.4465	-0.8583
36	C	-6.5021	3.9100	5.0715	81	C	-1.7734	-5.4479	-1.2879
37	C	0.6999	1.9318	2.3841	82	C	-1.9354	-5.7369	-2.6500
38	C	-0.7059	1.6871	2.8852	83	C	-2.8275	-6.8776	-3.0951
39	C	-8.1216	3.1374	3.3022	84	C	-7.3939	-1.1656	-2.9525
40	C	-8.2068	3.4417	1.8043	85	C	-7.0114	-2.3221	-3.8859
41	C	-8.6191	2.2022	0.9987	86	C	-6.2434	-3.4246	-3.1419
42	C	-8.5322	2.3980	-0.5214	87	C	-5.5395	-4.4368	-4.0554
43	C	-8.7294	1.0926	-1.3060	88	C	-4.9787	-5.6082	-3.2431
44	C	-8.3707	1.2101	-2.7949	89	Na	0.0195	-0.8849	0.4162
45	N	-2.1456	-1.3436	1.3932	90	C	-8.1532	6.8721	3.5207
91	S	-8.6490	5.4826	4.5455	139	H	-0.6861	1.5209	3.9708
92	O	-8.4090	5.8667	5.9487	140	H	-9.0962	2.8217	3.6898
93	O	-10.006	5.0835	4.1359	141	H	-7.4281	2.3055	3.4554
94	C	-3.4395	-7.5373	-6.3791	142	H	-7.2234	3.7918	1.4630
95	S	-4.5554	-7.6327	-4.9760	143	H	-8.9180	4.2584	1.6258
96	O	-5.8979	-7.2152	-5.4121	144	H	-9.6376	1.8974	1.2795
97	O	-4.4064	-8.9686	-4.3646	145	H	-7.9613	1.3662	1.2805
98	C	7.7649	-6.2582	2.1486	146	H	-7.5402	2.8076	-0.7643
99	S	7.2304	-6.1257	3.8573	147	H	-9.2624	3.1528	-0.8474
100	O	8.3831	-5.7016	4.6743	148	H	-9.7663	0.7409	-1.1980
101	O	6.5524	-7.3890	4.1993	149	H	-8.0979	0.3227	-0.8435
102	C	5.8579	8.4581	-3.2987	150	H	-7.3750	1.6715	-2.8779
103	S	5.9286	7.4547	-4.7864	151	H	-9.0687	1.9018	-3.2879
104	O	5.0598	8.0963	-5.7896	152	H	-5.4960	-3.1965	1.9468
105	O	7.3482	7.2310	-5.1191	153	H	-5.3149	-1.4072	0.2296
106	H	-5.1213	0.7640	-0.1661	154	H	-3.4761	-3.7736	3.3183
107	H	-4.8077	2.6037	-1.8156	155	H	2.8024	-3.9442	5.8229
108	H	-2.5450	2.9222	-2.8468	156	H	0.7567	-2.9048	4.9579
109	H	-0.3955	1.3288	-3.4502	157	H	2.0176	-3.7875	0.9343
110	H	0.4010	0.7456	-1.9668	158	H	4.0796	-4.8423	1.8215
111	H	3.3774	2.8754	-5.2954	159	H	4.6226	-6.2999	4.3746
112	H	1.6904	1.6451	-4.0005	160	H	4.8411	-5.0100	5.5559
113	H	0.7394	5.2474	-1.8387	161	H	-0.8443	-1.7200	3.8381
114	H	2.4646	6.4726	-3.1318	162	H	-1.1741	-3.4662	3.7738
115	H	4.4364	4.8827	-5.9576	163	H	6.9418	-3.4901	5.1474
116	H	3.6077	6.3995	-5.5955	164	H	5.6419	-2.9005	4.1075
117	H	9.1760	3.2951	1.7458	165	H	7.2170	-3.1493	2.1104
118	H	10.6348	3.2061	0.7678	166	H	8.5282	-3.4341	3.2607
119	H	7.9810	1.9658	-0.1300	167	H	7.8991	-1.1920	4.3712

120	H	9.4440	2.1445	-1.0839	168	H	6.7722	-0.9376	3.0446
121	H	9.1741	4.6701	-0.9423	169	H	8.8130	-1.2798	1.4505
122	H	7.6874	4.4289	-0.0303	170	H	9.8239	-1.1534	2.8834
123	H	6.7851	3.0561	-1.9897	171	H	8.7428	1.1383	3.3334
124	H	8.2264	3.4873	-2.9001	172	H	7.8589	0.9466	1.8294
125	H	7.5962	5.9341	-2.6591	173	H	10.8577	1.3369	2.2359
126	H	6.1209	5.4444	-1.8166	174	H	10.2735	0.6212	0.7427
127	H	5.5115	4.0214	-3.8162	175	H	-8.0414	0.0815	-4.6000
128	H	6.8668	4.7182	-4.7049	176	H	-9.3599	-0.5458	-3.6209
129	H	3.2387	4.1703	2.6289	177	H	5.1660	0.5894	0.7092
130	H	4.2561	2.4868	1.1115	178	H	6.0415	-1.0997	-0.8865
131	H	0.9006	3.7937	3.4556	179	H	4.4185	-2.4552	-2.2362
132	H	-5.0574	1.5916	5.1716	180	H	2.1747	-3.0683	-2.6976
133	H	-2.8871	1.0768	4.1376	181	H	1.2354	-1.5742	-2.9192
134	H	-2.8031	5.0091	2.3836	182	H	-1.3251	-5.1732	-4.6389
135	H	-4.9996	5.5121	3.4164	183	H	0.2075	-3.3993	-3.9117
136	H	-6.3723	4.7631	5.7404	184	H	-0.7693	-4.2398	0.1987
137	H	-6.8101	3.0581	5.6922	185	H	-2.3226	-6.0258	-0.5484
138	H	-1.1444	0.8062	2.4062	186	H	-2.2364	-7.5938	-3.6732
187	H	-3.2123	-7.4133	-2.2179					
188	H	-7.8432	-1.5813	-2.0402					
189	H	-6.4682	-0.6630	-2.6329					
190	H	-6.3986	-1.9317	-4.7119					
191	H	-7.9123	-2.7522	-4.3475					
192	H	-6.9406	-3.9515	-2.4736					
193	H	-5.4893	-2.9614	-2.4881					
194	H	-4.7124	-3.9422	-4.5819					
195	H	-6.2296	-4.8102	-4.8189					
196	H	-5.7913	-6.2421	-2.8657					
197	H	-4.4604	-5.2098	-2.3662					
198	H	-7.1224	7.1387	3.7639					
199	H	-8.8240	7.7053	3.7487					
200	H	-8.2408	6.5880	2.4703					
201	H	-2.4147	-7.7171	-6.0470					
202	H	-3.5333	-6.5482	-6.8310					
203	H	-3.7449	-8.3147	-7.0850					
204	H	6.8995	-6.5173	1.5357					
205	H	8.5123	-7.0559	2.1122					
206	H	8.2030	-5.3149	1.8218					
207	H	4.8111	8.5869	-3.0168					
208	H	6.3109	9.4236	-3.5411					
209	H	6.4163	7.9683	-2.4997					

Table S30. Cartesian coordinates of [K(**C12**)]$^{+}$ at its optimized geometry.

Atom		x	y	z	Atom		x	y	z
1	N	-2.6416	-0.3535	0.8694	46	N	5.7678	4.9250	-3.5686
2	N	4.4509	-5.8150	3.8685	47	O	-0.0300	2.1957	-2.4763
3	O	-0.4420	-2.0036	1.9645	48	C	-4.6927	2.2833	-2.5659

4	C	-3.7339	-0.2301	0.0937
5	C	-4.9090	-0.9543	0.3467
6	C	-4.9487	-1.8181	1.4367
7	C	-3.8195	-1.9384	2.2434
8	C	-2.6861	-1.1877	1.9209
9	C	-1.4322	-1.3057	2.7524
10	C	1.7152	-3.5000	4.5747
11	C	0.8081	-2.6180	3.9780
12	C	0.4736	-2.7845	2.6305
13	C	1.0856	-3.8020	1.8825
14	C	1.9831	-4.6714	2.4946
15	C	2.2985	-4.5482	3.8577
16	C	3.1685	-5.5638	4.5728
17	C	10.0039	-1.5884	0.7056
18	C	8.8480	-1.7374	1.7097
19	C	8.3530	-3.1751	1.9065
20	C	7.2213	-3.2755	2.9394
21	C	6.4861	-4.6224	2.9106
22	C	5.4230	-4.6993	4.0085
23	N	2.3669	-1.0874	-1.3202
24	N	-5.9312	-4.9890	-3.6517
25	O	-0.1528	-2.3770	-2.2043
26	C	4.3261	-3.0391	-1.6890
27	C	4.4885	-2.0472	-0.7275
28	C	3.4845	-1.0789	-0.5705
29	C	3.1707	-3.0424	-2.4675
30	C	-2.7908	-3.2147	-4.6662
31	C	-1.6268	-2.6443	-4.1438
32	C	-1.2891	-2.8722	-2.8048
33	C	-2.1364	-3.6458	-1.9999
34	C	-3.2861	-4.2174	-2.5421
35	C	-3.6266	-4.0235	-3.8887
36	C	-4.7923	-4.7331	-4.5584
37	C	2.2156	-2.0461	-2.2487
38	C	0.9461	-2.0082	-3.0654
39	C	-6.7851	-3.8017	-3.3846
40	C	-7.4638	-3.8066	-2.0130
41	C	-8.1516	-2.4685	-1.7065
42	C	-8.6926	-2.3915	-0.2707
43	C	-9.1129	-0.9754	0.1517
44	C	-9.3360	-0.8360	1.6664
45	N	-2.4683	0.8523	-1.6739
49	C	-4.7985	1.4150	-1.4845
50	C	-3.6586	0.7139	-1.0619
51	C	-3.4594	2.4266	-3.1978
52	C	2.6547	3.3234	-4.7674
53	C	1.5018	2.6617	-4.3331
54	C	1.1134	2.7661	-2.9934
55	C	1.9083	3.4940	-2.0953
56	C	3.0469	4.1541	-2.5492
57	C	3.4296	4.0966	-3.8986
58	C	4.5796	4.9160	-4.4544
59	C	-2.3733	1.6871	-2.7209
60	C	-1.0369	1.7754	-3.4186
61	C	6.5254	3.6461	-3.5902
62	C	7.4126	3.3961	-2.3687
63	C	7.8942	1.9399	-2.3155
64	C	8.8806	1.6671	-1.1709
65	C	9.1049	0.1707	-0.9245
66	C	10.1381	-0.1500	0.1691
67	C	-9.4311	0.6121	2.1833
68	N	2.5042	0.4889	1.0071
69	N	-4.4465	6.3748	3.2461
70	O	0.4379	2.4297	1.5145
71	C	3.6283	0.0122	0.4415
72	C	4.8970	0.5100	0.7779
73	C	4.9940	1.5257	1.7234
74	C	3.8287	2.0191	2.3062
75	C	2.6034	1.4691	1.9193
76	C	1.3211	1.9583	2.5529
77	C	-1.7685	4.7642	3.3637
78	C	-0.8721	3.7155	3.1453
79	C	-0.4791	3.4047	1.8361
80	C	-1.0279	4.1123	0.7594
81	C	-1.9243	5.1517	0.9983
82	C	-2.2949	5.5100	2.3022
83	C	-3.1762	6.7182	2.5457
84	C	-8.2122	1.4719	1.8058
85	C	-7.9742	2.7014	2.6925
86	C	-6.8768	3.6087	2.1158
87	C	-6.3116	4.6369	3.1055
88	C	-5.4163	5.6465	2.3793
89	K	-0.0446	0.1112	-0.2949
90	C	-6.6304	-7.2681	-2.2860
91	S	-6.7563	-6.4586	-3.8837
92	O	-5.9975	-7.2772	-4.8463
93	O	-8.1807	-6.1888	-4.1513
94	C	-4.1511	7.6543	5.6263
95	S	-5.0951	7.6996	4.0996
96	O	-6.4996	7.3843	4.4056
97	O	-4.8051	8.9770	3.4173
139	H	1.0149	-2.7146	-3.8979
140	H	-7.5235	-3.6717	-4.1862
141	H	-6.0991	-2.9507	-3.4310
142	H	-6.7017	-4.0038	-1.2473
143	H	-8.2001	-4.6168	-1.9535
144	H	-8.9622	-2.2886	-2.4270
145	H	-7.4239	-1.6565	-1.8527

98	C	6.8990	6.8081	-1.9227	146	H	-7.9030	-2.7350	0.4140
99	S	6.7145	6.3429	-3.6469	147	H	-9.5298	-3.0932	-0.1454
100	O	8.0507	6.0360	-4.1908	148	H	-10.017	-0.6663	-0.3933
101	O	5.9138	7.3951	-4.2981	149	H	-8.3196	-0.2836	-0.1589
102	C	5.4340	-7.9108	2.3930	150	H	-8.4963	-1.3231	2.1847
103	S	5.0944	-7.3815	4.0748	151	H	-10.237	-1.3916	1.9605
104	O	4.0264	-8.2523	4.5979	152	H	-5.5571	2.8464	-2.9055
105	O	6.3737	-7.3167	4.8066	153	H	-5.7423	1.2984	-0.9645
106	H	-5.7662	-0.8584	-0.3096	154	H	-3.3361	3.0970	-4.0423
107	H	-5.8441	-2.3949	1.6484	155	H	2.9406	3.2433	-5.8137
108	H	-3.8091	-2.6026	3.1018	156	H	0.9264	2.0766	-5.0413
109	H	-1.6465	-1.8683	3.6645	157	H	1.6115	3.5516	-1.0521
110	H	-1.0499	-0.3156	3.0285	158	H	3.6406	4.7324	-1.8465
111	H	1.9582	-3.3703	5.6268	159	H	4.2577	5.9519	-4.5794
112	H	0.3746	-1.8172	4.5670	160	H	4.8457	4.5399	-5.4523
113	H	0.8269	-3.9152	0.8334	161	H	-0.7600	0.7983	-3.8353
114	H	2.4331	-5.4686	1.9088	162	H	-1.1001	2.4965	-4.2381
115	H	3.3546	-5.2254	5.6016	163	H	7.1123	3.5649	-4.5151
116	H	2.6400	-6.5166	4.6375	164	H	5.7535	2.8713	-3.6249
117	H	9.8486	-2.2716	-0.1430	165	H	6.8431	3.6220	-1.4575
118	H	10.9411	-1.9043	1.1833	166	H	8.2800	4.0665	-2.3866
119	H	7.9953	-1.1289	1.3763	167	H	8.3627	1.6674	-3.2723
120	H	9.1575	-1.3148	2.6769	168	H	7.0187	1.2834	-2.2047
121	H	9.1864	-3.8269	2.2068	169	H	8.4990	2.1230	-0.2450
122	H	7.9969	-3.5594	0.9389	170	H	9.8381	2.1641	-1.3849
123	H	6.4914	-2.4733	2.7549	171	H	9.4135	-0.3179	-1.8606
124	H	7.6274	-3.0942	3.9451	172	H	8.1375	-0.2783	-0.6569
125	H	7.2111	-5.4372	3.0238	173	H	11.1481	0.0132	-0.2305
126	H	6.0019	-4.7491	1.9332	174	H	10.0235	0.5550	1.0063
127	H	4.8091	-3.7948	3.9615	175	H	-9.5281	0.5778	3.2781
128	H	5.8937	-4.7231	5.0005	176	H	-10.348	1.0910	1.8107
129	H	5.0870	-3.8021	-1.8247	177	H	5.7873	0.1261	0.2928
130	H	5.3706	-2.0364	-0.0978	178	H	5.9635	1.9341	1.9933
131	H	3.0071	-3.7997	-3.2274	179	H	3.8627	2.8161	3.0422
132	H	-3.0390	-3.0342	-5.7099	180	H	1.5456	2.7699	3.2494
133	H	-1.0000	-2.0308	-4.7817	181	H	0.8298	1.1478	3.1065
134	H	-1.8717	-3.8112	-0.9592	182	H	-2.0522	5.0079	4.3846
135	H	-3.9205	-4.8341	-1.9120	183	H	-0.4830	3.1617	3.9925
136	H	-4.4515	-5.6984	-4.9390	184	H	-0.7325	3.8533	-0.2528
137	H	-5.1222	-4.1506	-5.4301	185	H	-2.3254	5.7092	0.1552
138	H	0.7795	-1.0023	-3.4705	186	H	-2.6325	7.4350	3.1676
187	H	-3.3899	7.2210	1.5938					
188	H	-8.3070	1.8012	0.7619					
189	H	-7.3063	0.8483	1.8441					
190	H	-7.6924	2.3675	3.7020					
191	H	-8.9040	3.2775	2.8069					
192	H	-7.2770	4.1275	1.2319					
193	H	-6.0475	2.9850	1.7499					
194	H	-5.7186	4.1209	3.8723					

195	H	-7.1220	5.1579	3.6234					
196	H	-6.0209	6.3447	1.7842					
197	H	-4.7855	5.1010	1.6712					
198	H	-5.5741	-7.4275	-2.0606					
199	H	-7.1521	-8.2258	-2.3698					
200	H	-7.0997	-6.6468	-1.5219					
201	H	-3.0867	7.7634	5.4087					
202	H	-4.3501	6.7070	6.1305					
203	H	-4.4940	8.4950	6.2358					
204	H	5.9061	6.9872	-1.5054					
205	H	7.4937	7.7258	-1.9036					
206	H	7.4109	6.0142	-1.3781					
207	H	4.4966	-7.9075	1.8336					
208	H	5.8391	-8.9248	2.4550					
209	H	6.1618	-7.2410	1.9339					

Table S31. Cartesian coordinates of [Li(**C12-NO**)]$^{+}$ at its optimized geometry.

Atom		x	y	z	Atom		x	y	z
1	N	2.2557	-0.1217	0.0221	46	N	4.0850	-6.5877	4.2402
2	N	-3.0852	5.5932	5.5526	47	O	-1.1418	-3.0830	2.9711
3	O	1.5212	2.6491	2.2066	48	C	-3.3954	0.4063	2.8707
4	C	4.3754	1.5571	-0.5660	49	C	-3.4606	1.1038	1.6664
5	C	4.2051	0.3640	-1.2651	50	C	-2.9032	0.5642	0.5129
6	C	3.1320	-0.4680	-0.9737	51	C	-2.7419	-0.8223	2.9042
7	C	3.4612	1.8918	0.4272	52	C	1.1999	-5.8652	2.3000
8	C	-0.1780	5.1073	4.2699	53	C	0.2313	-4.8868	2.0522
9	C	0.6445	4.5243	3.3141	54	C	-0.2087	-4.0780	3.1045
10	C	0.6678	3.1283	3.1649	55	C	0.3109	-4.2667	4.3950
11	C	-0.1351	2.3286	3.9833	56	C	1.2723	-5.2450	4.6206
12	C	-0.9492	2.9355	4.9472	57	C	1.7379	-6.0594	3.5744
13	C	-0.9914	4.3233	5.1059	58	C	2.7669	-7.1425	3.8154
14	C	-1.8708	4.9694	6.1553	59	C	-2.1969	-1.3534	1.7425
15	C	2.3999	1.0437	0.7171	60	C	-1.4503	-2.6543	1.6488
16	C	1.3713	1.2973	1.7841	61	C	4.7961	-5.8070	3.1871
17	C	-4.1439	4.6044	5.2212	62	C	5.5724	-4.5982	3.7188
18	C	-5.1184	5.0159	4.1138	63	C	5.9909	-3.6495	2.5847
19	C	-5.9753	3.8172	3.6808	64	C	7.0211	-2.5956	3.0174
20	C	-7.0225	4.1399	2.6058	65	C	7.2283	-1.4869	1.9750
21	C	-7.7153	2.8781	2.0729	66	C	8.4250	-0.5690	2.2788
22	C	-8.8260	3.1424	1.0448	67	N	1.8202	-1.9265	-2.4417
23	N	-1.7975	1.5772	-1.4261	68	N	-7.0485	-3.8914	-4.1850
24	N	6.2697	4.2769	-4.8557	69	O	-0.6878	-3.5881	-3.1380
25	O	0.2193	3.5167	-2.5844	70	C	2.9403	-1.7625	-1.6676
26	C	-2.9729	1.2605	-0.7909	71	C	3.9026	-2.7660	-1.6198
27	C	-4.1852	1.6706	-1.3364	72	C	3.7291	-3.9439	-2.3407
28	C	-4.2175	2.3733	-2.5367	73	C	2.5791	-4.0862	-3.1122
29	C	-3.0120	2.6499	-3.1760	74	C	1.6278	-3.0755	-3.1637
30	C	-1.8029	2.2477	-2.6222	75	C	0.3821	-3.1629	-3.9934

31	C	-0.4801	2.4865	-3.2915	76	C	-3.5986	-3.4293	-5.4218
32	C	3.3395	3.8334	-4.5588	77	C	-2.2781	-3.2868	-4.9807
33	C	2.0582	3.3958	-4.2056	78	C	-1.9399	-3.6898	-3.6844
34	C	1.4733	3.8511	-3.0165	79	C	-2.9270	-4.2421	-2.8518
35	C	2.1901	4.7266	-2.1871	80	C	-4.2301	-4.3900	-3.3139
36	C	3.4702	5.1359	-2.5465	81	C	-4.5902	-3.9850	-4.6095
37	C	4.0666	4.7041	-3.7416	82	C	-5.9827	-4.2097	-5.1678
38	C	5.4282	5.2535	-4.1307	83	C	-9.3396	1.8589	0.3625
39	C	8.4119	0.7692	1.5188	84	C	-8.4089	1.3374	-0.7445
40	C	8.3582	0.6554	-0.0117	85	C	-8.7224	-0.0913	-1.2079
41	C	8.2466	2.0185	-0.7139	86	C	-7.8590	-0.5437	-2.3952
42	C	7.8342	1.9097	-2.1889	87	C	-7.9683	-2.0479	-2.6850
43	C	7.5165	3.2610	-2.8521	88	C	-7.2167	-2.4304	-3.9632
44	C	6.6847	3.0571	-4.1260	89	O	1.2604	-0.9300	0.3281
45	N	-2.4683	0.8523	-1.6739	90	O	0.9443	-0.9548	-2.5238
91	O	-1.8846	-1.2464	-0.5386	139	H	1.5306	2.7151	-4.8643
92	O	-0.6572	1.2548	-0.8742	140	H	1.7335	5.0677	-1.2626
93	Li	-0.1341	-0.6009	-0.9912	141	H	4.0147	5.8086	-1.8877
94	C	-3.7818	8.1686	4.9270	142	H	5.2872	6.1182	-4.7882
95	S	-3.6281	7.0238	6.3019	143	H	5.9390	5.6310	-3.2366
96	O	-4.9686	6.8210	6.8841	144	H	7.5386	1.3495	1.8533
97	O	-2.5509	7.5288	7.1723	145	H	9.2958	1.3549	1.8100
98	C	-9.0225	-5.2233	-2.7626	146	H	7.4876	0.0489	-0.2954
99	S	-8.5082	-4.7503	-4.4379	147	H	9.2419	0.1129	-0.3792
100	O	-8.1695	-5.9863	-5.1661	148	H	9.1937	2.5699	-0.6234
101	O	-9.5358	-3.8593	-5.0120	149	H	7.4915	2.6218	-0.1886
102	C	4.4226	-7.6102	6.7373	150	H	6.9381	1.2746	-2.2458
103	S	5.0395	-7.7380	5.0563	151	H	8.6122	1.3875	-2.7635
104	O	4.7647	-9.1052	4.5706	152	H	8.4433	3.8011	-3.0855
105	O	6.4322	-7.2621	5.0289	153	H	6.9545	3.8847	-2.1449
106	C	6.4356	5.3811	-7.3356	154	H	5.7560	2.5364	-3.8707
107	S	7.4168	4.9670	-5.8886	155	H	7.2223	2.4249	-4.8351
108	O	8.3633	3.9021	-6.2611	156	H	-3.8339	0.8228	3.7708
109	O	7.9539	6.2242	-5.3322	157	H	-3.9280	2.0799	1.6064
110	H	5.2051	2.2153	-0.7956	158	H	-2.6405	-1.3859	3.8234
111	H	4.8899	0.0680	-2.0513	159	H	1.5369	-6.4893	1.4759
112	H	3.5511	2.8107	0.9923	160	H	-0.1648	-4.7717	1.0498
113	H	-0.1878	6.1900	4.3729	161	H	-0.0437	-3.6334	5.2029
114	H	1.2762	5.1318	2.6725	162	H	1.6728	-5.3753	5.6230
115	H	-0.1324	1.2487	3.8903	163	H	2.8813	-7.7541	2.9115
116	H	-1.5576	2.3044	5.5907	164	H	2.4154	-7.8062	4.6106
117	H	-1.3191	5.7558	6.6703	165	H	-2.0558	-3.4035	1.1201
118	H	-2.1697	4.2253	6.9057	166	H	-0.5327	-2.5026	1.0711
119	H	0.3664	1.1292	1.3847	167	H	4.0058	-5.4533	2.5191
120	H	1.5318	0.5969	2.6152	168	H	5.4502	-6.4519	2.5856
121	H	-4.6941	4.3172	6.1273	169	H	6.4558	-4.9422	4.2657
122	H	-3.5968	3.7207	4.8810	170	H	4.9402	-4.0583	4.4362
123	H	-4.5502	5.3946	3.2539	171	H	5.0953	-3.1494	2.1870
124	H	-5.7707	5.8271	4.4584	172	H	6.4109	-4.2318	1.7509

125	H	-6.4821	3.3941	4.5604	173	H	7.9785	-3.0964	3.2234
126	H	-5.3088	3.0280	3.3032	174	H	6.7060	-2.1378	3.9669
127	H	-6.5394	4.6667	1.7695	175	H	6.3095	-0.8837	1.9159
128	H	-7.7704	4.8338	3.0167	176	H	7.3568	-1.9432	0.9834
129	H	-8.1429	2.3147	2.9158	177	H	8.4417	-0.3484	3.3561
130	H	-6.9507	2.2240	1.6306	178	H	9.3581	-1.1110	2.0656
131	H	-8.4616	3.8396	0.2752	179	H	4.7802	-2.6067	-1.0031
132	H	-9.6596	3.6521	1.5474	180	H	4.4737	-4.7314	-2.3008
133	H	-5.0969	1.4192	-0.8066	181	H	2.4007	-4.9840	-3.6944
134	H	-5.1613	2.6905	-2.9662	182	H	0.5339	-3.8949	-4.7939
135	H	-2.9879	3.1847	-4.1193	183	H	0.1630	-2.1856	-4.4306
136	H	-0.6712	2.7961	-4.3247	184	H	-3.8510	-3.1052	-6.4289
137	H	0.1112	1.5637	-3.2977	185	H	-1.5390	-2.8594	-5.6487
138	H	3.7764	3.4813	-5.4894	186	H	-2.6518	-4.5574	-1.8495
187	H	-4.9810	-4.8241	-2.6593					
188	H	-6.1079	-3.6160	-6.0847					
189	H	-6.1013	-5.2593	-5.4443					
190	H	-10.334	2.0401	-0.0679					
191	H	-9.4763	1.0790	1.1268					
192	H	-7.3678	1.3650	-0.3934					
193	H	-8.4586	2.0238	-1.6030					
194	H	-9.7849	-0.1720	-1.4805					
195	H	-8.5699	-0.7792	-0.3629					
196	H	-6.8057	-0.2977	-2.1967					
197	H	-8.1453	0.0285	-3.2895					
198	H	-9.0268	-2.3236	-2.7683					
199	H	-7.5527	-2.6089	-1.8379					
200	H	-6.1959	-2.0416	-3.8992					
201	H	-7.6907	-1.9646	-4.8385					
202	H	-2.8011	8.2821	4.4610					
203	H	-4.1218	9.1217	5.3418					
204	H	-4.5095	7.7877	4.2093					
205	H	-8.3134	-4.8369	-2.0314					
206	H	-9.0364	-6.3148	-2.7345					
207	H	-10.022	-4.8237	-2.5866					
208	H	3.3552	-7.8393	6.7567					
209	H	4.6088	-6.5977	7.0996					
210	H	4.9718	-8.3423	7.3357					
211	H	5.6363	6.0709	-7.0570					
212	H	6.0251	4.4598	-7.7525					
213	H	7.1090	5.8619	-8.0505					

Table S32. Cartesian coordinates of [Na(**C12-NO**)]$^{+}$ at its optimized geometry.

Atom		x	y	z	Atom		x	y	z
1	N	-2.0576	-1.2090	0.1049	46	N	3.1131	-1.0125	4.7309
2	N	4.6756	-7.2528	-1.8879	47	O	2.0987	0.2167	-1.5376
3	O	-1.1351	-4.5098	-0.9992	48	C	2.4138	0.1137	-6.1072
4	C	-4.5481	-2.4300	0.1597	49	C	1.1400	0.6491	-6.2860
5	C	-4.3795	-1.1413	0.6626	50	C	0.4964	1.2928	-5.2356
6	C	-3.1301	-0.5308	0.6348	51	C	3.0072	0.2127	-4.8503
7	C	-3.4419	-3.0991	-0.3580	52	C	2.9522	1.2973	1.8919
8	C	2.2244	-5.6862	-2.0197	53	C	2.5714	1.3723	0.5497
9	C	1.1243	-4.8338	-1.8787	54	C	2.5230	0.2035	-0.2133
10	C	-0.0110	-5.2775	-1.1940	55	C	2.8478	-1.0287	0.3608
11	C	-0.0424	-6.5754	-0.6689	56	C	3.2245	-1.0885	1.7029
12	C	1.0678	-7.4036	-0.8026	57	C	3.2882	0.0738	2.4848
13	C	2.2229	-6.9723	-1.4741	58	C	3.7738	0.0432	3.9211
14	C	3.3962	-7.9317	-1.5922	59	C	2.3640	0.8773	-3.8140
15	C	-2.1997	-2.4811	-0.3797	60	C	2.9177	1.0152	-2.4309
16	C	-0.9325	-3.1041	-0.8935	61	C	1.6702	-0.7593	5.0060
17	C	5.2290	-6.4515	-0.7634	62	C	0.8037	-2.0210	5.0956
18	C	6.1302	-5.2913	-1.1994	63	C	-0.6831	-1.6924	4.8953
19	C	6.3163	-4.2453	-0.0903	64	C	-1.6306	-2.8452	5.2616
20	C	7.3473	-3.1688	-0.4617	65	C	-3.0600	-2.6162	4.7499
21	C	7.3104	-1.9307	0.4448	66	C	-4.1005	-3.6081	5.2961
22	C	8.4610	-0.9488	0.1710	67	N	-2.3870	1.8167	0.5481
23	N	-1.8346	1.2639	-4.5456	68	N	4.9791	7.3357	0.1875
24	N	-10.248	0.3936	0.2235	69	O	-0.9092	5.0711	1.0473
25	O	-5.3221	1.6672	-3.7028	70	C	-2.8745	0.7697	1.2904
26	C	-0.8702	1.8446	-5.3365	71	C	-3.1389	0.9528	2.6440
27	C	-1.2131	2.8626	-6.2161	72	C	-2.8627	2.1705	3.2598
28	C	-2.5267	3.3238	-6.2754	73	C	-2.2926	3.1883	2.5009
29	C	-3.4789	2.7427	-5.4430	74	C	-2.0513	2.9996	1.1470
30	C	-3.1257	1.7113	-4.5815	75	C	-1.4058	4.0096	0.2382
31	C	-4.0761	0.9772	-3.6776	76	C	1.7777	6.5079	-1.0493
32	C	-8.6620	1.4527	-2.2891	77	C	0.6173	5.7968	-0.7215
33	C	-7.5302	1.9231	-2.9441	78	C	0.1947	5.7555	0.6104
34	C	-6.3788	1.1223	-3.0242	79	C	0.9117	6.4542	1.5940
35	C	-6.3763	-0.1471	-2.4384	80	C	2.0577	7.1590	1.2473
36	C	-7.5287	-0.6048	-1.7892	81	C	2.5180	7.1890	-0.0799
37	C	-8.6830	0.1763	-1.7011	82	C	3.7773	7.9436	-0.4486
38	C	-9.9266	-0.3557	-1.0194	83	C	8.2954	0.4345	0.8276
39	C	-5.4098	-3.6497	4.4868	84	C	7.3185	1.3598	0.0862
40	C	-6.1319	-2.3020	4.3394	85	C	7.1587	2.7425	0.7336
41	C	-7.3607	-2.3701	3.4189	86	C	6.3154	3.7060	-0.1126
42	C	-7.8812	-0.9889	2.9951	87	C	6.1216	5.0867	0.5317
43	C	-8.9989	-1.0474	1.9422	88	C	5.3898	6.0411	-0.4160
44	C	-9.2572	0.3335	1.3229	89	O	-0.8699	-0.6617	0.0942
45	N	1.1392	1.4524	-4.0344	90	O	-2.2458	1.7064	-0.7459
91	O	0.5730	2.1541	-3.0919	139	H	-7.5189	2.9076	-3.4029
92	O	-1.5327	0.2546	-3.7699	140	H	-5.5014	-0.7850	-2.4786

93	Na	-0.4646	0.6637	-1.7230
94	C	6.1833	-7.1283	-4.1805
95	S	5.8054	-8.1711	-2.7688
96	O	7.0382	-8.3405	-1.9780
97	O	5.1247	-9.3721	-3.2844
98	C	6.5172	8.1519	2.3071
99	S	6.2449	8.4142	0.5515
100	O	5.7449	9.7912	0.3883
101	O	7.4699	8.0172	-0.1679
102	C	5.1270	-2.7283	5.3451
103	S	4.0515	-1.4875	6.0705
104	O	4.8942	-0.3760	6.5544
105	O	3.1535	-2.1450	7.0331
106	C	-12.622	1.5708	-0.3886
107	S	-11.875	0.3335	0.6793
108	O	-11.957	0.8148	2.0690
109	O	-12.480	-0.9721	0.3487
110	H	-5.5230	-2.9045	0.1751
111	H	-5.2129	-0.5864	1.0781
112	H	-3.5191	-4.1081	-0.7442
113	H	3.1005	-5.3316	-2.5542
114	H	1.1647	-3.8358	-2.3025
115	H	-0.9279	-6.9090	-0.1358
116	H	1.0413	-8.3993	-0.3653
117	H	3.2072	-8.6445	-2.3977
118	H	3.4742	-8.5179	-0.6648
119	H	-0.6758	-2.6628	-1.8674
120	H	-0.1096	-2.8824	-0.2053
121	H	5.7495	-7.1001	-0.0470
122	H	4.3509	-6.0444	-0.2551
123	H	5.6843	-4.8021	-2.0753
124	H	7.1099	-5.6746	-1.5095
125	H	6.6218	-4.7370	0.8445
126	H	5.3454	-3.7710	0.1140
127	H	7.1768	-2.8467	-1.4998
128	H	8.3535	-3.6130	-0.4432
129	H	7.3481	-2.2403	1.5001
130	H	6.3435	-1.4260	0.3097
131	H	8.5734	-0.8120	-0.9153
132	H	9.3986	-1.4069	0.5156
133	H	-0.4340	3.2924	-6.8358
134	H	-2.8002	4.1241	-6.9544
135	H	-4.5110	3.0702	-5.4498
136	H	-3.6763	0.9371	-2.6572
137	H	-4.1856	-0.0549	-4.0376
138	H	-9.5454	2.0845	-2.2347
187	H	2.6122	7.6847	2.0207
188	H	3.8927	7.9643	-1.5407
189	H	3.7131	8.9765	-0.1046

141	H	-7.5204	-1.5988	-1.3486
142	H	-10.781	-0.2536	-1.6945
143	H	-9.8083	-1.4258	-0.8144
144	H	-5.1813	-4.0315	3.4802
145	H	-6.0921	-4.3817	4.9424
146	H	-5.4327	-1.5637	3.9244
147	H	-6.4241	-1.9225	5.3298
148	H	-8.1641	-2.9442	3.9030
149	H	-7.0897	-2.9311	2.5120
150	H	-7.0382	-0.4139	2.5830
151	H	-8.2287	-0.4298	3.8756
152	H	-9.9224	-1.4347	2.3922
153	H	-8.7090	-1.7544	1.1543
154	H	-8.3265	0.7222	0.8965
155	H	-9.5794	1.0410	2.0895
156	H	2.9189	-0.3939	-6.9214
157	H	0.6159	0.5549	-7.2307
158	H	3.9799	-0.2251	-4.6519
159	H	2.9833	2.2074	2.4858
160	H	2.3021	2.3282	0.1099
161	H	2.7995	-1.9285	-0.2454
162	H	3.4738	-2.0488	2.1457
163	H	3.6330	1.0305	4.3797
164	H	4.8500	-0.1561	3.9260
165	H	3.9449	0.6405	-2.4084
166	H	2.9045	2.0630	-2.1181
167	H	1.3336	-0.1627	4.1529
168	H	1.5376	-0.1332	5.8985
169	H	0.9574	-2.5077	6.0632
170	H	1.1284	-2.7316	4.3241
171	H	-0.8429	-1.4055	3.8453
172	H	-0.9526	-0.8104	5.4951
173	H	-1.6371	-2.9728	6.3540
174	H	-1.2477	-3.7888	4.8455
175	H	-3.0474	-2.6734	3.6511
176	H	-3.3677	-1.5908	4.9974
177	H	-3.6680	-4.6192	5.2993
178	H	-4.3160	-3.3657	6.3471
179	H	-3.5405	0.1179	3.2069
180	H	-3.0657	2.3131	4.3156
181	H	-2.0233	4.1404	2.9407
182	H	-2.1216	4.3846	-0.5055
183	H	-0.5911	3.5224	-0.3087
184	H	2.1052	6.5297	-2.0860
185	H	0.0601	5.2902	-1.5022
186	H	0.5641	6.4207	2.6224

190	H	9.2754	0.9304	0.8704					
191	H	7.9714	0.3098	1.8720					
192	H	6.3308	0.8830	0.0098					
193	H	7.6770	1.4883	-0.9464					
194	H	8.1526	3.1850	0.8968					
195	H	6.7040	2.6313	1.7291					
196	H	5.3304	3.2563	-0.3056					
197	H	6.7953	3.8297	-1.0946					
198	H	7.1021	5.4987	0.7988					
199	H	5.5450	4.9882	1.4610					
200	H	4.4538	5.5696	-0.7283					
201	H	5.9861	6.2126	-1.3217					
202	H	5.2595	-6.9475	-4.7335					
203	H	6.8993	-7.6764	-4.7994					
204	H	6.6195	-6.1892	-3.8380					
205	H	5.5951	8.3917	2.8402					
206	H	7.3216	8.8274	2.6116					
207	H	6.8066	7.1150	2.4834					
208	H	5.7065	-2.2837	4.5332					
209	H	4.5093	-3.5489	4.9760					
210	H	5.7970	-3.0748	6.1367					
211	H	-12.466	1.3015	-1.4350					
212	H	-12.172	2.5397	-0.1653					
213	H	-13.692	1.5816	-0.1639					

Table S33. Cartesian coordinates of [K(**C12-NO**)]$^{+}$ at its optimized geometry.

Atom		x	y	z	Atom		x	y	z
1	N	-1.0464	-2.0118	1.1286	46	N	2.5064	3.2227	6.0663
2	N	7.3809	-5.1849	-0.3596	47	O	2.9079	0.4219	0.2253
3	O	1.0769	-4.9279	1.0423	48	C	3.8465	-1.8395	-3.1206
4	C	-2.0873	-3.4621	3.2542	49	C	2.9207	-1.9078	-4.1599
5	C	-2.5127	-2.1578	3.0084	50	C	1.7243	-1.2097	-4.0765
6	C	-1.9877	-1.4363	1.9450	51	C	3.5458	-1.0588	-2.0075
7	C	-1.1019	-4.0076	2.4375	52	C	2.0071	3.1473	2.5655
8	C	4.4263	-5.1763	-0.5090	53	C	1.9521	2.3382	1.4233
9	C	3.0734	-4.8550	-0.3670	54	C	2.8230	1.2493	1.3134
10	C	2.4018	-5.2030	0.8101	55	C	3.7091	0.9604	2.3652
11	C	3.0819	-5.8868	1.8242	56	C	3.7414	1.7705	3.4935
12	C	4.4331	-6.1858	1.6718	57	C	2.8992	2.8899	3.6099
13	C	5.1303	-5.8313	0.5076	58	C	3.0067	3.8283	4.7940
14	C	6.5978	-6.2012	0.3833	59	C	2.3551	-0.3455	-1.9461
15	C	-0.5782	-3.2719	1.3825	60	C	1.9517	0.5702	-0.8225
16	C	0.5331	-3.7361	0.4797	61	C	1.0179	3.1558	6.1163
17	C	7.5092	-3.8746	0.3329	62	C	0.4132	2.0491	6.9928
18	C	7.7199	-2.6828	-0.6075	63	C	-0.9763	1.6702	6.4606
19	C	7.3613	-1.3455	0.0569	64	C	-1.8128	0.7444	7.3575
20	C	7.6651	-0.1351	-0.8397	65	C	-2.9657	0.1110	6.5630
21	C	6.9561	1.1536	-0.4009	66	C	-4.0214	-0.6347	7.3947
22	C	7.3590	2.3820	-1.2334	67	N	-3.1890	0.3320	0.7303

23	N	-0.4654	-1.8901	-4.8871	68	N	-0.1368	6.7290	-3.6195
24	N	-8.2052	-2.7316	-0.2592	69	O	-4.7726	3.2678	-0.7763
25	O	-3.4152	-1.7494	-4.4494	70	C	-2.3079	-0.0035	1.7288
26	C	0.7096	-1.2288	-5.1541	71	C	-1.7972	0.9818	2.5625
27	C	0.9197	-0.6587	-6.4035	72	C	-2.1764	2.3123	2.3992
28	C	-0.0727	-0.7132	-7.3799	73	C	-3.0828	2.6257	1.3914
29	C	-1.2784	-1.3361	-7.0654	74	C	-3.5808	1.6337	0.5571
30	C	-1.4739	-1.9210	-5.8210	75	C	-4.5924	1.8755	-0.5386
31	C	-2.7425	-2.6006	-5.4085	76	C	-2.5564	4.1391	-3.6183
32	C	-6.5653	-2.1388	-2.6291	77	C	-3.3742	3.3617	-2.7909
33	C	-5.6104	-1.6834	-3.5387	78	C	-3.9068	3.9242	-1.6277
34	C	-4.3736	-2.3279	-3.6310	79	C	-3.6286	5.2612	-1.3113
35	C	-4.0971	-3.4343	-2.8185	80	C	-2.8193	6.0217	-2.1490
36	C	-5.0633	-3.8754	-1.9114	81	C	-2.2660	5.4731	-3.3165
37	C	-6.3054	-3.2373	-1.7999	82	C	-1.4243	6.3210	-4.2468
38	C	-7.3415	-3.7821	-0.8297	83	C	6.4146	3.5906	-1.0894
39	C	-4.8806	-1.6075	6.5628	84	C	5.1047	3.4445	-1.8790
40	C	-5.5957	-0.9813	5.3548	85	C	4.1656	4.6535	-1.7653
41	C	-6.2900	-2.0118	4.4494	86	C	2.9403	4.5450	-2.6839
42	C	-6.7289	-1.4259	3.0996	87	C	2.0114	5.7669	-2.6228
43	C	-7.3503	-2.4553	2.1437	88	C	0.8907	5.6544	-3.6596
44	C	-7.6129	-1.8384	0.7623	89	O	-0.5581	-1.3457	0.1180
45	N	1.4624	-0.4173	-2.9818	90	O	-3.6755	-0.5915	-0.0556
91	O	0.3709	0.2930	-2.9322	139	H	-5.8105	-0.8254	-4.1742
92	O	-0.6170	-2.4963	-3.7484	140	H	-3.1328	-3.9315	-2.8707
93	K	-1.9019	-0.8838	-2.1007	141	H	-4.8364	-4.7246	-1.2707
94	C	8.6237	-5.2492	-2.8097	142	H	-7.9810	-4.4999	-1.3540
95	S	8.7999	-5.7677	-1.0996	143	H	-6.8399	-4.3488	-0.0356
96	O	9.9795	-5.0884	-0.5323	144	H	-4.2274	-2.4150	6.1990
97	O	8.7562	-7.2406	-1.0840	145	H	-5.6213	-2.0884	7.2176
98	C	0.7436	9.0433	-2.4392	146	H	-4.8644	-0.4310	4.7482
99	S	0.4142	8.2898	-4.0355	147	H	-6.3264	-0.2343	5.6993
100	O	-0.7073	9.0302	-4.6413	148	H	-7.1560	-2.4471	4.9688
101	O	1.6852	8.2039	-4.7797	149	H	-5.5962	-2.8445	4.2608
102	C	4.8013	3.0297	7.5125	150	H	-5.8519	-0.9739	2.6126
103	S	3.2419	3.9175	7.4397	151	H	-7.4430	-0.6060	3.2655
104	O	3.5353	5.3503	7.2324	152	H	-8.2916	-2.8313	2.5645
105	O	2.4352	3.5420	8.6117	153	H	-6.6754	-3.3159	2.0440
106	C	-10.466	-2.9450	-1.7533	154	H	-6.6700	-1.4851	0.3301
107	S	-9.8334	-3.1282	-0.0819	155	H	-8.2691	-0.9703	0.8581
108	O	-10.438	-2.0859	0.7639	156	H	4.7814	-2.3861	-3.1778
109	O	-10.003	-4.5420	0.3064	157	H	3.1052	-2.5106	-5.0425
110	H	-2.5022	-4.0323	4.0783	158	H	4.2290	-0.9823	-1.1711
111	H	-3.2532	-1.6797	3.6385	159	H	1.3441	4.0071	2.6277
112	H	-0.7108	-5.0026	2.6091	160	H	1.2443	2.5760	0.6377
113	H	4.9417	-4.8990	-1.4240	161	H	4.3728	0.1057	2.2729
114	H	2.5543	-4.3393	-1.1686	162	H	4.4413	1.5390	4.2929
115	H	2.5504	-6.1486	2.7344	163	H	2.4656	4.7599	4.5840
116	H	4.9586	-6.6910	2.4789	164	H	4.0564	4.0955	4.9392

117	H	6.6995	-7.1466	-0.1538	165	H	1.9267	1.6029	-1.1951
118	H	7.0141	-6.3584	1.3886	166	H	0.9506	0.3123	-0.4600
119	H	0.1573	-3.9156	-0.5370	167	H	0.7356	2.9581	5.0780
120	H	1.2932	-2.9492	0.4141	168	H	0.5868	4.1338	6.3741
121	H	8.2977	-3.9145	1.0953	169	H	0.3562	2.3774	8.0342
122	H	6.5577	-3.7444	0.8551	170	H	1.0689	1.1690	6.9689
123	H	7.0860	-2.8148	-1.4943	171	H	-0.8413	1.1856	5.4837
124	H	8.7593	-2.6558	-0.9569	172	H	-1.5565	2.5846	6.2657
125	H	7.8970	-1.2410	1.0114	173	H	-2.2022	1.3121	8.2149
126	H	6.2899	-1.3573	0.3056	174	H	-1.1797	-0.0520	7.7756
127	H	7.3586	-0.3644	-1.8712	175	H	-2.5284	-0.5855	5.8321
128	H	8.7522	0.0281	-0.8752	176	H	-3.4578	0.8956	5.9721
129	H	7.1650	1.3535	0.6605	177	H	-3.5226	-1.2106	8.1876
130	H	5.8716	0.9930	-0.4714	178	H	-4.6658	0.0950	7.9066
131	H	7.4086	2.1013	-2.2965	179	H	-1.0988	0.6858	3.3358
132	H	8.3792	2.6780	-0.9520	180	H	-1.7819	3.0839	3.0511
133	H	1.8651	-0.1600	-6.5884	181	H	-3.4216	3.6426	1.2363
134	H	0.0845	-0.2653	-8.3549	182	H	-5.5701	1.4913	-0.2271
135	H	-2.0908	-1.3817	-7.7834	183	H	-4.3192	1.3269	-1.4422
136	H	-2.5227	-3.5766	-4.9695	184	H	-2.1535	3.6948	-4.5256
137	H	-3.3886	-2.7332	-6.2818	185	H	-3.6035	2.3409	-3.0771
138	H	-7.5202	-1.6266	-2.5574	186	H	-4.0617	5.6911	-0.4128
187	H	-2.6155	7.0585	-1.8928					
188	H	-1.2293	5.7739	-5.1789					
189	H	-1.9664	7.2306	-4.5072					
190	H	6.9325	4.4940	-1.4414					
191	H	6.1922	3.7607	-0.0252					
192	H	4.5682	2.5421	-1.5541					
193	H	5.3537	3.2867	-2.9393					
194	H	4.7211	5.5691	-2.0169					
195	H	3.8366	4.7683	-0.7220					
196	H	2.3695	3.6391	-2.4306					
197	H	3.2857	4.4110	-3.7195					
198	H	2.6069	6.6686	-2.8059					
199	H	1.5742	5.8605	-1.6199					
200	H	0.3335	4.7318	-3.4702					
201	H	1.3160	5.5771	-4.6696					
202	H	7.7160	-5.6997	-3.2159					
203	H	9.5046	-5.6143	-3.3455					
204	H	8.5746	-4.1611	-2.8613					
205	H	-0.1870	9.0644	-1.8689					
206	H	1.0964	10.0600	-2.6340					
207	H	1.5079	8.4728	-1.9102					
208	H	5.3788	3.2120	6.6040					
209	H	4.5907	1.9658	7.6343					
210	H	5.3421	3.4172	8.3803					
211	H	-9.9136	-3.5988	-2.4312					
212	H	-10.364	-1.9003	-2.0522					
213	H	-11.520	-3.2376	-1.7311					

References

1. D. Brynn Hibbert and P. Thordarson, *Chem. Commun.*, 2016, **52**, 12792–12805.
2. P. Thordarson, *Chem. Soc. Rev.*, 2011, **40**, 1305–1323.
3. Bindfit. http://app.supramolecular.org/bindfit/.
4. H. Bakirci, A. L. Koner and W. M. Nau, *Chem. Commun.*, 2005, 5411–5413.
5. D.-S. Guo, V. D. Uzunova, X. Su, Y. Liu and W. M. Nau, *Chem. Sci.*, 2011, **2**, 1722–1734.
6. D. C. Luehrs, R. T. Iwamoto and J. Kleinberg, *Inorg. Chem.*, 1966, **5**, 201–204.
7. J. Massaux, J. F. Desreux and G. Duyckaerts, *J. Chem. Soc., Dalton Trans.*, 1980, 865–868.
8. Y. Inoue, T. Hakushi, Y. Liu and L. H. Tong, *J. Org. Chem.*, 1993, **58**, 5411–5413.
9. H.-J. Buschmann, E. Cleve and E. Schollmeyer, *J. Solution Chem.*, 1994, **23**, 569–577.
10. K. E. Matthes, D. Parker, H. J. Buschmann and G. Ferguson, *Tetrahedron Lett.*, 1987, **28**, 5573–5576.
11. B. G. Cox, H. Schneider and J. Stroka, *J. Am. Chem. Soc.*, 1978, **100**, 4746–4749.
12. É. I. Sinyavskaya, L. V Tsymbal, K. B. Yatsimirskii, S. A. Pisareva, T. Y. Medved and M. I. Kabachnik, *Bull. Acad. Sci. USSR, Div. Chem. Sci.*, 1986, **35**, 160–165.
13. K. B. Yatsimirskii, M. I. Kabachnik, É. I. Sinyavskaya, T. Y. Medved', Yu. M. Polikarpov and B. K. Shcherbakov, *Russ. J. Inorg. Chem. (Engl. Transl.)*, 1984, **29**, 510–512.
14. K. B. Yatsimirskii, M. I. Kabachnik, É. I. Sinyavskaya, L. V Tsymbal, S. A. Pisareva and T. Y. Medved', *Bull. Acad. Sci. USSR, Div. Chem. Sci.*, 1988, **37**, 1874–1879.
15. N. Kihara, K. Saigo, Y. Kabata, M. Ohno and M. Hasegawa, *Chem. Lett.*, 1989, **18**, 1289–1292.
16. B. P. Czech, A. Czech, B. E. Knudsen and R. A. Bartsch, *Gazz. Chim. Ital.*, 1987, **117**, 717–721.
17. J. J. Rebek, J. E. Trend, R. V Wattley and S. Chakravorti, *J. Am. Chem. Soc.*, 1979, **101**, 4333–4337.
18. P. D. J. Grootenhuis, P. D. Van Der Wal and D. N. Reinhoudt, *Tetrahedron*, 1987, **43**, 397–404.
19. C. J. Chandler, L. W. Deady and J. A. Reiss, *Aust. J. Chem.*, 1988, **41**, 1051–1061.
20. F. Arnaud-Neu, E. M. Collins, M. Deasy, G. Ferguson, S. J. Harris, B. Kaitner, A. J. Lough, M. A. McKervey and E. Marques, *J. Am. Chem. Soc.*, 1989, **111**, 8681–8691.
21. P. J. Dijkstra, J. A. J. Brunink, K. E. Bugge, D. N. Reinhoudt, S. Harkema, R. Ungaro, F. Ugozzoli and E. Ghidini, *J. Am. Chem. Soc.*, 1989, **111**, 7567–7575.
22. D. J. Cram, R. A. Carmack and R. C. Helgeson, *J. Am. Chem. Soc.*, 1988, **110**, 571–577.
23. G. M. Lein and D. J. Cram, *J. Chem. Soc., Chem. Commun.*, 1982, 301–304.
24. D. J. Cram and G. M. Lein, *J. Am. Chem. Soc.*, 1985, **107**, 3657–3668.
25. D. J. Cram, I. B. Dicker, M. Lauer, C. B. Knobler and K. N. Trueblood, *J. Am. Chem. Soc.*, 1984, **106**, 7150–7167.
26. R. J. M. Nolte and D. J. Cram, *J. Am. Chem. Soc.*, 1984, **106**, 1416–1420.
27. R. C. Helgeson, T. L. Tarnowski and D. J. Cram, *J. Org. Chem.*, 1979, **44**, 2538–2550.
28. D. MacDougall, W. B. Crummett and et al., *Anal. Chem.*, 1980, **52**, 2242–2249.
29. M. D. Hanwell, D. E. Curtis, D. C. Lonie, T. Vandermeersch, E. Zurek and G. R. Hutchison, *J. Cheminform.*, 2012, **4**, 17.
30. M. J. Frisch, G. W. Trucks, H. B. Schlegel, G. E. Scuseria, M. A. Robb, J. R. Cheeseman, G. Scalmani, V. Barone, G. A. Petersson, H. Nakatsuji, X. Li, M. Caricato, A. V. Marenich, J. Bloino, B. G. Janesko, R. Gomperts, B. Mennucci, H. P. Hratchian, J. V. Ortiz, A. F. Izmaylov, J. L. Sonnenberg, D. Williams-Young, F. Ding, F. Lipparini, F. Egidi, J. Goings, B. Peng, A. Petrone, T. Henderson, D. Ranasinghe, V. G. Zakrzewski, J. Gao, N. Rega, G.

Zheng, W. Liang, M. Hada, M. Ehara, K. Toyota, R. Fukuda, J. Hasegawa, M. Ishida, T. Nakajima, Y. Honda, O. Kitao, H. Nakai, T. Vreven, K. Throssell, J. A. Montgomery, Jr., J. E. Peralta, F. Ogliaro, M. J. Bearpark, J. J. Heyd, E. N. Brothers, K. N. Kudin, V. N. Staroverov, T. A. Keith, R. Kobayashi, J. Normand, K. Raghavachari, A. P. Rendell, J. C. Burant, S. S. Iyengar, J. Tomasi, M. Cossi, J. M. Millam, M. Klene, C. Adamo, R. Cammi, J. W. Ochterski, R. L. Martin, K. Morokuma, O. Farkas, J. B. Foresman, and D. J. Fox, *Gaussian 16, Revision A.03*, 2016, Gaussian, Inc., Wallingford CT.
31. A. D. Becke, *J. Chem. Phys.*, 1993, **98**, 5648–5652.
32. C. Lee, W. Yang and R. G. Parr, *Phys. Rev. B*, 1988, **37**, 785–789.
33. P. J. Stephens, F. J. Devlin, C. F. Chabalowski and M. J. Frisch, *J. Phys. Chem.*, 1994, **98**, 11623–11627.
34. M. Caricato, G. Scalmani, G. W. Trucks and M. J. Frisch, *J. Phys. Chem. Lett.*, 2010, **1**, 2369–2373.
35. J. Tomasi, B. Mennucci and R. Cammi, *Chem. Rev.*, 2005, **105**, 2999–3094.
